# 3D minichannel-morphed adipose stem cell-cartilage granule reconstituted tough grafts for repairing segmental tracheal defects in preclinical models

**DOI:** 10.1038/s41467-026-77738-z

**Published:** 2026-09-11

**Authors:** Xinchi Zhang, Na Wei, Bing Liu, Pingping Yuan, Xiaopeng Wu, Zheqian Zhang, Yidong Hu, Pengyu Wang, Jingyi Zhang, Yimin Zhao, Wei Wu

**Affiliations:** 1 State Key Laboratory of Oral & Maxillofacial Reconstruction and Regeneration, National Clinical Research Center for Oral Diseases, Shaanxi Key Laboratory of Stomatology, Department of Oral & Maxillofacial Surgery, School of Stomatology, The Fourth Military Medical University, Xi’an, PR China; 2 State Key Laboratory of Oral & Maxillofacial Reconstruction and Regeneration, National Clinical Research Center for Oral Diseases, Shaanxi Key Laboratory of Stomatology, Digital Dentistry Center and Department of Prosthodontics, School of Stomatology, The Fourth Military Medical University, Xi’an, PR China; 3 State Key Laboratory of Oral & Maxillofacial Reconstruction and Regeneration, National Clinical Research Center for Oral Diseases, Shaanxi Engineering Research Center for Dental Materials and Advanced Manufacture, Department of Anesthesiology, School of Stomatology, The Fourth Military Medical University, Xi’an, PR China

**Keywords:** Regenerative medicine, Tissue engineering, Biomedical materials

## Abstract

Segmental tracheal reconstruction requires biomimetic substitutes that replicate both the rigid stenting of cartilage rings and the nutritional support of vascularized fascia. However, conventional tissue engineering is hampered by scarce donor chondrocytes and lengthy cell expansion. Here, we show a rapid tracheal reconstruction strategy using three-dimensional printed porous minichannel scaffolds filled with cartilage granules, platelet-rich plasma, and adipose-derived stem cells. Short-term ectopic incubation enables the grafts to acquire native-like mechanical resilience and transmural vascularization within two weeks. Upon orthotopic transplantation to repair segmental tracheal defects in rabbit and porcine models, these bioengineered grafts maintain airway patency and support preclinical animal survival for up to 115 days. Mechanistically, adipose-derived stem cells enhance cartilage granule survival within the hypoxic minichannels by upregulating glycolytic activity in chondrocytes. By eliminating the need for cell isolation and expansion, this approach offers a translatable solution for bioengineered airway repair.

## Abstract (web summary)

There, the authors develop a tracheal reconstruction strategy using 3D-printed minichannels with stem cell–cartilage composites in preclinical models. Bypassing cell expansion, these tough grafts achieve rapid vascularization and repair segmental airway defects.

## Introduction

Congenital stenosis, tracheoesophageal fistula, tumors, and acquired iatrogenic tracheal injuries pose significant threats to human life because of severe impairment of airway function^1–3^. Among tracheal repair techniques, end-to-end anastomosis is considered the gold standard and is widely employed in clinical practice^1,4^. However, when the length of the resected trachea exceeds 6 cm, the increased tension at the anastomosis site necessitates tracheal replacement^5^. A critical challenge in tracheal replacement lies in replicating the structural and functional integrity of tracheal walls, particularly the cartilage rings that provide stenting and the vascularized fascia that support them^6–9^. Recent advances in regenerative medicine have spurred the development of various tissue engineering strategies aimed at reconstructing cartilage rings, vascularized fascia, and ciliated epithelium^10–12^. While animal studies have demonstrated that chondrocyte-seeded polymeric scaffolds can regenerate sufficient cartilaginous extracellular matrix (ECM) to serve as airway stents and further form cartilage ring-stented vascularized fascia through prefabrication strategies, the clinical translation of these approaches remains limited. Key barriers include the scarcity of young, well-differentiated chondrocytes in humans and the lengthy, complex laboratory procedures required^7,13–15^.

Inspired by the use of minced cartilage techniques in articular cartilage repair^16–20^, we developed a mechanical processing technique to transform harvested cartilage tissues into granular cartilage. With this approach, the plasticity of minced cartilage is enhanced, which enables the repair of cartilaginous defects with unique anatomical morphologies and structures while avoiding the lengthy culture period for expanding chondrocytes. In our previous research, we demonstrated that starvation-pretreated mesenchymal stem cells (MSCs) increase the survival of cell bricks (comprising chondrocyte fragments and secreted cartilaginous ECM) in subcutaneously implanted constructs^21,22^. This effect is primarily mediated through the promotion of angiogenesis around the transplant site and the attenuation of chondrocyte apoptosis induced by starvation-activated autophagy^23,24^. The presence of host-derived platelet-rich plasma (PRP), known for its high biological efficacy, provides both the fluidity necessary for granular cartilage manipulation and essential nutritional support^25–27^. Nevertheless, engineering tracheal cartilage presents two critical challenges: the precise reconstruction of cartilage granules into C-shaped configurations and their effective integration with vascularized fascia^10,11,28^. To address these challenges, the development of advanced 3D in vivo models that can overcome the limitations associated with granular cartilage manipulation would significantly advance the field of regenerative tracheal graft construction.

In tracheal tissue engineering, biomaterial selection is key for scaffolds that need to provide immediate mechanical stability while gradually transferring load to regenerating tissue. Poly(ε-caprolactone) (PCL) offers excellent mechanical strength and a slow degradation profile, with a reported in vivo half-life of approximately 12–24 months, making it suitable for providing sustained, long-term airway support and maintaining lumen patency during the critical remodeling phase^29–31^. Poly (glycerol sebacate) (PGS) is a type of elastomer, which is known for its flexibility and relatively quick degradation (on the order of weeks to months), this allows for early cellular infiltration, vascular ingrowth, and tissue remodeling^32,33^. The PCL-PGS composite used in this study was designed to leverage the complementary properties of both polymers: PCL provides the necessary mechanical resilience to prevent graft collapse, while PGS enhances biocompatibility, creates transient porosity for host integration, and modulates the overall degradation of the construct. This material system shows promise in applications needing a balance between durability and tissue remodeling, like cardiac patches and vascular grafts^34,35^.

**Fig. 1:**
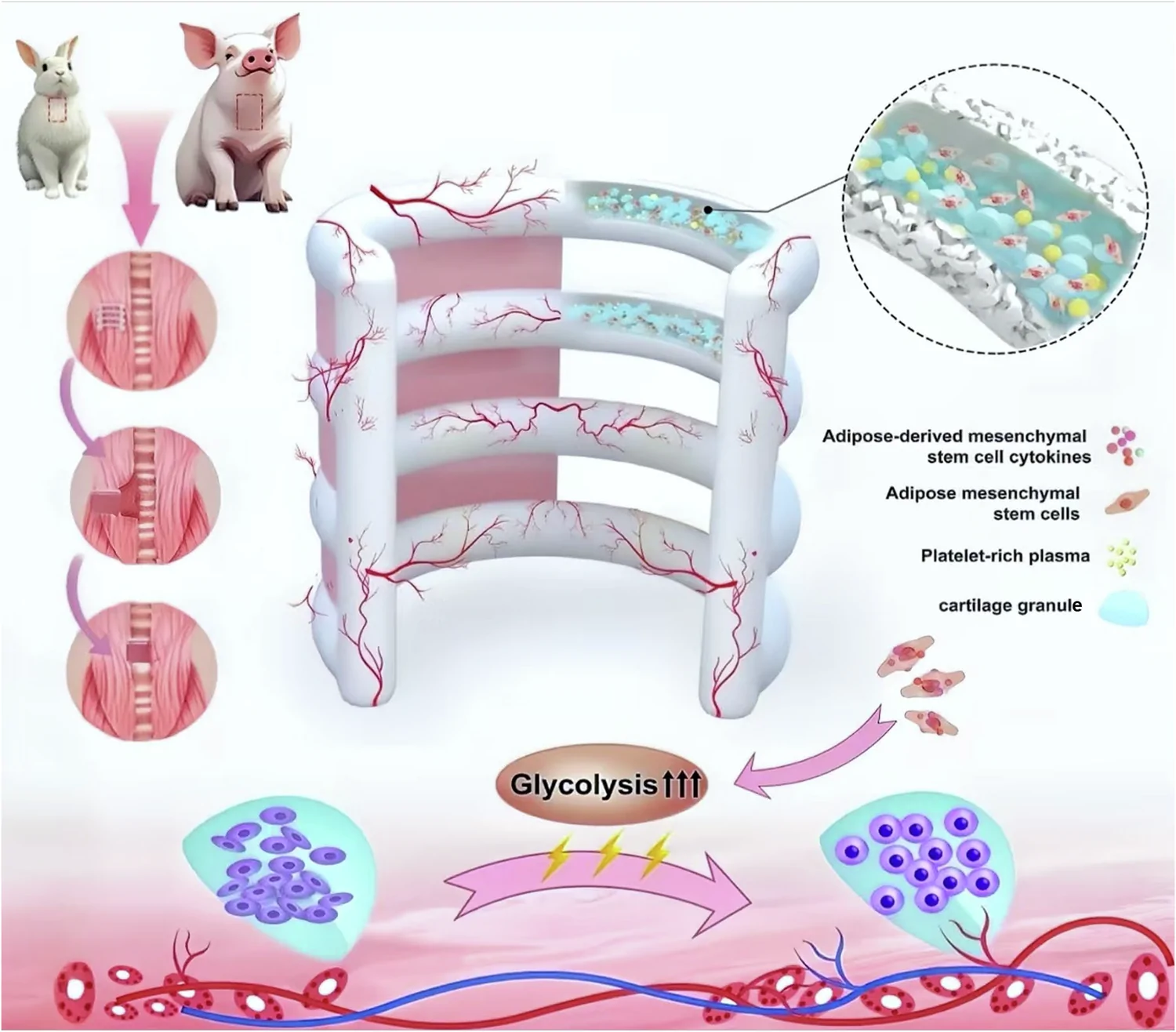
Schematic illustration of the injectable cartilage granule-filled 3D minichannel tracheal scaffold. A microporous, hollow-coated scaffold enables the C-shaped molding of cartilage granules. The addition of adipose stem cells (ADSCs) facilitates rapid vascularization of the graft and maturation of the cartilage rings. This biomimetic tracheal graft can be used in preclinical animal models to repair segmental tracheal defects.

In this study, we used a caramel template sacrificing approach to fabricate a 3D-shaped (PCL-PGS) minichannel, enabling the injection of adipose stem cell-cartilage granule-platelet-rich plasma constructs. These minichannels exhibit appropriate mechanical strength to resist tissue compression, whereas micropores in the walls facilitate nutrient perfusion and cellular communication between transplants and recipient tissues. By optimizing the ratio of adipose-derived mesenchymal stem cells (ADSCs) to cartilage granules, the constructs underwent rapid vascularization and integration with ingrown fascia, achieving mechanical properties comparable to those of natural tracheal cartilage (Fig. 1). Notably, this study represents the first instance of preparing tracheal cartilage ring structures using granular cartilage for the repair of segmental tracheal defects. This method addresses the challenges associated with the limited availability of young chondrocytes, prolonged cell culture during tracheal cartilage preparation, and the difficulty of achieving adequate graft revascularization, thereby offering key advantages for clinical translation, including reduced ex vivo manipulation, accelerated graft manufacturing, and enhanced potential for host integration and mechanical strength.

## Results

### Preparation of 3D shaped minichannels

**Fig. 2:**
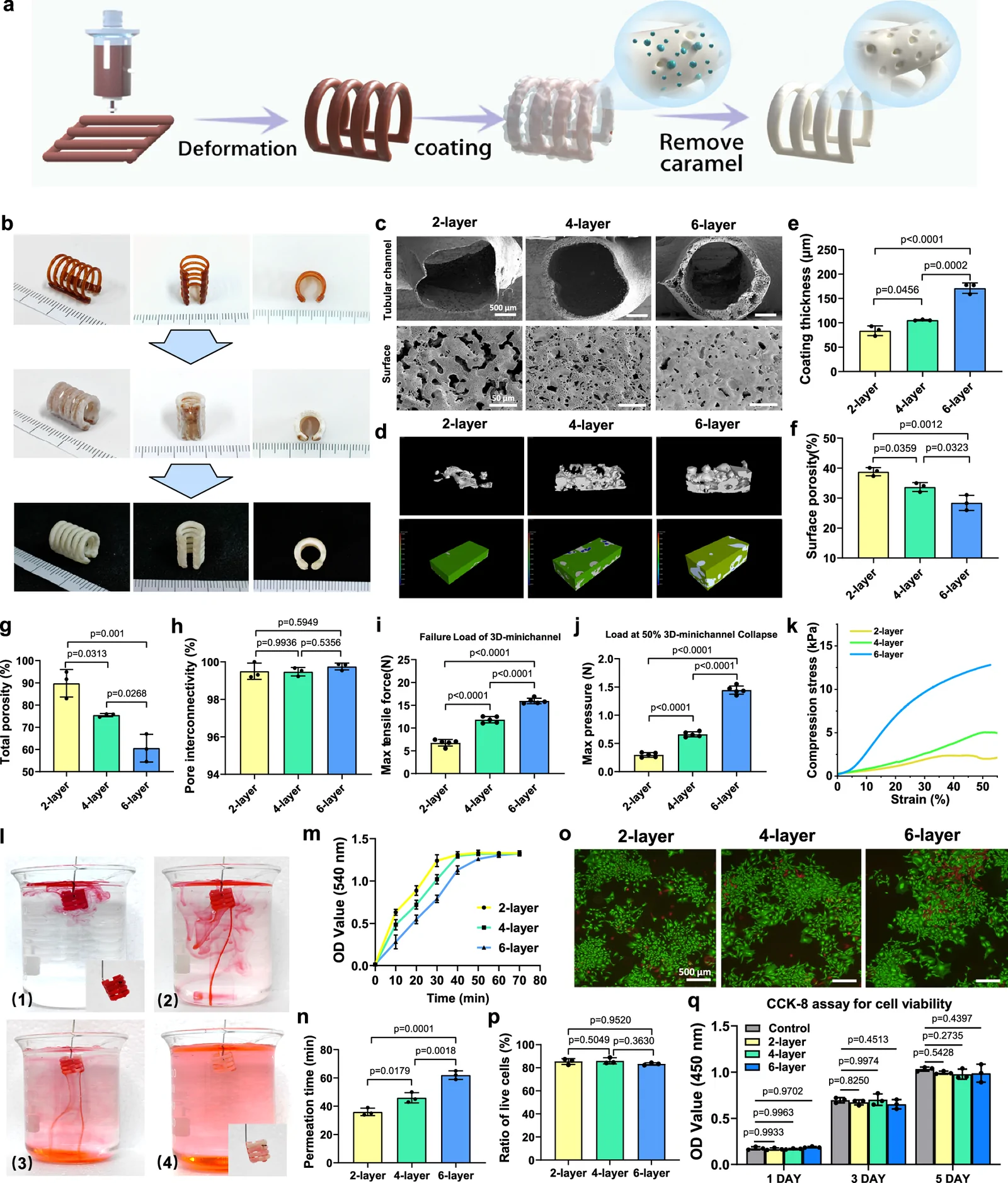
Characterization of 3D C-shaped minichannels. **a** Schematic illustration of the caramel template sacrificing approach for fabricating 3D C-shaped minichannel scaffolds. **b** Fabrication process of the 3D C-shaped minichannel scaffold. **c** SEM images of the microporous structure of minichannels with different coating layers (*n* = 3 independent experiments with similar results; scale bars: 500 μm and 50 μm). **d** Reconstructed nano-CT images of scaffolds with different coating layers. **e** Quantitative analysis of the thickness of different coating layers (*n* = 3 independent samples). **f** Surface porosity analysis of minichannels with different coating layers (*n* = 3 independent samples). **g** Quantification of total porosity (*n* = 3 independent samples) and **h** pore interconnectivity in 2-, 4-, and 6-layered scaffolds (*n* = 3 independent samples). **i** Max tensile force of minichannels for different coating layers (*n* = 5 independent samples). **j** Load required for 50% structural collapse in minichannel constructs for different coating layers (*n* = 5 independent samples). **k** Stress‒strain curves of minichannels in different groups. **l** Macroscopic view of red ink diffusion through minichannels. **m** Optical density progression over time for red ink diffusion through the scaffold layers (*n* = 3 independent samples). **n** Quantitative analysis of ink diffusion times across scaffolds with different coating layers (*n* = 3 independent samples). **o** Fluorescence images showing live cells (green) in the different groups of scaffolds at 3 days (*n* = 3 independent experiments with similar results; scale bars: 500 μm). **p** Quantitative evaluation of chondrocyte viability and cytocompatibility using live/dead staining across different groups (*n* = 3 independent biological samples). **q** CCK-8 assay results for cell proliferation in different groups (*n* = 3 independent biological samples). Data in (**e**−**j**, **m**, **n**, **p**, **q**) are presented as mean values ± SD. For (**e**−**j**, **m**, **n**, **p**), significance was determined via two-tailed one-way analysis of variance (ANOVA) followed by Tukey’s post-hoc test. For (**q**), significance was determined via two-tailed one-way ANOVA with Dunnett’s multiple comparisons test. Source data are provided as a Source Data file.

Figure 2a illustrates the fabrication process of a three-dimensional (3D) trachea-like minichannel scaffold utilizing the caramel template sacrificing technique. Initially, caramel, which served as the printing material, was precisely printed into a two-dimensional (2D) scaffold on the basis of predefined rectangular templates that matched the anatomical dimensions of the animal trachea (Fig. 2b). The pliable caramel structure was then molded into a 3D trachea-mimicking shape, as demonstrated in the Supplementary Materials (Suppl. Fig. 1, Suppl. Movie 1 and 2). Scanning electron microscopy (SEM) analysis confirmed that the 3D minichannels exhibited a hollow, microporous architecture, indicative of their permeability (Fig. 2c). FTIR spectroscopy further confirmed scaffold biosafety, with spectra showing characteristic PCL (1157.3 cm^−1^) and PGS (1190.1 cm^−1^) peaks and no detectable residual HFIP (C-F bonds at 1200–1250 cm^−1^; Suppl. Fig. 2). The mechanical properties of the scaffolds were dependent on the number of coating layers applied. Specifically, scaffolds with a 2-layer coating exhibited structural instability, leading to channel collapse, whereas scaffolds with 4-layer and 6-layer coatings maintained consistent circular cross-sections. Quantitative analysis revealed a direct correlation between the number of coating layers and wall thickness, with wall thicknesses measuring 83.72 ± 9.98 μm for the 2-layer scaffolds, 105.46 ± 1.21 μm for the 4-layer scaffolds, and 171.02 ± 10.69 μm for the 6-layer scaffolds (Fig. 2e). Increasing the number of coating layers reduced the superficial and total porosities (Fig. 2f, g). Specifically, the total porosity was 89.81 ± 6.18% for the 2-layer, 75.48 ± 0.73% for the 4-layer, and 60.60 ± 6.21% for the 6-layer minichannels. Nano-CT analysis revealed consistently high connectivity ( > 99%) across all groups (2-layer: 99.50 ± 0.43%; 4-layer: 99.48 ± 0.22%; 6-layer: 99.75 ± 0.19%; Fig. 2d, h), suggesting that increased coating layers did not compromise pore connectivity. The persistently high connectivity ( > 99%) across thickness variants confirmed the unique ability of 3D minichannels to preserve pore topology, a critical property favoring host integration. Mechanical testing demonstrated that both the axial and longitudinal strengths increased with additional coating layers. The maximum tensile stress values were 6.76 ± 0.73 N for the 2-layer scaffolds, 11.83 ± 0.69 N for the 4-layer scaffolds, and 15.94 ± 0.59 N for the 6-layer scaffolds (Fig. 2i). Compressive resistance, a critical factor for preventing graft collapse during muscle compression or neck movement, was also positively correlated with the number of coating layers. The load required to induce 50% structural collapse increased significantly across groups: 0.30 ± 0.04 N for the 2-layer scaffold, 0.66 ± 0.04 N for the 4-layer scaffold, and 1.44 ± 0.07 N for the 6-layer scaffold (Fig. 2j). Stress-strain analysis up to 50% strain further revealed distinct differences in compressive performance, showing a progressive enhancement with increasing coating layers (Fig. 2k).

Permeability analysis was conducted via red ink diffusion assays to evaluate the material exchange efficiency across the scaffold microarchitecture. As shown in Fig. 2l, micropores within the scaffold walls facilitated bidirectional diffusion between the scaffold interior and exterior. Quantitative assessment revealed a statistically inverse correlation between the number of coating layers and permeability. Specifically, the diffusion time increased progressively with increasing number of layers: 36.00 ± 2.65 min (2-layer), 46.00 ± 3.61 min (4-layer), and 62.00 ± 3.00 min (6-layer) (Fig. 2m, n). These results indicate that thicker coatings reduce total porosity, thereby limiting fluid transport, which is a critical consideration for nutrient perfusion in vivo. To evaluate scaffold biocompatibility, cell viability and proliferation were assessed. Live/dead staining displayed predominant green fluorescence across all groups (Fig. 2o), with quantitative analysis confirming high live cell ratios in the 2-layer (84.55 ± 2.64%), 4-layer (83.49 ± 1.07%), and 6-layer (86.45 ± 2.32%) scaffolds, with no statistically significant differences (Fig. 2p). Additionally, CCK-8 assays at 1, 3, and 5 days revealed comparable time-dependent cell proliferation among all groups (Fig. 2q), demonstrating that variations in layer counts and porosity did not impede cellular growth. Furthermore, endotoxin testing confirmed that the PCL-PGS minichannels were not contaminated (OD value: 0.12 ± 0.03), with no significant difference from the sterile water controls (Suppl. Fig. 3). These findings show that 3D minichannel scaffolds have dual functionality: one is controlled permeability for nutrient exchange, and the other is robust cytocompatibility for sustained cell viability.

### Preparation of cartilage granules and tracheal grafts

**Fig. 3:**
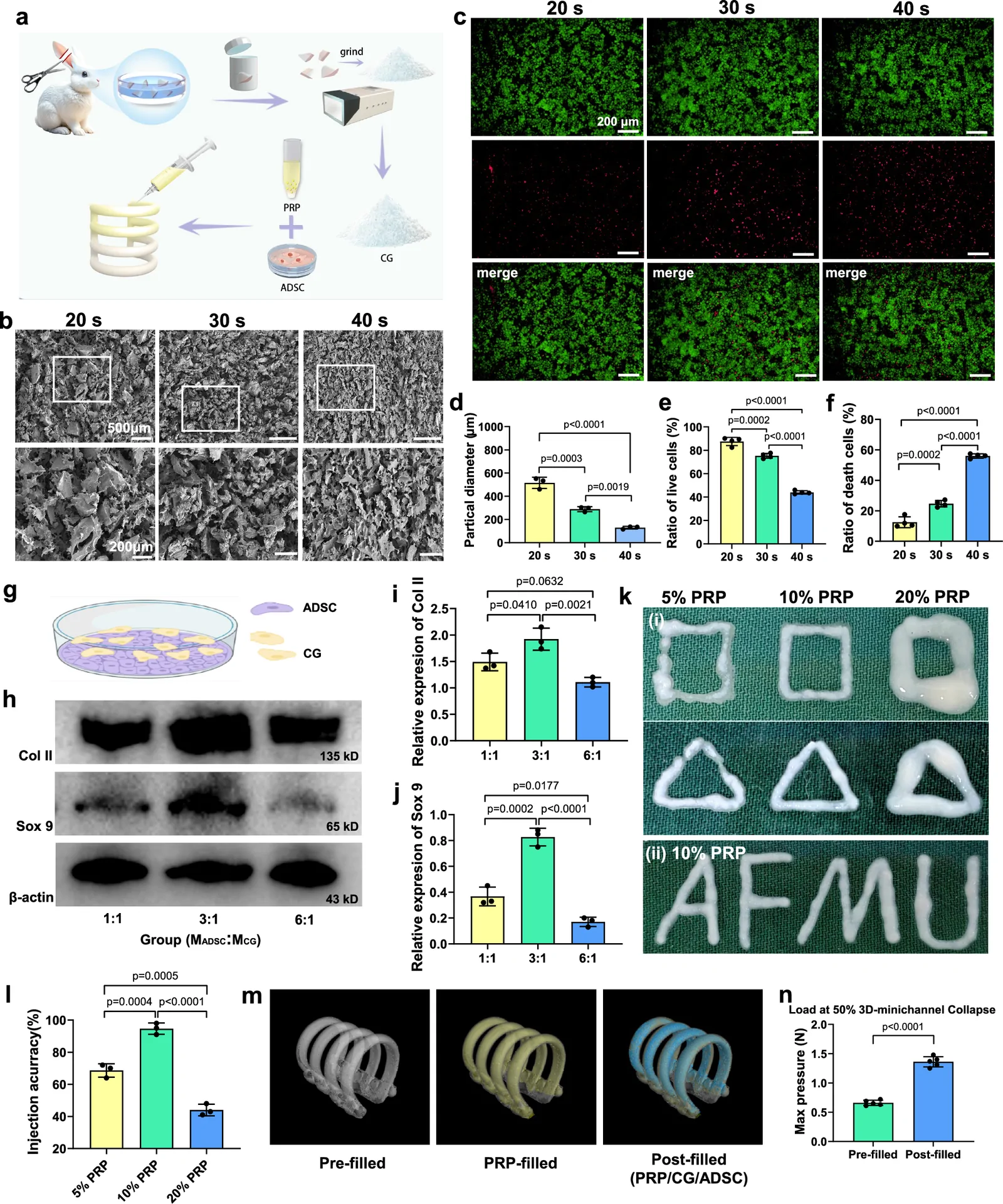
Preparation and characterization of injectable cartilage granules. **a** Schematic diagram of the preparation process of injectable cartilage granules. **b** SEM images of cartilage granules ground for different durations (20 s, 30 s, 40 s; n = 3 independent experiments with similar results; scale bars: 500 μm, 200 μm). **c** Live/dead cell staining of cartilage granules after different grinding times (*n* = 3 independent biological experiments with similar results; scale bars: 200 μm). **d** Comparison of particle sizes among the different groups (*n* = 3 independent biological samples). **e**, **f** Quantitative analysis of live/dead cells in cartilage granules (*n* = 4 independent biological samples). **g**, **h** Western blot analysis of collagen II (Col II) and Sox 9 in cocultures of cartilage granules (CG) and adipose-derived stem cells (ADSCs) (mass ratio of ADSCs to CG, M_ADSC_: M_CG_ = 1:1; 3:1; 6:1). **i**, **j** Relative expression of Col II (i) and Sox 9 (**j**) in different groups (*n* = 3 independent biological samples). **k** Images of injectable cartilage granule mixtures with varying platelet-rich plasma (PRP) ratios. **l** Comparative analysis of injection precision among the different groups (*n* = 3 independent samples). **m** 3D images showing cartilage granules filled with 3D minichannels were acquired via micro-CT scanning: (i) Hollow 3D minichannels, (ii) PRP-filled 3D minichannels, and (iii) PRP/CG/ADSC composite-filled 3D minichannels. **n** Comparative analysis of the maximum pressure before and after scaffold filling (*n* = 5 independent samples). Data in (**d**−**f**, **i**, **j**, **l**), and n are presented as mean values ± SD. For (**n**), significance was determined via a two-tailed unpaired Student’s *t*-test. For (**d**−**f**, **i**, **j**, **l**), statistical significance was determined via two-tailed one-way analysis of variance (ANOVA) followed by Tukey’s post-hoc test. Source data are provided as a Source Data file.

Figure 3a illustrates the preparation process of injectable cartilage granules. Approximately 4 cm² of cartilaginous tissue was harvested from the auricles of adult rabbits and processed via cryo-grinding technology to produce mini-granules (Suppl. Fig. 4). As the grinding time increased, the size of the cartilage granules progressively decreased (Fig. 3b), whereas the cell viability significantly decreased due to mechanical damage (Fig. 3c). Quantitative analysis revealed average granule diameters of 515.37 ± 48.33 μm (20 s), 288.52 ± 21.11 μm (30 s), and 129.16 ± 11.62 μm (40 s) (Fig. 3d). Cell viability assessments revealed a progressive decrease with increasing grinding duration: 87.53 ± 3.52% (20 s), 75.30 ± 2.12% (30 s), and 44.00 ± 1.44% (40 s) (Fig. 3e, f). To assess potential viability enhancement, cartilage fragments were treated with commercial cryopreservation solution prior to grinding. Live/dead analysis showed no significant difference in chondrocyte survival between cryo-grinding with and without cryoprotectants (78.66 ± 2.41% vs. 75.57 ± 2.22% at 30 s; Suppl. Fig. 5). Consequently, cryoprotectants were excluded from further research. On the basis of the balance between granule size and cell viability, 30 s was selected as the optimal grinding time for subsequent experiments.

Our previous study showed that stem cell-chondrocyte co-culture significantly enhances chondrocyte survival in vivo by attenuating apoptosis^21^. To leverage these protective effects, we isolated and cultured allogeneic adipose-derived stem cells (ADSCs), which are known to promote cartilage repair through matrix secretion (collagen II and aggrecan)^36,37^ and enhance angiogenesis via endothelial cross-talk^38,39^. The stemness and multilineage differentiation potential (adipogenic, osteogenic, and chondrogenic) of our isolated ADSCs were comprehensively verified (Suppl. Fig. 6), laying a solid foundation for their subsequent application. Tube formation assays further validated their angiogenic potential, demonstrating that the ADSC group increased the mean mesh size compared to the control group (Suppl. Fig. 7). To determine the optimal ratio of ADSCs to cartilage granules (CGs), in vitro coculture was performed for two weeks across three groups categorized by the wet mass ratio (M_ADSC_: M_CG_ = 1:1, 3:1, and 6:1). Western blot analysis demonstrated that the expression levels of cartilage-specific markers (Col II and Sox 9) were higher in the 3:1 group than in the 1:1 and 6:1 group (Fig. 3g–j). Subsequent in vivo ectopic implantation in nude mice further confirmed superior cartilage matrix deposition and structural integrity in the 3:1 group, as shown by Safranin-O and Alcian blue staining (Suppl. Fig. 8). Consequently, the 3:1 ratio was selected for subsequent experiments.

The incorporation of autologous PRP provided the necessary fluidity for injectable cartilage granule-stem cell aggregates, enabling syringe-based delivery. Upon injection, platelet activation initiates coagulation, stabilizing the C-shaped cartilage ring structure. Composites of PRP and cartilage granule-ADSCs with different weight ratios (5% PRP, 10% PRP, and 20% PRP) were utilized for injection to create various patterns (Fig. 3k, Suppl. Movie 3). The injection accuracy varied significantly among groups with different PRP concentrations. The 10% PRP group achieved the highest accuracy (94.67 ± 3.51%), which was significantly higher than that of the 5% PRP group (68.67 ± 4.16%) and the 20% PRP group (44.00 ± 3.61%). These findings indicate that 10% PRP provides optimal performance (Fig. 3l). Rheological analysis (Suppl. Fig. 9) revealed that the 5% PRP mixture presented high zero-shear viscosity (115,953.33 ± 20216.14 Pa·s) and a high yield strain (42.40 ± 8.83%), indicating particle aggregation and poor dispersion. This led to discontinuous flow under shear stress, causing extrusion jams. In the 20% PRP group, the significant reduction in viscosity and yield stress led to high fluidity, which resulted in a final injection accuracy of only 43.67 ± 2.08%. Conversely, the 10% PRP mixture demonstrated optimal rheological properties with moderate viscosity (36,543.67 ± 4411.75 Pa·s) and low yield strain (37.10 ± 8.84%), resulting in smooth injectability and high precision (95.67 ± 2.08%). The 10% PRP ratio optimized fluidity by balancing granule dispersion and the lubricating effects of PRP, which prevented aggregation while maintaining structural cohesion postinjection. Therefore, the 10% PRP ratio was selected for subsequent experiments.

The C-shaped hollow minichannel scaffold, combined with injectable cartilage granules, facilitated the construction of trachea-like transplants (Suppl. Movie 4). To clarify the filling effect of injecting cartilage granules in 3D minichannels, three-dimensional reconstruction of the constructs was performed. As demonstrated in the micro-CT 3D reconstruction images before and after injection (Fig. 3m), the PGS-PCL hollow minichannels, PRP, and cartilage granule–stem cell composites were pseudo-colored gray, yellow, and blue based on their relative densities. PRP uniformly perfused the internal lumens of the minichannels (Fig. 3m-ii), within which the blue-coded cartilage granules were also homogeneously distributed (Fig. 3m-iii). This internal reinforcement increased the compressive strength of the graft at 50% structural collapse from 0.66 ± 0.04 N to 1.36 ± 0.09 N (Fig. 3n).

### Optimization of vascularized 3D minichannel constructs

**Fig. 4:**
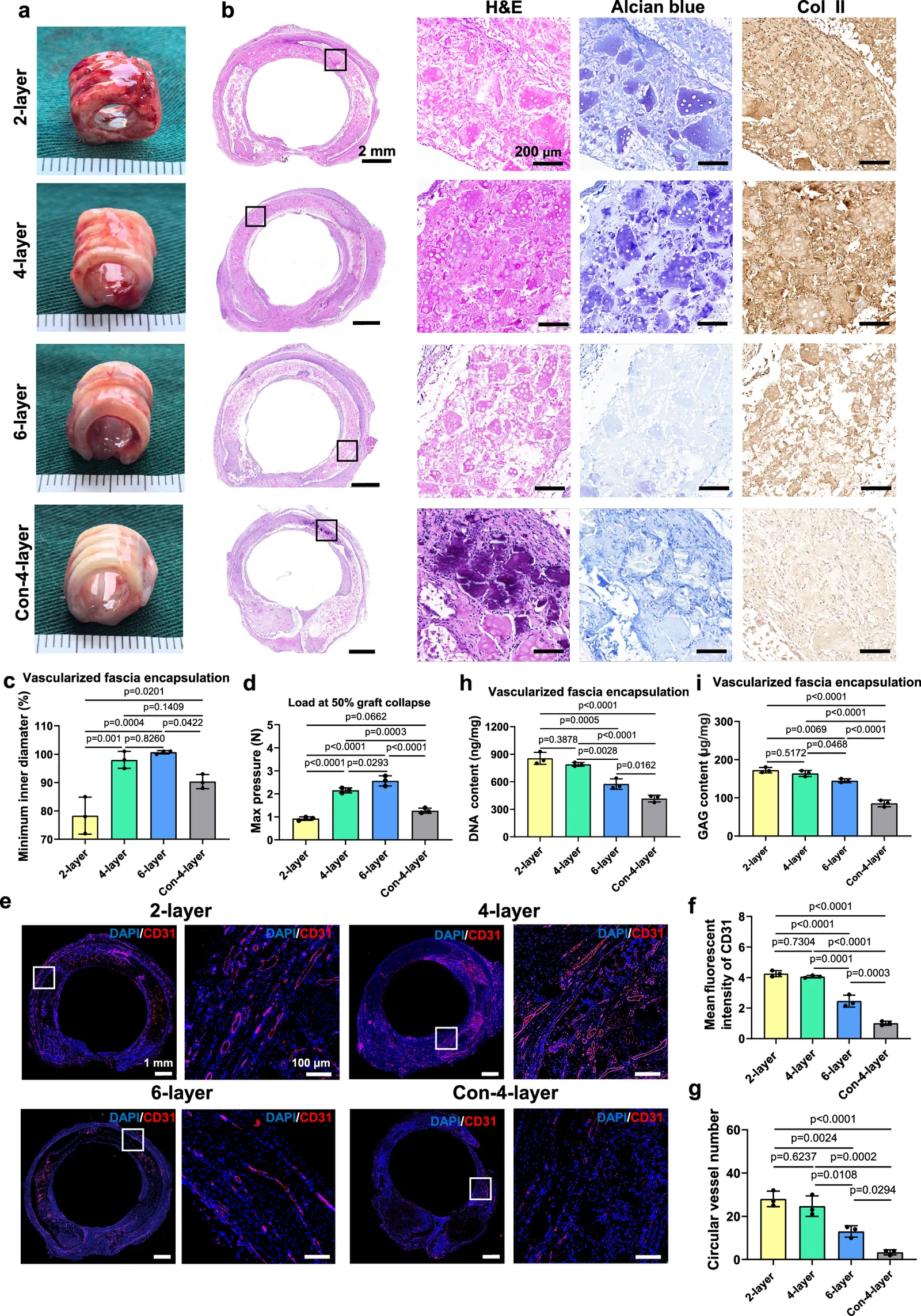
In vivo assessment of C-shaped minichannel tracheal scaffold vascularization. **a** Macroscopic image of each group of grafts after 2 weeks of implantation, highlighting the stability of the C-shaped grafts. **b** Histological sections of tracheal grafts from different groups were stained with H&E, Alcian blue and Col II (*n* = 3 independent experiments with similar results; scale bars: 2 mm and 200 μm). **c** Minimum inner diameter analysis of each group of grafts (*n* = 3 independent samples). **d** Compressive strength testing for different groups of constructs. The mechanical testing results revealed the maximum pressure required to achieve 50% collapse of the constructs (*n* = 3 independent samples). **e** Immunofluorescence staining for CD31-positive endothelial cells (*n* = 3 independent experiments with similar results; scale bars: 1 mm and 100 μm). **f**,** g** Quantitative analysis of CD31-positive cells (**f**) and blood vessels (**g**) (*n* = 3 independent biological samples). **h**, **i** Quantitative analysis of the DNA content (**h**) and glycosaminoglycan (GAG) (**i**) content in different group constructs (*n* = 3 independent biological samples). Data in c-d and f-i are presented as mean values ± SD. Statistical significance was determined via two-tailed one-way analysis of variance (ANOVA) followed by Tukey’s post-hoc test. Source data are provided as a Source Data file.

To evaluate vascularization capacity and cartilage integration in tracheal substitutes, we implanted C-shaped 3D minichannel constructs with various coating layers (2, 4, or 6 layers) into rabbit platysma for two weeks of in vivo remodeling. As demonstrated in Fig. 4a, the 4-layer constructs maintained structural fidelity with an intact C-shaped morphology and were fully encapsulated by vascularized fascia, whereas the 2-layer grafts exhibited lumen collapse (inner diameter was reduced compared with that of the other groups; Fig. 4c) and particle leakage (Fig. 4b). Mechanical testing revealed insufficient compressive strength in 2-layer grafts (0.92 ± 0.08 N for 2-layer groups, 2.15 ± 0.11 N for 4-layer groups, and 2.56 ± 0.22 N for 6-layer groups at 50% collapse; Fig. 4d), rendering them unsuitable for transplantation and operation. The 4-layer group achieved clinically relevant strength for surgical handling (Suppl. Movie 5), whereas the 6-layer groups presented restricted cellular infiltration due to dense coatings (Fig. 4b). In comparison, the 2-layer and 4-layer groups displayed extensive cell infiltration between the coating and cartilage granules, capillary formation penetrating the coating, and deeper alcian blue and type II collagen staining, indicating increased cartilage matrix content.

To explore the factors influencing the rapid vascularization of trachea-like grafts, a control group without ADSCs (con-4-layer) was established. The con-4-layer group presented a limited number of surface capillaries and histological signs of cartilage granule necrosis (Fig. 4b). CD31 immunofluorescence staining demonstrated superior endothelial recruitment and vessel penetration in both the 2-layer and 4-layer groups relative to the 6-layer and Con-4-layer groups (Fig. 4e). Quantitatively, the 2-layer and 4-layer groups exhibited significantly higher mean CD31 fluorescence intensity (4.26 ± 0.20 and 4.06 ± 0.08, respectively) than the 6-layer (2.45 ± 0.39) and Con-4-layer groups (1.00 ± 0.14; Fig. 4f). Correspondingly, the count of circular functional vessels per 20× field reached 28.00 ± 3.61 (2-layer) and 24.67 ± 4.73 (4-layer), markedly surpassing those in the 6-layer (13.00 ± 2.65) and Con-4-layer groups (3.33 ± 1.15) (Fig. 4g). These findings underscore the crucial role of ADSCs in promoting angiogenesis and ensuring the effective survival of cartilage granules in vivo. Notably, these vessels were predominantly localized within the surrounding fibrous tissue and the porous minichannels walls, with minimal infiltration into the dense cartilage granule aggregates themselves. This spatial pattern of vascularization supports the fascial integration while preserving the hypoxic core of the cartilage modules. Additionally, coating thickness could hinder the transmural growth of capillaries, thereby affecting the survival and integration of cartilage granules within the minichannels. The results of the porosity analysis of the scaffolds with different coating layers (Fig. 2g) confirmed that the reduction in blood vessel formation in the thicker coatings (Fig. 4e–g) was due to the decreased total porosity and extended nutrient diffusion distance, even though these pores are highly interconnected ( > 99%). Biochemical analyses revealed elevated glycosaminoglycan (GAG) (2-layer = 172.61 ± 7.10, 4-layer = 164.08 ± 7.88 μg/mg) and DNA contents (2-layer = 854.57 ± 65.76, 4-layer = 788.15 ± 20.75 ng/mg) in thinner coatings, indicative of chondrocyte survival and host cell recruitment (Fig. 4h, i). These data suggest that scaffolds with greater mechanical strength (thicker coatings or higher levels of graft vascularization) better maintain the lumen size, reducing the risk of collapse. Overall, the 4-layer design emerged as optimal, balancing mechanical resilience, vascular permeability, and matrix remodeling critical for preventing airway collapse while enabling host integration.

To assess the functional duration of the PCL-based scaffold in graft construction, scaffold-free CGPA constructs were implanted subcutaneously in rabbits for two weeks. Gross observation revealed that the injected cartilage granules completely turned into spheric cartilage tissues, with a smooth appearance, and extensive angiogenesis (Suppl. Fig. 10a). H&E staining revealed well-integrated cartilage granules with distinct chondrocyte lacunae and a deeply stained extracellular matrix (ECM) (Suppl. Fig. 10b). Mechanical testing revealed that the stress–strain curves of the scaffold-free CGPA grafts closely resembled those of the native tracheal cartilage (Suppl. Fig. 10c). Quantitative analysis revealed a maximum compressive force at a 50% strain of 2.99 ± 0.14 N for the scaffold-free CGPA grafts, which was greater than that of the native tracheal cartilage (Suppl. Fig. 10d). Compared with hollow PGS-PCL scaffolds, the cartilage granule-filled constructs exhibited significantly enhanced compressive strength after 2 weeks of prevascularization, confirming that the PCL-based scaffold functions primarily as a biomimetic template during early remodeling rather than as a permanent load-bearing structure (Fig. S11). These results suggest that the PCL minichannels serve primarily as a molding template during the initial two weeks, rather than providing long-term mechanical support, as the cartilage granules rapidly reconstitute into mechanically robust constructs.

### In situ repair of segmental tracheal defects in rabbits via 3D minichannel grafts

**Fig. 5:**
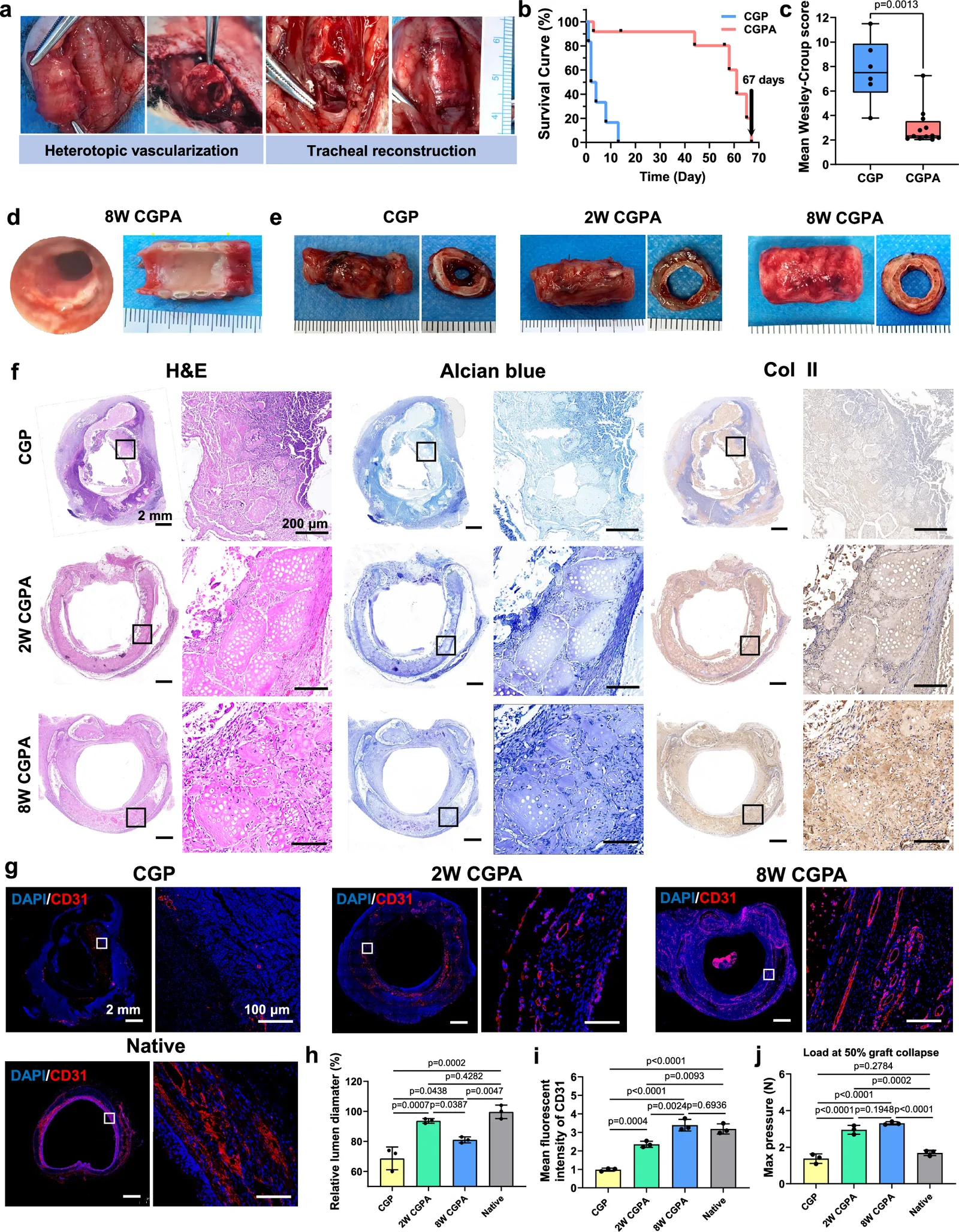
A 3D C-shaped graft was transplanted into a rabbit tracheal defect model. **a** Schematic illustration of the surgical procedure: (1) Heterotopic vascularization through prefabrication and (2) end-to-end anastomosis for tracheal reconstruction. **b** Survival curves of rabbits in the CGP and CGPA groups (the analysis excluded data from scheduled sampling points). **c** Quantitative comparison of Wesley-Croup scores between the CGP and CGPA groups (*n* = 6 animals for CGP, *n* = 12 animals for CGPA). **d** Bronchoscopic images (8 W CGPA) and **e** gross appearance of each group of tracheal grafts (2 W CGP, 2 W CGPA, and 8 W CGPA). **f** Representative histological images (H&E, Alcian blue, and Col II) of tracheal grafts from different groups (*n* = 3 animals per group with similar results; scale bars: 2 mm and 200 μm). **g** CD31 immunofluorescence staining revealed angiogenesis in the grafts (*n* = 3 animals per group with similar results; scale bars: 2 mm and 100 μm). **h** Inner diameter analysis of tracheal grafts from different groups (*n* = 3 independent samples). **i** Quantitative analysis of CD31 intensity in the different groups of grafts (n = 3 independent biological samples). **j** Compressive strength testing of different groups of tracheal grafts (50% collapse of the constructs, *n* = 3 independent samples). Data in c are presented as box plots showing the median (center line), 25th and 75th percentiles (bounds of box), and minimum to maximum values (whiskers) (CGP: median = 7.50, 25th–75th percentiles = 5.87–9.87, range (min-max) = 3.79–11.50; CGPA: median = 2.34, 25th-75th percentiles = 2.14–3.55, range (min-max) = 2.04–7.25), with individual data points overlaid as dot plots. Statistical significance for c was determined by a two-tailed Mann–Whitney *U* test. Data in (**h**–**j**) are presented as mean values ± SD. For (**h**–**j**), significance was determined via two-tailed one-way analysis of variance (ANOVA) followed by Tukey’s post-hoc test. Source data are provided as a Source Data file.

A total of 18 rabbits underwent successful construction of 2 cm segmental tracheal defects and received pedicled trachea-like 3D minichannel graft transplantation. The animals were divided into two groups: the CGP group (grafts composed of Cartilage Granules + PRP, without ADSCs; *n* = 6) and the CGPA group (grafts composed of Cartilage Granules + PRP + ADSCs; *n* = 12). The surgical procedure is illustrated in Fig. 5a. Following implantation, the grafts were immediately exposed to airflow and airway contamination. The well-integrated fascia ensured graft sealing, and no air leakage was detected after anastomosis, whereas the 3D minichannel-based cartilage rings effectively prevented graft collapse.

K‒M analysis demonstrated improved survival outcomes in CGPA-treated rabbits compared with those in CGP controls (Fig. 5b). The CGPA group exhibited significantly lower Wesley-Croup scores (median = 2.335, IQR: 2.135–3.548) compared with the CGP group (median = 7.500, IQR: 5.868–9.873), indicating improved respiratory outcomes in the CGPA group (Fig. 5c). Animal welfare was monitored daily using this system, and a score of ≥ 10 was set as the humane endpoint for euthanasia. In the CGP group, 50% of the rabbits did not survive beyond one week (median survival: 8 days; Supplementary Movie 6) because the unsupported grafts collapsed, causing severe respiratory distress that reached the humane endpoint threshold ( ≥ 10). In contrast, the CGPA group demonstrated superior outcomes, achieving survival rates of 91.67% (11/12) at 2 weeks and 77.78% (7/9, excluding scheduled two-week harvests) at 8 weeks, with the longest survival reaching 67 days. Within the CGPA group, one rabbit died from acute postoperative airway bleeding, while the remaining unscheduled deaths or euthanasias occurred when luminal mucus accumulation caused progressive respiratory distress without airway intervention, reaching the humane endpoint score. Longitudinal bronchoscopy surveillance and terminal gross analysis at 8 weeks confirmed sustained airway patency in the CGPA grafts, with no evidence of luminal collapse or stenosis at the graft-host interfaces (Fig. 5d, e; Suppl. Movie 7). Conversely, the C-shaped ring structure in the CGP group exhibited significant deformation. Quantitative analysis revealed significant differences in inner diameter between the groups: at 2 weeks, the native tracheal diameter of the CGP group was 68.67 ± 7.57%, whereas that of the CGPA group was 93.67 ± 1.53%. The CGPA constructs maintained functional lumen dimensions, which was attributed to vascularized fascial maturation conferring infection resistance (Fig. 5h).

Histological evaluation revealed distinct differences in graft remodeling. In the CGPA group, H&E, Alcian blue, and type II collagen immunohistochemistry revealed tightly connected cartilage granules with typical chondrocyte lacunae and ECM deposition within minichannels at both 2 and 8 weeks (Fig. 5f). CD31 immunofluorescence staining revealed extensive transmural neovascularization (Fig. 5g), and the relative CD31 intensities at both timepoints in the CGPA group were significantly greater (2 W: 2.36 ± 0.17; 8 W: 3.39 ± 0.31) than those in the CGP group (0.98 ± 0.08) (Fig. 5i). Notably, the vascularization level in the 8-week CGPA grafts showed no significant difference from the native trachea (3.18 ± 0.27), indicating successful host-driven neovascularization. Moreover, the CGP group exhibited channel collapse, cartilage necrosis, and inflammatory cell aggregation due to graft infection. The compressive strength values at 2 weeks were 2.95 ± 0.24 N (CGPA), 1.69 ± 0.15 N (native) and 1.37 ± 0.26 N (CGP) (Fig. 5j). Epithelial regeneration within the graft lumen was evaluated over time (Suppl. Fig. 12). At 2 weeks, cytokeratin-positive (CK) cells were sparsely distributed. By 8 weeks, CK immunoreactivity had increased significantly, forming a more contiguous epithelial-like layer with fluorescence intensity comparable to that of native trachea (19.20 ± 1.91 vs. 18.42 ± 2.14). In contrast, acetylated tubulin (Ac-Tub) expression was detected but was sparse and of low intensity (7.75 ± 1.13 vs. native 13.79 ± 0.77), suggesting that the regenerated epithelium at this stage was predominantly of a squamous phenotype, not yet fully differentiated into a mature, ciliated respiratory epithelium. This pattern of squamous epithelial regeneration, supported by the underlying vascularized fascia, appeared sufficient to provide a protective barrier. The controlled local inflammatory response corroborated this functional protection, as levels of the pro‑inflammatory cytokines IL‑1β (3.59 ± 0.55) and TNF‑α (2.43 ± 1.00) at 8 weeks were not significantly elevated compared to native trachea (2.14 ± 0.96 and 1.48 ± 0.57, respectively) (Suppl. Fig. 13a–c). These data indicate that rapid graft vascularization facilitates the establishment of a protective squamous epithelial lining, which contributes to graft integration and resistance to infection during the critical remodeling phase.

**Fig. 6:**
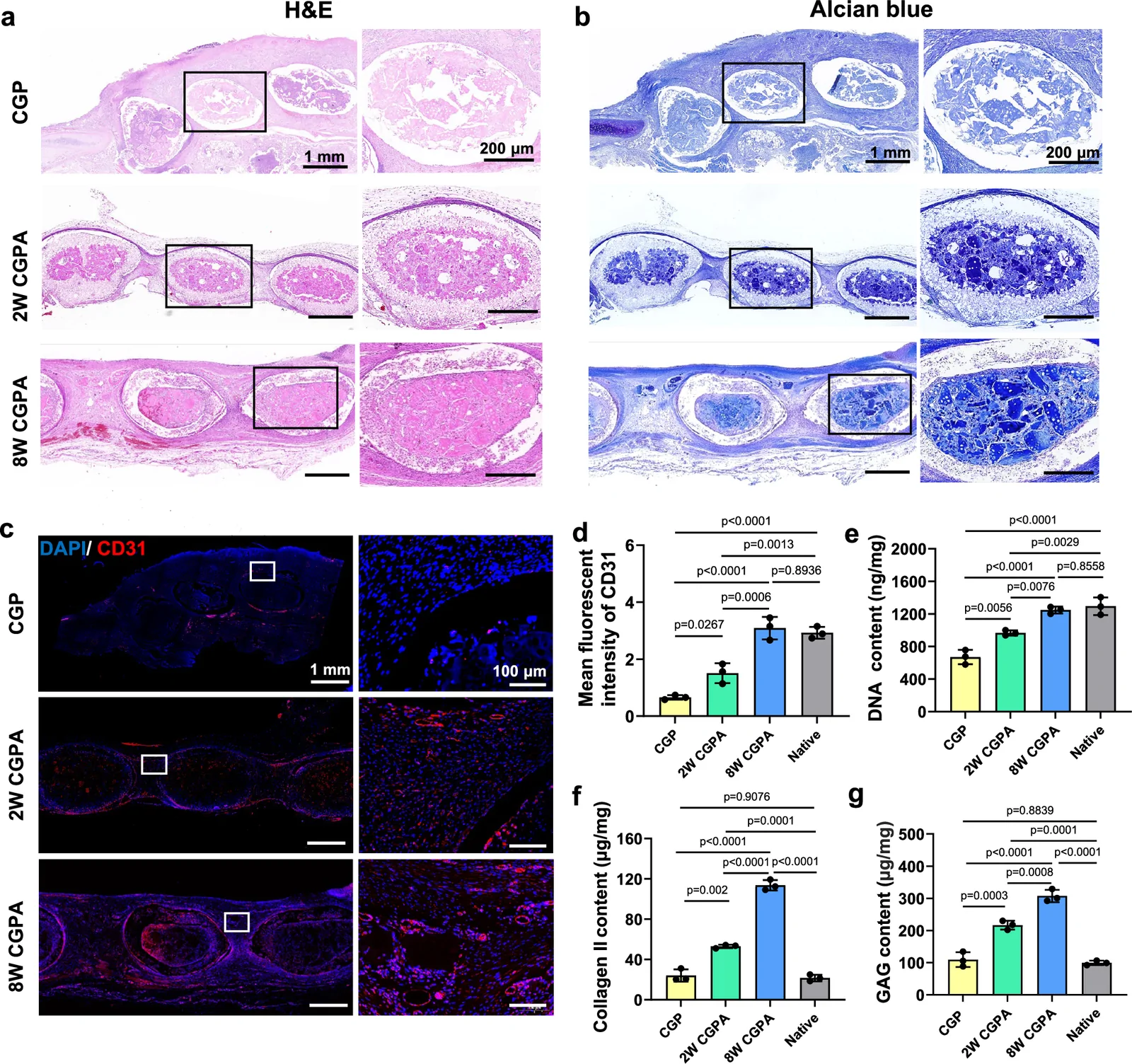
Longitudinal histological analysis of tracheal grafts in rabbits. **a**, **b** Representative histological sections stained with H&E (**a**) and Alcian blue (**b**) (*n* = 3 animals per group with similar results; scale bars: 1 mm and 200 μm). **c** CD31 immunofluorescence staining for in vivo vascularization (*n* = 3 animals per group with similar results; scale bars: 1 mm and 100 μm). **d** Quantitative analysis of CD31 intensity in the different groups of grafts (*n* = 3 independent biological samples). **e**–**g** Quantitative analysis of the DNA content (**e**), Col II content (**f**), and GAG content (**g**) in each group of tracheal grafts (*n* = 3 independent biological samples). Data in (**d**–**g**) are presented as mean values ± SD. Statistical significance was determined via two-tailed one-way analysis of variance (ANOVA) followed by Tukey’s post-hoc test. Source data are provided as a Source Data file.

Longitudinal section analysis (Fig. 6a, b) revealed uneven luminal surfaces with collapse toward the lumen in the CGP group, along with exudate accumulation contributing to asphyxiation. The CGPA group presented smooth luminal surfaces, organized tissue fascia, and enhanced cellular infiltration within minichannels. CD31 immunofluorescence staining confirmed extensive transmural neovascularization (Fig. 6c), with quantitative analysis showing comparable vascular density between the 8-week CGPA grafts and native trachea (Fig. 6d). DNA content analysis revealed no significant difference between CGPA grafts and the native trachea at 8 weeks (Fig. 6e), indicating adequate host cell integration. Biochemical analysis revealed significantly greater collagen II and GAG contents in the CGPA group than in both the control and native trachea at all time points (Fig. 6f, g). To characterize epithelial regeneration, immunofluorescence staining for CK and Ac-Tub along the graft axis at 2 and 8 weeks was performed (Suppl. Fig. 14). At 2 weeks, CK-positive squamous epithelial cells were confined to the anastomotic regions (6.47 ± 1.25), with only sporadic positive cells at quarter points (2.21 ± 0.81) and rare scattered cells in the midportion (1.19 ± 0.41). Ac-Tub expression was negligible beyond the anastomosis, with only occasional positive cells at the anastomotic margins (2.89 ± 1.09). By 8 weeks, CK-positive coverage had extended discontinuously from the anastomotic regions (18.87 ± 2.33) to the quarter points (12.99 ± 1.55), both significantly increased versus 2 weeks. However, the midportion showed only sparse CK-positive cells (2.10 ± 0.70), which was not significantly different from 2 weeks. Notably, Ac-Tub-positive ciliated cells appeared more prominently at anastomotic sites (11.21 ± 1.64, vs. 2 weeks) and extended in a scattered pattern to the quarter points, while the midportion remained negative (1.58 ± 0.45, vs. 2 weeks). These findings confirm a centripetal “creeping regeneration” pattern in which host-derived epithelium progressively migrates from the defect margins toward the graft center over time. However, complete squamous epithelial coverage of the graft lumen was not achieved within the 8-week observation period. To assess the potential hazards associated with 3D minichannel grafts, an in vivo analysis of biocompatibility was conducted (Suppl. Fig. 15). No discernible anomalies, such as textural variations or tissue ulceration, were observed in the other organs.

### Addition of ADSCs upregulates glycolysis in chondrocytes enhances the survival of cartilage granules

**Fig. 7:**
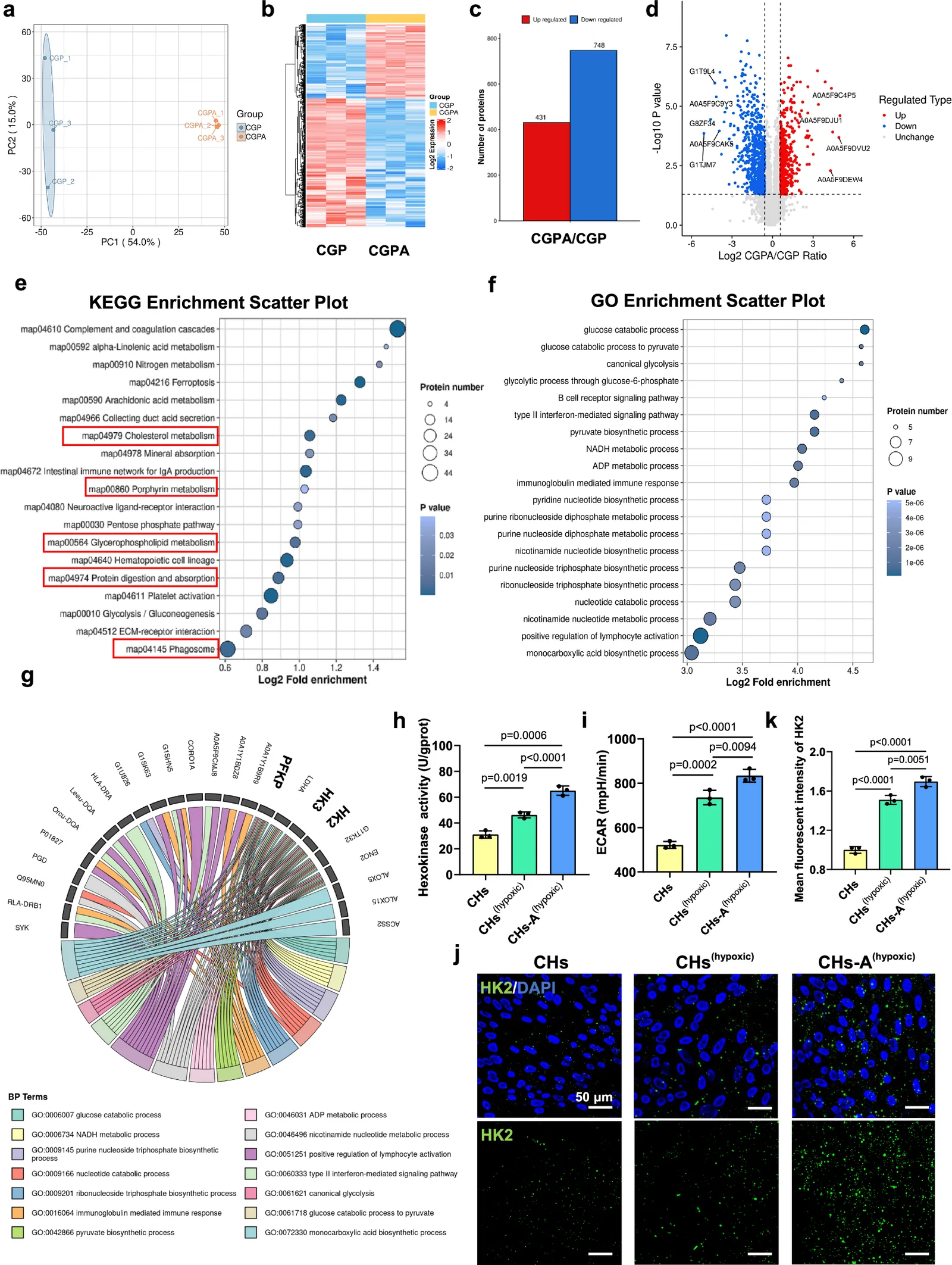
Proteomic analysis of injectable cartilage granules after subcutaneous implantation. **a** Principal component analysis (PCA) of differentially expressed proteins between CGP and CGPA groups (*n* = 3 independent biological samples). **b** Heatmap of hierarchical clustering for differentially expressed proteins. **c**, **d** Volcano plot (**c**) and quantitative histogram (**d**) of upregulated (red) and downregulated (blue) proteins. **e** KEGG pathway enrichment scatter plot showing metabolic pathway involvement**. f** GO enrichment analysis of metabolism-related biological functions showing upregulation of glycolytic processes. Bubble size represents protein count and color intensity indicates significance level**. g** GO Chord diagram displaying key glycolytic enzymes enriched in the CGPA group. **h** Hexokinase activity assay results demonstrating the enzymatic activity of hexokinase in chondrocytes under different conditions (*n* = 3 independent samples). **i** Extracellular acidification rate (ECAR) measurements showing the glycolytic activity of chondrocytes under normoxic and hypoxic conditions (*n* = 3 independent biological samples). **j** Immunofluorescence staining of hexokinase 2 (HK2) in chondrocytes cultured under different conditions (*n* = 3 independent experiments with similar results; scale bars: 50 μm). **k** Relative fluorescence intensity of HK2 normalized to that of the normoxic control group (*n* = 3 independent biological samples). Statistical significance for differential protein expression (**d**) was determined using two-tailed Student’s *t*-test, whereas KEGG (**e**) and GO enrichment (**f**) analyses were evaluated using two-tailed Fisher’s exact test. *p*-values were adjusted for multiple comparisons using the Benjamini-Hochberg method. Data in h-i and k are presented as mean values ± SD. Statistical significance was determined via two-tailed one-way analysis of variance (ANOVA) followed by Tukey’s post-hoc test. Source data are provided as a Source Data file.

Cartilage granule-based 3D minichannel grafts (CGPs vs CGPAs) were implanted in vivo for 2 weeks (*n* = 3 independent samples) for 4-DIA-based quantitative proteomics analysis. A total of 40,140 proteins were identified, of which 5560 contained quantitative information. Principal component analysis (PCA) revealed the distribution of differentially expressed proteins between the two groups of cartilage granules (CGP vs. CGPA) (Fig. 7a). A heatmap revealed significant differences in proteomic expression between the CGP group and the CGPA group at the remodeling stage in vivo (Fig. 7b). A volcano plot revealed 1179 differentially expressed proteins, with 431 upregulated (red dots) and 748 downregulated (blue dots) (Fig. 7c, d). The upregulated genes included energy metabolism-related proteins such as fatty acid binding protein 7 (FABP7), hexokinase 2 (HK2), hexokinase 3 (HK3), and ATP-dependent 6-phosphofructokinase (PFKP). The pathways enriched by KEGG analysis were highly correlated with carbohydrate metabolism, lipid metabolism, and amino acid metabolism, such as glycolysis, the pentose phosphate pathway, cholesterol metabolism, and glycerophospholipid metabolism (Fig. 7e). Moreover, GO enrichment analysis was conducted on the differentially expressed proteins related to energy metabolism pathways, and the addition of ADSCs led to the upregulation of biological functions associated with glycolysis (Fig. 7f). To identify the key upstream differentially expressed proteins related to these biological processes, we used GO Chord plots. The data indicated that six genes were related to glycolytic processes, such as enolase 2 (ENO2), HK2, HK3, L-lactate dehydrogenase (LDHA), and PFKP (Fig. 7g). The addition of ADSCs to the cartilage granules significantly increased the glycolytic metabolism level of the cartilage. This enhancement enables the cartilage granules to resist hypoxic environments in the early stages of poor vascularization^40^, effectively increasing the survival rate of the cartilage granules.

To validate these findings, we assessed the metabolic activity of chondrocytes by measuring hexokinase activity and cellular respiration rates under different culture conditions. The results demonstrated that, under hypoxic conditions, the CHs_hypoxic_ presented significantly greater hexokinase activity than did the CHs (CHs: 31.12 ± 2.80 U/g prot, CHs_hypoxic_: 46.22 ± 2.35 U/g prot), as well as an increased extracellular acidification rate (CHs: 521.86 ± 15.63 mpH/min; CHs_hypoxi_c: 735.68 ± 32.41 mpH/min) (Fig. 7h, i). When ADSCs were introduced via transwell coculture with chondrocytes, the hexokinase activity in the CH-A_hypoxic_ group further increased to 65.02 ± 3.61 U/gprot, and the extracellular acidification rate increased to 833.91 ± 28.59 mpH/min. Immunofluorescence staining of HK2 further confirmed the differential expression of this key glycolytic enzyme across the experimental groups in vitro (Fig. 7j, k). These data indicate that the introduction of ADSCs significantly increases glycolytic activity in chondrocytes.

### Transplantation of cartilage granule-based 3D minichannel grafts into Pigs

**Fig. 8:**
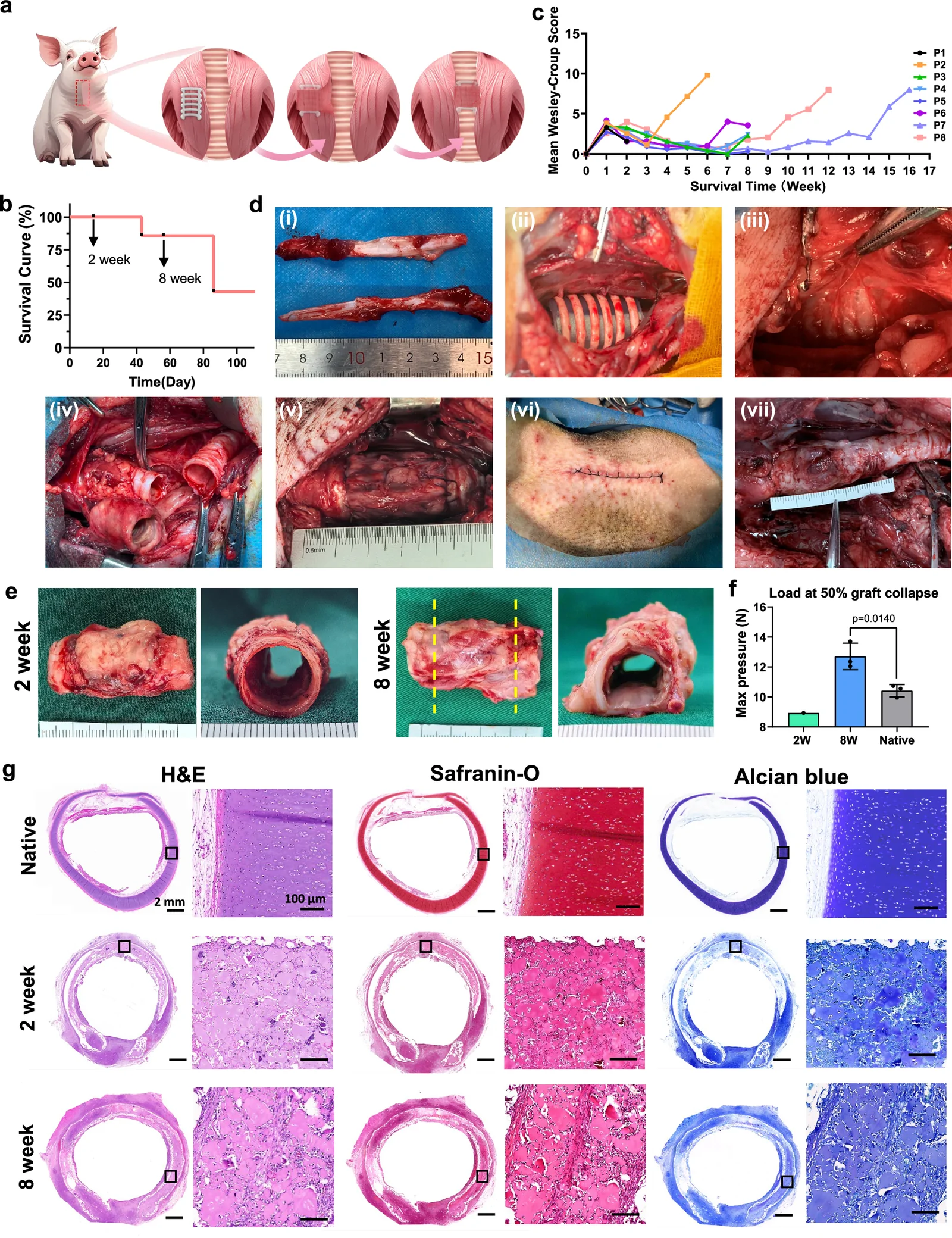
Surgical and histological analysis of tracheal grafts in pigs. **a** Schematic illustration of segmental tracheal defect repair in pigs. **b** Survival curve depicting the survival rate of pigs following CGPA graft implantation (*n* = 8 animals; censored animals were euthanized for scheduled sampling). **c** Daily Wesley-Croup scores following segmental tracheal reconstruction. **d** Overview of the surgical procedure: autologous rib cartilage harvesting (i), ectopic prevascularization of the 3D graft (ii, iii), creation of a 3-cm segmental tracheal defect (iv), end-to-end graft anastomosis (v), wound closure (vi), and the fully reconstructed trachea in situ (vii). **e** Gross images of tracheal grafts at 2 weeks and 8 weeks post-implantation. **f** Compressive strength testing of the cartilage granule-based 3D minichannel grafts and native tracheal grafts (50% collapse of the constructs, *n* = 3 independent samples in the 8 W and Native groups). **g** Histological evaluation of cartilage granule-based 3D minichannel grafts (scale bars: 2 mm and 100 μm). For (**f**), data for 8 W and Native groups are presented as mean values ± SD; data for 2 W represents a descriptive sample (*n* = 1 animal). Statistical significance between 8 W and Native groups was determined via a two-tailed unpaired Student’s t-test. Source data are provided as a Source Data file.

To assess the clinical feasibility of the 3D minichannel tracheal scaffold, we conducted tracheal repair surgeries in a porcine model. Given the limitations of auricular cartilage for clinical applications, rib cartilage was selected as the source material for cartilage granule preparation. The viability of rib cartilage cells post grinding was systematically analyzed to ensure granule quality and functionality for scaffold construction. SEM analysis revealed that increasing the grinding time resulted in a more uniform granule morphology with a reduced particle size. Quantitative particle size analysis revealed a progressive decrease: 542.67 ± 42.19 μm (20 s), 390.67 ± 30.02 μm (30 s), and 205.00 ± 42.72 μm (40 s) (Suppl. Fig. 16a, b). Cell viability assessment through staining confirmed decreased viability with extended grinding: 64.67 ± 2.52% (20 s), 59.33 ± 3.06% (30 s), and 35.00% ± 4.58% (40 s) (Suppl. Fig. 16c, d). On the basis of the balance between cell viability and injectability, 30 s was determined to be the optimal grinding duration for rib cartilage. After 2-week prevascularization, 3D minichannel scaffolds loaded with autologous cartilage, PRP, and allogeneic ADSCs were used for end-to-end anastomosis to repair 3 cm tracheal defects in Bama minipigs. The surgical procedure for repairing tracheal defects in pigs is illustrated in Fig. 8a, d.

Kaplan–Meier analysis indicated an 88.9% survival rate (6/7) at 8 weeks post-implantation, with one animal electively euthanized at the 2-week endpoint for histological assessment (1/8) (Fig. 8b). Animal welfare was monitored daily using the Wesley-Croup scoring system, and a score of ≥ 10 was set as the humane endpoint for euthanasia (Fig. 8c). Four animals showing only mild respiratory signs were euthanized as scheduled at 8 weeks. The unscheduled euthanasias occurred at postoperative days 43 (score 12), 86, and 115 when luminal mucus accumulation caused progressive respiratory distress without airway intervention, reaching the humane endpoint score. Gross examination of explanted grafts confirmed that all grafts maintained structural patency above 70% without collapse or tissue necrosis (Suppl. Fig. 17), demonstrating that these euthanasias were caused by temporary mucus-induced dyspnea prior to complete ciliated re-epithelialization rather than graft structural failure.

Gross examination at 2 weeks revealed complete graft encapsulation by vascularized fibrous tissue, with smooth luminal surfaces and no evidence of material exposure or infection (Fig. 8e). The well-vascularized and remodeled cartilaginous granules formed a C-shaped ring structure, enabling the graft to achieve a mechanical strength comparable to that of the native trachea (8 week vs. Native, Fig. 8f). Histological analysis (Fig. 8g) revealed patterns of rib cartilage source remodeling. The rib cartilage exhibited a dense extracellular matrix with sparse chondrocytes, resulting in tightly packed cartilage microparticles devoid of discernible lacunar structures within remodeled rings. Notably, the cartilage granules achieved complete fusion, maintaining minichannel architecture without collapse or deformation, thereby recapitulating the biomechanical and structural properties of native tracheal tissue. Histological analysis of porcine tracheal grafts indicated progressive epithelial regeneration during the observation period (Suppl. Fig. 12d, 14d–f). At 2 weeks post-implantation, only discontinuous CK-positive cells were present. By 8 weeks, continuous CK-positive epithelial coverage was established, with a fluorescence intensity of 21.41 ± 1.83, nearing that of native trachea (25.37 ± 2.31). Evidence of ciliated epithelial regeneration also appeared at this time point, as shown by increased Ac‑Tub fluorescence intensity (8.58 ± 1.18 at 8 weeks vs. 2.43 ± 1.23 at 2 weeks), though still below the native level (17.07 ± 0.83). This progressive epithelialization, particularly the emergence of ciliated structures, highlights the enhanced regenerative outcome in the large animal model, which may be attributed to improved vascularization and epithelial cell migration supported by the cartilage granule‑based scaffold.

These findings underscore the translational potential of cartilage granule-based grafts for segmental tracheal reconstruction, particularly in scenarios where autologous chondrocyte availability is limited. The successful integration and biomechanical stability of the cartilage granule-based 3D minichannel grafts in a large animal model suggested translational potential, but significant challenges remained, particularly regarding epithelial regeneration and the prevention of late-phase complications.

## Discussion

Despite reported clinical successes in cartilage regeneration^23,41,42^, replacing segmental tracheal defects remains a major surgical bottleneck, principally because functional graft integration requires systematically overcoming four hurdles: cell and scaffold preparation, mechanical stenting, graft vascularization, and luminal epithelialization. Traditional tissue-engineered constructs, ranging from decellularized allografts to 3D-printed polymeric frameworks, commonly fail because they cannot satisfy these criteria at the same time^7,15,43^. Decellularized matrices suffer from low cell retention and chondrogenic failure, leading to structural collapse, whereas standard 3D-printed scaffolds often delay host vascularization and invite infection due to limited cell-seeding efficiency and transmural porosity^44–46^. In this study, our strategy effectively addresses the first three hurdles. By embedding autologous cryo-ground cartilage granules, PRP, and ADSCs within 3D-printed PCL-PGS minichannels, we eliminate the need for lengthy in vitro cell expansion, deliver immediate mechanical stenting against breathing-induced circumferential compression, and achieve rapid transmural vascularization within two weeks. The fourth hurdle, establishing a mature, ciliated epithelium across the graft center, remains only partially overcome within our 8-week observation window, highlighting where future regenerative interventions must focus.

Our approach represents a paradigm shift in tracheal cartilage regeneration. Instead of relying on traditional scaffold-based strategies to attach cell, we developed a 3D-printed minichannel to shape submillimeter-scale cartilage granules into anatomically precise C-shaped rings. The choice of cartilage source was determined by the combination of surgical practicality and preclinical modeling considerations. Even though there are phenotypic differences between elastic and hyaline cartilage, the main functional requirement for the graft (mechanical airway stabilization) was successfully met. The platform is agnostic to cartilage type, as shown by the effective use of hyaline (costal) cartilage in the porcine model, which highlights its translational adaptability to clinically available autologous sources. By avoiding enzymatic digestion, we kept the native cartilage ECM, enabling it to be directly reconstituted into functional cartilage rings. This study shows, for the first time, this approach is feasible, and after two weeks, the host is being remodeled nearly completely, and the graft is being integrated robustly.

As the cartilage carrier, the 3D minichannel scaffold embodies three essential design features: porous channel walls, tunable mechanical properties, and a biomimetic C-shaped configuration. The porous walls are particularly critical, as salt-leaching-derived micropores and rapid PGS degradation create pathways for nutrient perfusion and capillary ingrowth while minimizing foreign material exposure and the risk of fibrous encapsulation^33,39,47–49^. However, porosity–vascularization trade-offs emerged: although pore connectivity exceeded 99% across all constructs (Fig. 2h), thicker coatings (6-layer) reduced total porosity and increased diffusion distance, thereby compromising transmural angiogenesis. Mechanically, the scaffold provides sufficient rigidity to maintain lumen patency during early remodeling, while PCL’s slow degradation ( > 12 months) and biocompatibility ensure sustained support without eliciting significant inflammation, as evidenced by negligible IL-1β/TNF-α elevation around PCL materials (Suppl. Fig. 13). Importantly, the scaffold-free CGPA grafts achieved mechanical properties exceeding those of native tracheal cartilage within 2 weeks (Suppl. Fig. 10), confirming that the PCL-based construct serves primarily as a molding template during the initial remodeling phase rather than as a permanent load-bearing structure. The caramel templating technique further enabled post-printing morphing into patient-specific C-shaped geometries, a capability rarely explored since the first application of caramel in cardiac patch fabrication^34^. These findings validate the 3D minichannel as a transient scaffold that promotes rapid host integration for tracheal cartilage reconstitution.

Although tissue engineering strategies utilizing cell seeding scaffolds have shown promise^8,11,48,49^, the application of minced cartilage has gained significant acceptance in cartilage repair over the past decade. Autologous cartilage fragments have successfully regenerated osteochondral defects^17,18^ in preclinical models, with established clinical protocols for knee lesions^16^. Recent advances have further demonstrated the integration of minced cartilage and hydrogels within 3D-printed scaffolds for craniofacial reconstruction^19^. For cartilage regeneration, we developed the cryo-grind cartilage granules technique, which bypasses the need for chondrocyte isolation via enzymatic digestion and addresses the critical shortage of autologous chondrocyte sources. While this method achieves a 60–70% percentage of viable granules, which is influenced by the grinding duration, it successfully regenerated functional cartilage rings from both auricular and costal cartilage granules, restoring circumferential tracheal defects in rabbits and pigs with robust mechanical strength within weeks. Notably, in porcine models, larger minichannel diameters enable shorter grinding durations, suggesting potential improvements in granule viability for human applications. This approach has two advantages. First, it removes the prolonged in vitro culture: cartilage can be excised and immediately processed into granules, then transplanted for prevascularization and remodeling, which significantly reduces contamination risks from extended culture periods^2^. Second, the cartilage-specific ECM within granules provides inherent bioactivity that is absent in synthetic materials^43,50^. This ECM includes chondroinductive factors vital for chondrocyte viability and proliferation, offering a microenvironment better than conventional cell-scaffold systems. In the engineered grafts, the Alcian blue and collagen II staing patterns are different from those of native tracheal cartilage. This in line with the biology of adult-derived cartilage, which shows that chondrocytes have limited synthetic activity. In our model, success isn’t just the generation of a lot of new cartilage, but it’s the preservation of the implanted cartilage matrix and its integration into a cohesive, load-bearing structure. The matrix of individual granules shows deep staing, and there is biochemical evidence of sustained GAG and collagen II content (Fig. 6f, g), which suggests effective matrix maintenance and granule viability. This preserved micro-architecture directly links to the graft’s mechanical strength, which is comparable to native trachea, and its ability to maintain airway patency, meeting the essential functional requirement for tracheal reconstruction.

A key consideration in tracheal graft design is balancing the need for rapid vascularization with the requirement for a hypoxic chondrogenic niche^51,52^. In our approach, the induced angiogenesis primarily serves a structural and protective function: it ensures the viability and robust integration of the vascularized fascia that envelops the graft. This fascial layer is critical for creating an airtight seal at the anastomosis, resisting infection, and providing mechanical support against collapse. Achieving this spatially regulated vascularization is essential for the long-term viability of engineered cartilage constructs, ensuring that the tissue does not undergo necrotic collapse^52^. Conversely, the cartilage granules confined within the mini channels remain in a relatively avascular and hypoxic microenvironment that is conducive to chondrocyte metabolism^53^. Thus, the newly formed blood vessels were primarily distributed in the fascial stroma and around the minichannels, while the cartilage core was in an avascular state conducive to matrix maintenance, thereby resolving the balance between graft vascularization and cartilage survival. In our study, the significantly elevated GAG content in CGPA grafts (Figs. 4i, 6g) reflects proteoglycan deposition, a hallmark of cartilage regeneration, which is crucial for the mechanical strength of cartilage. Critically, this ECM maturation occurred without inducing microenvironment acidosis, a known risk in high-GAG environments^54,55^. Histological assessment confirmed the presence of viable chondrocytes with intact lacunae and the absence of cartilage necrosis, whereas the bicarbonate buffer capacity of PRP^56^ maintained a balanced pH during early remodeling. This balance was further ensured by ADSC-driven vascularization, which facilitated efficient clearance of metabolic byproducts^57^.

In line with current clinical understanding, the regeneration of a functional ciliated epithelium remains a formidable challenge in tracheal tissue engineering. Current approaches for functional airway epithelial reconstruction include pre-seeding epithelial cells, iPSC-derived mesenchymal stem cells/chondrocytes (outer layer) and human bronchial epithelial cells (hBECs) (inner layer) onto tracheal grafts or fabricating 3D-bioprinted tracheal constructs with epithelial cells^48,58–60^. However, these strategies are clinically limited owing to the long culture period (2–6 weeks), high contamination risk, and restricted cell seeding efficiency and cell survival rate^48^. Our strategy prioritizes the establishment of a vascularized, mechanically stable graft that can host and protect epithelial progenitor cells during the remodeling phase. Between 2010 and 2020, most researchers stented tissue-engineered tracheal grafts to ensure early luminal patency. However, stent placement may physically hinder epithelial migration and provoke local inflammation, creating a well‑recognized dilemma: adequate patency but compromised re‑epithelialization^3,11,48^. In the present study, we attempted to avoid stenting. Nevertheless, some animals developed sputum accumulation and fatal complications, suggesting that without temporary mechanical support, maintaining the patency of free tracheal grafts may be challenging before complete re‑epithelialization. Without a stent, there is a risk of sputum stasis and obstruction; with a stent, epithelial quality may suffer, increasing infection, granulation, and restenosis risks^8,14^. It is speculated that tracheostomy or bronchoscopic intervention may be resorted to for relief of the above complications in human cases. However, their feasibility and efficacy would require dedicated evaluation in future large‑animal studies with longer follow‑up. Addressing the above dilemma may not require a binary choice. First, degradable or removable stents could provide early support and then disappear as epithelialization progresses^61^. Second, bioactive coatings or growth factor delivery might also accelerate epithelial migration and reduce stent dependence^62^. For instance, our previous work demonstrated that the decellularized extracellular matrix (dECM) enriched with stem-cell-derived vesicles can actively recruit epithelial progenitors^63^. By optimizing the culturing strategy, such bioactive matrices could be reinforced to provide sufficient mechanical strength while remaining highly bioactive. Admittedly, these strategies need systematic validation in large animal models. We believe that the observed complications did not negate the value of a stent-free approach. Rather, they reminded us that before slow epithelialization was adequately addressed, relying solely on stent-free grafts for long segment reconstruction may still pose preclinical safety concerns. Future studies may benefit from synergistic optimization of stent design and epithelialization-promoting strategies.

In addition to metabolic modulation, ADSCs exert potent antiapoptotic effects through paracrine signaling. Consistent with previous studies demonstrating that mesenchymal stem cells secrete trophic factors such as VEGF, IGF-1, and HGF, which inhibit caspase-3 activation and upregulate antiapoptotic proteins such as Bcl-2^21,60,64–66^, our live/dead staining revealed significantly reduced chondrocyte apoptosis in the ADSC coculture groups compared with the control groups. This antiapoptotic mechanism works with improved glycolysis to keep chondrocyte survival for a long time during the critical prevascularization phase. A key finding of this study is the dual role of ADSCs in promoting cartilage granule survival and angiogenesis. In our previous work, where starvation-preconditioned bone marrow mesenchymal stem cells (BMSCs) enhanced the survival of co-transplanted chondrocytes^21^, we demonstrated that allogeneic ADSCs not only increased cartilage granule viability within minichannels but also significantly enhanced angiogenesis. These findings match prior reports on ADSCs in cartilaginous grafts^36,66^. Proteomic profiling demonstrated that ischemia-primed ADSCs upregulate glycolytic pathways within cartilage granules, inducing a metabolically active state. This reprogramming generates key glycolytic intermediates, notably glyceraldehyde‑3‑phosphate and phosphoenolpyruvate, and these intermediates serve as biosynthetic substrates for amino acid, lipid, and nucleotide production^38,66^. The consistency between these metabolic findings and our histological observations of enhanced angiogenesis in the CGPA group further strengthens the mechanistic link between ADSC-mediated metabolic reprogramming and improved graft vascularization. This metabolic reprogramming enables granules to generate internal energy, sustaining chondrocyte viability in hypoxic environments while simultaneously promoting capillary infiltration. ADSC metabolic support and apoptosis inhibition are two critical roles that establish them as key mediators in the tracheal reconstruction process, especially in the challenging hypoxic environment.

The limits on long-term survival were primarily attributable to progressive airway obstruction caused by the accumulation of secretions. This highlights a functional limitation of the graft: incomplete mucociliary clearance remains a key challenge in long‑segment tracheal reconstruction. Postmortem examinations in both rabbit and porcine subjects revealed no evidence of graft collapse, stenosis, or tissue necrosis, while histopathological analysis of lung tissue demonstrated the absence of pneumonia or systemic infection (Suppl. Fig. 16). Longitudinal assessment of the graft revealed a progressive creeping regeneration pattern, with squamous epithelium migrating from the anastomotic margins toward the graft center over time. By 8 weeks, squamous coverage had extended to the quarter points, and scattered ciliated cells had also begun to appear at these sites, indicating ongoing functional differentiation. Although early re-epithelialization was observed within the grafts, the regenerated epithelium remained functionally immature, lacking sufficient mucociliary clearance to prevent local secretion accumulation across the segmental defect. To prevent unmitigated animal suffering, a Wesley-Croup score of ≥ 10 was selected as the strict humane endpoint, as scores above 10 reflect severe respiratory distress. While bronchoscopic clearance transiently restored airway patency during monitoring, performing repeated invasive interventions under general anesthesia was ethically impermissible in this animal model, which limited the follow-up duration.

While our 3D minichannel grafts successfully maintained structural integrity, several limitations must be noted. Experimentally, the observation period was insufficient to evaluate long-term ( > 1 year) biomechanical stability against late-stage tracheomalacia^3,5,10^, and the 3 cm defect in our porcine model did not reach the critical threshold ( > 6 cm or 50% tracheal length) typically requiring replacement in human clinical scenarios^8,48^. For direct clinical application, two main issues remain. First, ciliary regeneration across the graft midportion was slow, underscoring the challenge of establishing rapid functional re-epithelialization. Second, the slow degradation of PCL may restrict radial and longitudinal growth, limiting its immediate suitability for growing pediatric airways. To address these limitations, the required next steps include refining scaffold material compositions to match tissue growth^67^, integrating bioactive coatings or temporary bioresorbable stents to accelerate ciliary maturation, and evaluating long-segment repairs in large animal models receiving clinical-grade postoperative care.

In conclusion, this work establishes clinically translatable grafts for tracheal reconstruction by integrating 3D biomanufacturing, host-derived cartilage, and stem cell-mediated vascularization. By eliminating dependency on chondrocyte isolation and lengthy in vitro culture, our approach may address some barriers to clinical adoption. Further refinements in epithelial integration could enable human trials, potentially transforming the management of extensive tracheal defects.

## Methods

### Ethical statement

All animal experiments in this study complied with all relevant ethical regulations for animal testing and research. The experimental protocols were approved by the Laboratory Animal Ethics Committee of the Fourth Military Medical University (Approval No. 2023kq040). The reporting of all animal experiments in this study abides by the ARRIVE guidelines. This study did not involve human participants, human tissue collected directly by the investigators, or clinical trials. Human umbilical vein endothelial cells used in this study were commercially obtained from Lonza (CC2517A, Lonza, Switzerland).

### Considerations for clinical translation

Although this study was conducted in a preclinical academic setting, all the procedures were designed with clinical translation in mind. Scaffold fabrication and cell processing were carried out under ISO-class cleanroom conditions via standardized operating protocols (SOPs) to minimize contamination. Sterilization was achieved via 75% ethanol disinfection combined with UV irradiation, which was validated through sterility testing. Although the current experiments were not performed in a fully GMP-certified facility, the protocols established here are aligned with GMP principles, ensuring the transition toward clinical trials.

### Preparation of the caramel-based printing material

To prepare the caramel-based printing material, a mixture was formulated with sucrose, fructose, dextran, glucose, and distilled water at a ratio of 10:1:1:2:10 (e.g., 50 g of sucrose (Cat. #1.18302.010, GHTECH, China), 5 g of fructose (Cat. #D-809612, Macklin, China), 5 g of dextran (Cat. #D-806715, Macklin, China), 10 g of glucose (Cat. #1.18327.010, GHTECH, China), and 50 ml of distilled water). The components were thoroughly combined and dissolved by vigorous stirring. The mixture was then heated on an induction cooktop at 300 °C while continuously stirred with a glass rod until the solution became viscous and turned pale yellow. The caramel syrup was subsequently transferred into a glass beaker and subjected to high-temperature cross-linking in an oven at 100 °C for 8 h.

### Preparation of 3D-printed hollow C-shaped scaffolds

A 3D printer (PC Printer BR 151 S; Particle Cloud Biotechnology Co., Ltd., Xi’an, China) was used to print sugar ink at 0.25 MPa and 100 °C, creating a 15 mm × 12 mm scaffold. This scaffold was then molded into a C-shaped tracheal scaffold on a cylindrical mold. PCL (Cat. #440744, Sigma-Aldrich, USA) and PGS were dissolved in hexafluoroisopropanol (Cat. #H-811026, Macklin, China) at a 5% (w/v), with a mass ratio of PCL to PGS of 4:1. NaCl particles (Tianjin Yongda Chemical Reagent Co., Ltd., average diameter 30 μm) were added at a 1:3 ratio relative to the polymer. The molded caramel templates were dipped into the polymer/salt suspension (2, 4, or 6 times to achieve varying layer thicknesses), air-dried in a fume hood for 24 h, and subsequently immersed in deionized water for 12 h (with water replaced every 4 h) to thoroughly leach out the caramel template and NaCl particles, yielding porous PCL-PGS minichannel scaffolds.

### Pore interconnectivity measurement

The pore interconnectivity was calculated via Eq. (1):

1 $${{{\rm{pore}}}}\; {{{\rm{interconnectivity}}}}\left(\%\right)=\frac{{{{\rm{maximum}}}}\; {{{\rm{pore}}}}\; {{{\rm{volume}}}}}{{{{\rm{total}}}}\; {{{\rm{pore}}}}\; {{{\rm{volume}}}}}\times 100\%$$

The analysis was performed via CTAn software (VG Studio MAX 3.5) on nanoscale CT scans (Zeiss, Xradia 520 versa, Germany) with a resolution of 2 μm (sample size of 1 mm × 1 mm).

### Residual solvent analysis

The postfabrication scaffolds were lyophilized and analyzed via FTIR spectroscopy (Thermo Scientific Nicolet iS 5) to detect residual hexafluoroisopropanol. The spectra were collected over 64 scans at a resolution of 4 cm^−1^ (range: 4000–500 cm^−1^). The absence of C‒F bond peaks (1200–1250 cm^−1^) confirmed solvent elimination.

### Mechanical property testing

Mechanical characterization of C-shaped tracheal grafts (7 mm diameter, 1 cm length) with 2-, 4-, and 6-layer configurations was performed via a UTM5105 universal testing machine (ElectroForce 3200 Series II, USA). Quasistatic compression tests were conducted at a 0.5 mm/min displacement rate, with the maximum force recorded at 50% deformation. For tubular grafts, compression was applied radially until the lumen collapsed to 50% deformation. In contrast, for the cartilage composite (amorphous cartilage tissue), conventional compression was used, with the maximum force defined as the force required to crush the sample. Uniaxial tensile testing was performed at a stretching speed of 1 mm/min by gripping both ends of the minichannels until complete structural failure occurred.

### Endotoxin detection

Endotoxin contamination of reagent-grade PCL (Cat. #440744, Sigma‒Aldrich, USA) was assessed via an endotoxin detection kit (Cat. #A39552, Thermo Fisher Scientific, China). A standard curve with endotoxin concentrations ranging from 0.1 EU/ml to 1 EU/ml was first established. The samples (1 g/ml in endotoxin-free water) were incubated at 37 °C for 24 h. The supernatant was then analyzed spectrophotometrically at 405 nm.

### Permeability performance test

To assess the permeability of minichannel scaffolds, a diffusion test was conducted using red ink as a model solute. Scaffolds with 2, 4, and 6 layers were tested. Each scaffold was injected with 500 µl of red ink and then immersed in 50 ml of distilled water. The rate of ink diffusion was measured by monitoring the absorbance at 540 nm with a spectrophotometer.

### Isolation and culture of primary cells

Primary rabbit chondrocytes and adipose-derived stem cells (ADSCs) were isolated from ear cartilage and inguinal fat pads of New Zealand White rabbits under aseptic conditions. Cartilage tissue was digested with 0.25% trypsin-EDTA (Cat. #25200-056; Thermo Fisher Scientific, USA) for 30 min, followed by 0.2% collagenase type II (Cat. #17101015; Thermo Fisher Scientific, USA) for 6–8 h at 37 °C. Adipose tissue was digested with 0.1% collagenase type I (Cat. #17100017; Thermo Fisher Scientific, USA) for 45 min at 37 °C. Primary minipig ADSCs were similarly isolated from male Bama minipigs. Chondrocytes were cultured in DMEM (Cat. #11320033; Thermo Fisher Scientific, USA) supplemented with 15% fetal bovine serum (FBS) (Cat. #A5256701; Thermo Fisher Scientific, USA), whereas ADSCs were expanded in α-MEM (Cat. #12571063; Thermo Fisher Scientific, USA) containing 10% FBS. Cells at passages 1-3 (P1-P3) were used for subsequent graft fabrication and in vitro evaluations.

### Scanning electron microscope (SEM) analysis

The scaffold microstructure was characterized via field-emission SEM (S-4800, Hitachi, Japan). The samples were freeze-dried for 24 h, sputter-coated with 10 nm gold-palladium, and imaged at an accelerating voltage of 5 kV. High-resolution images (500x and 5000x magnification) were analyzed via ImageJ software to quantify the wall thickness and surface porosity.

### Preparation of granulated cartilage

Auricular cartilage was harvested from male New Zealand White rabbits under sterile conditions. The cartilage was cut into 0.5 × 0.5 cm^2^ fragments, washed three times with PBS (Cat. #WHB823F181, Pricella, China), placed into a 25-mL grinding jar, and flash-frozen in liquid nitrogen for 3 min without cryoprotectants. The frozen tissue was immediately homogenized using a high-throughput tissue grinder (Cat. #TL2010S, DHS Life Science & Technology Co., Ltd., China) at an oscillation speed of 1,000 oscillations per minute for 20, 30, or 40 s to produce cartilage granules.

### Western blot analysis

Auricular cartilage granules (100 mg) co-cultured with ADSCs for 4 weeks at varying ratios (1:1, 3:1, 6:1) were homogenized in liquid nitrogen. Total protein was extracted using RIPA lysis buffer (Cat. #WB3100, NCM Biotech, China) supplemented with 1% protease and phosphatase inhibitor cocktail (Cat. #P001, NCM Biotech, China) for 3 h on ice. Protein concentrations were determined using a BCA assay kit (Cat. #23225, Thermo Fisher Scientific, USA). Equal amounts of protein (30 μg per lane) were resolved on 10% SDS-PAGE gel gradients and transferred onto PVDF membranes (Cat. #SSC-PVDF-45, SanseBio, China). Membranes were blocked with 5% non-fat milk in TBST for 1 h at room temperature and incubated overnight at 4 °C with primary antibodies: anti-SOX9 (Cat. #67439-1-Ig, Proteintech, 1:1000), anti-Col II (Cat. #28459-1-AP, Proteintech, 1:1000), and anti-β-actin (Cat. #20536-1-AP, Proteintech, 1:1000). After washing with TBST, membranes were incubated with HRP-conjugated secondary antibodies (Cat. #SA00001-1 and Cat. #SA00001-2, Proteintech, 1:5000) for 1 h at room temperature. Protein bands were visualized using an ECL substrate (Cat. #P10100, NCM Biotech, China) and imaged on a ChemiDoc Imaging System (Bio-Rad, USA), with band intensities quantified using ImageJ software.

### Preparation of tissue-engineered tracheal cartilage rings

Autologous platelet-rich plasma (PRP) was prepared using a two-step centrifugation protocol: whole blood was collected into sodium citrate tubes, centrifuged at 250 × *g* for 10 min to collect the plasma layer, and further centrifuged at 1200 × *g* for 10 min to pellet platelets, which were resuspended in one-tenth of the original plasma volume. Third-passage ADSCs were harvested and resuspended in the prepared PRP. Granulated cartilage was thoroughly mixed with PRP containing ADSCs at a 3:1 wet weight ratio (cartilage granules to ADSCs, with a final PRP concentration of 10% v/v). The composite was loaded into a sterile 1 ml syringe and injected directly into the porous 3D-printed minichannels to construct C-shaped cartilage rings. Grafts loaded with cartilage granules and PRP without ADSCs served as the control group (CGP).

### Three-dimensional CT reconstruction analysis

Micro-CT (Always Imaging, AX3000-D, China) was utilized to scan and reconstruct C-shaped tracheal scaffolds before and after the injection of cartilage granules, with enhanced image reconstruction on the basis of the density of different components performed via VG Studio MAX 3.5 software.

### Rheological characterization of PRP-cartilage composites

The viscoelastic and rheological properties of PRP-cartilage granule composites with varying PRP volume fractions (5 %, 10%, and 20%) were evaluated using a parallel-plate rheometer (MCR 102e, Anton Paar, Austria) equipped with a 25 mm diameter plate at a gap of 1.0 mm. Dynamic strain sweeps (0.01% to 100%) were performed at a constant frequency of 1.0 Hz and a temperature of 25 °C to determine the storage modulus (*G’*) and loss modulus (*G”*). Shear-rate sweeps were conducted across 0.01–100 s^−^^1^ to evaluate viscosity coefficients and yield stresses.

### Evaluation of injection accuracy and shape fidelity

Composite hydrogels containing varying PRP concentrations (5%, 10%, and 20% v/v) were injected to form predefined geometric and letter patterns. Standardized macro-photographs were analyzed using ImageJ software (v.1.53, NIH, USA) to measure the experimental projected area (A_exp_) relative to the target area (A_target_). Injection accuracy was calculated via Eq. (2):

2 $${{{\rm{Injection\; accuracy}}}}\left(\%\right)=\left(1-\frac{\left|{A}_{\exp }-{A}_{{target}}\right|}{{A}_{{target}}}\right)\times 100\%$$

### Scaffold-free CGPA Graft preparation and testing

Scaffold-free CGPA grafts were prepared by injecting 1 ml of the cartilage granule–ADSC–PRP mixture into the abdominal fascia of rabbits. After 2 weeks of prevascularization, the grafts were harvested for mechanical testing (5 × 5 × 1.5 mm). Native tracheal cartilage samples of the same size were used as controls. Uniaxial compression tests were performed at a 50% strain rate, and the maximum compressive force was recorded.

### Animal experiments

All animal experiments were approved by the Laboratory Animal Ethics Committee of the Fourth Military Medical University (Approval No. 2023kq040) and conducted in compliance with institutional animal care guidelines and the ARRIVE guidelines. New Zealand White rabbits, Bama minipigs, and BALB/c nude mice were procured from Chengdu Dossy Experimental Animals Co., Ltd. Only male animals were used in this preclinical feasibility study. Sex was not considered as an independent experimental variable in the study design or data analysis, as the study focused on scaffold mechanical stenting, vascularized fascia integration, and chondrocyte metabolic responses independent of sex differences.

### Animal welfare monitoring and euthanasia

All animals were housed under standard controlled conditions (22 ± 2 °C, 55 ± 10% humidity, 12 h light/dark cycle) with free access to water and a standard commercial diet provided by the licensed supplier (Chengdu Dossy Experimental Animals Co., Ltd., license no. SCXK (Chuan) 2024-0028). Animal welfare and clinical conditions were monitored daily (at 24 h intervals) by two blinded experimental animal researchers. Evaluation parameters included respiratory function (assessed via an adapted Wesley-Croup scale^68^, Supplementary Table 1), general behavior, activity levels, and food/water intake. Humane endpoints were strictly defined as a Wesley-Croup score ≥ 10, severe respiratory distress, unresponsiveness, or weight loss exceeding 20%. All animals were euthanized according to protocols approved by the Institutional Animal Care and Use Committee. All experimental animals were euthanized with an overdose of pentobarbital sodium. In all animal cases, death was confirmed by the absence of heartbeat, respiration, corneal reflex, and response to toe pinch.

### Rabbit tracheal defect model

Male New Zealand White rabbits (6 months old, 3.5−4.5 kg) were randomly allocated to the CGP (*n* = 6) or CGPA (*n* = 12) group. The animals were anesthetized with intramuscular injection of xylazine hydrochloride (0.05 mg/kg) and 3% pentobarbital sodium (15 mg/kg). C-shaped scaffolds loaded with cartilage granules were prevascularized ectopically under the platysma muscle for 2 weeks. Subsequently, a 20-mm circumferential tracheal defect was created, and the prevascularized graft was implanted via end-to-end anastomosis using 5-0 absorbable sutures. Surgical sites were checked for airtightness, disinfected with iodophor, and closed in layers. After the operation, penicillin (2 × 10^4^ U/kg) was administered once daily for 3 days.

### Porcine experiments

Male Bama minipigs (4–5 months old, 12–15 kg, *n* = 8) were used to assess the tracheal scaffold in a large animal model. The animals were anesthetized with intramuscular injection of xylazine hydrochloride (0.05 mg/kg) and 3% pentobarbital sodium (15 mg/kg). Autologous costal cartilage (total length of approximately 12 cm) was harvested from the unilateral 14th and 15th floating ribs and ground for 30 s to prepare cartilage granules. The granules were mixed with autologous PRP and allogeneic ADSCs at a 3:1 ratio, injected into 3D-printed minichannels, and prevascularized subcutaneously for 2 weeks. Subsequently, a 30 mm segmental tracheal defect was created, and the prevascularized graft was implanted via end-to-end anastomosis using 4−0 absorbable sutures. Surgical sites were checked for airtightness, disinfected with iodophor, and closed in layers. After the operation, penicillin (2 × 10^4^ U/kg) was administered once daily for 3 days.

### Nude mouse experiments

Male BALB/c nude mice (6−8 weeks old, 18–22 g, *n* = 9) were randomly divided into three groups (*n* = 3 per group) to evaluate in vivo tissue integration of the cartilage granule composites at different ADSC-to-cartilage ratios. The animals were anesthetized via intraperitoneal injection of 1% pentobarbital sodium (50 mg/kg). The surgical plane of anesthesia was confirmed by the absence of pedal withdrawal reflex. Approximately 1 ml of the cartilage granule composite was implanted subcutaneously into the dorsal region of each mouse. The surgical incisions were disinfected and closed. Following a 2-week incubation period, the ectopic implants were harvested to assess the morphological integration and matrix remodeling of the cartilage granules.

### Gross and quantitative analysis

Images of the tracheal opening lumens were acquired via a standardized imaging setup. The lumen area was measured via ImageJ software, and percentage of the lumen opening area was calculated via Eq. (3):

3 $${{{\rm{Opening}}}}\; {{{\rm{area}}}}\; {{{\rm{ratio}}}}\; {{{\rm{of}}}}\; {{{\rm{the}}}}\; {{{\rm{graft}}}}\; {{{\rm{lumen}}}}(\%)=\frac{{{{{\rm{Lumen}}}}\; {{{\rm{Area}}}}}_{{post}}\,}{{{{{\rm{Lumen}}}}\; {{{\rm{Area}}}}}_{{pre}}}\times 100\%$$

The percentage of stenosis was recorded for each sample.

### Quantitative analysis of collagen, DNA, and GAG

To quantify changes in cartilage matrix content in vivo, 25 mg of tissue was collected from minichannels at different time points. Collagen, DNA, and GAG contents were measured via specific assays. Collagen was assayed via the Sircol Collagen Assay Kit (Cat. #S1000, Biocolor, UK). The tissue was ground in liquid nitrogen, digested with pepsin, and purified according to the manufacturer’s instructions. The total collagen content was calculated from a standard curve (μg/mg tissue). DNA was quantified via a DNA quantification kit (Cat. #250221, Qiagen, USA). The tissue was ground, digested with proteinase K, and purified. The DNA concentration was measured at 260 nm (ng/mg tissue). GAG content was measured via the Blyscan GAG Assay Kit (Cat. #B1000, Biocolor, UK). The tissue was ground, digested with papain, and analyzed. The GAG content was determined from a standard curve (μg/mg tissue).

### Histological staining

The harvested samples were fixed with 4% paraformaldehyde (Cat. #PC-00005, Plant Chem Medicine, China) and then embedded in paraffin for tissue sectioning. The sections on the slides were cut to a thickness of 5−8 μm. After dewaxing and rehydration, H&E staining (Cat. #G1120, Solarbio, China) was performed to characterize the cell distribution and morphology. Alcian blue staining (Cat. #G1560, Solarbio, China) was used for the analysis and detection of cartilage matrix formation.

### Immunohistochemical staining

The paraffin-embedded sections were dewaxed, rehydrated, and subjected to antigen retrieval. After being blocked with goat serum, the sections were incubated with a primary Col II antibody (Cat. #28459-1-AP; Proteintech, China; 1:1000) at 4 °C overnight, followed by three washes with PBS and incubation with a secondary antibody at room temperature. Staining was visualized via DAB (Cat. #DA1016, Solarbio, China) chromogenic solution.

### Immunofluorescence staining and quantitative analysis

Paraffin sections were dewaxed in xylene, rehydrated through graded ethanol, and subjected to antigen retrieval in 10 mM sodium citrate buffer (pH 6.0) at 95 °C for 15 min. Sections were blocked with 5% normal goat serum (Solarbio, China) for 1 h at room temperature and incubated overnight at 4 °C with primary antibodies against CD31 (Cat. #NB600-562, Novus, USA, 1:200), IL-1β (Cat. #66737-1-Ig, Proteintech, China, 1:300), Ac-Tubulin (Cat. #66200-1-Ig, Proteintech, China, 1:300), TNF-α (Cat. #26405-1-AP, Proteintech, China, 1:400), and CK (Cat. #ab961, Abcam, UK, ready-to-use) overnight at 4 °C, followed by incubation with Alexa Fluor-conjugated secondary antibodies (Cat. #A-11001 / Cat. #A-11012, Thermo Fisher Scientific, 1:500) for 1 h at room temperature in the dark. Nuclei were counterstained with DAPI (Cat. #PC-90017, PCM, 1:1000). Mean fluorescence intensities and CD31-positive vessel counts were quantified across three randomly selected fields per section using ImageJ software.

### Proteomic sequencing

Frozen graft tissues (CGP and CGPA at 2 weeks post-implantation, *n* = 3 independent biological samples per group) were ground under liquid nitrogen, lysed in buffer containing 1% SDS and 1% protease inhibitor cocktail, and cleared by centrifugation at 12,000 × *g* (4 °C, 10 min). Proteins were quantified by BCA assay, precipitated with pre-cooled acetone, redissolved in 200 mM TEAB, reduced with 5 mM DTT (56 °C, 30 min), alkylated with 11 mM iodoacetamide (room temperature, dark, 15 min), and digested overnight with trypsin at a 1:50 mass ratio. Tryptic peptides were dissolved in 0.1% formic acid and loaded onto a NanoElute UHPLC system (Bruker Daltonics) equipped with a 25 cm reversed-phase column (100 μm i.d.) at a flow rate of 500 nL/min. Elution was performed with a gradient of solvent B (0.1% formic acid in acetonitrile): 6%–24% for 14 min, 24–35% for 2 min, 35–80% for 2 min, and 80% for 2 min. Peptides were analyzed on a timsTOF Pro 2 mass spectrometer (Bruker Daltonics) using a capillary source (1.75 kV) in dia-PASEF mode. MS1 scans ranged from 300 to 1500 m/z, and 20 PASEF MS/MS scans were acquired per cycle (scan range: 400–850 m/z, isolation window: 7 m/z).

Raw DIA data were searched using DIA-NN (v1.8) against the *Leporidae* database (Leporidae_9979_UP_20241014_seqkit. fasta, 45,516 entries). Trypsin/P was set as the cleavage enzyme (up to 1 missed cleavage; minimum peptide length: 7 residues). N-terminal Met excision and Cys carbamidomethylation were set as fixed modifications. Precursor and protein FDRs were controlled at < 1%. Relative quantification values were log₂-transformed; differentially expressed proteins (DEPs) were selected based on Student’s *t*-test (*p* < 0.05) and fold change (FC > 1.5 or < 1/1.5). Functional GO/KEGG enrichment analyses were evaluated using two-tailed Fisher’s exact test with Benjamini–Hochberg adjustment (*p* < 0.05).

### Hexokinase fluorescence staining

Chondrocytes were seeded at a density of 1 × 10⁶ cells per well in 6-well plates. The experiment was divided into three groups: (1) control group: chondrocytes cultured under normal conditions (21% O₂, 5% CO₂); (2) hypoxia group: chondrocytes cultured under hypoxic conditions (1% O₂, 5% CO₂) for 3 days; and (3) coculture group: chondrocytes cultured under hypoxic conditions with ADSCs in a transwell system for 3 days, with ADSCs seeded at a density of 1 × 10⁵ cells per insert. After 3 days, the cells were washed three times with PBS, fixed in 4% paraformaldehyde for 15 min at room temperature, washed again with PBS, and incubated with primary antibodies against Hexokinase 2 (HK2) (Cat. #arp54303, AvivaSysBio, USA, 1:200) overnight at 4 °C, followed by incubation with Alexa Fluor-conjugated secondary antibodies for 1 h at room temperature. Fluorescence images were captured via a fluorescence microscope, and the fluorescence intensity was quantified via ImageJ software. The average fluorescence intensity was calculated for each group.

### Determination of the hexokinase content

Following the respective in vitro treatments under normoxic and hypoxic conditions, chondrocytes were harvested, washed with pre-cooled PBS, and lysed to collect cellular supernatant fractions in accordance with the manufacturer’s instructions. Hexokinase (HK) activity was quantitatively determined using a commercial Hexokinase Activity Assay Kit (Cat. #E-BC-K610-M, Elabscience, China). Briefly, blank controls, standard wells containing hexokinase standards at known concentrations, and sample wells containing the extracted supernatant were established in 96-well plates. After the reaction system was thoroughly mixed and incubated at 37 °C for 10 min, the optical density (OD) was measured spectrophotometrically at 450 nm using a microplate reader. Enzyme activity was calculated based on the standard curve and normalized to total protein content.

### Determination of the extracellular acidification rate (ECAR)

To evaluate cellular glycolytic function, the ECAR of chondrocytes across different culture groups was assessed using an Extracellular Acidification Rate (ECAR) Fluorometric Assay Kit (Cat. #E-BC-F069, Elabscience, China). After the designated in vitro culture periods, the culture medium was gently aspirated, and the cells were washed twice with pre-warmed PBS. The wells were subsequently replenished with the assay working solution containing the specific fluorescent probe. Following precise incubation and stabilization according to the manufacturer’s protocol, fluorescence intensity levels were measured using a multi-mode fluorescence microplate reader. The ECAR values were quantified and calculated based on standard calibration parameters.

### Statistics and reproducibility

All presented data originate from distinct biological samples. Quantitative image analysis was performed using ImageJ software (v1.48 v, NIH, Bethesda, MD, USA). Statistical analyses were conducted using GraphPad Prism 9.0 (GraphPad Software, La Jolla, CA, USA). Data normality was verified prior to parametric testing. Comparisons between two groups were performed using two-tailed Student’s *t*- tests. Comparisons among multiple groups were analyzed using one-way analysis of variance (ANOVA) followed by Tukey’s post-hoc test. Non-parametric clinical Wesley-Croup scores were evaluated using two-tailed Mann- Whitney *U* tests. Quantitative data are expressed as mean ± SD. Statistical significance was set at *p* < 0.05.

This study was designed to ensure data reliability and transparency. Regarding sample size, no statistical method was used to predetermine sample size. Sample sizes were selected on the basis of preliminary experiments, previous studies using comparable models, and experimental feasibility. No data were excluded from the analyses. Samples and animals were randomly assigned at the time of grouping to minimize any potential bias. For all experiments, investigators were strictly blinded to group allocation during data collection and analysis. All experiments were repeated at least three times independently, yielding consistent results, and exact sample sizes are defined in the respective figure legends.

### Reporting summary

Further information on research design is available in the Nature Portfolio Reporting Summary linked to this article.

## Supplementary information

Supplementary Information

Description of Additional Supplementary Files

Supplementary Movie 1

Supplementary Movie 2

Supplementary Movie 3

Supplementary Movie 4

Supplementary Movie 5

Supplementary Movie 6

Supplementary Movie 7

Reporting Summary

Transparent Peer Review file

## Source data

Source Data

## Data Availability

Source data are provided with this paper. Source data for the graphs and charts in all Figures and Supplementary Figs. are provided as Source Data files. The mass spectrometry proteomics data have been deposited to the ProteomeXchange Consortium via the PRIDE partner repository with the dataset identifier PXD067839. These data are publicly accessible without restriction. Source data are provided with this paper.

## References

[1] He, J. et al. End-to-side anastomosis in complex tracheal resection and reconstruction: a case series study. *Transl. Lung Cancer Res.* **11**, 165–172 10.21037/tlcr-22-32 (2022). PMC8902089 35280311

[2] Adamo, D., Galaverni, G., Genna, V. G., Lococo, F. & Pellegrini, G. The growing medical need for tracheal replacement: reconstructive strategies should overcome their limits. *Front. Bioeng. Biotechnol.* **10** 10.3389/fbioe.2022.846632 (2022). PMC9132048 35646864

[3] Martinod, E. et al. Feasibility of bioengineered tracheal and bronchial reconstruction using stented aortic matrices. *Jama* **319** 10.1001/jama.2018.4653 (2018). PMC6134437 29800033

[4] Xia, X., Zhu, X., Zhu, Y., Diao, W. & Chen, X. Sleeve resection with end-to-end anastomosis in the reconstruction of tracheal defects exceeding six rings: a clinical feasibility study and safety assessment. *Front. Surg.* **10** 10.3389/fsurg.2023.1229522 (2024). PMC11055456 38681138

[5] Grillo, H. C. Tracheal replacement: a critical review. *Ann. Thorac. Surg.* **73**, 1995–2004 10.1016/s0003-4975(02)03564-6 (2002). 12078821

[6] Musgrove, K. A., McCarthy, D. & de Biasi, A. R. Aortic arch replacement and autologous pericardial tracheal patch for an aorto-tracheal fistula. *J. Card. Surg.* **37**, 5472–5474 10.1111/jocs.16912 (2022). PMC10087843 36150146

[7] Tan, Z. H. et al. Partial decellularization eliminates immunogenicity in tracheal allografts. *Bioeng. Transl. Med.* **8** 10.1002/btm2.10525 (2023). PMC10487308 37693070

[8] Ren, J. et al. Reconstruction of the trachea and carina: Surgical reconstruction, autologous tissue transplantation, allograft transplantation, and bioengineering. *Thorac. Cancer* **13**, 284–295 10.1111/1759-7714.14315 (2022). PMC8807246 35023311

[9] Ludwig, C. Prophylaxis and management of postoperative complications after tracheobronchial surgery. *J. Thorac. Dis.* **12**, 6179–6184 10.21037/jtd.2020.03.63 (2020). PMC7656419 33209456

[10] Wei, S. et al. Tissue-engineered tracheal implants: advancements, challenges, and clinical considerations. *Bioengi Transl. Med.* **9** 10.1002/btm2.10671 (2024). PMC11256149 39036086

[11] Mammana, M., Bonis, A., Verzeletti, V., Dell’Amore, A. & Rea, F. Tracheal Tissue Engineering: Principles and State of the Art. *Bioengineering* **11** 10.3390/bioengineering11020198 (2024). PMC10886658 38391684

[12] Xu, X. et al. Application of tissue engineering techniques in tracheal repair: a bibliometric study. *Bioengineered* **14** 10.1080/21655979.2023.2274150 (2023). PMC10629433 37927226

[13] McPhail, M. J., Janus, J. R. & Lott, D. G. Advances in regenerative medicine for otolaryngology/head and neck surgery. *BMJ*10.1136/bmj.m718 (2020). 32349978

[14] Hiwatashi, S., Iwai, R., Nakayama, Y., Moriwaki, T. & Okuyama, H. Successful tracheal regeneration using biofabricated autologous analogues without artificial supports. *Sci. Rep.* **12** 10.1038/s41598-022-24798-y (2022). PMC9700768 36434016

[15] Zhao, L. et al. Engineered tissue-stent biocomposites as tracheal replacements. *Tissue Eng. Part A* **22**, 1086–1097 10.1089/ten.TEA.2016.0132 (2016). PMC5312617 27520928

[16] Salzmann, G. M., Niemeyer, P., Hochrein, A., Stoddart, M. J. & Angele, P. Articular cartilage repair of the knee in children and adolescents. *Orthopaedic J. Sports Med.* **6** 10.1177/2325967118760190 (2018). PMC5858627 29568785

[17] Marmotti, A. et al. Autologous cartilage fragments in a composite scaffold for one stage osteochondral repair in a goat model. *Eur. Cells Mater.* **26**, 15–32 10.22203/eCM.v026a02 (2013). 23913344

[18] Salzmann, G. M., Ossendorff, R., Gilat, R. & Cole, B. J. Autologous minced cartilage implantation for treatment of chondral and osteochondral lesions in the knee joint: an overview. *Cartilage* **13**, 1124S–1136S 10.1177/1947603520942952 (2020). PMC8808955 32715735

[19] Brown, N. E., Ellerbe, L. R., Hollister, S. J. & Temenoff, J. S. Development and characterization of heparin-containing hydrogel/3D-printed scaffold composites for craniofacial reconstruction. *Ann. Biomed. Eng.* **52**, 2287–2307 10.1007/s10439-024-03530-z (2024). 38734845

[20] Olesen, M. L. et al. No effect of platelet-rich plasma injections as an adjuvant to autologous cartilage chips implantation for the treatment of chondral defects. *Cartilage* **13**, 277S–284S 10.1177/1947603519865318 (2019). PMC8804778 31327251

[21] Yuan, P. et al. Extracellular vesicles derived from starving BMSCs enhance survival of chondrocyte aggregates in grafts by attenuating chondrocyte apoptosis and enabling stable cartilage regeneration for craniofacial reconstruction. *Acta Biomater.* **140**, 659–673 10.1016/j.actbio.2021.12.011 (2022). 34902618

[22] Li, Z. et al. Angiogenic potential of human bone marrow-derived mesenchymal stem cells in chondrocyte brick-enriched constructs promoted stable regeneration of craniofacial cartilage. *Stem Cells Transl. Med* **6**, 601–612 10.5966/sctm.2016-0050 (2017). PMC5442805 28191761

[23] Yilmaz, H., Abdulazez, I. F., Gursoy, S., Kazancioglu, Y. & Ustundag, C. B. Cartilage Tissue Engineering in Multilayer Tissue Regeneration. *Ann. Biomed. Eng.* 10.1007/s10439-024-03626-6 (2024). 39400772

[24] Shi, J. & Du, G. Metabolic reprogramming of glycolysis favors cartilage progenitor cells rejuvenation. *Joint Bone Spine* **91** 10.1016/j.jbspin.2023.105634 (2024). 37684000

[25] Liu, X. et al. An in situ photocrosslinkable platelet rich plasma – Complexed hydrogel glue with growth factor controlled release ability to promote cartilage defect repair. *Acta Biomaterialia* **62**, 179–187 10.1016/j.actbio.2017.05.023 (2017). 28501713

[26] Jiang, G. et al. A 3D-printed PRP-GelMA hydrogel promotes osteochondral regeneration through M2 macrophage polarization in a rabbit model. *Acta Biomaterialia* **128**, 150–162 10.1016/j.actbio.2021.04.010 (2021). 33894346

[27] Zhou, T. et al. A hyaluronic acid/platelet-rich plasma hydrogel containing MnO2 nanozymes efficiently alleviates osteoarthritis in vivo. *Carbohydrate Polymers* **292** 10.1016/j.carbpol.2022.119667 (2022). 35725167

[28] Liu, L. et al. Tissue-engineered composite tracheal grafts create mechanically stable and biocompatible airway replacements. *J. Tissue Eng.* **13** 10.1177/20417314221108791 (2022). PMC9243572 35782992

[29] Hirsch, W. D. B. et al. An analysis of the biocompatibility, cytotoxicity, and bone conductivity of polycaprolactone: an in vivo study. *Polymers* **16** 10.3390/polym16162271 (2024). PMC11359069 39204491

[30] Li, H. et al. 3D-printed PCL scaffolds combined with injectable sodium alginate/magnesium-doped mesoporous bioactive glass nanosphere hydrogel for meniscus regeneration: In vitro, In vivo, and multiomics-based therapeutic analyses. *Bioact. Mater.* **48**, 313–335 10.1016/j.bioactmat.2025.02.016 (2025). PMC11889361 40060143

[31] Shai, S.-E. et al. Long-term survival and regeneration following transplantation of 3D-printed biodegradable PCL tracheal grafts in large-scale porcine models. *Bioengineering* **11** 10.3390/bioengineering11080832 (2024). PMC11351403 39199790

[32] Wang, Y., Ameer, G. A., Sheppard, B. J. & Langer, R. A tough biodegradable elastomer. *Nat. Biotechnol.* **20**, 602–606 10.1038/nbt0602-602 (2002). 12042865

[33] Wu, W., Allen, R. A. & Wang, Y. Fast-degrading elastomer enables rapid remodeling of a cell-free synthetic graft into a neoartery. *Nat. Med* **18** 1148–1153 10.1038/nm.2821 (2012). PMC3438366 22729285

[34] Huang, S. et al. A perfusable, multifunctional epicardial device improves cardiac function and tissue repair. *Nat. Med.* **27**, 480–490 10.1038/s41591-021-01279-9 (2021). 33723455

[35] Wu, W., Jia, S., Chen, W., Liu, X. & Zhang, S. Fast degrading elastomer stented fascia remodels into tough and vascularized construct for tracheal regeneration. *Mater. Sci. Eng. C. Mater. Biol. Appl.* **101**, 1–14 10.1016/j.msec.2019.02.108 (2019). 31029302

[36] Bunnell, B. A. Adipose tissue-derived mesenchymal stem cells. *Cells* **10** 10.3390/cells10123433 (2021). PMC8700397 34943941

[37] Song, Y. et al. Adipose-derived mesenchymal stem cell-derived exosomes biopotentiated extracellular matrix hydrogels accelerate diabetic wound healing and skin regeneration. *Adv. Sci.* **10** 10.1002/advs.202304023 (2023). PMC10602544 37712174

[38] Zhu, C. et al. Adipose-derived stem cells promote glycolysis and peritoneal metastasis via TGF-β1/SMAD3/ANGPTL4 axis in colorectal cancer. *Cell. Mole. Life Sci.* **81** 10.1007/s00018-024-05215-1 (2024). PMC11033247 38643448

[39] Sharma, D. et al. Upgrading prevascularization in tissue engineering: a review of strategies for promoting highly organized microvascular network formation. *Acta Biomaterialia* **95**, 112–130 10.1016/j.actbio.2019.03.016 (2019). 30878450

[40] Zhang, F. et al. An extra-erythrocyte role of haemoglobin body in chondrocyte hypoxia adaption. *Nature* **622**, 834–841 10.1038/s41586-023-06611-6 (2023). PMC10600011 37794190

[41] Feng, M., Ahmed, K. H., Punjabi, N. & Inman, J. C. A Contemporary review of trachea, nose, and ear cartilage bioengineering and additive manufacturing. *Biomimetics* **9** 10.3390/biomimetics9060327 (2024). PMC11202182 38921207

[42] Zhou, G. et al. In Vitro Regeneration of Patient-specific Ear-shaped Cartilage and Its First Clinical Application for Auricular Reconstruction. *EBioMedicine* **28**, 287–302 10.1016/j.ebiom.2018.01.011 (2018). PMC5835555 29396297

[43] Zhang, B. et al. A novel decellularized trachea preparation method for the rapid construction of a functional tissue engineered trachea to repair tracheal defects. *J. Mater. Chem. B* **10**, 4810–4822 10.1039/d1tb02100a (2022). 35237780

[44] Khalid, U. et al. Surgical innovations in tracheal reconstruction: a review on synthetic material fabrication. *Medicina* **60** 10.3390/medicina60010040 (2023). PMC10820818 38256300

[45] Bhora, F. Y. et al. Circumferential three-dimensional-printed tracheal grafts: research model feasibility and early results. *Ann. Thorac. Surg.* **104**, 958–963 10.1016/j.athoracsur.2017.03.064 (2017). 28619543

[46] Nahumi, A., Peymani, M., Asadi, A., Abdolmaleki, A. & Panahi, Y. Decellularized tracheal scaffold as a promising 3D scaffold for tissue engineering applications. *Tissue Cell* **85** 10.1016/j.tice.2023.102258 (2023). 37918216

[47] Zhang, X. et al. A 3D-printed cuttlefish bone elastomeric sponge rapidly controlling noncompressible hemorrhage. *Small* **20**, e2307041 10.1002/smll.202307041 (2024). 38072798

[48] Soriano, L. et al. Development and clinical translation of tubular constructs for tracheal tissue engineering: a review. *Eur. Resp. Rev.* **30** 10.1183/16000617.0154-2021 (2021). PMC9488721 34750116

[49] Khalid, T. et al. Development of tissue-engineered tracheal scaffold with refined mechanical properties and vascularisation for tracheal regeneration. *Front. Bioeng Biotechnol.* **11** 10.3389/fbioe.2023.1187500 (2023). PMC10281188 37346796

[50] Guimaraes, A. B. et al. Evaluation of a physical-chemical protocol for porcine tracheal decellularization. *Transpl. Proc.* **51**, 1611–1613 10.1016/j.transproceed.2019.01.042 (2019). 31155202

[51] Yang, B. et al. Recapitulating hypoxic metabolism in cartilaginous organoids via adaptive cell-matrix interactions enhances histone lactylation and cartilage regeneration. *Nat. Commun.* **16** 10.1038/s41467-025-57779-6 (2025). PMC11923171 40108220

[52] Rademakers, T., Horvath, J. M., Blitterswijk, C. A. & LaPointe, V. L. S. Oxygen and nutrient delivery in tissue engineering: approaches to graft vascularization. *J. Tissue Eng. Regenerative Med.* **13**, 1815–1829 10.1002/term.2932 (2019). PMC6852121 31310055

[53] Yang, Y. et al. Secretive derived from hypoxia preconditioned mesenchymal stem cells promote cartilage regeneration and mitigate joint inflammation via extracellular vesicles. *Bioact. Mater.* **27**, 98–112 10.1016/j.bioactmat.2023.03.017 (2023). PMC10063382 37006826

[54] Simonaro, C. M. et al. Mechanism of Glycosaminoglycan-Mediated Bone and Joint Disease. * Am. J. Pathol.* **172**, 112–122 10.2353/ajpath.2008.070564 (2008). PMC2189614 18079441

[55] Leal, A. F. et al. Mucopolysaccharidoses: cellular consequences of glycosaminoglycans accumulation and potential targets. *Int. J. Mol. Sci.* **24** 10.3390/ijms24010477 (2022). PMC9820209 36613919

[56] Everts, P., Onishi, K., Jayaram, P., Lana, J. F. & Mautner, K. Platelet-Rich Plasma: New Performance Understandings and Therapeutic Considerations in 2020. *Int. J. Mol. Sci.* **21** 10.3390/ijms21207794 (2020). PMC7589810 33096812

[57] Mamun, A. A., Shao, C., Geng, P., Wang, S. & Xiao, J. Recent advances in molecular mechanisms of skin wound healing and its treatments. *Front. Immunol.* **15** 10.3389/fimmu.2024.1395479 (2024). PMC11148235 38835782

[58] Park, J. H. et al. 3D bioprinting of a trachea-mimetic cellular construct of a clinically relevant size. *Biomaterials* **279** 10.1016/j.biomaterials.2021.121246 (2021). PMC8663475 34775331

[59] Dang, L. H. et al. Partial decellularized scaffold combined with autologous nasal epithelial cell sheet for tracheal tissue engineering. *Int. J. Mol. Sci.* **22** 10.3390/ijms221910322 (2021). PMC8508999 34638663

[60] Kim, I. G. *et al*. Transplantation of a 3D-printed tracheal graft combined with iPS cell-derived MSCs and chondrocytes. *Sci. Rep.* **10** 10.1038/s41598-020-61405-4 (2020). PMC7062776 32152475

[61] Liu, C. S. et al. Preparation and evaluation of a silk fibroin–polycaprolactone biodegradable biomimetic tracheal scaffold. *J. Biomed. Mater. Res. Part B: Appl. Biomater.* **110**, 1292–1305 10.1002/jbm.b.35000 (2022). 35061311

[62] Chen, Y. et al. Multiplexed self-adaptable Janus hydrogels rescue epithelial malfunction to promote complete trachea repair. *Nat. Commun.* **16** 10.1038/s41467-025-61135-z (2025). PMC12219433 40593738

[63] Zeng, T. et al. Cartilaginous extracellular matrix enriched with human gingival mesenchymal stem cells derived “Matrix bound extracellular vesicles” enabled functional reconstruction of tracheal defect. *Adv. Sci. (Weinh.)* **9**, e2102735 10.1002/advs.202102735 (2022). PMC8805569 34841733

[64] Wei, J. et al. TGF-β2 enhances glycolysis in chondrocytes via TβRI/p-Smad3 signaling pathway. *Biochimica et Biophysica Acta (BBA) - Molecular Cell Research* **1871** 10.1016/j.bbamcr.2024.119788 (2024). 38879132

[65] Tan, C., Li, L., Han, J., Xu, K. & Liu, X. A new strategy for osteoarthritis therapy: Inhibition of glycolysis. *Front. Pharmacol.* **13** 10.3389/fphar.2022.1057229 (2022). PMC9685317 36438808

[66] Hu, N. et al. Hypoxia-pretreated ADSC-derived exosome-embedded hydrogels promote angiogenesis and accelerate diabetic wound healing. *Acta Biomaterialia* **157**, 175–186 10.1016/j.actbio.2022.11.057 (2023). 36503078

[67] Liang, Y., Wei, S. & Zhang, A. Bioengineered tracheal graft with enhanced vascularization and mechanical stability for functional airway reconstruction. *Regenerative Ther.* **29**, 364–380 10.1016/j.reth.2025.03.016 (2025). PMC12005227 40248768

[68] Zopf, D. A., Flanagan, C. L., Wheeler, M., Hollister, S. J. & Green, G. E. Treatment of Severe Porcine Tracheomalacia With a 3-Dimensionally Printed, Bioresorbable, External Airway Splint. *JAMA Otolaryngol.–Head Neck Surg.* **140** 10.1001/jamaoto.2013.5644 (2014). PMC4429780 24232078

