# Supplementary material for: 3D minichannel-morphed adipose stem cell-cartilage granule reconstituted tough grafts for repairing segmental tracheal defects in preclinical models
Source: Nat Commun. 2026 Sep 11;17:10735. doi: 10.1038/s41467-026-77738-z (PMC13658815; doi:10.1038/s41467-026-77738-z)
Supplement: Supplementary file 1 [file 41467_2026_77738_MOESM10_ESM.pdf]

nature portfolio

Corresponding author(s): Wei Wu

Last updated by author(s): Aug 08, 2026

Reporting Summary

Nature Portfolio wishes to improve the reproducibility of the work that we publish. This form provides structure for consistency and transparency in reporting. For further information on Nature Portfolio policies, see our Editorial Policies and the Editorial Policy Checklist.

Statistics

For all statistical analyses, confirm that the following items are present in the figure legend, table legend, main text, or Methods section.

n/a	Confirmed	
☐	☒	The exact sample size (*n*) for each experimental group/condition, given as a discrete number and unit of measurement
☐	☒	A statement on whether measurements were taken from distinct samples or whether the same sample was measured repeatedly
☐	☒	The statistical test(s) used AND whether they are one- or two-sided<br>*Only common tests should be described solely by name; describe more complex techniques in the Methods section.*
☒	☐	A description of all covariates tested
☐	☒	A description of any assumptions or corrections, such as tests of normality and adjustment for multiple comparisons
☐	☒	A full description of the statistical parameters including central tendency (e.g. means) or other basic estimates (e.g. regression coefficient) AND variation (e.g. standard deviation) or associated estimates of uncertainty (e.g. confidence intervals)
☐	☒	For null hypothesis testing, the test statistic (e.g. *F*, *t*, *r*) with confidence intervals, effect sizes, degrees of freedom and *P* value noted<br>*Give P values as exact values whenever suitable.*
☒	☐	For Bayesian analysis, information on the choice of priors and Markov chain Monte Carlo settings
☒	☐	For hierarchical and complex designs, identification of the appropriate level for tests and full reporting of outcomes
☒	☐	Estimates of effect sizes (e.g. Cohen's *d*, Pearson's *r*), indicating how they were calculated

Our web collection on statistics for biologists contains articles on many of the points above.

Software and code

Policy information about availability of computer code

Data collection

Scanning Electron Microscopy (SEM): Field-emission scanning electron microscope (S-4800, Hitachi, Tokyo, Japan).
Fourier Transform Infrared Spectroscopy (FTIR): FTIR spectrometer (Nicolet iS 5, Thermo Fisher Scientific, MA, USA).
Rheological Properties: Parallel-plate rheometer (MCR 102e, Anton Paar, Graz, Austria).
Micro-Computed Tomography (Micro-CT): Nano-CT (Zeiss Xradia 520 versa, Oberkochen, Germany) and micro-CT (AX3000-D, Always Imaging, Shanghai, China).
Mechanical Testing: Universal testing machine (UTM5105 / ElectroForce 3200, Sansi Zongheng / Bose, China/USA).
Fluorescence Microscopy: Inverted fluorescence microscope (IX71, Olympus, Tokyo, Japan).
Proteomic Analysis: Label-free LC–MS/MS on NanoElute UHPLC coupled with a timsTOF Pro 2 mass spectrometer (Bruker Daltonics, Billerica, MA, USA) in dia-PASEF mode.
No custom code or algorithm was used or generated in this study.

Data analysis

Image Analysis: ImageJ software (v1.48v / v1.53, National Institutes of Health, Bethesda, MD, USA) was used for quantitative analysis of histological, fluorescence, and SEM images.
Statistical Analysis: GraphPad Prism 9.0 (GraphPad Software, La Jolla, CA, USA) was used for statistical analyses and graph generation.
CT Reconstruction and Analysis: CTAn and VG Studio MAX 3.5 (Volume Graphics, Heidelberg, Germany) were used for 3D reconstruction and pore interconnectivity analysis.
Proteomic Data Processing: DIA-NN search engine (v1.8) was used for processing raw DIA mass spectrometry data and protein identification.
Western Blot Analysis: ImageJ software was used for quantification of western blot band intensities.

For manuscripts utilizing custom algorithms or software that are central to the research but not yet described in published literature, software must be made available to editors and reviewers. We strongly encourage code deposition in a community repository (e.g. GitHub). See the Nature Portfolio guidelines for submitting code & software for further information.

Data

Policy information about availability of data

All manuscripts must include a data availability statement. This statement should provide the following information, where applicable:

- Accession codes, unique identifiers, or web links for publicly available datasets
- A description of any restrictions on data availability
- For clinical datasets or third party data, please ensure that the statement adheres to our policy

The mass spectrometry proteomics data generated in this study have been deposited in the PRIDE database under accession code PXD067839 (https://proteomecentral.proteomexchange.org/ui?pxid=PXD067839). Source data for all graphs, charts, and original uncropped scans in Figures and Supplementary Figures are provided as Source Data files alongside this paper. All other data supporting the findings of this study are available within the article and its Supplementary Information.

Research involving human participants, their data, or biological material

Policy information about studies with human participants or human data. See also policy information about sex, gender (identity/presentation), and sexual orientation and race, ethnicity and racism.

Reporting on sex and gender	This work does not involve human research
Reporting on race, ethnicity, or other socially relevant groupings	This work does not involve human research
Population characteristics	This work does not involve human research
Recruitment	This work does not involve human research
Ethics oversight	This work does not involve human research

Note that full information on the approval of the study protocol must also be provided in the manuscript.

Field-specific reporting

Please select the one below that is the best fit for your research. If you are not sure, read the appropriate sections before making your selection.

☒ Life sciences ☐ Behavioural & social sciences ☐ Ecological, evolutionary & environmental sciences

For a reference copy of the document with all sections, see nature.com/documents/nr-reporting-summary-flat.pdf

Life sciences study design

All studies must disclose on these points even when the disclosure is negative.

Sample size	No statistical method was used to predetermine sample size. Sample sizes were chosen based on standard practices in preclinical tissue engineering studies and prior experimental experience. A minimum of n = 3 independent biological samples or experiments were performed for in vitro characterizations. For animal studies, sample sizes (n = 18 rabbits [CGP, n = 6, CGPA, n = 12], n = 8 Bama minipigs, n = 9 BALB/c nude mice [n = 3 per group]) were selected to ensure adequate statistical power for evaluating mechanical stenting, vascularization, and survival outcomes.
Data exclusions	No data were excluded from the analyses. All collected experimental data were included in the statistical evaluations.
Replication	All key experiments were repeated independently at least three times with similar results. All attempts at replication were successful, and data consistency was confirmed across independent biological replicates.
Randomization	Animals and experimental samples were randomly assigned to intervention groups at the time of study initiation to minimize potential bias.
Blinding	For all experiments in this study, investigators were strictly blinded to group allocation during data collection and analysis.

Reporting for specific materials, systems and methods

We require information from authors about some types of materials, experimental systems and methods used in many studies. Here, indicate whether each material, system or method listed is relevant to your study. If you are not sure if a list item applies to your research, read the appropriate section before selecting a response.

Materials & experimental systems

n/a	Involved in the study
☐	☒ Antibodies
☒	☐ Eukaryotic cell lines
☒	☐ Palaeontology and archaeology
☐	☒ Animals and other organisms
☒	☐ Clinical data
☒	☐ Dual use research of concern
☒	☐ Plants

Methods

n/a	Involved in the study
☒	☐ ChIP-seq
☒	☐ Flow cytometry
☒	☐ MRI-based neuroimaging

Antibodies

Antibodies used

Collagen II (WB, IHC, 1:1000, Cat. #28459-1-AP, Proteintech, China);
SOX9 (WB, 1:1000, Cat. #67439-1-Ig, Proteintech, China);
β-Actin (WB, 1:1000, Cat. #20536-1-AP, Proteintech, China);
CD31 (IF, 1:200, Cat. #NB600-562, Novus, USA);
IL-1β (IF, 1:300, Cat. #66737-1-Ig, Proteintech, China);
Ac-Tubulin (IF, 1:300, Cat. #66200-1-Ig, Proteintech, China);
TNF-α (IF, 1:400, Cat. #26405-1-AP, Proteintech, China);
Cytokeratin (CK; IF, ready-to-use, Cat. #ab961, Abcam, UK);
Hexokinase 2 (HK2; IF, 1:200, Cat. #arp54303, AvivaSysBio, USA).

Validation

All primary antibodies were obtained from commercial suppliers and validated by the manufacturers for reactivity in the specified species (rabbit, pig, mouse, human) and application types (WB, IHC, or IF). Antibody specificity was further confirmed by positive/negative control tissue staining and expected molecular weight verification in western blotting.

Animals and other research organisms

Policy information about studies involving animals; ARRIVE guidelines recommended for reporting animal research, and Sex and Gender in Research

Laboratory animals

Male New Zealand White rabbits (6 months old, 3.5–4.5 kg), male Bama minipigs (4–5 months old, 12–15 kg,), and male BALB/c nude mice (6–8 weeks old, 18–22 g) were purchased from Chengdu Dossy Experimental Animals Co., Ltd. All animals were housed in individual cages under controlled conditions (22 ± 2 °C, 55 ± 10% humidity, 12-h light/dark cycle) with free access to standard food and water.

Wild animals

The study did not involve wild animals.

Reporting on sex

Male animals were used across all in vivo models. Sex was not considered an independent variable in the experimental design or analysis, as the primary biological mechanisms under investigation (scaffold mechanical stenting, vascular fascial integration, and chondrocyte glycolytic adaptation) are independent of sex differences.

Field-collected samples

The study did not involve samples collected from the field.

Ethics oversight

All animal research protocols complied with all relevant ethical regulations for animal testing and were reported in accordance with the ARRIVE guidelines. All experimental protocols were reviewed and approved by the Laboratory Animal Ethics Committee of the Fourth Military Medical University (Approval No. 2023kq040).

Note that full information on the approval of the study protocol must also be provided in the manuscript.

Plants

Seed stocks

This work does not involve plants.

Novel plant genotypes

This work does not involve plants.

Authentication

This work does not involve plants.
