# Supplementary material for: 3D minichannel-morphed adipose stem cell-cartilage granule reconstituted tough grafts for repairing segmental tracheal defects in preclinical models
Source: Nat Commun. 2026 Sep 11;17:10735. doi: 10.1038/s41467-026-77738-z (PMC13658815; doi:10.1038/s41467-026-77738-z)
Supplement: Supplementary file 2 [file 41467_2026_77738_MOESM11_ESM.pdf]

nature portfolio

Peer Review File

3D minichannels morphed adipose stem cell-cartilage granule reconstituted tough grafts for repairing segmental tracheal defects

Corresponding Author: Professor Wei Wu

This file contains all reviewer reports in order by version, followed by all author rebuttals in order by version.

Attachments originally included by the reviewers as part of their assessment can be found at the end of this file.

Version 0:

Reviewer comments:

Reviewer #1

(Remarks to the Author)
Overall
A significant complication noted in many animal studies of tissue engineered tracheal segmental replacement is granulation tissue and severe stenosis. In the current paper, the authors do not discuss granulation tissue or stenosis in their animal models. However, in the representative retrieved porcine graft, it does appear grossly as though narrowing and stenosis did occur (Fig. 8d, 8 week pig graft).

The authors should comment on the presence/degree of granulation tissue and stenosis, as this is widely noted in many animal models, across a range of scaffolding materials1-3. Thus authors should show the results from all six pigs (gross pictures of the lumen opening) at least in the supplemental data into which the grafts were implanted.

Although the authors note that the use of minced cartilage has been not widely applied in tracheal tissue engineering, it has been widely applied in repair of articular cartilage defects for a number of years4-7. Furthermore, it the use of minced cartilage seeded with hydrogels in 3D printed scaffolds with through penetration through the structures has been demonstrated8. The authors should note this in the discussion.

It would be useful to have a clinical measures of ease or difficulty in respiration for the experimental animals as another measure of the success of the graft. This could include the Wesley-Croup score.

An important note the authors make is the reliance of epithelialization of the graft from the host being a limitation in lines 607-609 of the manuscript. However, the authors don’t cite nor discuss previous work in which epithelial cells were implanted with tissue engineered grafts9, 10. The authors should discuss how their approach could be modified if possible to deliver epithelial cells.

Specific Critiques:
Lines 156-157: There is not data on the number of cycles or loading rate for the mechanical test data. Also, the duration of 130seconds and what looks like 12-15 cycles (Fig. 2h) cannot qualify as a fatigue test, which would typically incur tens of thousands to millions of load cycles. This figure could be removed from the paper.

Lines 241-241 (Figure 3O). The use of bone mineral density to describe the filling of the scaffolds does not provide significant insight without a phantom and potentially the use of contrast. For example, it would be useful to have a contrast enhanced image to show how the minced cartilage is distributed in the scaffold mini-channels. Specifically having one image of empty channels, one with PRP alone and one with PRP plus the minced cartilage in Fig. 3 would be useful for readers, rather than only a generic bone mineral density number that could result from a number of factors.

1. Bhora FY, Lewis EE, Rehmani SS, Ayub A, Raad W, Al-Ayoubi AM, Lebovics RS. Circumferential Three-Dimensional–Printed Tracheal Grafts: Research Model Feasibility and Early Results. The Annals of Thoracic Surgery. 2017;104(3):958-

63. doi: https://doi.org/10.1016/j.athoracsur.2017.03.064.
2. Weber J, Martins RS, Muslim Z, Baig MZ, Poulikidis K, Al Shetawi AH, Bhora FY. Anastomotic stenosis of bioengineered trachea grafts is driven by transforming growth factor β1-induced signaling, proinflammatory macrophages, and delayed epithelialization. JTCVS Open. 2023;15:489-96. doi: 10.1016/j.xjon.2023.07.016.
3. Weber JF, Rehmani SS, Baig MZ, Jadoon Y, Bhora FY. Successes and Failures in Tracheal Bioengineering: Lessons Learned. The Annals of Thoracic Surgery. 2021;112(4):1089-94. doi: 10.1016/j.athoracsur.2020.10.021.
4. Marmotti A, Bruzzone M, Bonasia DE, Castoldi F, Von Degerfeld MM, Bignardi C, Mattia S, Maiello A, Rossi R, Peretti GM. Autologous cartilage fragments in a composite scaffold for one stage osteochondral repair in a goat model. Eur Cell Mater. 2013;26:15-31; discussion -2. Epub 2013/08/06. doi: 10.22203/ecm.v026a02. PubMed PMID: 23913344.
5. Olesen ML, Christensen BB, Foldager CB, Hede KC, Jorgensen NL, Lind M. No Effect of Platelet-Rich Plasma Injections as an Adjuvant to Autologous Cartilage Chips Implantation for the Treatment of Chondral Defects. Cartilage. 2021;13(2_suppl):277S-84S. Epub 2019/07/23. doi: 10.1177/1947603519865318. PubMed PMID: 31327251; PMCID: PMC8804778.
6. Salzmann GM, Niemeyer P, Hochrein A, Stoddart MJ, Angele P. Articular Cartilage Repair of the Knee in Children and Adolescents. Orthop J Sports Med. 2018;6(3):2325967118760190. Epub 2018/03/24. doi: 10.1177/2325967118760190. PubMed PMID: 29568785; PMCID: PMC5858627.
7. Salzmann GM, Ossendorff R, Gilat R, Cole BJ. Autologous Minced Cartilage Implantation for Treatment of Chondral and Osteochondral Lesions in the Knee Joint: An Overview. Cartilage. 2021;13(1_suppl):1124S-36S. Epub 2020/07/28. doi: 10.1177/1947603520942952. PubMed PMID: 32715735; PMCID: PMC8808955.
8. Brown NE, Ellerbe LR, Hollister SJ, Temenoff JS. Development and Characterization of Heparin-Containing Hydrogel/3D-Printed Scaffold Composites for Craniofacial Reconstruction. Ann Biomed Eng. 2024;52(8):2287-307. Epub 20240511. doi: 10.1007/s10439-024-03530-z. PubMed PMID: 38734845.
9. Kim IG, Park SA, Lee S-H, Choi JS, Cho H, Lee SJ, Kwon Y-W, Kwon SK. Transplantation of a 3D-printed tracheal graft combined with iPS cell-derived MSCs and chondrocytes. Scientific Reports. 2020;10(1):4326. doi: 10.1038/s41598-020-61405-4.
10. Park JH, Ahn M, Park SH, Kim H, Bae M, Park W, Hollister SJ, Kim SW, Cho DW. 3D bioprinting of a trachea-mimetic cellular construct of a clinically relevant size. Biomaterials. 2021;279:121246. Epub 2021/11/15. doi: 10.1016/j.biomaterials.2021.121246. PubMed PMID: 34775331; PMCID: PMC8663475.

Reviewer #2

(Remarks to the Author)
This is an excellent and important paper. The ideas are innovative and can be broadly applied.

I do not see definitive evidence that it was the lack of respiratory epithelium that was the cause of the deaths in the animal model particularly with only a relatively short segmental defect. Video bronchoscopy (especially with studies looking at mobility), cultures and full necropsy results would enable a more robust conclusion.

The paper can readily be remediated by noting the limitations in ascribing lack of respiratory epithlium as the cause of death.

Reviewer #3

(Remarks to the Author)
General Comment
This manuscript presents a compelling example of integrating traditional scaffold materials, hydrogels, and regenerative medicine components—specifically, a cartilage granule–PRP–ADSC composite—for tracheal reconstruction. The approach is innovative, and the data generated in both small and large animal models are encouraging. However, several methodological, interpretative, and clarity-related issues should be addressed to enhance the scientific rigour and translational relevance of the study.

Specific Comments
Introduction
1. Paediatric Growth Compatibility
The scaffold does not appear to support growth, which may limit its suitability for congenital cases. Please clarify whether the design targets adults only and consider addressing this in the Discussion or Limitations.

Results
2.2 Rheology and Structural Properties
2. PRP Mixture and Viscosity
The 5% PRP group is described as having "low viscosity," yet extrusion jams were observed. Low viscosity typically facilitates flow; the issue may instead relate to poor dispersion or granule aggregation. Please clarify and, if available, support with rheological data.
3. Interpretation of BMD Values
BMD is typically applied to mineralised constructs. Since the scaffold lacks hydroxyapatite, the increased value may simply reflect radiodensity. Please explain its relevance or consider alternate terminology.

2.3 Capillary Infiltration

4. Thickness vs. Porosity
Rather than thickness alone, reduced porosity likely limits capillary ingrowth. Please clarify if pore interconnectivity was evaluated and considered a contributing factor.

2.4 Rabbit Model
5. Typographical Error
Please confirm whether "CGP group was superior to CGP and native trachea" should read "CGPA group."
6. Excess GAG and pH Effects
Higher GAG levels may indicate chondrogenesis but could acidify the microenvironment. Were any pH-related effects observed, or could this be discussed as a potential limitation?

2.6 Pig Model
7. Endpoint Selection
Adverse events appeared by week 6, yet the study endpoint was set at week 8. Please clarify whether this was predefined or adjusted based on animal condition.
8. Epithelial Migration Evidence
Both Rabbit and Pig experiments encountered adverse events relating to sputum retention. Was there any indication of epithelial migration onto the scaffold surface, particularly with the fascia pre-coverage? If so, was this ciliated or squamous? If epithelialisation did not occur or occurred and was non-ciliated, then such retention would be expected.

Materials and Methods
9. PCL Longevity and Scaffold Role
Please specify the scaffold’s intended functional duration and discuss whether inflammation might compromise PCL integrity. Has CGPA been tested without the PCL framework?
10. Reagent-Grade PCL
Was the reagent-grade PCL purified or endotoxin-tested prior to in vivo use?
11. Residual HFIP
Has residual solvent been assessed post-fabrication? Data (e.g., FTIR, TGA) would help confirm safety.
12. Cryo-Grinding Protocol
Were cryoprotectants used during cartilage grinding? Including this detail would aid reproducibility.
13. GMP/Quality Standards
Were any GMP or equivalent standards applied to fabrication or sterilization? This would speed regulatory approvals for clinical trials.

Supplementary
14. CCK-8 Volume Discrepancy
Using 1 mL in a 96-well plate seems inconsistent with well capacity. Please clarify.
15. Adverse Events Interpretation
Since epithelial regeneration was limited, could the complications also involve scaffold inflammation or remodelling issues? The latter is a major issue with previous attempts at tracheal scaffold production (e.g. Eur Respir J. 2020 Jun 18;55(6):1901200). Clarifying this would improve interpretation.
16. Inflammatory Markers
Were cytokines such as IL-1β, TNF-α, or IL-6 assessed to evaluate immune response? This could help differentiate structural from immunological failure.

Reviewer #4

(Remarks to the Author)
I co-reviewed this manuscript with one of the reviewers who provided the listed reports. This is part of the Nature Communications initiative to facilitate training in peer review and to provide appropriate recognition for Early Career Researchers who co-review manuscripts.

Version 1:

Reviewer comments:

Reviewer #3

(Remarks to the Author)
I am happy with submitted changes.

Reviewer #4

(Remarks to the Author)
I co-reviewed this manuscript with one of the reviewers who provided the listed reports. This is part of the Nature Communications initiative to facilitate training in peer review and to provide appropriate recognition for Early Career Researchers who co-review manuscripts.

Reviewer #5

(Remarks to the Author)
This manuscript details the use of a novel tracheal construct for eventual use for a host of airway pathologies requiring replacement of multiple tracheal rings. There is an unmet clinical need for improving airway issues in patients with tracheomalacia, stenosis, and malignancies etc. The authors use a combination of 3-D printing along with adipose derived stem cells and cartilage chips to facilitate rapid cartilage formation. The preclinical studies included both rabbits and a porcine model for testing of this concept.
There remain a number of questions that should be addressed, and they are listed below:
1. The time points are rather short for this type of study. Even at 2 months you already are seeing failures so what is the effective lifespan of these constructs?
2. The epithelial layer is probably the most important component of the tracheal construct. The authors have done extensive optimization of many of the parameters for trachea but have completely ignored the most important aspect for success: ciliated epithelium to move fluid throughout the airway.
3. Why is angiogenesis important? cartilage formation is better with low oxygen tension. Is the vascular supply for the dorsal fibrous tissue covering? Please explain.
4. Material characterization of the tracheal constructs vs native trachea would have strengthened the paper.
5. Degradative half life is not defined for the polymers used in the studies. How might longer or shorter half-life affected the outcomes?
6. Clinical translation: how do the authors propose a strategy for eventual human use?
7. The authors use cartilage from the ear which is elastic cartilage while the tracheal rings are hyaline cartilage which is a different molecular species, why not use nasal septum instead which is hyaline
8. What is PGS or CGP, it is not identified anywhere that I could find, PCL is poly caprolactone.
9. What is the size of the cartilage granules?
10. Pg13 ln 331-333: How do ADSC's promote granule survival? are the granules viable? Not clear from the text. What about immunomodulatory mechanisms of action?
11. Histology results from figs 5 and 8 do not demonstrate good cartilage formation via Alcian blue staining. The cartilage formation is quite minimal and discontinuous when compared to native cartilage tissue.

Version 2:

Reviewer comments:

Reviewer #6

(Remarks to the Author)
The experimental study entitled "3D minichannels-morphed 'adipose stem cell–cartilage granule' reconstituted tough grafts for repairing segmental tracheal defects in preclinical animal models" by Zhang et al. was reviewed. This study represents an enormous amount of animal experimentation aimed at developing an autologous tissue-engineered graft for the repair of segmental tracheal defects. The novelty of this work lies in the use of a 3D minichannel scaffold incorporating adipose-derived stem cells, cartilage granules, and platelet-rich plasma. The engineered graft was first implanted into muscle for two weeks and subsequently used to repair tracheal defects in both rabbit and porcine models.
Comments and Questions
1. The authors demonstrated impressive short-term outcomes up to 8 weeks after tracheal implantation. The graft appeared sufficiently rigid and showed evidence of vascularization within two weeks. The authors should be congratulated for successfully completing these technically demanding experiments.
2. My major concern relates to outcomes beyond 8 weeks. In both rabbit and pig experiments, a considerable number of animals died after this time point. The manuscript would benefit from a clearer explanation of the causes of death. Were these deaths related to airway complications, graft failure, infection, or other systemic issues?
3. The macroscopic and endoscopic findings demonstrate a relatively dark appearance of the graft lumen, suggesting possible ischemic change, whereas the external surface appears more pink and well vascularized. Could the authors explain this discrepancy in color and clarify whether this finding reflects insufficient luminal perfusion or delayed mucosalization?
4. As discussed by the authors, the graft does not contain epithelium, which is essential for long-term airway clearance and function. Did the authors observe any migration or regeneration of native tracheal epithelium from the anastomotic edges into the graft during the follow-up period?

Version 3:

Reviewer comments:

Reviewer #2

(Remarks to the Author)
The authors present very interesting data that has the potential for use in replacing the trachea. The defects that require this will be long segment (short segment defects are amenable to resection with excellent expectation of results and interventions similar to those identified here should not be undertaken.
Many types of scaffolds will have an excellent appearance even for the first several months. It is the stability of the scaffolds

past one year of time that is key. The animals do not survive this time period. There is insufficient data to warrant use in humans at present outside of exceptional circumstances.

The limitations of the study and that this data should not be seen as a justification for human use at present and this should be explicitly and unequivocally stated in the text.

Reviewer #3

(Remarks to the Author)
The issue of epithelium is important. We found that free tissue engineered tracheal grafts do re-epithelialize but that this process can take many months depending on the length of graft. Most workers between 2010 and 2020 would stent such grafts, but this compromises epithelialization, so difficult line to tread. Adressing this more fully requires further revision.

Reviewer #6

(Remarks to the Author)
I appreciate the authors' responses to my comments and questions. I am satisfied with most of the revisions; however, I still have concerns regarding the explanation for the cause of late death.
Tracheal graft stenosis caused by secretion retention should be considered a consequence of graft failure. The authors state that tracheostomy and bronchoscopic interventions would eliminate this complication in human cases, but this remains speculative.

Version 4:

Reviewer comments:

Reviewer #6

(Remarks to the Author)
I am satisfied with the authors' responses to all of my comments and questions. Overall, this small-animal study demonstrates satisfactory short-term outcomes of the tracheal graft. However, the long-term results remain suboptimal because of insufficient epithelial regeneration.

Open Access This Peer Review File is licensed under a Creative Commons Attribution 4.0 International License, which permits use, sharing, adaptation, distribution and reproduction in any medium or format, as long as you give appropriate credit to the original author(s) and the source, provide a link to the Creative Commons license, and indicate if changes were made.
In cases where reviewers are anonymous, credit should be given to 'Anonymous Referee' and the source.
The images or other third party material in this Peer Review File are included in the article's Creative Commons license, unless indicated otherwise in a credit line to the material. If material is not included in the article's Creative Commons

license and your intended use is not permitted by statutory regulation or exceeds the permitted use, you will need to obtain permission directly from the copyright holder.
To view a copy of this license, visit https://creativecommons.org/licenses/by/4.0/

Replies to the reviewers' comments

Reviewer #1:

1. A significant complication noted in many animal studies of tissue engineered tracheal segmental replacement is granulation tissue and severe stenosis. In the current paper, the authors do not discuss granulation tissue or stenosis in their animal models. However, in the representative retrieved porcine graft, it does appear grossly as though narrowing and stenosis did occur (Fig. 8d, 8 week pig graft). The authors should comment on the presence/degree of granulation tissue and stenosis, as this is widely noted in many animal models, across a range of scaffolding materials1-3. Thus authors should show the results from all six pigs (gross pictures of the lumen opening) at least in the supplemental data into which the grafts were implanted.

Response: Thank you for the valuable suggestions. We sincerely agree with the reviewer for highlighting the issue of granulation-induced stenosis, which is indeed a common challenge in tissue-engineered tracheal replacement. In the revised manuscript, we have added gross lumen images of all six porcine grafts to the **Supplementary Materials (Figure S16)**, which are also described and discussed in **Section 2.6** and **Section** 3.

(Main text: Section 4.16)

4.16 Gross and quantitative analysis

Images of the tracheal opening lumens were acquired via a standardized imaging setup. The lumen area was measured via ImageJ software, and the percentage of the lumen opening area was calculated via the following formula:

$$\text{Opening area ratio of the graft lumen (\%)} = \frac{\text{Lumen Area after tracheal implantation}}{\text{Lumen Area before tracheal implantation}} \times 100$$

The percentage of stenosis was recorded for each sample.

(Supplementary Materials: Part II, Figure S16)

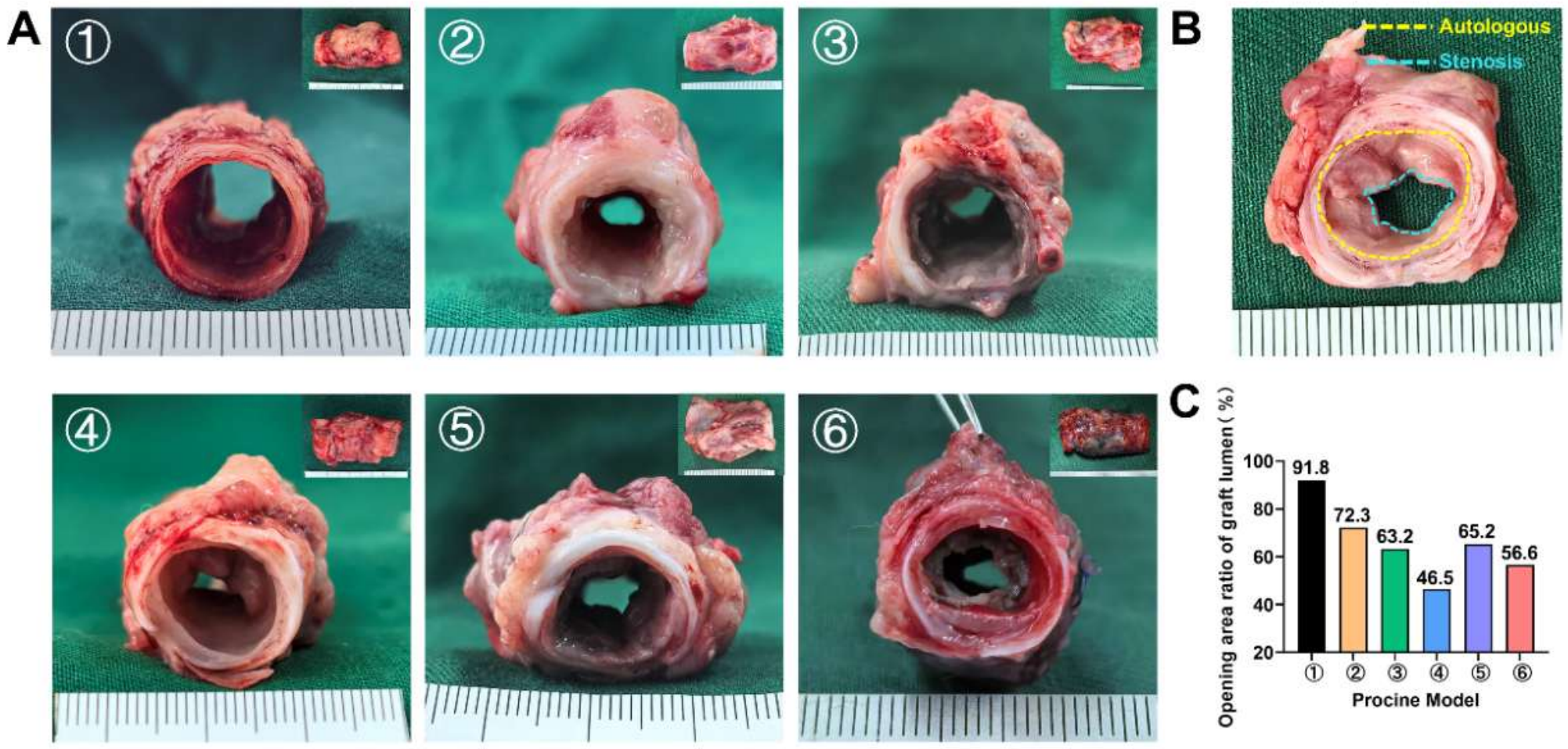

Figure S16. Gross analysis of explanted porcine tracheal grafts. (A) Lumen opening images of grafts harvested from six pigs. ① Tracheal graft harvested at 2 weeks, showing no significant granulation tissue or luminal stenosis in the graft. ② Tracheal graft harvested from a pig died of respiratory failure on day 43 due to progressive respiratory distress and sputum accumulation, indicating obvious tracheal stenosis. ③-⑥ Tracheal grafts harvested at 8 weeks, showing severe granulation hyperplasia and luminal stenosis. (B, C) Quantitative analysis of graft lumen stenosis. Percentage of the lumen opening area of the acquired tracheal grafts relative to the graft lumen area before tracheal implantation.

(Main text) Please refer to Para 3, Section 2.6 as follows:

Consistent with previous reports on tissue-engineered tracheal grafts *(Ann Thorac Surg. 2017 Sep;104(3):958-96334-36; Ann Thorac Surg. 2021 Oct;112(4):1089-1094; JTCVS Open. 2023 Jul 27:15:489-496)*, luminal stenosis was observed in some animals due to granulation tissue formation. Gross images of all the grafts (n = 6; Figure S16) revealed progressive stenosis at 2--8 weeks, with more pronounced narrowing of the grafts at 8 weeks. The integrity of the cartilaginous structure in all the grafts was maintained without collapse, confirming the mechanical resilience of the remodeled cartilage granules.

(Main text) Please refer to Para 5, Section 3 as follows:

Although "3D minichannels combined with cartilage granules" may achieve mechanical stability and graft integration, granulation-induced stenosis, a widely reported complication in tracheal bioengineering, has been observed in both rabbit and porcine grafts. The long-term survival limitation was attributed primarily to unresolved epithelial regeneration. Reliance solely on native

epithelial migration from anastomotic sites was insufficient for long-segment regeneration.

2. Although the authors note that the use of minced cartilage has been not widely applied in tracheal tissue engineering, it has been widely applied in repair of articular cartilage defects for a number of years4-7. Furthermore, it the use of minced cartilage seeded with hydrogels in 3D printed scaffolds with through penetration through the structures has been demonstrated 8. The authors should note this in the discussion.

Response: We sincerely appreciate your insightful suggestion. We acknowledge the established use of minced cartilage in articular cartilage repair and its integration with 3D-printed scaffolds. In the revised manuscript, we have integrated these points and supplemented them with relevant references in **Section 1** and **Section 3**.

(Main text) Please refer to Para 2, Section 1, as follows:

Inspired by the use of minced cartilage techniques in articular cartilage repair *(Orthop J Sports Med. 2018 Mar 13;6(3):2325967118760190; Eur Cell Mater. 2013 Aug 4:26:15-31; Cartilage. 2021 Dec;13(1_suppl):1124S-1136S; Ann Biomed Eng. 2024 Aug;52(8):2287-2307; Cartilage. 2021 Dec;13(2_suppl):277S-284S)*, we developed a mechanical processing technique to transform harvested cartilage tissues into granular cartilage. With this approach, the plasticity of minced cartilage is enhanced, which enables the repair of cartilaginous defects with unique anatomical morphologies and structures while avoiding the lengthy culture period for expanding chondrocytes.

(Main text) Please refer to Para 3, Section 3 as follows:

Although tissue engineering strategies utilizing "cell seeding scaffolds" have shown promise [8,11,39,40], the application of minced cartilage has gained significant acceptance in cartilage repair over the past decade. Autologous cartilage fragments have successfully regenerated osteochondral defects [17,18] in preclinical models, with established clinical protocols for knee lesions [16]. Recent advances have further demonstrated the integration of minced cartilage and hydrogels within 3D-printed scaffolds for craniofacial reconstruction [19]. However, tracheal reconstruction imposes distinct biomechanical and biological challenges: (1) Structural demands: Unlike articular surfaces, tracheal grafts require C-shaped rings that are resistant to circumferential compression from neck movement and breathing [6,7,9]. (2) Vascularization urgency: unlike other cartilaginous grafts, tracheal grafts are exposed

immediately to air once they are implanted, necessitating rapid fascial integration and graft vascularization to prevent infection.

3. It would be useful to have a clinical measure of ease or difficulty in respiration for the experimental animals as another measure of the success of the graft. This could include the Wesley-Croup score.

Response: Thank you for your valuable suggestions. We implemented Wesley-Croup scoring to assess respiratory function quantitatively in the rabbit and porcine models. In the revised manuscript, we have supplied **Figure 5C, Figure 8C** and **Supplementary Table S1**. The relevant descriptions have been added to **Sections 2.5 and 2.6**. These findings provide clinically relevant functional data supporting graft efficacy.

(Main text: Section 4.15.1)

4.15.1 Wesley-Croup scoring

Respiratory function was assessed via an adapted Wesley-Croup scale *(JAMA Otolaryngol Head Neck Surg. 2014 Jan;140(1):66-71.)* (Table S1). Evaluations were performed at 24-hour intervals by two blinded experimental animal researchers. A score ≥10 mandated immediate euthanasia per the animal ethics approval (2023kq040). Statistical analysis was performed via Mann–Whitney U tests (rabbits) and linear regression (pigs).

(Main text: Section 2.5, Figure 5C)

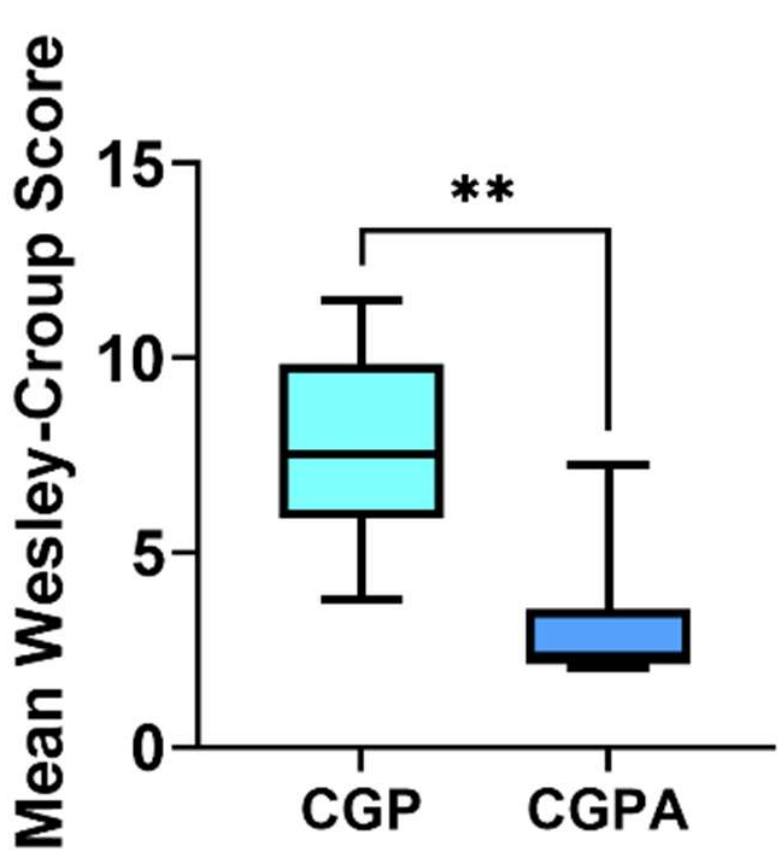

Figure 5C. Quantitative comparison of Wesley-Croup scores between the CGP and CGPA groups. Fig. 5C. Significance was determined by the Mann–Whitney U test. $^{**}p < 0.01$.

(Main text) Please refer to Para 2, Section 2.4 as follows:

Quantitative assessment via the Wesley-Croup scoring system (Suppl Table S1) revealed significantly better respiratory function in the CGPA group (3.03 ± 1.49) than in the CGP control group (7.70 ± 2.62, $p < 0.01$), indicating enhanced graft performance (Fig. 5C).

(Main text: Section 2.6, Figure 8C)

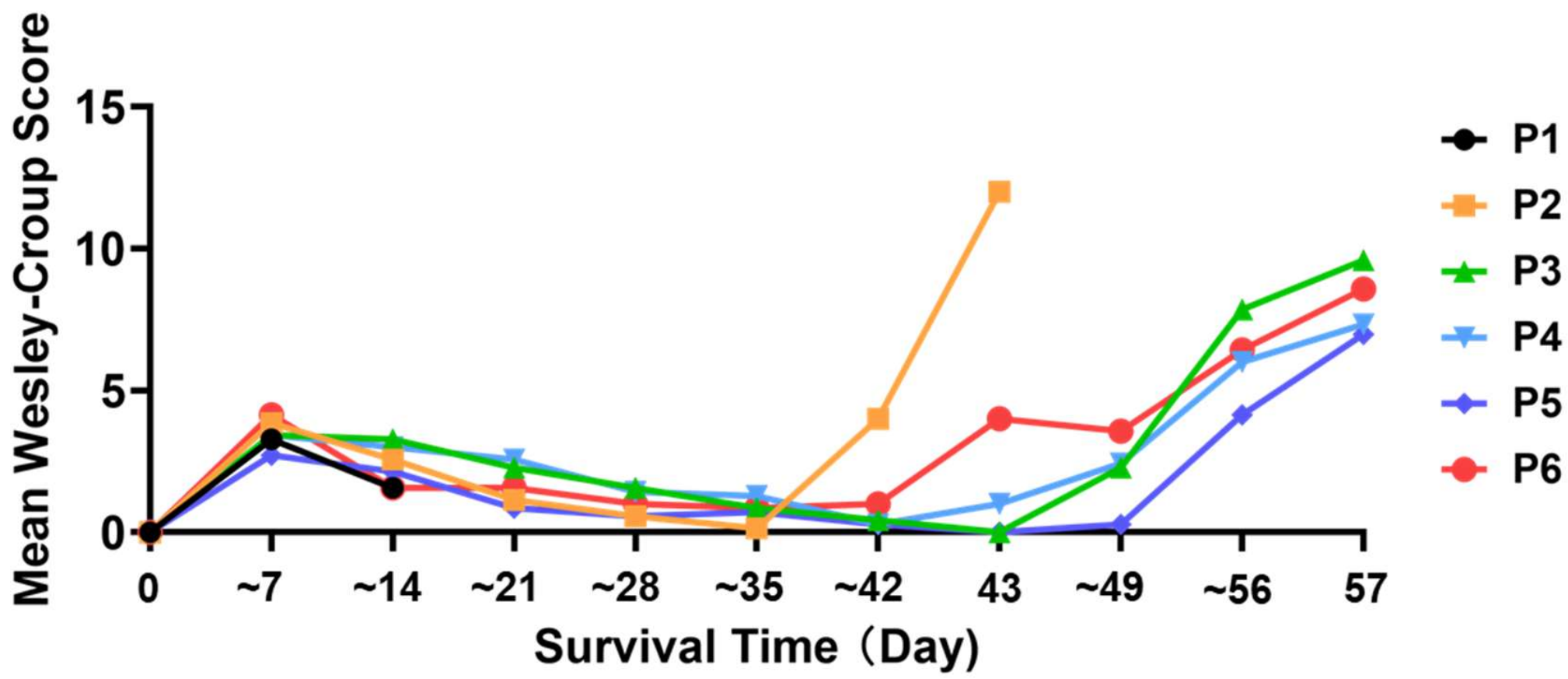

Figure 8C. Daily Wesley-Croup scores (n = 6 pigs) following segmental tracheal reconstruction.

(Main text) Please refer to Para 2, Section 2.6 as follows:

In this study, animal welfare was monitored daily via the Wesley-Croup scoring system. The animals whose score exceeded 10 were euthanized immediately for ethical reasons. One pig experienced severe respiratory dysfunction by postoperative day 43 (6 weeks), with a Wesley-Croup score of 12, and was subsequently euthanized (Fig. 8C). The remaining four animals, although exhibiting progressive respiratory symptoms by 8 weeks, did not exceed the endpoint threshold and were euthanized as planned at the predefined 8-week time point.

(Supplementary Materials: Part III, Supplementary Table S1)

Supplementary Table S1.

Wesley-Croup scoring system for animal models

Severity Classification:

Mild: ≤4 | Moderate: 5–6 | Severe: 7–11 | Respiratory failure: ≥12

Signs, Symptoms, and Indications	Score
Subcostal retractions	

None	0
Mild	1
Moderate	2
Severe	3
Stridor	
None	0
With agitation	1
At rest	2
Cyanosis	
None	0
With agitation	4
At rest	5
Level of Consciousness	
Normal	0
Disoriented	5
Air entry	
Normal	0
Decreased	1
Markedly decreased	2

4. An important note the authors make is the reliance of epithelialization of the graft from the host being a limitation in lines 607-609 of the manuscript. However, the authors don't cite nor discuss previous work in which epithelial cells were implanted with tissue engineered grafts9, 10. The authors should discuss how their approach could be modified if possible to deliver epithelial cells.

Response: Thank you for your insightful suggestions. We appreciate your comment regarding the limitation of host-dependent epithelialization in our tracheal grafts. To address this limitation, 3D minichannels can be integrated with existing epithelialization strategies in further studies. In the

revised manuscript, a relevant discussion has been added to **Section 3**.

(Main text) Please refer to Para 5, Section 3, as follows:

Current approaches include preseeding epithelial cells, iPSC-derived mesenchymal stem cells/chondrocytes (outer layer) and human bronchial epithelial cells (hBECs) (inner layer) onto tracheal grafts or fabricating 3D-bioprinted tracheal constructs with epithelial cells *(Eur Respir J. 2020 Jun 18;55(6):1901200; Biomaterials. 2021 Dec: 279:121246; Int J Mol Sci. 2021 Sep 25;22(19):10322; Sci Rep. 2020 Mar 9;10(1):4326)*. However, these strategies are clinically limited owing to the long culture period (2–6 weeks), high contamination risk, and restricted cell seeding efficiency and cell survival rate *(Eur Respir J. 2020 Jun 18;55(6):1901200)*. For epithelialization, our minichannel strategy may be modified via two approaches. First, during the prevascularization phase, the micrometer-scale mesh may be fabricated intraluminally via electrohydrodynamic (EHD) printing *(Sci Transl Med. 2023 Sep 20;15(714): eabo4272)* as an adhesive interface for efficiently seeding respiratory epithelial cells or mucosa sheets. Simultaneously, with vascularization, the luminal surface undergoes epithelial remodeling. Second, on the basis of our previous explorations of airway epithelial regeneration, cartilaginous ECM enriched with gingival extracellular vesicles (gEVs) could be bioengineered as a bioactive barrier against contamination to recruit epithelial progenitors via JAK2-STAT1 signaling *(Adv Sci (Weinh)). 2022 Jan;9(2): e2102735)*.

5. Lines 156-157: There is not data on the number of cycles or loading rate for the mechanical test data. Also, the duration of 130seconds and what looks like 12-15 cycles (Fig. 2I) cannot qualify as a fatigue test, which would typically incur tens of thousands to millions of load cycles. This figure could be removed from the paper.

Response: Thank you for identifying this limitation. We have removed the original **Figure 2I** and all related text describing "fatigue testing" in **Paragraph 1, Section 2.1**.

6. Lines 241-241 (Figure 3O). The use of bone mineral density to describe the filling of the scaffolds does not provide significant insight without a phantom and potentially the use of contrast. For example, it would be useful to have a contrast enhanced image to show how the minced cartilage is distributed in the scaffold mini-channels. Specifically having one image of

empty channels, one with PRP alone and one with PRP plus the minced cartilage in Fig. 3 would be useful for readers, rather than only a generic bone mineral density number that could result from a number of factors.

Response: Thank you for this scientific suggestion. We agree that "BMD" was inappropriate for nonmineralized scaffolds; we have replaced **Figure 3M** with enhanced micro-CT reconstruction images and removed the original **Figure 3N**. The revised **Figure 3M** more clearly illustrates the structure of 3D minichannels and the distribution of cartilage granules in the constructs. In the revised manuscript, we have removed the original descriptions and added new result descriptions in **Section 2.4**.

(Main text: Section 2.2, Figure 3M)

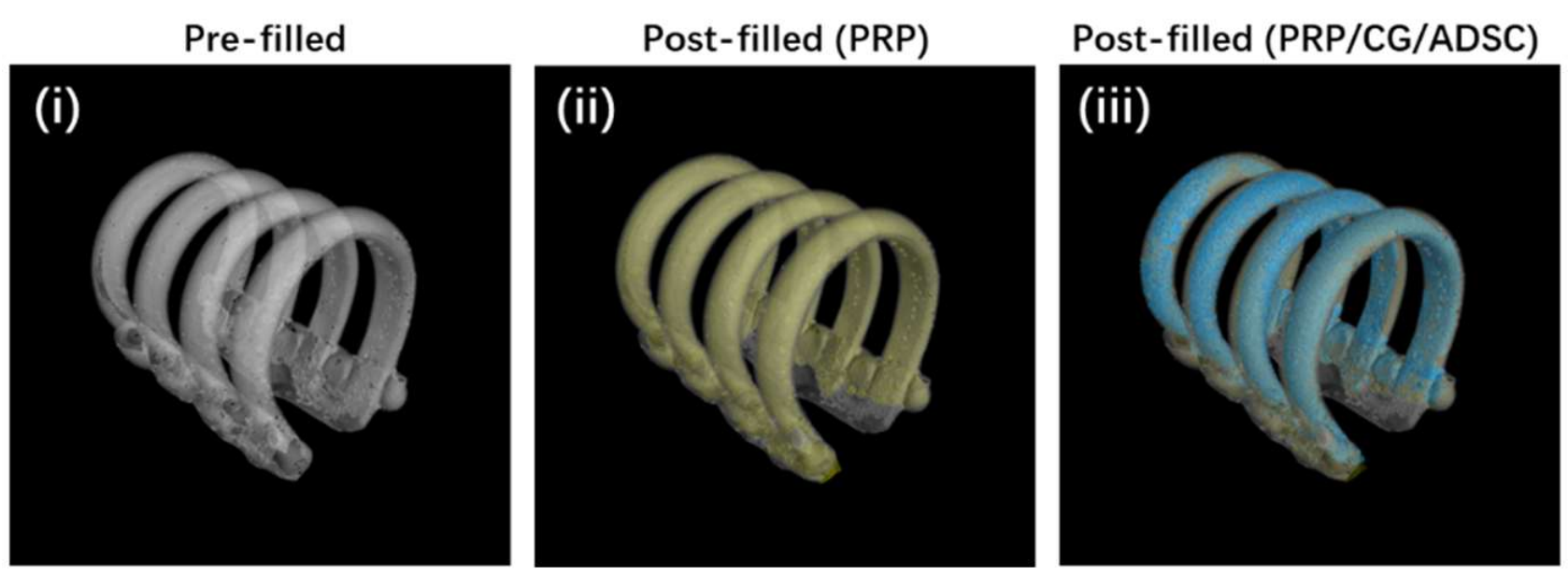

Figure 3M. 3D images showing cartilage granules filled with 3D minichannels were acquired via micro-CT scanning: (i) Hollow 3D minichannels, (ii) PRP-filled 3D minichannels, and (iii) PRP/CG/ADSC composite-filled 3D minichannels.

(Main text) Please refer to Para 4, Section 2.2 as follows:

To clarify the filling effect of injecting cartilage granules in 3D minichannels, three-dimensional reconstruction of the constructs was performed. As shown in Fig. 3M, the PGS-PCL hollow minichannel, PRP, and cartilage granule-stem cell composite were designated gray, yellow, and blue, respectively, on the basis of their densities. Fig. 3M (ii) shows that PRP uniformly filled the interior of the minichannels, whereas (iii) the blue cartilage granules mixed within PRP were also uniformly distributed inside the minichannels...

We have tried our best to improve the manuscript and made some changes in the manuscript. These changes did not influence the content or framework of the paper. Here, we did not list the changes but marked them in red in the revised manuscript.

We earnestly appreciate your warm work and hope that the corrections will be met with approval.

Once again, thank you very much for your comments and suggestions. Please free to inform me if you remain further concerned, we will revise them according to your further requests.

Reviewer #2: This is an excellent and important paper. The ideas are innovative and can be broadly applied.

Response: Thank you for your praise. We have provided point-by-point responses as follows, and we believe that this manuscript now meets the publication standard of *Nat Commun.*

1. I do not see definitive evidence that it was the lack of respiratory epithelium that was the cause of the deaths in the animal model particularly with only a relatively short segmental defect. Video bronchoscopy (especially with studies looking at mobility), cultures and full necropsy results would enable a more robust conclusion. The paper can readily be remediated by noting the limitations in ascribing lack of respiratory epithlium as the cause of death.

Response: Thank you for emphasizing the definitive causality between epithelial absence and mortality. We have provided three **bronchoscopy videos (Videos S7:** 13-Day CGP Group; **Video S8:** 65-Day CGPA Group; **Video S9:** 67-Day CGPA Group) from rabbits exhibiting severe respiratory distress symptoms, which included granulation causing airway stenosis and mucus obstruction. To further investigate the causes of death, we supplemented the experiment with immunofluorescence staining of the epithelium and inflammation in airway graft sections. The results have been added to the **Supplementary Materials (Figures S12 and S13)**. Additionally, we performed gross morphological analysis of all six pig grafts in the **Supplementary Materials (Figure S16)**, which revealed progressive luminal stenosis and corroborated the fatal respiratory failure caused by epithelial deficiency. The corresponding discussions have been integrated into **Sections 2.4 and 2.6** and **Section 3**.

(Main text) Please refer to Pars 2 and 3, Section 2.4, as follows:

In the CGP group, 50% of the rabbits did not survive beyond one week, with respiratory distress due to airway collapse being the primary cause of death (median survival: 8 days; Video S7, Table S1A). ... Despite the preserved circular structure, CGPA grafts acquired at longer time points (65 Days: Videos S8; 67 Days: Videos S9) exhibited stenosis and mobile mucus, indicating the essential role of the respiratory epithelium in long-term survival.

(Main text: Section 4.18)

4.18 Immunofluorescence Staining and Quantitative Analysis

The sections were dewaxed, rehydrated, and subjected to antigen retrieval in sodium citrate buffer. After blocking with 5% normal goat serum, the sections were incubated with primary antibodies against CD31 (NB600-562, Novus, USA, 1:200), IL-1β (67439-1-Ig, Proteintech, China, 1:300), Ac-Tubulin (66200-1-Ig, Proteintech, China, 1:300), TNF-α (26405-1-AP, Proteintech, China, 1:400), and CK (ab961, Abcam, UK, ready-to-use) overnight at 4 °C, followed by incubation with Alexa Fluor-conjugated secondary antibodies for 1 hour at room temperature. Nuclei were counterstained with DAPI (PC-90017, PCM, 1:1000). The fluorescence intensity was quantified via ImageJ software by measuring the mean intensity in three random fields per section (200x magnification), and the results are expressed as the means ± SDs.

(Supplementary Materials: Part II, Figure S12)

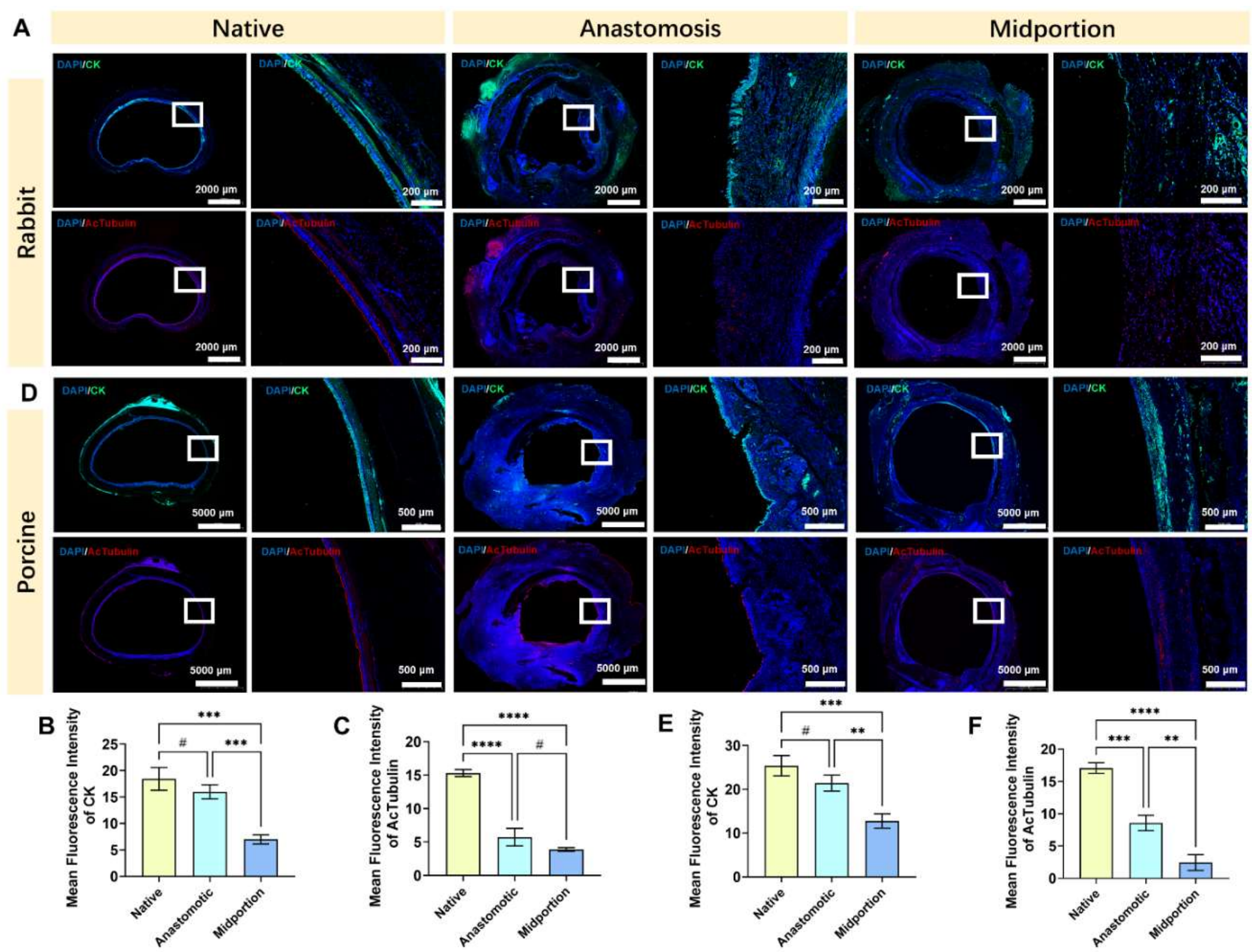

Figure S12. Epithelial marker expression in tracheal grafts. (A) Immunofluorescence of cytokeratin (CK, green) and AC-tubulin (red) in rabbit grafts: native trachea, graft anastomosis, and midportion (scale bars: 200 μm, 2000 μm). (B–C) Quantification of CK (B) and AC-tubulin (C) fluorescence intensity in rabbit grafts. (D) CK/AC-tubulin staining of pig grafts (scale bars: 500 μm, 5000 μm). (E–F) CK (E) and AC-tubulin (F) intensities in pig grafts. The significance of Fig. S12B,

C, E, and F was determined via one-way analysis of variance (ANOVA) followed by Tukey's post hoc analysis. $^{\#}p > 0.05$; $^{**}p < 0.01$, $^{***}p < 0.001$, $^{****}p < 0.0001$.

(Main text) Please refer to Para 4, Section 2.4, as follows:

To elucidate the mechanisms underlying the formation of inflammatory granulomas and the accumulation of mucus after tracheal transplantation in rabbits, epithelial and inflammation-related factors were stained. Immunofluorescence at 8 weeks revealed discontinuous cytokeratin-positive (CK+) cells at the graft–host anastomoses (Fig S12A), suggesting partial epithelial migration from the native tissue. However, no AC-tubulin+ ciliated epithelium was detected in the graft segments ($p < 0.001$ vs. native; Fig S12B, C). The absence of functional cilia, coupled with disorganized CK+ cells at anastomoses and negligible coverage in mid-grafts, explains persistent sputum retention despite fascial integration.

(Main text) Please refer to Para 3, Section 2.6 as follows:

Epithelial regeneration in the tracheal grafts of pigs was further investigated. Immunofluorescence staining revealed discontinuous CK+ cells near anastomoses (Fig. S12D, E). AC-tubulin staining revealed only sparse, noncontinuous ciliary structures in these regions (8.58 ± 1.18 vs native 17.07 ± 0.83; $p < 0.01$; Fig. S12F), and no ciliated epithelium could be detected in the mid-graft regions. Poor epithelialization, particularly the absence of functional ciliated epithelium, leads to progressive sputum accumulation and ultimately respiratory failure.

(Supplementary Materials: Part II, Figure S13)

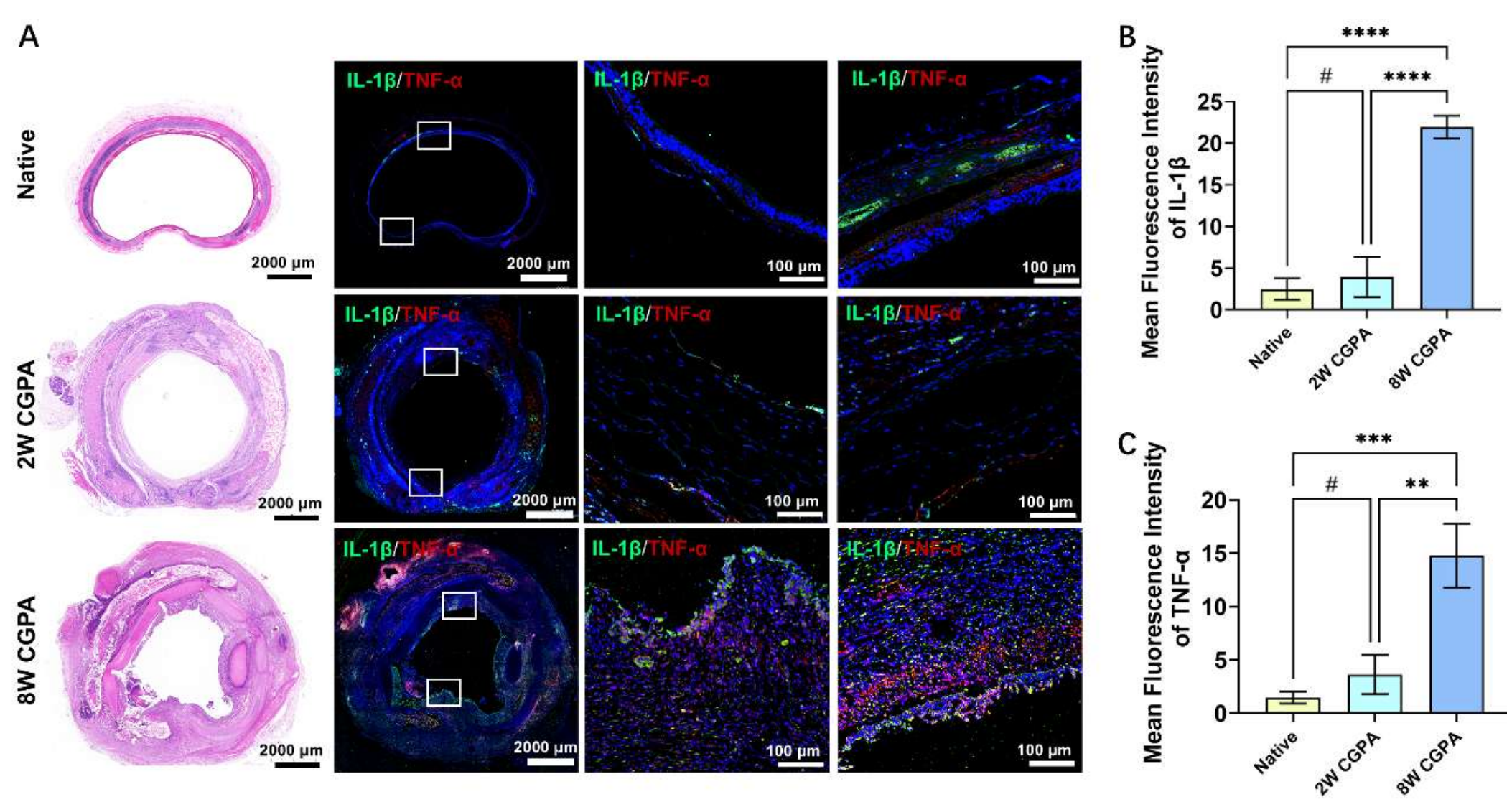

Figure S13. Inflammation marker expression in different tracheal grafts. (A) Immunofluorescence for IL-1β (green) and TNF-α (red) in grafts. Native tissue shows baseline signals (scale bars: 100 μm, 2000 μm). The 2 W CGPA grafts exhibited mild inflammation. 8 W CGPA grafts reveal intense IL-1β/TNF-α in granulation tissue. (B) Quantitative analysis of IL-1β intensity. (C) Quantitative analysis of TNF-α intensity. Fig. S13B, and C significance was determined via one-way analysis of variance (ANOVA) followed by Tukey's post hoc analysis. $^{\#}p > 0.05$; $^{**}p < 0.01$, $^{***}p < 0.001$, $^{****}p < 0.0001$.

(Main text) Please refer to Para 4, Section 2.4, as follows:

Notably, the inflammatory cascade is secondary to epithelial dysfunction. While early grafts (2 W) did not significantly increase the levels of cytokines (IL-1β: 3.93 ± 2.41; TNF-α: 3.65 ± 1.85; $p > 0.05$ vs. native), 8 W grafts exhibited dramatic increases in the levels of IL-1β (21.95 ± 1.38) and TNF-α (14.77 ± 2.99; $p < 0.01$), which are localized in luminal granulations (Fig. S13). These findings confirmed that inflammation arises from sputum retention, which is due to an insufficient epithelial barrier and prolonged exposure to the opening airway, rather than scaffold incompatibility.

(Main text) Please refer to Para 5, Section 3, as follows:

Although immunostaining (Fig. S12) confirmed discontinuous cytokeratin expression at anastomotic sites in both species, indicating host-derived epithelial migration, ciliated epithelium was scarce in grafts from both rabbits and pigs. Fascial precoverage facilitated graft vascularization but failed to augment epithelial regeneration. Consequently, the function of mucus clearance remains impaired, leading to sputum retention and inflammation-driven tracheal stenosis.

(Main text: Section 4.16)

4.16 Gross and quantitative analysis

Images of the tracheal opening lumens were acquired via a standardized imaging setup. The lumen area was measured via ImageJ software, and the percentage of the lumen opening area was calculated via the following formula:

$$\text{Opening area ratio of the graft lumen (\%)} = \frac{\text{Lumen Area after tracheal implantation}}{\text{Lumen Area before tracheal implantation}} \times 100$$

The percentage of stenosis was recorded for each sample.

(Supplementary Materials: Part II, Figure S16)

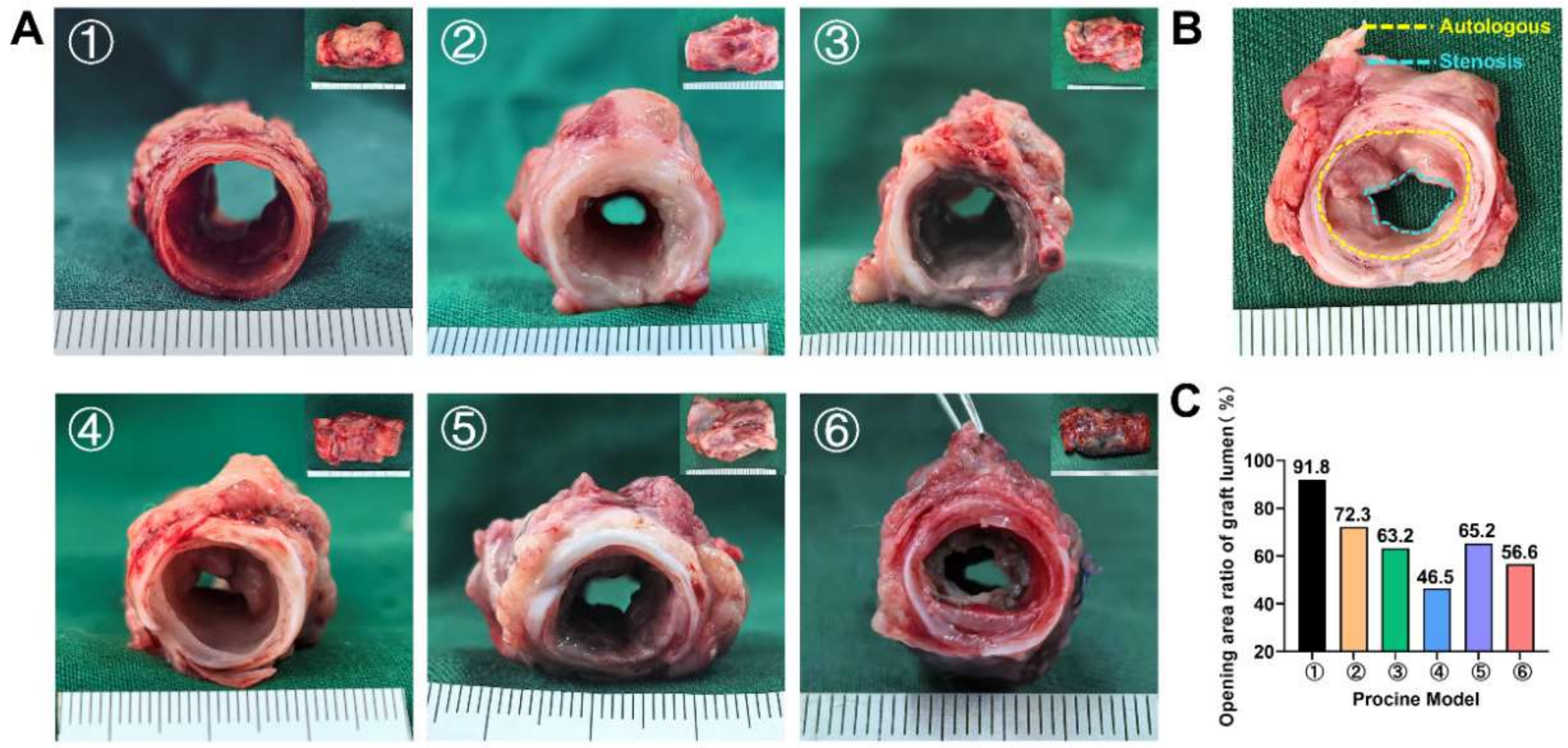

Figure S16. Gross analysis of explanted porcine tracheal grafts. (A) Lumen opening images of grafts harvested from six pigs. ① Tracheal graft harvested at 2 weeks, showing no significant granulation tissue or luminal stenosis in the graft. ② Tracheal graft harvested from a pig died of respiratory failure on day 43 due to progressive respiratory distress and sputum accumulation, indicating obvious tracheal stenosis. ③-⑥ Tracheal grafts harvested at 8 weeks, showing severe granulation hyperplasia and luminal stenosis. (B, C) Quantitative analysis of graft lumen stenosis. Percentage of the lumen opening area of the acquired tracheal grafts relative to the graft lumen area before tracheal implantation.

(Main text) Please refer to Para 3, Section 2.6 as follows:

Consistent with previous reports on tissue-engineered tracheal grafts *(Ann Thorac Surg. 2017 Sep;104(3):958-96334-36; Ann Thorac Surg. 2021 Oct;112(4):1089-1094; JTCVS Open. 2023 Jul 27:15:489-496)*, luminal stenosis was observed in some animals due to granulation tissue formation. Gross images of all the grafts (n = 6; Figure S16) revealed progressive stenosis at 2--8 weeks, with more pronounced narrowing of the grafts at 8 weeks. The integrity of the cartilaginous structure in all the grafts was maintained without collapse, confirming the mechanical resilience of the remodeled cartilage granules.

(Main text) Please refer to Para 5, Section 3 as follows:

Although "3D minichannels combined with cartilage granules" may achieve mechanical stability and graft integration, granulation-induced stenosis, a widely reported complication in tracheal bioengineering, has been observed in both rabbit and porcine grafts. The long-term survival limitation was attributed primarily to unresolved epithelial regeneration. Reliance solely on native

epithelial migration from anastomotic sites was insufficient for long-segment regeneration.

We have tried our best to improve the manuscript and made some changes in the manuscript. These changes did not influence the content or framework of the paper. Here, we did not list the changes but marked them in red in the revised manuscript.

We earnestly appreciate your warm work and hope that the corrections will be met with approval.

Once again, thank you very much for your comments and suggestions. Please free to inform me if you remain further concerned, we will revise them according to your further requests.

Reviewer #3: This manuscript presents a compelling example of integrating traditional scaffold materials, hydrogels, and regenerative medicine components-specifically, a cartilage granule-PRP-ADSC composite-for tracheal reconstruction. The approach is innovative, and the data generated in both small and large animal models are encouraging. However, several methodological, interpretative, and clarity-related issues should be addressed to enhance the scientific rigour and translational relevance of the study.

Response: Thank you for your positive feedback on the innovation and results of our study, as well as for your thorough evaluation. We have carefully considered all the comments provided and have made point-to-point revisions accordingly to enhance the scientific rigor and translational relevance of the manuscript. We believe that the manuscript now meets the publication standards of *Nat Commun* and hope that the changes satisfactorily address your concerns.

1. Paediatric Growth Compatibility: The scaffold does not appear to support growth, which may limit its suitability for congenital cases. Please clarify whether the design targets adults only and consider addressing this in the Discussion or Limitations.

Response: Thank you for this clinically critical suggestion. Our scaffold design primarily targets adult tracheal reconstruction, where growth adaptation is not needed. Nevertheless, we acknowledge the need for growth-compatible designs in pediatric applications, and we have added these considerations in **Section 3**.

(Main text) Please refer to Para 7, Section 3 as follows:

Although our graft shows potential for adult tracheal reconstruction, further research is needed to address its limited growth adaptability for application in congenital pediatric cases. The PCL-based scaffold slowly degrades *in vivo*, which may restrict radial and longitudinal expansion in growing airways. Rationalizing degrading and mechanical properties may improve the growth compatibility of tracheal grafts. Replacing partial PCL content with fast degrading materials such as PLA, PLGA, and PGS may match the growth potential of the host tissue *(Regen Ther. 2025 Apr 9:29:364-380)*. Additionally, integrating bioactive factors (e.g., TGF-β) into minichannel walls may stimulate endogenous cartilage growth, potentially enabling growth-compatible designs *(Adv Sci (Weinh)). 2024 Oct;11(39): e2405420)*.

2. PRP Mixture and Viscosity: The 5% PRP group is described as having "low viscosity," yet extrusion jams were observed. Low viscosity typically facilitates flow; the issue may instead relate to poor dispersion or granule aggregation. Please clarify and, if available, support with rheological data.

Response: Thank you for this scientific suggestion. The term 'low viscosity' was indeed inaccurate for describing the 5% PRP mixture. We have revised the description in **Section 2.2** and added rheological data to the **Supplementary Materials (Figure S9)** to clarify this mechanism.

(Supplementary Materials: Part I, Materials and Methods)

Rheological characterization of PRP-cartilage composites

The rheological properties of the PRP-cartilage granule mixtures (5% PRP, 10% PRP, and 20% PRP) were analyzed via a parallel-plate rheometer (Anton Paar-MCR 102e). Measured storage (G') and loss (G'') moduli at 1 Hz. Shear-rate sweep: 0.01–100 s. All tests were conducted at 25 °C (n = 3).

(Supplementary Materials: Part II, Figure S9)

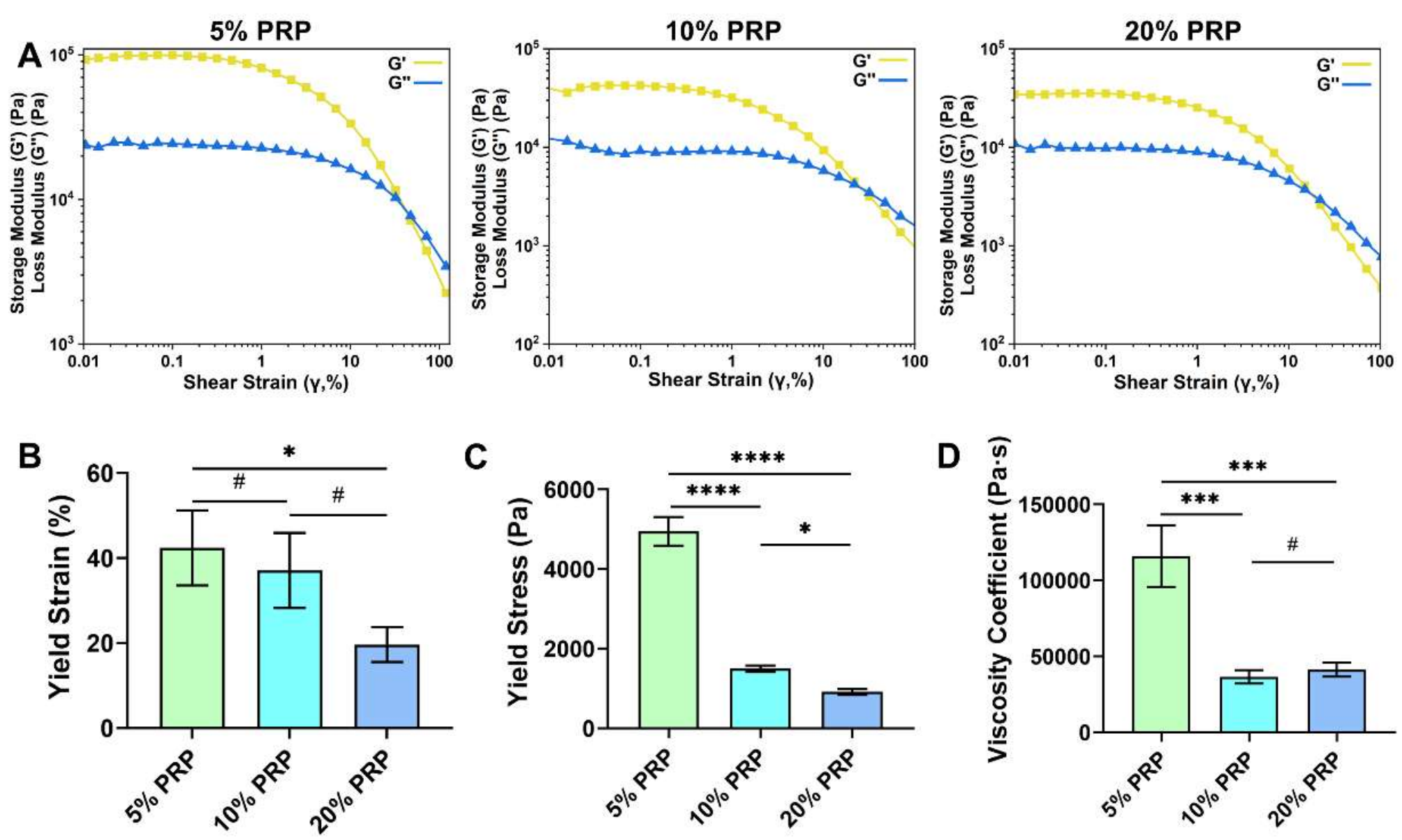

Figure S9. Rheological properties of PRP-cartilage granule mixtures. (A) Storage (G') and loss (G'') moduli vs. shear strain for hydrogels with 5%, 10%, and 20% PRP. (B) Yield strain, (C) yield stress, (D) viscosity coefficient across different PRP concentrations (n = 3). As shown in Fig S9B,

C, and D, significance was determined via one-way analysis of variance (ANOVA) followed by Tukey's post hoc analysis. $^{\#}p > 0.05$; $^{*}p < 0.05$, $^{***}p < 0.001$, $^{****}p < 0.0001$.

(Main text) Please refer to Para 3, Section 2.2 as follows:

Rheological analysis (Fig. S9) revealed that the 5% PRP mixture presented high zero-shear viscosity (115,953.33 ± 20216.14 Pa·s) and a high yield strain (42.40 ± 8.83%), indicating particle aggregation and poor dispersion. This led to discontinuous flow under shear stress, causing extrusion jams. In the 20% PRP group, the significant reduction in viscosity and yield stress led to high fluidity, which resulted in a final injection accuracy of only 43.67 ± 2.08%. Conversely, the 10% PRP mixture demonstrated optimal rheological properties with moderate viscosity (36,543.67 ± 4411.75 Pa·s) and low yield strain (37.10 ± 8.84%), resulting in smooth injectability and high precision (95.67 ± 2.08%). The 10% PRP ratio optimized fluidity by balancing granule dispersion and the lubricating effects of PRP, which prevented aggregation while maintaining structural cohesion postinjection.

3. Interpretation of BMD Values: BMD is typically applied to mineralised constructs. Since the scaffold lacks hydroxyapatite, the increased value may simply reflect radiodensity. Please explain its relevance or consider alternate terminology.

Response: Thank you for this scientific suggestion. We agree that "BMD" was inappropriate for nonmineralized scaffolds; we have replaced **Figure 3M** with enhanced micro-CT reconstruction images and removed Figure 3N. The revised **Figure 3M** more clearly illustrates the structure of the microchannel scaffold and the distribution of cartilage microparticles in the minichannels. In the revised manuscript, the corrected descriptions are presented in **Section 2.4**.

(Main text: Section 2.2, Figure 3M)

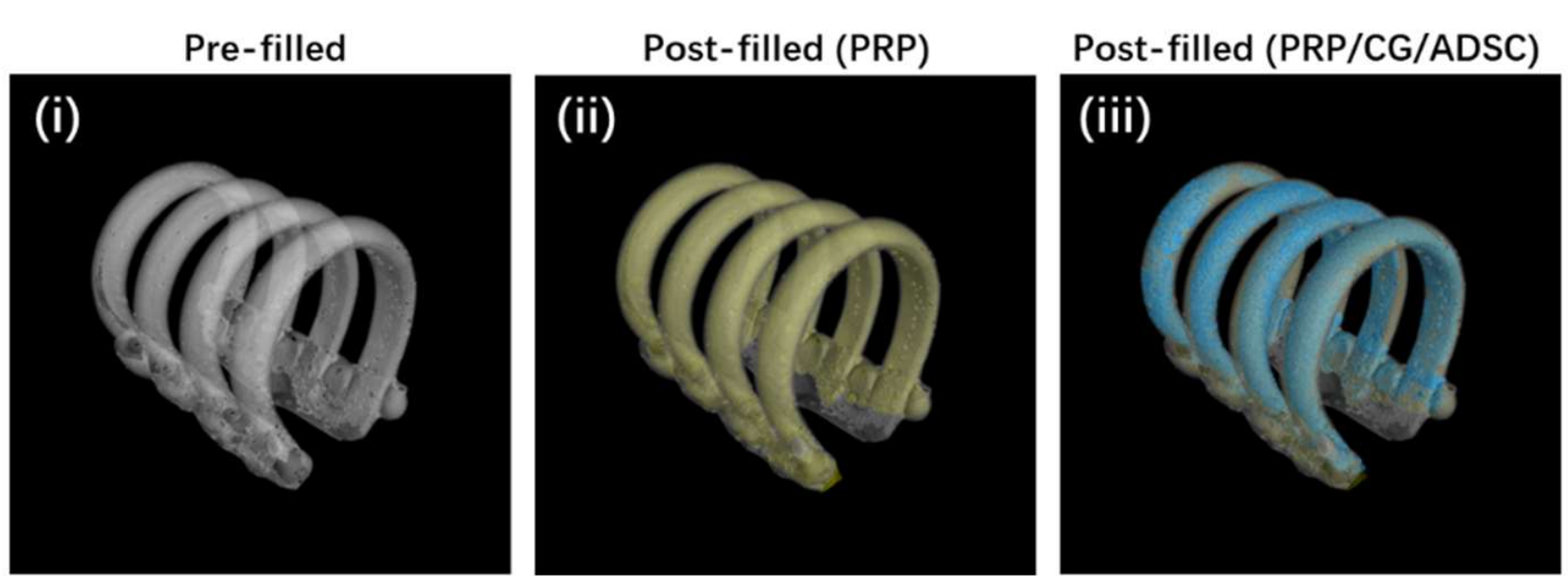

Figure 3M. 3D images showing cartilage granules filled with 3D minichannels were acquired

via micro-CT scanning: (i) Hollow 3D minichannels, (ii) PRP-filled 3D minichannels, and (iii) PRP/CG/ADSC composite-filled 3D minichannels.

(Main text) Please refer to Para 4, Section 2.2 as follows:

To clarify the filling effect of injecting cartilage granules in 3D minichannels, three-dimensional reconstruction of the constructs was performed. As shown in Fig. 3M, the PGS-PCL hollow minichannel, PRP, and cartilage granule-stem cell composite were designated gray, yellow, and blue, respectively, on the basis of their densities. Fig. 3M (ii) shows that PRP uniformly filled the interior of the minichannels, whereas (iii) the blue cartilage granules mixed within PRP were also uniformly distributed inside the minichannels.

4. Thickness vs. Porosity: Rather than thickness alone, reduced porosity likely limits capillary ingrowth. Please clarify if pore interconnectivity was evaluated and considered a contributing factor.

Response: Thank you for the professional questions. We have added **Figure 2D, 2G and 2H** to the revised manuscript, which includes nanoscale CT images of minichannels with different coating layers as well as quantitative analyses of their total porosity and interconnectivity. The relevant descriptions have been added to **Sections 2.1**, **2.3** and **3**.

(Main text: Section 4.6)

4.6 Pore Interconnectivity Measurement

The pore interconnectivity was calculated via the following formula:

Maximum pore volume/total pore volume (%). The analysis was performed via CTAn software (VG Studio MAX 3.5) on nanoscale CT scans (Zeiss, Xradia 520 versa, Germany) with a resolution of 2 μm (sample size of 1 mm × 1 mm).

(Main text: Section 2.1, Figures 2D, 2G and 2H)

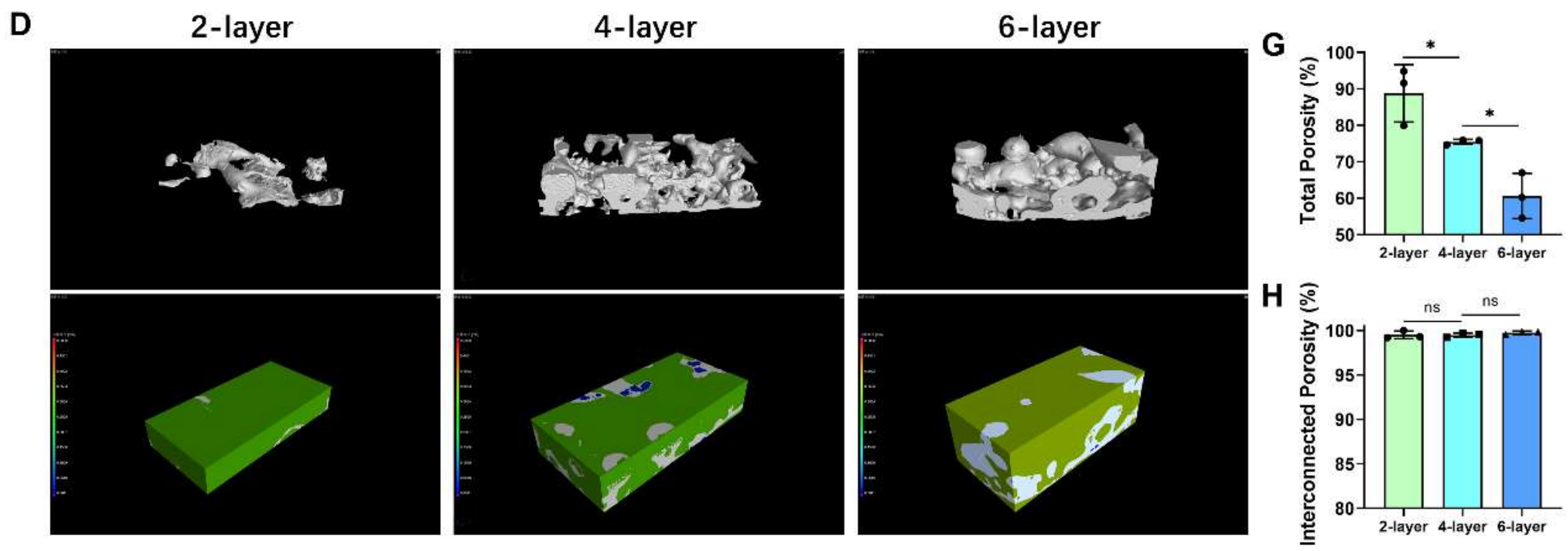

Figure 2. Characterization of 3D C-shaped minichannels.

(D) Nano-CT reconstruction images of minichannels with different coating layers. (G) Quantification of total porosity and (H) pore connectivity in 2-, 4-, and 6-layer scaffolds. Fig. 2G, and H significance was determined by one-way analysis of variance (ANOVA) followed by Tukey's post hoc analysis. $^{\#}p > 0.05$; $^{*}p < 0.05$.

(Main text) Please refer to Para 1, Section 2.1 as follows:

Increasing the number of coating layers significantly reduced the superficial and total porosities (Fig. 2E, G) ($p < 0.05$). Specifically, the total porosity was 88.81% ± 7.87% for the 2-layer, 75.48% ± 0.73% for the 4-layer, and 60.60% ± 6.21% for the 6-layer minichannels. Nano-CT analysis revealed consistently high connectivity (> 99%) across all groups (2 L: 99.50 ± 0.43%; 4 L: 99.48 ± 0.22%; 6 L: 99.75 ± 0.19%; $p > 0.05$, Fig. 2D, G and H), suggesting that increased coating layers did not compromise pore connectivity. The persistently high connectivity (> 99%) across thickness variants confirmed the unique ability of 3D minichannels to preserve pore topology, a critical property favoring host integration.

(Main text) Please refer to Para 2, Section 2.3 as follows:

The results of the porosity analysis of the scaffolds with different coating layers (Fig. 2G) confirmed that the reduction in blood vessel formation in the thicker coatings (Fig. 4E-G) was due to the decreased total porosity and extended nutrient diffusion distance, even though these pores are highly interconnected (> 99%).

(Main text) Please refer to Para 2, Section 3 as follows:

Notably, although pore connectivity exceeded 99% in all 3D minichannel coatings (Fig. 2H), the reduced porosity and increased diffusing distance in thicker coatings (6-layer) compromised capillary ingrowth, highlighting the pore volume fraction and transmural distance as dominant

regulators of graft vascularization.

5. Typographical Error: Please confirm whether "CGP group was superior to CGP and native trachea" should read "CGPA group."

Response: Thank you for pointing out this error. We confirm that this was a mistake and have corrected "CGP group" to "CGPA group" in the revised manuscript.

(Main text) Please refer to Para 5, Section 2.4, as follows:

… . Consistent with the histological results, the mechanical strength of the CGPA group was superior to that of both the CGP group and the native trachea.

6. Excess GAG and pH Effects: Higher GAG levels may indicate chondrogenesis but could acidify the microenvironment. Were any pH-related effects observed, or could this be discussed as a potential limitation?

Response: We appreciate the reviewer for raising the important point regarding the potential risk of microenvironment acidification associated with elevated GAG levels. We acknowledge this as a valid consideration. In the revised manuscript, we have added discussion of this issue in **Section 3**.

(Main text) Please refer to Para 4, Section 3, as follows:

In our study, the significantly elevated GAG content in CGPA grafts (Fig. 4I, 6G) reflects proteoglycan deposition, a hallmark of cartilage regeneration, which is crucial for the mechanical strength of cartilage. Critically, this ECM maturation occurred without inducing microenvironment acidosis, a known risk in high-GAG environments *(Am J Pathol. 2008 Jan;172(1):112-22; Int J Mol Sci. 2022 Dec 28;24(1):477)*. Histological assessment confirmed the presence of viable chondrocytes with intact lacunae and the absence of cartilage necrosis, whereas the bicarbonate buffer capacity of PRP *(Int J Mol Sci. 2020 Oct 21;21(20):7794)* maintained a balanced pH during early remodeling. This balance was further ensured by ADSC-driven vascularization, which facilitated efficient clearance of metabolic byproducts *(Front Immunol. 2024 May 21:15:1395479)*. Consequently, elevated GAG levels are directly correlated with increased compressive strength.

7. Endpoint Selection: Adverse events appeared by week 6, yet the study endpoint was set at

week 8. Please clarify whether this was predefined or adjusted based on animal condition.

Response: Thank you for this important methodological inquiry. The endpoint was predefined in the experimental protocol as 8 weeks post-implantation. However, on the basis of published research *(JAMA Otolaryngol Head Neck Surg. 2014 Jan;140(1):66-71)*, we also implemented an endpoint system based on the Wesley-Croup scoring system to monitor animal welfare daily. The animal exhibiting a score exceeding 10 (indicating severe respiratory distress) was euthanized immediately for ethical reasons. In the revised manuscript, we have supplied **Figure 8C** and **Supplementary Table S1**. The relevant descriptions have been added to **Section 2.8**.

(Main text: Section 4.15.1)

4.15.1 Wesley-Croup scoring

Respiratory function was assessed via an adapted Wesley-Croup scale *(JAMA Otolaryngol Head Neck Surg. 2014 Jan;140(1):66-71.)* (Table S1). Evaluations were performed at 24-hour intervals by two blinded experimental animal researchers. A score ≥10 mandated immediate euthanasia per the animal ethics approval (2023kq040). Statistical analysis was performed via Mann–Whitney U tests (rabbits) and linear regression (pigs).

(Main text: Section 4.15.3)

4.15.3 Porcine experiments... According to the experimental design, one animal was euthanized at the 2-week time point for early histological assessment, while the remaining five animals were euthanized at the 8-week endpoint for long-term evaluation. Animal welfare was assessed daily via the Wesley-Croup scoring system to objectively evaluate clinical conditions.

(Main text: Section 2.8, Figure 8C)

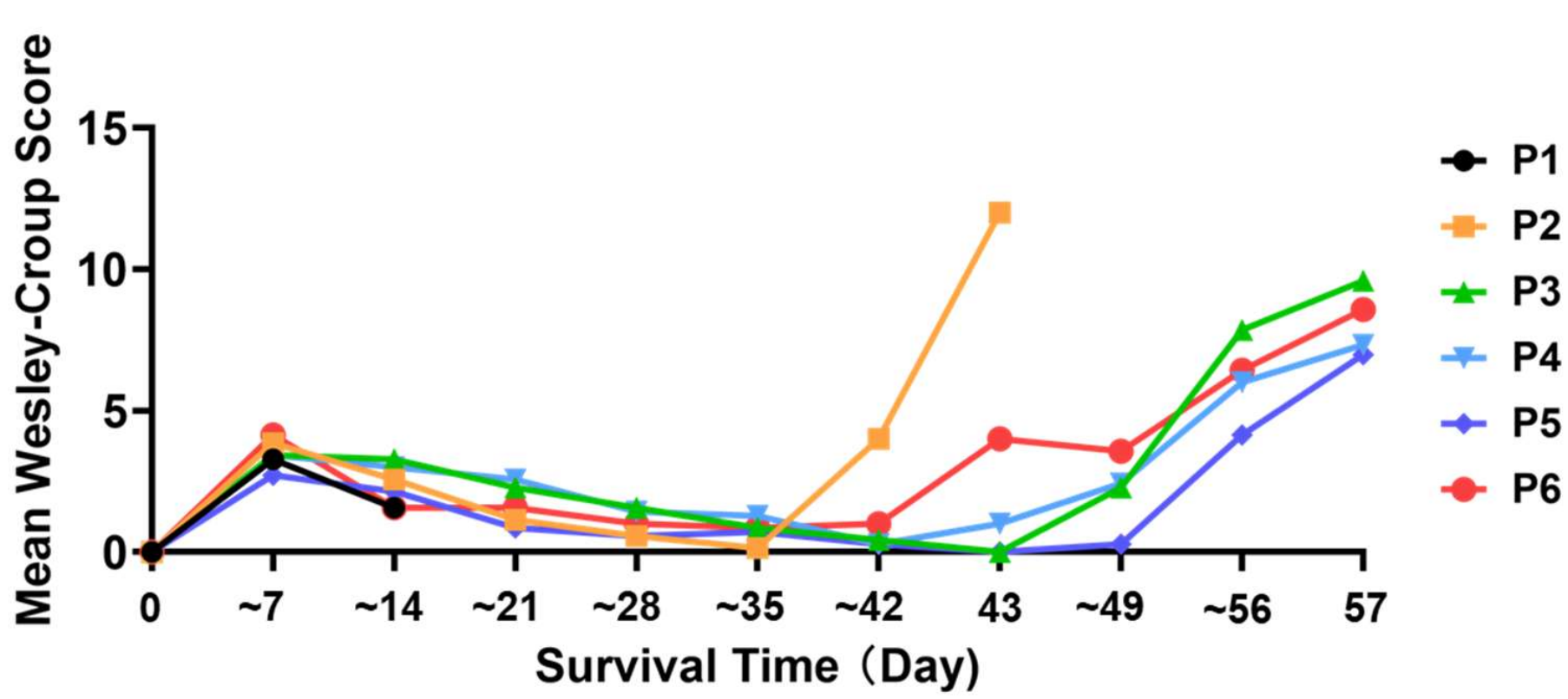

Figure 8C. Daily Wesley-Croup scores (n = 6 pigs) following segmental tracheal

reconstruction.

(Main text) Please refer to Para 2, Section 2.6 as follows:

In this study, animal welfare was monitored daily via the Wesley-Croup scoring system. The animals whose score exceeded 10 were euthanized immediately for ethical reasons. One pig experienced severe respiratory dysfunction by postoperative day 43 (6 weeks), with a Wesley-Croup score of 12, and was subsequently euthanized (Fig. 8C). The remaining four animals, although exhibiting progressive respiratory symptoms by 8 weeks, did not exceed the endpoint threshold and were euthanized as planned at the predefined 8-week time point.

(Supplementary Materials: Part III, Supplementary Table S1)

Supplementary Table S1.

Wesley-Croup scoring system for animal models

Severity Classification:

Mild: ≤4 | Moderate: 5–6 | Severe: 7–11 | Respiratory failure: ≥12

Signs, Symptoms, and Indications	Score
Subcostal retractions	
None	0
Mild	1
Moderate	2
Severe	3
Stridor	
None	0
With agitation	1
At rest	2
Cyanosis	
None	0
With agitation	4
At rest	5
Level of Consciousness	
Normal	0

Disoriented	5
Air entry	
Normal	0
Decreased	1
Markedly decreased	2

8. Epithelial Migration Evidence: Both the Rabbit and Pig experiments encountered adverse events relating to sputum retention.

(1) Was there any indication of epithelial migration onto the scaffold surface, particularly with the fascia pre-coverage?

Response: Yes, there was evidence of partial epithelial migration, particularly near the graft–host anastomoses, as indicated by discontinuous cytokeratin-positive cells in both the rabbit and pig models.

(2) If so, was this ciliated or squamous? If epithelialisation did not occur or occurred and was non-ciliated, then such retention would be expected.

Response: The migrated epithelium was not ciliated. AC-tubulin staining confirmed the absence of functional ciliated epithelium, which likely contributed to impaired mucus clearance and sputum retention.

We performed immunofluorescence staining for epithelial markers (cytokeratin for the general epithelium; AC-tubulin for the ciliated epithelium) on 8-week graft sections from the rabbit and pig models. The relevant content has been added to the **Supplementary Material (Figure S12)** and **to Sections 2.4**, **2.6** and 3 of the revised manuscript.

(Main text: Section 4.18)

4.18 Immunofluorescence Staining and Quantitative Analysis

The sections were dewaxed, rehydrated, and subjected to antigen retrieval in sodium citrate buffer. After blocking with 5% normal goat serum, the sections were incubated with primary antibodies against CD31 (NB600-562, Novus, USA, 1:200), IL-1β (67439-1-Ig, Proteintech, China, 1:300), Ac-Tubulin (66200-1-Ig, Proteintech, China, 1:300), TNF-α (26405-1-AP, Proteintech, China,

1:400), and CK (ab961, Abcam, UK, ready-to-use) overnight at 4 °C, followed by incubation with Alexa Fluor-conjugated secondary antibodies for 1 hour at room temperature. Nuclei were counterstained with DAPI (PC-90017, PCM, 1:1000). The fluorescence intensity was quantified via ImageJ software by measuring the mean intensity in three random fields per section (200x magnification), and the results are expressed as the means ± SDs.

(Supplementary Materials: Part II, Figure S12)

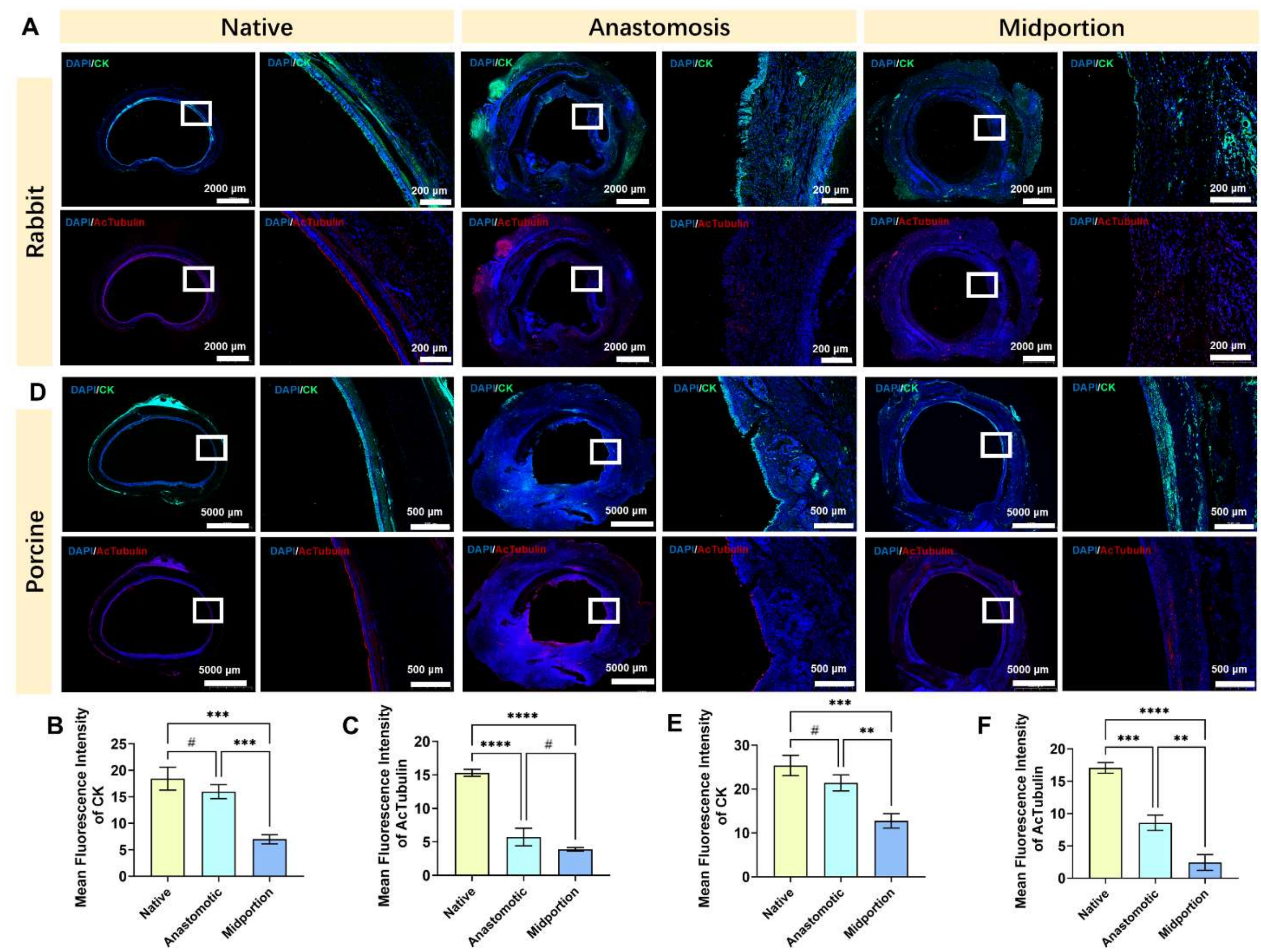

Figure S12. Epithelial marker expression in tracheal grafts. (A) Immunofluorescence of cytokeratin (CK, green) and AC-tubulin (red) in rabbit grafts: native trachea, graft anastomosis, and midportion (scale bars: 200 μm, 2000 μm). (B–C) Quantification of CK (B) and AC-tubulin (C) fluorescence intensity in rabbit grafts. (D) CK/AC-tubulin staining of pig grafts (scale bars: 500 μm, 5000 μm). (E–F) CK (E) and AC-tubulin (F) intensities in pig grafts. The significance of Fig. S12B, C, E, and F was determined via one-way analysis of variance (ANOVA) followed by Tukey's post

hoc analysis. $^{\#}p > 0.05$; $^{**}p < 0.01$, $^{***}p < 0.001$, $^{****}p < 0.0001$.

(Main text) Please refer to Para 4, Section 2.4, as follows:

To elucidate the mechanisms underlying the formation of inflammatory granulomas and the accumulation of mucus after tracheal transplantation in rabbits, epithelial and inflammation-related factors were stained. Immunofluorescence at 8 weeks revealed discontinuous cytokeratin-positive (CK+) cells at the graft–host anastomoses (Fig S12A), suggesting partial epithelial migration from the native tissue. However, no AC-tubulin+ ciliated epithelium was detected in the graft segments ($p < 0.001$ vs. native; Fig S12B, C). The absence of functional cilia, coupled with disorganized CK+ cells at anastomoses and negligible coverage in mid-grafts, explains persistent sputum retention despite fascial integration.

(Main text) Please refer to Para 3, Section 2.6 as follows:

Epithelial regeneration in the tracheal grafts of pigs was further investigated. Immunofluorescence staining revealed discontinuous CK+ cells near anastomoses (Fig. S12D, E). AC-tubulin staining revealed only sparse, noncontinuous ciliary structures in these regions (8.58 ± 1.18 vs native 17.07 ± 0.83; $p < 0.01$; Fig. S12F), and no ciliated epithelium could be detected in the mid-graft regions. Poor epithelialization, particularly the absence of functional ciliated epithelium, leads to progressive sputum accumulation and ultimately respiratory failure.

(Main text) Please refer to Para 5, Section 3, as follows:

Although immunostaining (Fig. S12) confirmed discontinuous cytokeratin expression at anastomotic sites in both species, indicating host-derived epithelial migration, ciliated epithelium was scarce in grafts from both rabbits and pigs. Fascial precoverage facilitated graft vascularization but failed to augment epithelial regeneration. Consequently, the function of mucus clearance remains impaired, leading to sputum retention and inflammation-driven tracheal stenosis.

9. PCL Longevity and Scaffold Role: Please specify the scaffold's intended functional duration and discuss whether inflammation might compromise PCL integrity. Has CGPA been tested without the PCL framework?

Response: Thank you for this very professional comment. We now provide results concerning cartilage formation from CGPA without the PCL framework and report that the intended functional

duration of PCL-based minichannel scaffolds is approximately 2 weeks. The lack of evidence that inflammation occurs after tracheal transplantation might compromise PCL integrity. In the revised manuscript, additional experiments have been supplied in the **Supplementary Materials (Figure S11),** and the relevant descriptions have been included in **Section 2.3** and **Section 3**.

(Supplementary Materials: Part I, Materials and Methods)

Scaffold-Free CGPA Graft Preparation and Testing

Scaffold-free CGPA grafts were prepared by injecting 1 ml of the cartilage granule–ADSC–PRP mixture into the abdominal fascia of rabbits. After 2 weeks of prevascularization, the grafts were harvested for mechanical testing (5 × 5 × 1.5 mm). Native tracheal cartilage samples of the same size were used as controls. Uniaxial compression tests were performed at a 50% strain rate, and the maximum compressive force was recorded (n = 3).

(Supplementary Materials: Part II, Figure S11)

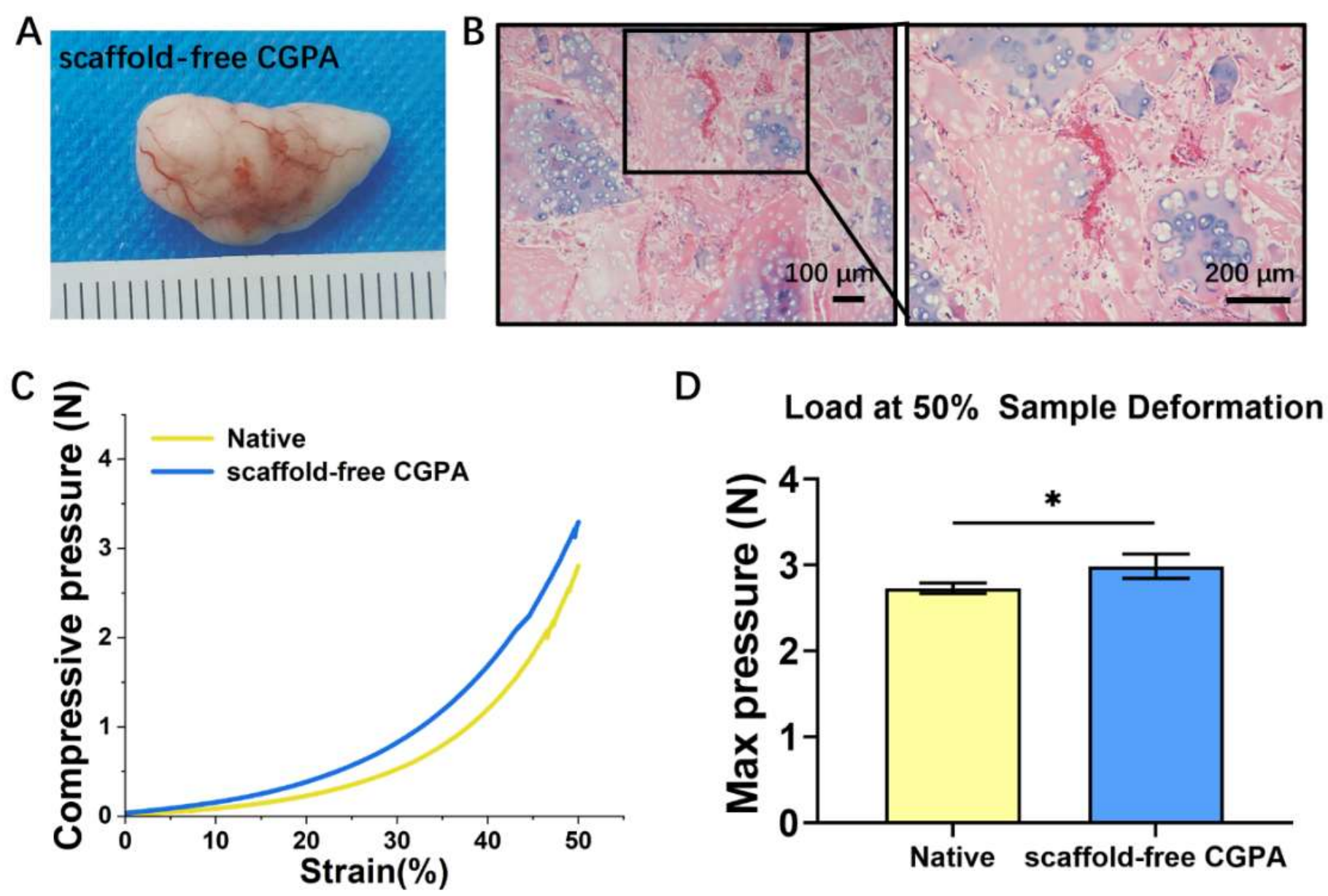

Figure S11. Characterization of scaffold-free CGPA grafts after 2 weeks of prevascularization. (A) Gross image of a scaffold-free CGPA graft showing cartilage formation with superficial vascularization. (B) H&E-stained section demonstrating well-integrated cartilage granules with visible cartilage lacunae and matrix deposition (scale bars: 100 μm, 200 μm). (C) Compressive stress–strain curves of native tracheal cartilage and scaffold-free CGPA grafts. (D) Quantitative analysis of the maximum compressive force at 50% deformation (Fig. S11D) was performed via

one-way analysis of variance (ANOVA) followed by Tukey's post hoc analysis. $^{*}p < 0.05$

(Main text) Please refer to Para 3, Section 2.3 as follows:

To assess the functional duration of the PCL-based scaffold in graft construction, scaffold-free CGPA constructs were implanted subcutaneously in rabbits for two weeks. Gross observation revealed that the injected cartilage granules completely turned into spheric cartilage tissues, with a porcelain white color, smooth appearance, and extensive angiogenesis. H&E staining revealed well-integrated cartilage granules with distinct chondrocyte lacunae and a deeply stained extracellular matrix (ECM) (Fig. S10B). Mechanical testing revealed that the stress–strain curves of the scaffold-free CGPA grafts closely resembled those of the native tracheal cartilage (Fig. S10C). Quantitative analysis revealed a maximum compressive force at a 50% strain of 2.99 ± 0.14 N for the scaffold-free CGPA grafts, which was significantly greater than that of the native tracheal cartilage (2.73 ± 0.06 N, $p < 0.05$) (Fig. S10D). Additionally, compared with the hollow minichannel scaffold group, the CGPA group acquired after the 2-week prevascularization period presented a significant increase in compressive strength (from 0.71 ± 0.15 N to 2.15 ± 0.11 N, $p < 0.001$), confirming that the PCL-based scaffold primarily serves as a biomimetic template during early remodeling. These results suggested that the cartilage granules may rapidly reconstitute into mechanically robust constructs, and the intended functional duration of PCL minichannels is approximately 2 weeks.

(Main text) Please refer to Para 3, Section 3 as follows:

Crucially, the scaffold-free CGPA grafts achieved mechanical properties superior to those of native tracheal cartilage within 2 weeks, highlighting the rapid remodeling potential of the cartilage granule-ADSC-PRP composite. This confirms that the PCL-based scaffold acts primarily as a molding template rather than as a permanent structural support, and the intended functional duration may be approximately 2 weeks. The immunofluorescence of inflammatory factors (Fig. S13) revealed that the inflammatory reaction, characterized by IL-1β and TNF-α, was mainly localized to the luminal granulation tissue but was scarcely detected around the PCL materials. After tracheal transplantation, no evidence of progressive degradation, collapse, or inflammatory compromise of the PCL scaffold in the grafts was detected when the scaffold was explanted at 2 and 8 weeks.

10. Reagent-Grade PCL: Was the reagent-grade PCL purified or endotoxin-tested prior to in

vivo use?

Response: We sincerely appreciate your comments regarding the purification of PCL and control of endotoxins, which are essential for the evaluation of biocompatibility. The PCL we used was purchased from Sigma–Aldrich (440744, Sigma–Aldrich, USA) and has been verified to have a purity of 99.8%, which is widely used in medical research. In the revised manuscript, we have added endotoxin testing for the PCL material and the PGS-PCL minichannels to the **Supplementary Materials (Figure S3),** and a relevant description has been added to **Section 2.1**.

(Main text: Section 4.9)

4.9 Endotoxin detection

Endotoxin contamination of reagent-grade PCL (440744, Sigma–Aldrich, USA) was assessed via an endotoxin detection kit (A39552, Thermo Fisher Scientific, China). A standard curve with endotoxin concentrations ranging from 0.1 EU/ml to 1 EU/ml was first established. The samples (1 g/ml in endotoxin-free water) were incubated at 37 °C for 24 hours. The supernatant was then analyzed spectrophotometrically at 405 nm.

(Supplementary Materials: Part II, Figure S3)

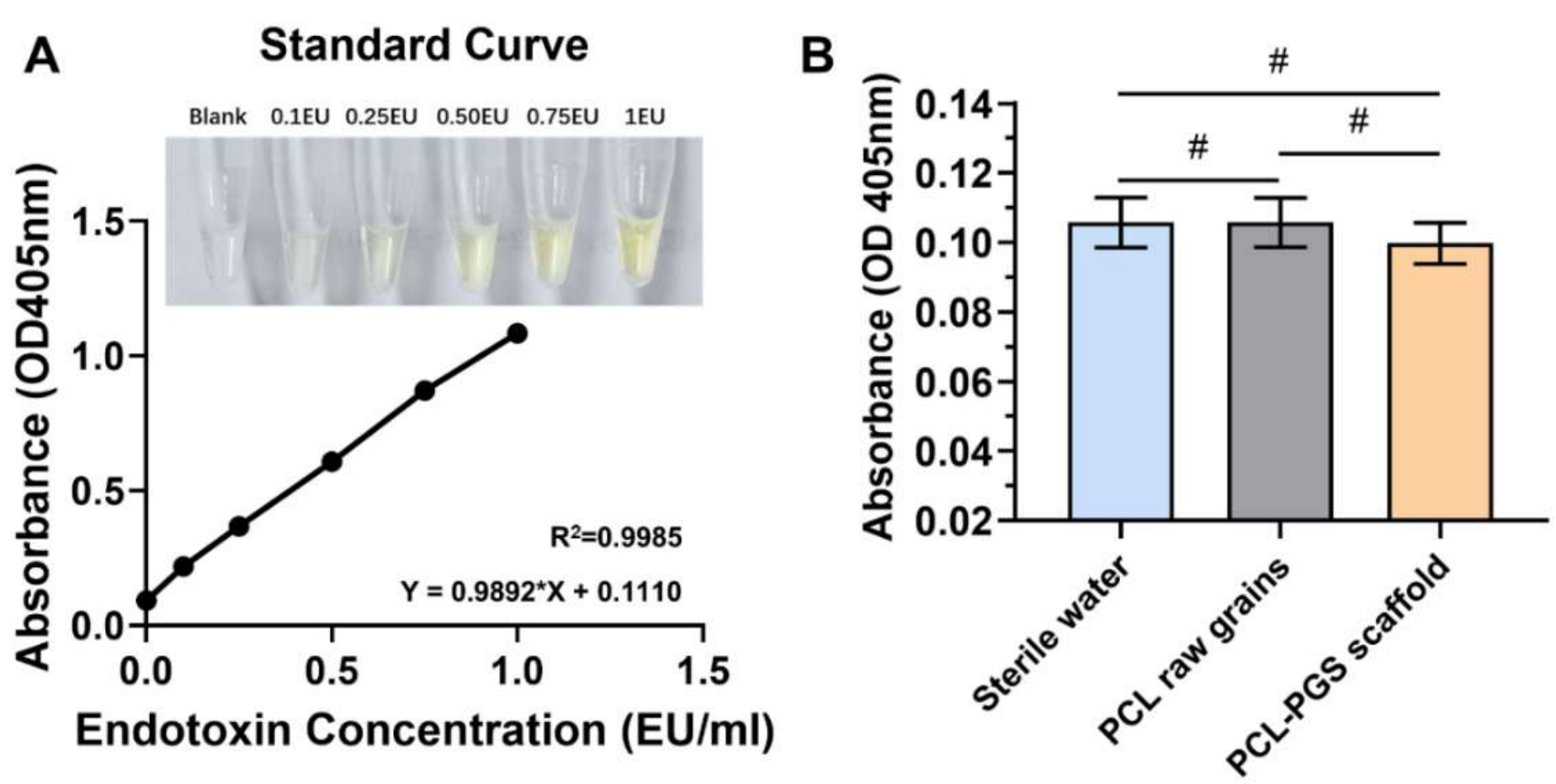

Figure S3. Endotoxin test results. (A) Endotoxin detection standard curve. (B) Endotoxin levels in sterile water, PCL raw material, and PCL-PGS minichannels. As shown in Fig. S3B, significance was determined by one-way analysis of variance (ANOVA) followed by Tukey's post hoc analysis. $^{\#}p > 0.05$.

(Main text) Please refer to Para 2, Section 2.1 as follows:

Furthermore, endotoxin testing confirmed that the PCL-PGS minichannels were not contaminated

(0.100 ± 0.006 EU/ml), with no significant difference from the sterile water controls ($p > 0.05$; Fig. S3).

11. Residual HFIP: Has residual solvent been assessed post-fabrication? Data (e.g., FTIR, TGA) would help confirm safety.

Response: We appreciate the reviewer for raising this important safety concern. We have supplemented the Fourier transform infrared spectroscopy (FTIR) analysis of the scaffold in the **Supplementary Materials (Figure S2)**, confirming the absence of residual hexafluoro-2-propanol solvent. In the revised manuscript, we have also included relevant content in **Section 2.1**.

(Main text: Section 4.7)

4.7 Residual solvent analysis

The postfabrication scaffolds were lyophilized and analyzed via FTIR spectroscopy (Thermo Scientific Nicolet iS 5) to detect residual hexafluoroisopropanol (HFIP). The spectra were collected over 64 scans at a resolution of 4 cm^{-1} (range: 4000–500 cm^{-1}). The absence of C–F bond peaks (1200--1250 cm^{-1}) confirmed solvent elimination.

(Supplementary Materials: Part II, Figure S2)

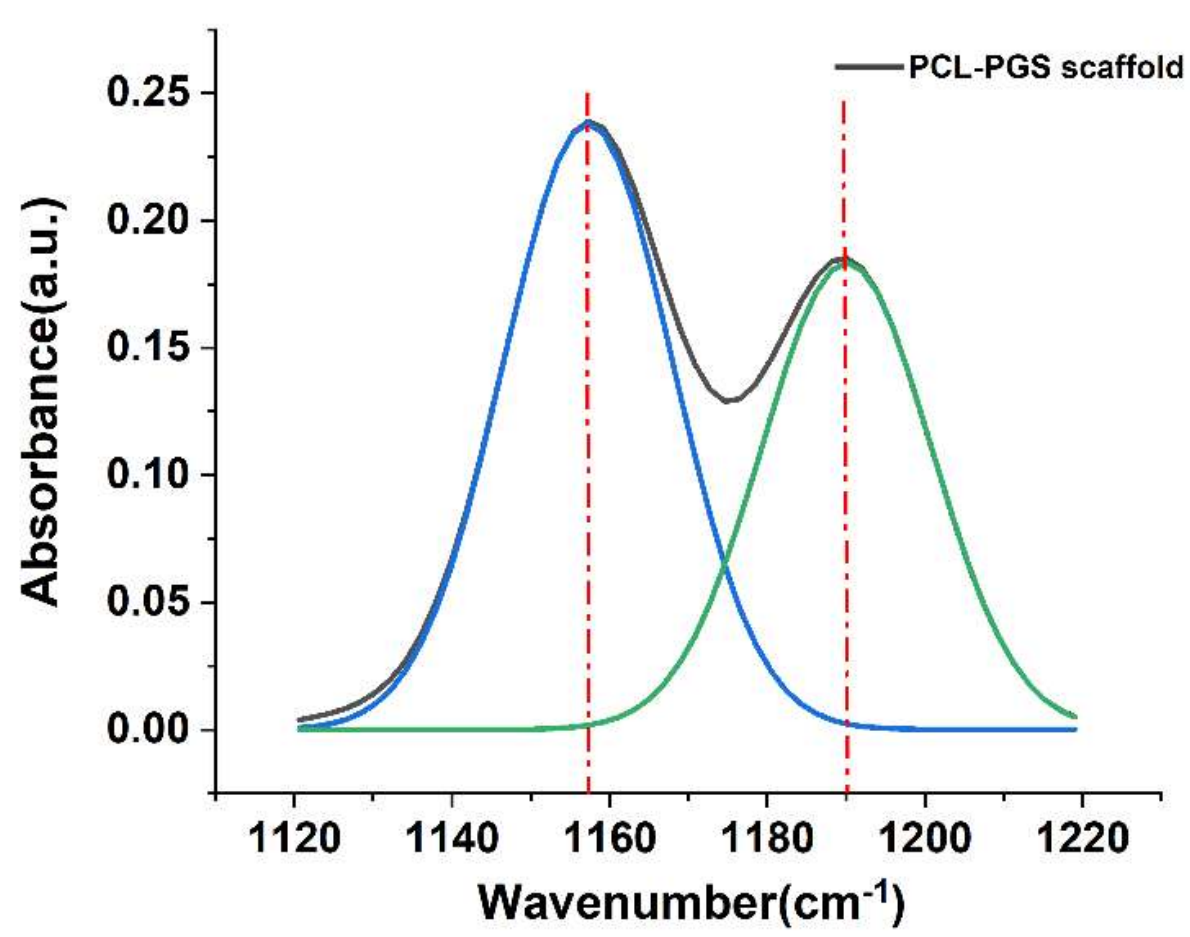

Figure S2. FTIR spectra of the PCL-PGS scaffold. FTIR spectra confirming the absence of residual HFIP in the scaffolds (gray). After deconvolution, characteristic PCL (1157.3 cm^{-1}, blue) and PGS (1190.1 cm^{-1}, green) peaks are labeled. HFIP control results in C–F bonds at 1218 cm^{-1}, which are absent in the scaffolds.

(Main text) Please refer to Para 1, Section 2.1 as follows:

FTIR spectroscopy further confirmed scaffold biosafety, with spectra showing characteristic PCL (1157.3 cm^{-1}) and PGS (1190.1 cm^{-1}) peaks and no detectable residual HFIP (C–F bonds at 1200–1250 cm^{-1}; Fig. S2).

12. Cryo-Grinding Protocol: Were cryoprotectants used during cartilage grinding? Including this detail would aid reproducibility.

Response: Thank you for this insightful question regarding the methodological details. In the cryogrinding protocol described in our study, no cryoprotectants (e.g., DMSO, glycerol, or commercial freezing media) were used. We have added a test for comparing cell viability in which cartilage tissues were pretreated with a cryoprotective agent (C40100, NCM Biotech, China) or not pretreated, as shown in the **Supplementary Materials (Figure S5).** In the revised manuscript, we have also included relevant content in **Section 2.2.**

(Main text: Section 4.12)

The cartilage was dissected into 1×1 cm pieces and washed three times with PBS. The washed cartilage fragments were then flash-frozen in liquid nitrogen for 3 minutes without the cryoprotectants. The frozen fragments were subsequently homogenized via a tissue grinder at 1600 × g for durations of 20, 30, or 40 s to generate cartilage granules.

(Supplementary Materials: Part II, Figure S5)

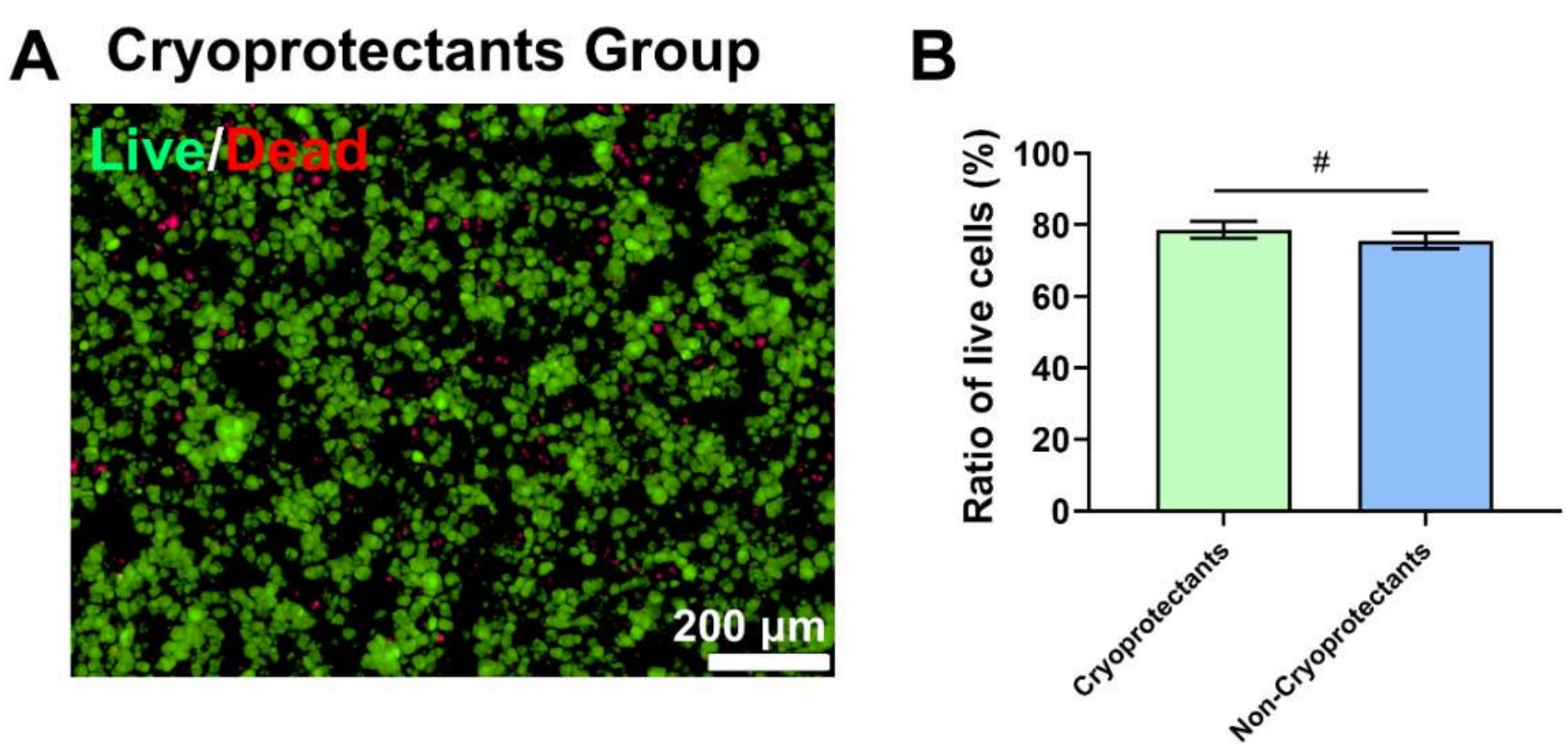

Figure S5. Analysis of cell viability in cartilage granules treated with cryopreservation solution. (A) Live/Dead Cell Staining of Cartilage Particles under Cryopreservation Conditions

(Scale bar: 200 μm). (B) Quantitative analysis of cell survival between groups with and without cryopreservation solution. The significance of the data in Fig. S5B was determined via one-way analysis of variance (ANOVA) followed by Tukey's post hoc analysis. #$p > 0.05$.

(Main text) Please refer to Para 1, Section 2.2 as follows:

To assess potential viability enhancement, cartilage fragments were treated with commercial cryopreservation solution prior to grinding. Compared with the standard cryo-grinding protocol, live/dead analysis revealed no significant improvement in chondrocyte survival (78.66% ± 2.41% vs. 75.57% ± 2.22% at 30 s; $p > 0.05$; Fig. S5). Consequently, cryoprotectants were excluded from further research.

13. GMP/Quality Standards: Were any GMP or equivalent standards applied to fabrication or sterilization? This would speed regulatory approvals for clinical trials.

Response: Yes, good manufacturing practice (GMP) standards were applied to both the fabrication and the sterilization processes in this study. Specifically, a GMP-certified laboratory at our university provided the foundational framework, and all experimental procedures, including material preparation, scaffold fabrication, and sterilization, were conducted in accordance with GMP-equivalent protocols. This approach ensures reproducibility, quality control, and compliance with regulatory requirements, thereby facilitating future approvals for clinical trials.

(Main text: Section 4.2)

4.2 Considerations for Clinical Translation

Although this study was conducted in a preclinical academic setting, all the procedures were designed with clinical translation in mind. Scaffold fabrication and cell processing were carried out under ISO-class cleanroom conditions via standardized operating protocols (SOPs) to minimize contamination. Sterilization was achieved via 75% ethanol disinfection combined with UV irradiation, which was validated through sterility testing. Although the current experiments were not performed in a fully GMP-certified facility, the protocols established here are aligned with GMP principles, ensuring the transition toward clinical trials.

14. CCK-8 Volume Discrepancy: Using 1 mL in a 96-well plate seems inconsistent with well

capacity. Please clarify.

Response: We apologize for the unintentional documentation error in the manuscript. We have corrected this mistake and rephrased it in the revised manuscript.

(Supplementary Materials: Part I, Materials and methods)

CCK-8 assays: The cytotoxicity of different layered materials on chondrocytes was evaluated via a CCK-8 assay kit (PC-1050, PCM, China). PCL/PGS scaffolds were placed in a 96-well plate with equal amounts of cartilage particulate matter. Each well was filled with 300 µl of culture medium and incubated in a CO_2 incubator. On days 1, 3, and 5, 100 µl of culture medium containing 10% CCK-8 reagent was added to each well of a 96-well plate. After 2 h of incubation at 37 °C, the absorbance was measured at 450 nm (n = 3).

15. Adverse Events Interpretation: Since epithelial regeneration was limited, could the complications also involve scaffold inflammation or remodelling issues? The latter is a major issue with previous attempts at tracheal scaffold production (e.g. Eur Respir J. 2020 Jun 18;55(6):1901200). Clarifying this would improve interpretation.

Response: Thank you for highlighting this important issue. Moreover, our results indicate that the PCL-based scaffold itself did not elicit significant inflammatory responses or adverse remodeling. The dominant factor was the lack of functional epithelial coverage, which impaired mucus clearance and promoted granulation tissue formation. We agree that optimizing epithelialization strategies in combination with our graft design will be essential to improve functional outcomes in future studies, the relevant description in **Section 3.**

(Main text) Please refer to Para 5, Section 3, as follows:

Although immunostaining (Fig. S12) confirmed discontinuous cytokeratin expression at anastomotic sites in both species, indicating host-derived epithelial migration, ciliated epithelium was scarce in grafts from both rabbits and pigs. Fascial precoverage facilitated graft vascularization but failed to augment epithelial regeneration. Consequently, the function of mucus clearance remains impaired, leading to sputum retention and inflammation-driven tracheal stenosis. Current approaches include preseeding epithelial cells, iPSC-derived mesenchymal stem cells/chondrocytes (outer layer) and human bronchial epithelial cells (hBECs) (inner layer) onto tracheal grafts or

fabricating 3D-bioprinted tracheal constructs with epithelial cells *(Eur Respir J. 2020 Jun 18;55(6):1901200; Biomaterials. 2021 Dec: 279:121246; Int J Mol Sci. 2021 Sep 25;22(19):10322; Sci Rep. 2020 Mar 9;10(1):4326)*. However, these strategies are clinically limited owing to the long culture period (2–6 weeks), high contamination risk, and restricted cell seeding efficiency and cell survival rate *(Eur Respir J. 2020 Jun 18;55(6):1901200)*. For epithelialization, our minichannel strategy may be modified via two approaches. First, during the prevascularization phase, the micrometer-scale mesh may be fabricated intraluminally via electrohydrodynamic (EHD) printing *(Sci Transl Med. 2023 Sep 20;15(714): eabo4272)* as an adhesive interface for efficiently seeding respiratory epithelial cells or mucosa sheets. Simultaneously, with vascularization, the luminal surface undergoes epithelial remodeling. Second, on the basis of our previous explorations of airway epithelial regeneration, cartilaginous ECM enriched with gingival extracellular vesicles (gEVs) could be bioengineered as a bioactive barrier against contamination to recruit epithelial progenitors via JAK2-STAT1 signaling *(Adv Sci (Weinh)). 2022 Jan;9(2): e2102735)*.

16. Inflammatory Markers: Were cytokines such as IL-1β, TNF-α, or IL-6 assessed to evaluate immune response? This could help differentiate structural from immunological failure.

Response: Thank you; this is a very professional suggestion. To more clearly illustrate the inflammatory response within the grafts, we have included additional immunofluorescence staining for inflammatory factors (IL-1β, TNF-α) in the **Supplementary Materials (Figure S13)** and included relevant content in **Section 2.4** and **Section 3**.

(Main text: Section 4.18)

4.18 Immunofluorescence Staining and Quantitative Analysis

The sections were dewaxed, rehydrated, and subjected to antigen retrieval in sodium citrate buffer. After blocking with 5% normal goat serum, the sections were incubated with primary antibodies against CD31 (NB600-562, Novus, USA, 1:200), IL-1β (67439-1-Ig, Proteintech, China, 1:300), Ac-Tubulin (66200-1-Ig, Proteintech, China, 1:300), TNF-α (26405-1-AP, Proteintech, China, 1:400), and CK (ab961, Abcam, UK, ready-to-use) overnight at 4 °C, followed by incubation with Alexa Fluor-conjugated secondary antibodies for 1 hour at room temperature. Nuclei were counterstained with DAPI (PC-90017, PCM, 1:1000). The fluorescence intensity was quantified via

ImageJ software by measuring the mean intensity in three random fields per section (200x magnification), and the results are expressed as the means ± SDs.

(Supplementary Materials: Part II, Figure S13)

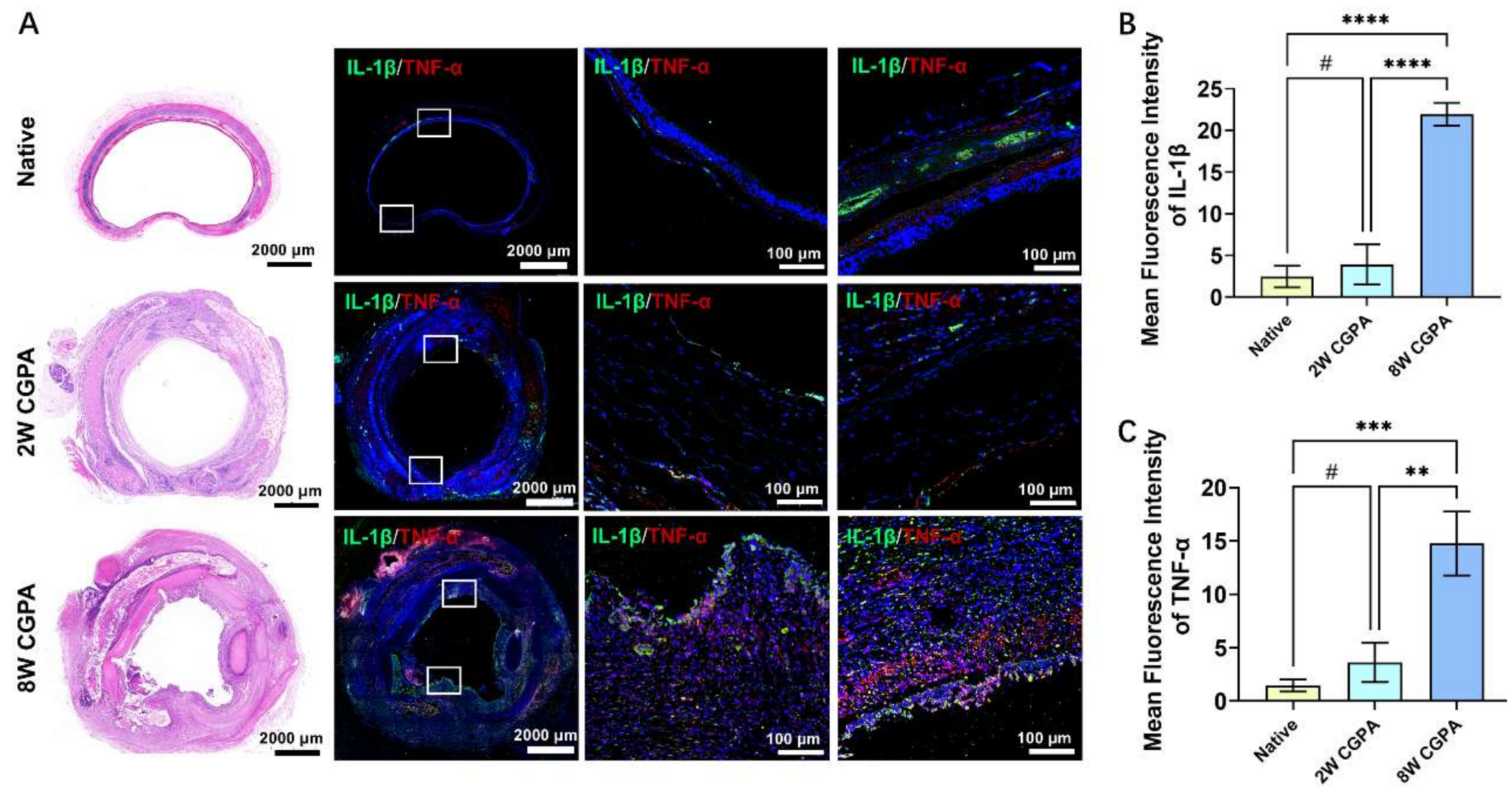

Figure S13. Inflammation marker expression in different tracheal grafts. (A) Immunofluorescence for IL-1β (green) and TNF-α (red) in grafts. Native tissue shows baseline signals (scale bars: 100 μm, 2000 μm). The 2 W CGPA grafts exhibited mild inflammation. 8 W CGPA grafts reveal intense IL-1β/TNF-α in granulation tissue. (B) Quantitative analysis of IL-1β intensity. (C) Quantitative analysis of TNF-α intensity. Fig. S13B, and C significance was determined via one-way analysis of variance (ANOVA) followed by Tukey's post hoc analysis. $^{\#}p > 0.05$; $^{**}p < 0.01$, $^{***}p < 0.001$, $^{****}p < 0.0001$.

(Main text) Please refer to Para 4, Section 2.4, as follows:

Notably, the inflammatory cascade is secondary to epithelial dysfunction. While early grafts (2 W) did not significantly increase the levels of cytokines (IL-1β: 3.93 ± 2.41; TNF-α: 3.65 ± 1.85; $p > 0.05$ vs. native), 8 W grafts exhibited dramatic increases in the levels of IL-1β (21.95 ± 1.38) and TNF-α (14.77 ± 2.99; $p < 0.01$), which are localized in luminal granulations (Fig. S13). These findings confirmed that inflammation arises from sputum retention, which is due to an insufficient epithelial barrier and prolonged exposure to the opening airway, rather than scaffold incompatibility.

(Main text) Please refer to Para 2, Section 3 as follows:

Although PCL degrades slowly (> 12 months), its biocompatibility minimizes inflammatory

responses, as evidenced by negligible IL-1β/TNF-α elevation in grafts at 2 weeks, confirming the suitability of PCL for sustained mechanical stabilization. In our research, adverse events reflected secondary pathophysiology: sputum accumulation in epithelium-deficient grafts triggered progressive inflammation (Fig. S13) and ultimately led to respiratory failure.

We have tried our best to improve the manuscript and made some changes in the manuscript. These changes did not influence the content or framework of the paper. Here, we did not list the changes but marked them in red in the revised manuscript.

We earnestly appreciate your warm work and hope that the corrections will be met with approval.

Once again, thank you very much for your comments and suggestions. Please free to inform me if you remain further concerned, we will revise them according to your further requests.

Reviewer #4: I co-reviewed this manuscript with one of the reviewers who provided the listed reports. This is part of the Nature Communications initiative to facilitate training in peer review and to provide appropriate recognition for Early Career Researchers who co-review manuscripts.

Response: We greatly appreciate the time and effort you have dedicated to evaluating our manuscript. We have carefully considered all the comments provided and have made point-to-point revisions accordingly. We believe that the manuscript now meets the publication standards of *Nat Commun* and hope that the changes satisfactorily address your concerns.

We have tried our best to improve the manuscript and made some changes in the manuscript. These changes did not influence the content or framework of the paper. Here, we did not list the changes but marked them in red in the revised manuscript.

We earnestly appreciate your warm work and hope that the corrections will be met with approval.

Once again, thank you very much for your comments and suggestions. Please free to inform me if you remain further concerned, we will revise them according to your further requests.

Appeal letter to *Nature Communications* on Rejected article entitled 3D minichannels morphed "Adipose Stem cells - Cartilage granules" reconstituted tough grafts for repairing segmental tracheal defects in preclinical animal models (NCOMMS-25-31353A)

Page No.

Cover …………………………………………………………………………………………… 1

Response to Concerns from Reviewer…………………………………………………………… 2

Response to Concerns from Reviewer #5

Reviewer #5: This manuscript details the use of a novel tracheal construct for eventual use for a host of airway pathologies requiring replacement of multiple tracheal rings. There is an unmet clinical need for improving airway issues in patients with tracheomalacia, stenosis, and malignancies etc. The authors use a combination of 3-D printing along with adipose derived stem cells and cartilage chips to facilitate rapid cartilage formation. The preclinical studies included both rabbits and a porcine model for testing of this concept. There remain a number of questions that should be addressed, and they are listed below.

Response: We sincerely thank you for the thorough and insightful evaluation of our manuscript. We have carefully considered each point and have provided detailed, point-by-point responses below. Corresponding clarifications and enhancements have been integrated into the revised manuscript to strengthen the rationale, interpretation, and clinical relevance of our findings. We believe these revisions have significantly improved the manuscript and hope it now meets the high standards for publication.

1. The time points are rather short for this type of study. Even at 2 months you already are seeing failures so what is the effective lifespan of these constructs?

Response: We appreciate your request regarding the time points of our study. We would like to clarify that the 8 weeks endpoint was a pre-scheduled time point for histological and biomechanical analysis, not an indicator of graft failure. This observation period is firmly established and widely accepted within the field of preclinical tracheal reconstruction, as evidenced by numerous high-impact studies utilizing similar or identical time frames across various large animal models, including rabbits, pigs, and sheep. This consensus highlights that our chosen time point is not only suitable but also meets the standards of translational research.

References	Species	Observation Duration
Tissue Eng Part A. 2016 Sep;22(17-18):1086-97.	rat	8 weeks
Biomater Biosyst. 2023 Feb 3:9:100074.	rat	8 weeks
Sci Transl Med. 2023 Sep 20;15(714):eabo4272.	rabbit	8 weeks
Laryngoscope. 2020 Dec;130(12):E767-E772.	rabbit	7 weeks
Biomaterials. 2020 Nov:260:120281.	rabbit	8 weeks
Ann Thorac Surg. 2017 Sep;104(3):958-963.	pig	34 days
Laryngoscope. 2018 Dec:128 Suppl 6:S1-S9.	pig	2 weeks
Sci Rep. 2023 Dec 8;13(1):21773.	pig	2 months
Tissue Eng Regen Med. 2025 Jun;22(4):469-479.	pig	45 days
Tissue Eng Part A. 2018 Sep;24(17-18):1301-1308.	sheep	10 weeks
Laryngoscope. 2019 Aug;129(8):1763-1771.	human	10-56 days

To further present the reconstruction effect of the constructs, we extended the observation duration in our porcine model. Specifically, two animals were monitored for 86 and 115 days post-implantation, respectively. The relevant results have been added in **Fig.8 and S16**, and corresponding descriptions have been added in **Section 2.6**.

(Main text) Please refer to Fig. 8B, C, as follows:

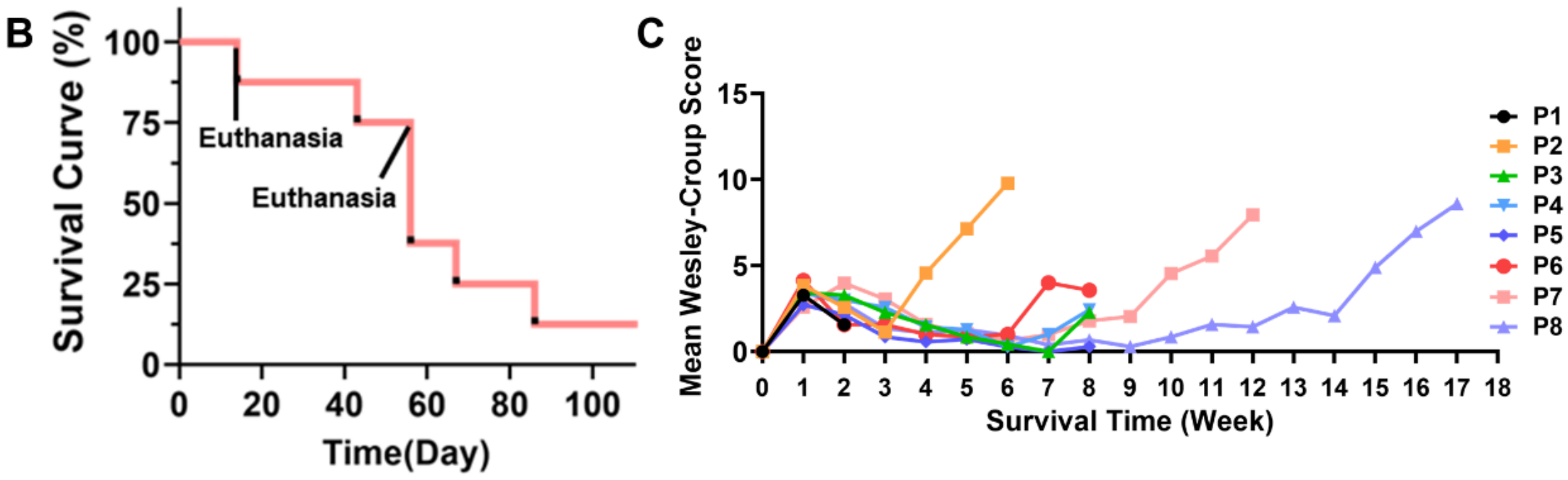

Figure 8. (B) Kaplan–Meier survival curve depicting the survival rate of pigs following CGPA graft implantation (n = 8). (C) Daily Wesley-Croup scores (n = 8 pigs) following segmental tracheal reconstruction.

(Main text) Please refer to Pars 2, Section 2.6, as follows:

Kaplan–Meier analysis indicated an 88.9% survival rate (6/7) at 8 weeks post-implantation, with one animal electively euthanized at the 2-week endpoint for histological assessment (1/8) (Fig. 8B). Animal welfare was monitored daily using the Wesley-Croup scoring system, and animals exceeding a score of 10 were euthanized promptly for ethical reasons (Fig. 8C). One pig developed respiratory dysfunction by postoperative day 43, reaching a Wesley-Croup score of 12, and was subsequently euthanized. Four animals, exhibiting only mild respiratory signs, were euthanized as scheduled at the predefined 8-week time point. The remaining two animals were monitored beyond 8 weeks and euthanized upon reaching the Wesley-Croup threshold at 86 and 115 days, respectively, confirming the potential for extended graft patency and functional integration.

(Main text) Please refer to Figure S16, as follows:

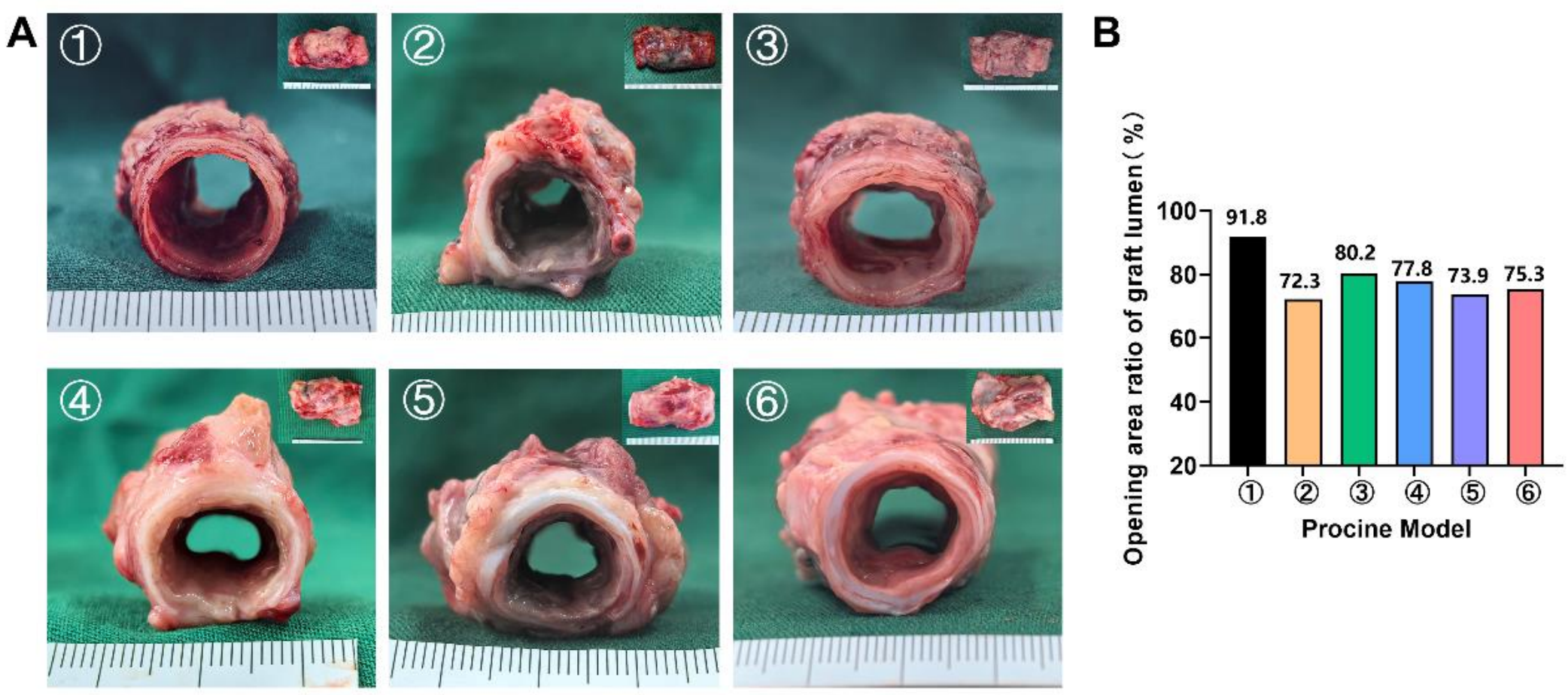

Figure S16. Gross analysis of explanted porcine tracheal grafts. (A) Lumen opening images of grafts harvested from six pigs. ①Tracheal graft harvested at 2 weeks, showing no significant granulation tissue or luminal stenosis in the graft. ②-④ Tracheal graft harvested at 8 weeks. ⑤

Tracheal grafts harvested at 12 weeks (86 days). ⑥ Tracheal grafts harvested at 16 weeks (115 days). (B) Quantitative analysis of the lumen opening area.

(Main text) Please refer to Pars 3, Section 2.6, as follows:

Gross examination of explanted grafts (Fig. S16) confirmed that the lumen patency area remained above 70% of the original opening area beyond 8 weeks, including in the two animals observed long-term. Notably, the cartilaginous architecture of all grafts was structurally intact without collapse, demonstrating the reliable mechanical resilience of the remodeled cartilage granule-based constructs.

2. The epithelial layer is probably the most important component of the tracheal construct. The authors have done extensive optimization of many of the parameters for trachea but have completely ignored the most important aspect for success: ciliated epithelium to move fluid throughout the airway.

Response: We fully acknowledge that ciliated epithelium remains the gold standard for long-term tracheal graft success. However, it is important to note that regeneration of a functional ciliated epithelium remains a major unmet challenge in both experimental and clinical tracheal replacement, as highlighted in recent comprehensive reviews (*Davide Adamo et al., Front Bioeng Biotechnol 2022; Martinod et al., JAMA Surg 2025*). Even in the most clinically advanced approaches, such as cryopreserved aortic allografts, epithelial regeneration is often incomplete, and long-term stenting is frequently required to maintain lumen patency and manage secretions. In many clinical scenarios, the establishment of a continuous protective epithelial lining- even if initially squamous- is already considered a favorable outcome, as it provides a critical barrier against infection, supports graft integration, and enables patient survival without the need for permanent external support (*Shixiong Wei et al., Regen Ther 2022; Fabre et al., Ann Thorac Surg 2013; Etienne et al., Eur Respir J 2018*).

Our data demonstrate that the vascularized grafts support progressive epithelialization, with cytokeratin-positive lining established by 8 weeks and early evidence of ciliated differentiation (acetylated tubulin positivity) in the porcine model. While the epithelium at this stage is not fully ciliated, the establishment of a continuous, protective squamous epithelial layer aligns with the current clinical reality in tracheal reconstruction. The relevant descriptions have integrated into the **Sections 2.4, 2.6** and **Section 3** in the revised manuscript.

(Supplementary Materials) Please refer to Figure S12, as follows:

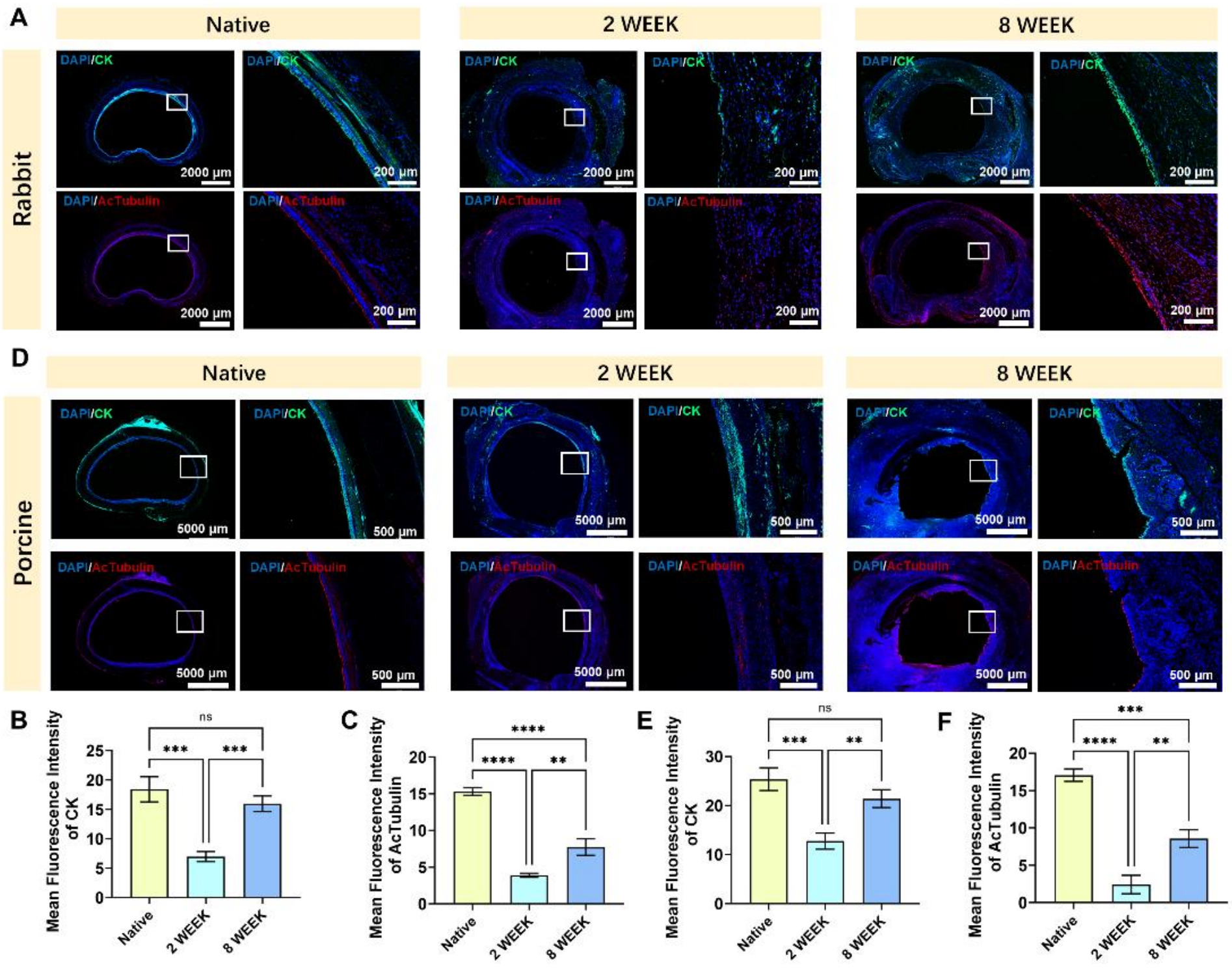

Figure S12. Epithelial marker expression in tracheal grafts. (A) Immunofluorescence of cytokeratin (CK, green) and AC-tubulin (red) in rabbit grafts: native trachea, graft anastomosis, and midportion (scale bars: 200 µm, 2000 µm). (B–C) Quantification of CK (B) and AC-tubulin (C) fluorescence intensity in rabbit grafts. (D) CK/AC-tubulin staining of pig grafts (scale bars: 500 µm, 5000 µm). (E–F) CK (E) and AC-tubulin (F) intensities in pig grafts. The significance of Fig. S12B, C, E, and F was determined via one-way analysis of variance (ANOVA) followed by Tukey's post hoc analysis. # $p > 0.05$; $^{**}p < 0.01$, $^{***}p < 0.001$, $^{****}p < 0.0001$.

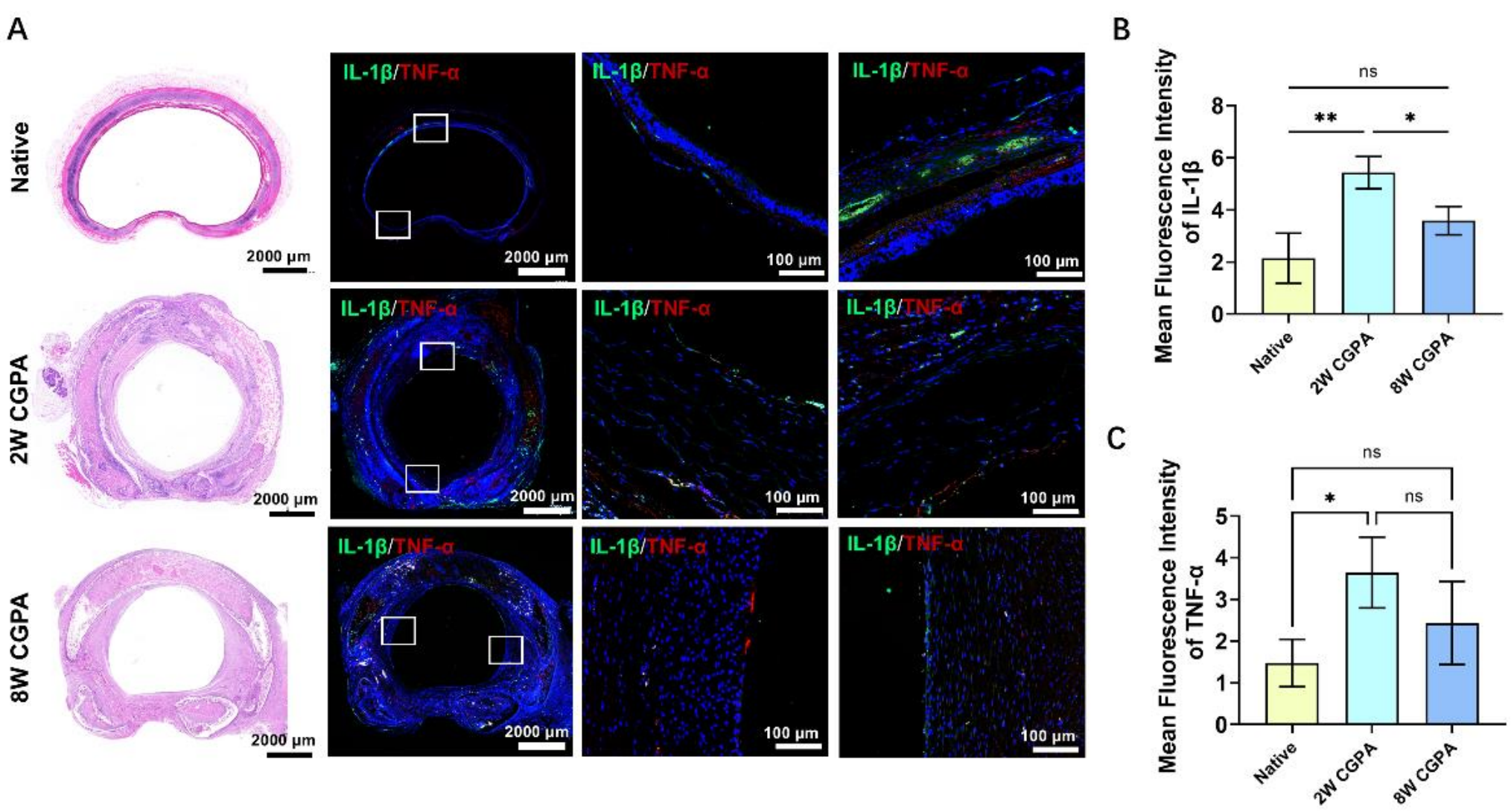

Figure S13. Inflammation marker expression in different tracheal grafts. (A) Immunofluorescence for IL-1β (green) and TNF-α (red) in grafts. Native tissue shows

baseline signals (scale bars: 100 μm, 2000 μm). The 2 W CGPA grafts exhibited mild inflammation. 8 W CGPA grafts reveal intense IL-1β/TNF-α in granulation tissue. (B) Quantitative analysis of IL-1β intensity. (C) Quantitative analysis of TNF-α intensity. Fig. S13B, and C significance was determined via one-way analysis of variance (ANOVA) followed by Tukey's post hoc analysis. # $p > 0.05$; ** $p < 0.01$, *** $p < 0.001$, **** $p < 0.0001$.

(Main text) Please refer to Pars 3, Section 2.4, as follows:

Epithelial regeneration within the graft lumen was evaluated over time (Fig. S12A-C). At 2 weeks, cytokeratin (CK)+ cells were sparsely distributed. By 8 weeks, CK immunoreactivity had increased significantly, forming a more contiguous epithelial-like layer with fluorescence intensity comparable to that of native trachea (15.98 ± 1.31 vs. 18.42 ± 2.14, $p > 0.05$). In contrast, acetylated tubulin (Ac-tubulin) expression was detected but was sparse and of low intensity (2.43 ± 1.23 vs. native 17.07 ± 0.83, $p < 0.01$), suggesting that the regenerated epithelium at this stage was predominantly of a squamous phenotype, not yet fully differentiated into a mature, ciliated respiratory epithelium. This pattern of squamous epithelial regeneration, supported by the underlying vascularized fascia, appeared sufficient to provide a protective barrier. The low local inflammatory response corroborated this functional protection, as levels of the pro-inflammatory cytokines IL-1β (3.59 ± 0.55) and TNF-α (2.43 ± 1.00) at 8 weeks were not significantly elevated compared to native trachea (2.14 ± 0.96 and 1.48 ± 0.57, respectively; $p > 0.05$) (Fig. S13). These data indicate that the establishment of a protective squamous epithelial layer contributes to resist infection during the long-term remodeling phase.

(Main text) Please refer to Pars 3, Section 2.6, as follows:

Histological analysis of porcine tracheal grafts indicated progressive epithelial regeneration during the observation period (Fig. S12D- F). At 2 weeks post-implantation, only discontinuous cytokeratin-positive (CK+) cells were present. By 8 weeks, continuous CK+ epithelial coverage was established, with a fluorescence intensity of 21.41 ± 1.83, nearing that of native trachea (25.37 ± 2.31). Evidence of ciliated epithelial regeneration also appeared at this time point, as shown by increased acetylated tubulin (Ac-Tubulin) fluorescence intensity (8.58 ± 1.18 at 8 weeks vs. 2.43 ± 1.23 at 2 weeks), though still below the native level (17.07 ± 0.83). This epithelialization, particularly the emergence of ciliated structures, highlights the enhanced regenerative outcome in the large animal model, which may be attributed to improved vascularization and epithelial cell migration supported by the cartilage granule-based scaffold.

(Main text) Please refer to Pars 6, Section 3, as follows:

In line with current clinical understanding, the regeneration of a functional ciliated epithelium remains a formidable challenge in tracheal tissue engineering. Our strategy prioritizes the establishment of a vascularized, mechanically stable graft that can host and protect epithelial progenitor cells during the remodeling phase. In this study, we observed evidence of progressive epithelialization in both rabbit and porcine models following graft implantation (Fig. S12). Immunofluorescence staining for cytokeratin revealed scattered CK+ cells at 2

weeks, with more continuous epithelial-like lining emerging by 8-10 weeks, suggesting a gradual host-driven epithelial repair process. Notably, in the porcine model, focal areas exhibited acetylated tubulin-positive structures, indicating early ciliated differentiation. While these findings highlight the inherent regenerative capacity of the host airway epithelium, the primary focus and strength of our strategy lie in achieving rapid vascularization, mechanical integrity, and stable cartilage ring formation – factors critical for preventing graft collapse and ensuring animal survival. The current graft design provides a vascularized stromal base that can serve as a substrate for future epithelialization strategies, such as the seeding of respiratory epithelial cells or the incorporation of bioactive factors to accelerate ciliated differentiation.

3. Why is angiogenesis important? cartilage formation is better with low oxygen tension. Is the vascular supply for the dorsal fibrous tissue covering? Please explain.

Response: We thank the reviewer for this insightful question regarding the relationship between angiogenesis and cartilage formation. While a hypoxic core is essential for chondrocyte metabolism (*Nat Commun. 2025 Mar 19;16(1): 2711*), graft survival and integration mandate vascularization to prevent necrosis (*J Tissue Eng Regen Med. 2019;13:1815–1829*). In our construct, neo vessels primarily perfuse the peripheral fascial stroma (the "dorsal fibrous covering"), ensuring mechanical integration and viability. Meanwhile, the dense cartilage granule core within minichannels maintains an avascular, hypoxic microenvironment for phenotype stability. We have clarified this crucial distinction in the **Section 3**.

(Main text) Please refer to Pars 2, Section 2.3, as follows:

Notably, these vessels were predominantly localized within the surrounding fibrous tissue and the porous minichannels walls, with minimal infiltration into the dense cartilage granule aggregates themselves. This spatial pattern of vascularization supports the fascial integration while preserving the hypoxic core of the cartilage modules.

(Main text) Please refer to Pars 5, Section 3, as follows:

A key consideration in tracheal graft design is balancing the need for rapid vascularization with the requirement for a hypoxic chondrogenic niche (*Nat Commun. 2025 Mar 19;16(1): 2711; J Tissue Eng Regen Med. 2019 Oct;13(10):1815-1829*). In our approach, the induced angiogenesis primarily serves a structural and protective function: it ensures the viability and robust integration of the vascularized fascia that envelops the graft. This fascial layer is critical for creating an airtight seal at the anastomosis, resisting infection, and providing mechanical support against collapse. Achieving this spatially regulated vascularization is essential for the long-term viability of engineered cartilage constructs, ensuring that the tissue does not undergo necrotic collapse (*J Tissue Eng Regen Med. 2019 Oct;13(10):1815-1829*). Conversely, the cartilage granules confined within the minichannels, remain in a relatively avascular and hypoxic microenvironment that is conducive to chondrocyte metabolism (*Bioact Mater. 2023 Mar 29:27:98-112*). Thus, the newly formed blood vessels were primarily distributed in the fascial stroma and around the minichannels, while the cartilage core was in an avascular state conducive to matrix maintenance, thereby resolving the balance between graft vascularization and cartilage survival.

4. Material characterization of the tracheal constructs vs native trachea would have strengthened the paper.

Response: Thank you for this suggestion. Our study was designed with a function-driven characterization strategy, and we have conducted comprehensive, direct comparisons between the bioengineered grafts and native trachea across multiple critical dimensions:

1. Mechanical Function: The compressive strength at 50% strain – the key metric for collapse resistance – was directly compared, showing grafts achieved strength comparable to (porcine model, Fig. 8F) or exceeding (rabbit model, Fig. 5I) native trachea.

2. Biochemical Composition: Quantitative analyses of glycosaminoglycan, collagen II, and DNA content (Fig. 6E-G) provided a direct comparison of matrix integrity and cell infiltration

3. Vascularization: We have now included immunofluorescence staining and quantification of CD31 for native trachea, demonstrating that the level of vascularization in the long-term CGPA grafts (8-week) approached that of native tissue **(Revised Fig. 5G, 5I, and 6D)**.

These integrated data provide a robust functional comparison, confirming that the engineered grafts recapitulate the essential properties of native trachea and supporting their repair potential.

(Main text) Please refer to Fig 5G, and 5I, as follows:

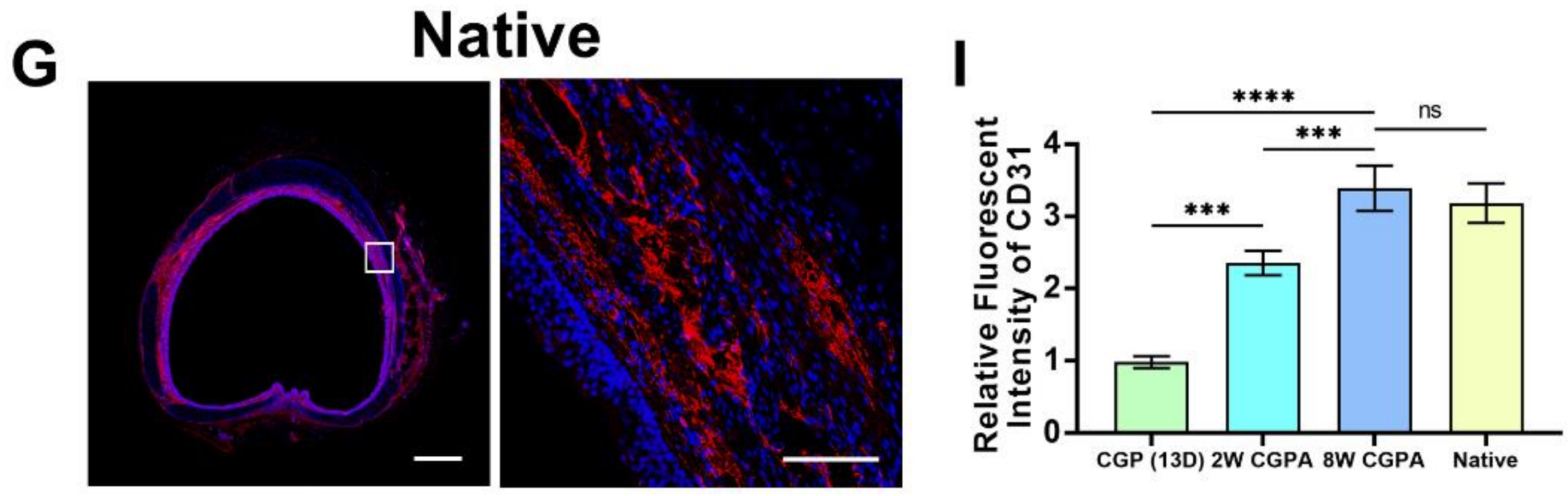

Figure 5. A 3D C-shaped graft was transplanted into a rabbit tracheal defect (2 cm) model. (G) CD31 immunofluorescence staining revealed angiogenesis in the grafts, which was used to visualize the density of blood vessels within the grafts (scale bars: 2 mm and 100 μm). (H) Inner diameter analysis of tracheal grafts from different groups (n=3). (I) Quantitative analysis of CD31 intensity in the different groups of grafts (n=3).

(Main text) Please refer to Pars 3, Section 2.4, as follows:

Notably, the vascularization level in the 8-week CGPA grafts showed no significant difference from the native trachea (3.18 ± 0.27, $p > 0.05$), indicating successful host-driven neovascularization.

(Main text) Please refer to Fig 6D, as follows:

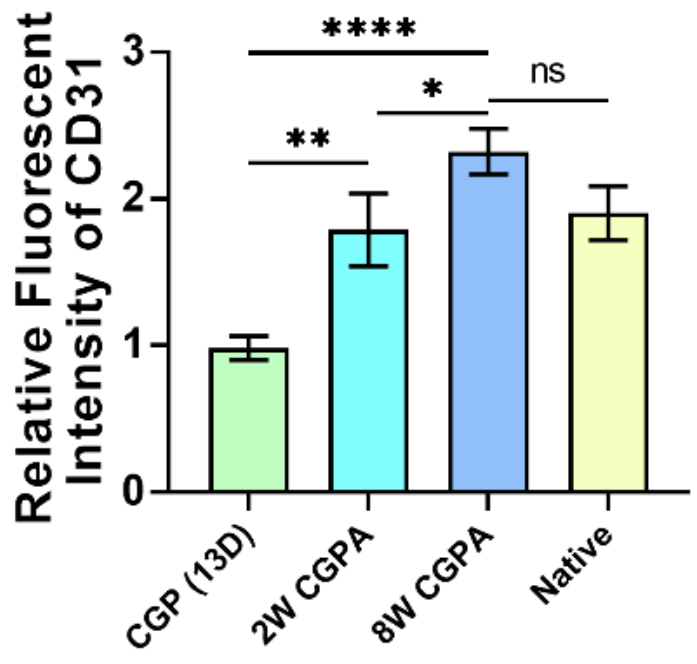

Figure 6. Longitudinal histological analysis of tracheal grafts in rabbits. (D) Quantitative analysis of CD31 intensity in the different groups of grafts (n = 3).

(Main text) Please refer to Pars 4, Section 2.4, as follows:

... CD31 immunofluorescence staining confirmed extensive transmural neovascularization (Fig. 6C), with quantitative analysis showing comparable vascular density between the 8-week CGPA grafts and native trachea (p>0.05) (Fig. 6D).

5. Degradative half life is not defined for the polymers used in the studies. How might longer or shorter half-life affected the outcomes?

Response: Thank you for this insightful comment. In the revised manuscript, we have now explicitly defined the degradation profiles of the polymers used and their functional relevance. The relevant content has been added in **Section 1**.

(Main text) Please refer to Pars 4, Section 1, as follows:

In tracheal tissue engineering, biomaterial selection is crucial for scaffolds that must provide immediate mechanical stability while gradually transferring load to regenerating tissue. Poly(ε-caprolactone) (PCL) offers excellent mechanical strength and a slow degradation profile, with a reported in vivo half-life of approximately 12–24 months, making it suitable for providing sustained, long-term airway support and maintaining lumen patency during the critical remodeling phase (*Polymers (Basel). 2024 Aug 10;16(16):2271; Bioact Mater. 2025 Feb 20:48:313-335; Bioengineering (Basel). 2024 Aug 14;11(8):832.).* In contrast, Poly (glycerol sebacate) (PGS) is an elastomer known for its flexibility and relatively rapid degradation (4-8 weeks *in vivo*), which facilitates early cellular infiltration, vascular ingrowth, and tissue remodeling (*Nat Biotechnol. 2002 Jun;20(6):602-6; Nat Med. 2012 Jul;18(7):1148-53.*). The PCL-PGS composite was designed to combine the strengths of both polymers. PCL provides the necessary mechanical resilience to prevent graft collapse, while PGS enhances biocompatibility, creates transient porosity for host integration, and modulates the overall degradation of the construct. This material system has shown promise in applications requiring a balance of durability and tissue remodeling, such as cardiac patches and vascular grafts (*Nat Med. 2021 Mar;27(3):480-490; Mater Sci Eng C Mater Biol Appl. 2019 Aug:101: 1-14.*).

6. Clinical translation: how do the authors propose a strategy for eventual human use?

Response: We thank the reviewer for this question regarding the clinical pathway of our technology. Our strategy for human translation is built on a simplified, surgery-integrated workflow designed to resolve major clinical bottlenecks: 1) avoiding lengthy in vitro cell

expansion, 2) using readily available autologous tissues (fat, cartilage, blood), and 3) leveraging a pre-vascularization step within the patient's own body (in situ). We have elaborated on this strategy in the **Section 3**.

(Main text) Please refer to Pars 9, Section 3, as follows:

Our approach is designed for clinical translation via a simplified, surgery-integrated pathway that directly addresses key translational bottlenecks. The workflow comprises: (1) concurrent harvest of autologous tissues (rib cartilage, adipose tissue, peripheral blood); (2) rapid, point-of-care processing into cartilage granules, ADSCs, and PRP within one hour, avoiding prolonged ex vivo culture; (3) injection into a patient-specific, sterilized 3D-printed minichannel scaffold; (4) short-term (2-week) in situ prevascularization within a vascularized donor site (e.g., pectoralis fascia); and (5) pedicled transfer to reconstruct the tracheal defect. This strategy leverages established surgical techniques-autologous tissue transfer and flap prefabrication-while minimizing complex tissue engineering steps. Future research efforts will focus on adjusting the size of the graft to match human dimensions and optimizing postoperative care, while integrating epithelial transplantation strategies to accelerate the functionalization of the graft epithelium, thereby meeting the needs of longer-term applications.

7. The authors use cartilage from the ear which is elastic cartilage while the tracheal rings are hyaline cartilage which is a different molecular species, why not use nasal septum instead which is hyaline

Response: We thank the reviewer for raising this point. While it is correct that native tracheal rings are composed of hyaline cartilage, we would like to clarify that the primary functional requirement for a tracheal replacement graft is not the replication of histological cartilage subtype, but the restoration of mechanical stability and patency of the airway conduit. For proof-of-concept studies in rabbits, auricular cartilage was chosen due to its established role in airway reconstruction models (*Sci Transl Med. 2023;15: eabo4272; Nat Commun. 2025 Jul 1;16(1):5734.*), excellent surgical accessibility, adequate tissue yield, and low donor-site morbidity-key practical considerations in preclinical research. Critically, our data demonstrate that the remodeled granule-based construct achieves the primary functional goal: mechanical stabilization of the airway. Furthermore, the platform's adaptability to different cartilage sources, including hyaline cartilage, was successfully demonstrated. We have provided more detailed rationale for source selection and its translational implications in Section 3.

(Main text) Please refer to Pars 2, Section 3, as follows:

The choice of cartilage source was guided by surgical practicality and preclinical modeling considerations. While the native trachea is composed of hyaline cartilage, the primary functional requirement for a tracheal replacement graft is the restoration of mechanical stability and airway patency, not the replication of a specific histological cartilage subtype. In this study, the remodeled granule-based construct successfully fulfilled this mechanical stability. Moreover, the strategy is inherently adaptable to different cartilage sources, as demonstrated by the effective use of hyaline (costal) cartilage in the porcine model. This flexibility underscores the translational strength of our platform, which can leverage the most clinically accessible autologous cartilage source in future applications.

8. What is PGS or CGP, it is not identified anywhere that I could find, PCL is poly caprolactone.

Response: We sincerely thank you for this important suggestion, which has allowed us to enhance the clarity and accessibility of our manuscript. These key terms have now been explicitly defined upon first use. We added relevant descriptions of the polymers used in **Section 1,** and emphasized the abbreviations of the experimental groups in the **Section 2.4**.

(Main text) Please refer to Pars 4, Section 1, as follows:

In tracheal tissue engineering, biomaterial selection is crucial for scaffolds that must provide immediate mechanical stability while gradually transferring load to regenerating tissue. Poly(ε-caprolactone) (PCL) offers excellent mechanical strength and a slow degradation profile, with a reported in vivo half-life of approximately 12–24 months, making it suitable for providing sustained, long-term airway support and maintaining lumen patency during the critical remodeling phase (*Polymers (Basel). 2024 Aug 10;16(16):2271; Bioact Mater. 2025 Feb 20:48:313-335; Bioengineering (Basel). 2024 Aug 14;11(8):832.).* In contrast, Poly (glycerol sebacate) (PGS) is an elastomer known for its flexibility and relatively rapid degradation (4-8 weeks *in vivo*), which facilitates early cellular infiltration, vascular ingrowth, and tissue remodeling (*Nat Biotechnol. 2002 Jun;20(6):602-6; Nat Med. 2012 Jul;18(7):1148-53.*). The PCL-PGS composite was designed to combine the strengths of both polymers. PCL provides the necessary mechanical resilience to prevent graft collapse, while PGS enhances biocompatibility, creates transient porosity for host integration, and modulates the overall degradation of the construct. This material system has shown promise in applications requiring a balance of durability and tissue remodeling, such as cardiac patches and vascular grafts (*Nat Med. 2021 Mar;27(3):480-490; Mater Sci Eng C Mater Biol Appl. 2019 Aug:101: 1-14.*).

(Main text) Please refer to Pars 1, Section 2.4, as follows:

The animals were divided into two groups: the CGP group (grafts composed of Cartilage Granules + PRP, without ADSCs; n = 6) and the CGPA group (grafts composed of Cartilage Granules + PRP + ADSCs; n = 12).

9. What is the size of the cartilage granules?

Response: Thank you for raising this methodological detail. The size of cartilage granules was systematically characterized and optimized, as detailed in **Section 2.2 and Figure 3B-E**. Briefly, the granule size was controlled by cryo-grinding duration. We selected 30 seconds as the optimal grinding time, which produced granules with an average diameter of 288.52 ± 21.11 μm.

10. Pg13 ln 331-333: How do ADSC's promote granule survival? are the granules viable? Not clear from the text. What about immunomodulatory mechanisms of action?

Response: Thank you for your inquiry about the mechanism of ADSC-enhanced granule survival and their viability. We have directly confirmed the viability of cartilage granules through **live/dead staining, alongside histological and biochemical assays**, all of which support the conclusion that the ADSC-enhanced grafts maintain viable cells that are essential for sustained graft function. The dedicated **Section 2.5** and the **Discussion** provide detailed investigation and discussion of the primary mechanism identified in our study: **ADSC-**

mediated metabolic reprogramming (enhanced glycolysis) to support chondrocyte survival under hypoxic stress. We have deleted the indicated text in Section 2.4 to focus on the result description and removed the mechanistic inference from that paragraph. While immunomodulation is a recognized function of MSCs, it was not the focus of our mechanistic investigation in this model.

11. Histology results from figs 5 and 8 do not demonstrate good cartilage formation via Alcian blue staining. The cartilage formation is quite minimal and discontinuous when compared to native cartilage tissue.

Response: We appreciate the reviewer's attention to the histological details. We would like to clarify a key conceptual and methodological aspect of our study: the study objective was not to stimulate new cartilage formation, but to preserve and functionally integrate transplanted adult cartilage granules into a stable, load-bearing structure. The well-preserved Alcian blue staining within individual granules is consistent with our design. We have added a clarification in the **Section 3** to contextualize these histological outcomes.

(Main text) Please refer to Pars 4, Section 3 as follows:

The Alcian blue staining observed in the engineered grafts differ from those of native tracheal cartilage. This is consistent with the biology of adult-derived cartilage, in which chondrocytes exhibit limited synthetic activity. In our model, success is defined not by the generation of large volumes of new cartilage, but by the preservation of the implanted cartilage matrix and its integration into a cohesive, load-bearing structure. The deep staining observed at the matrix of individual granules, together with the biochemical evidence of sustained GAG and collagen II content (Fig. 6F, G), indicates effective matrix maintenance and granule viability. This preserved micro-architecture correlated directly with the graft's ability to achieve mechanical strength comparable to native trachea, fulfilling the essential functional criterion for tracheal reconstruction.

All reviewer points have been addressed point-by-point in the appeal letter, with relevant text, figure, and data integrations in the revised manuscript. We believe these revisions have significantly strengthened the translational potential and experimental support of our study, aligning it with the journal's emphasis on impactful and clinically relevant tissue engineering advances.

We are grateful for the constructive feedback and remain open to further refinements should the manuscript be considered for re-review.

Response to Reviewer #6:

The experimental study entitled “3D minichannels-morphed ‘adipose stem cell–cartilage granule’ reconstituted tough grafts for repairing segmental tracheal defects in preclinical animal models” by Zhang et al. was reviewed. This study represents an enormous amount of animal experimentation aimed at developing an autologous tissue-engineered graft for the repair of segmental tracheal defects. The novelty of this work lies in the use of a 3D minichannel scaffold incorporating adipose-derived stem cells, cartilage granules, and platelet-rich plasma. The engineered graft was first implanted into muscle for two weeks and subsequently used to repair tracheal defects in both rabbit and porcine models.

Response: We sincerely thank you for your thoughtful and constructive evaluation of our revised manuscript. We greatly appreciate your recognition of the extensive preclinical work undertaken in this study, as well as your positive comments regarding the novelty of our 3D minichannel scaffold strategy. Your acknowledgment of the potential value of this approach for segmental tracheal reconstruction is truly encouraging to our team. Thank you again for your valuable suggestions to improve our work.

Comments and Questions

1. The authors demonstrated impressive short-term outcomes up to 8 weeks after tracheal implantation. The graft appeared sufficiently rigid and showed evidence of vascularization within two weeks. The authors should be congratulated for successfully completing these technically demanding experiments.

Response: Thank you very much for your encouraging and positive feedback. We are delighted to know that you appreciate the short-term outcomes of our study, including the mechanical rigidity of the graft and the rapid vascularization achieved within two weeks. Your recognition of the technical demands of these preclinical experiments is especially meaningful to our team.

2. My major concern relates to outcomes beyond 8 weeks. In both rabbit and pig experiments, a considerable number of animals died after this time point. The manuscript would benefit from a clearer explanation of the causes of death. Were these deaths related to airway complications, graft failure, infection, or other systemic issues?

Response: Thank you for raising this important question. All deaths occurring beyond the 8-week time point were attributable to euthanasia following the onset of respiratory symptoms that met our predefined ethical endpoint (Wesley-Croup score ≥10) *(JAMA Otolaryngol Head Neck Surg. 2014 Jan;140(1):66-71.)*, rather than graft failure, infection, or systemic complications.

Respiratory distress results from progressive airway obstruction. This was caused by luminal secretions that accumulated over time, forming viscous "sputum crusts" that gradually narrowed the graft lumen. Even though early epithelialization was observed, the regenerated epithelium at this stage remained functionally insufficient to maintain a moist luminal environment and clear secretions across the segmental defect.

Notably, this complication is entirely preventable in human clinical practice through standard postoperative airway care that cannot be ethically or practically implemented in animal models. In patients, interventions such as temporary tracheostomy, bedside suctioning, nebulized saline for humidification, and bronchoscopic suctioning are routinely available, minimally invasive, and repeatable without general anesthesia. These protocols effectively maintain airway patency during the critical epithelialization period. Unfortunately, the above interventions, such as tracheostomy, bronchoscopic suctioning and nebulization, are not permitted in rabbits or pigs, mainly owing to the lack of perioperative compliance in animals, and repeated general anesthesia for suctioning introduces unacceptable risks of trauma and infection. Although bronchoscopic suctioning under general anesthesia transiently alleviated the obstruction of animals in our study, repeated procedures were not ethically permissible. In spite of only 8 weeks of follow-up in rabbit and pig models, we believethat the mechanically robust and well-vascularized graft would achieve superior long-term outcomes with appropriate clinical care in humans. We have added this clarification to **Section 3**.

(Main text) Please refer to Pars 7, Section 3, as follows:

The limited long-term survival observed in the animal models was primarily attributable to progressive airway obstruction resulting from the accumulation and inspissation of luminal secretions, rather than to graft failure, infection, or systemic complications. Postmortem examinations in both rabbit and porcine subjects revealed no evidence of graft collapse, stenosis, or tissue necrosis, while histopathological analysis of lung tissue demonstrated the absence of

pneumonia or systemic infection (Fig. S16). Despite early epithelialization observed in the grafts, the regenerated epithelium remained functionally immature at this stage, failing to maintain a moist luminal environment or effectively clear secretions across the segmental defect. This functional inadequacy resulted in the progressive accumulation and inspissation of luminal secretions over time. Notably, this complication is entirely preventable in human clinical practice through standard postoperative airway care that cannot be ethically or practically implemented in animal models. In patients, interventions such as temporary tracheostomy, bedside suctioning, nebulized saline to humidify and liquefy secretions, and bronchoscopic suctioning are routinely available, minimally invasive, and repeatable without general anesthesia. In contrast, such measures are not feasible in rabbits or pigs due to the lack of species-appropriate tracheostomy devices, the risks of repeated general anesthesia, and the inability of animals to cooperate with nebulization or positioning. While bronchoscopic suctioning under general anesthesia transiently alleviated obstruction in this study, repeated interventions were not ethically permissible. Given appropriate clinical monitoring and standard postoperative care, the mechanically robust and infection-resistant graft described herein is expected to achieve excellent long-term outcomes in human applications.

3. The macroscopic and endoscopic findings demonstrate a relatively dark appearance of the graft lumen, suggesting possible ischemic change, whereas the external surface appears more pink and well vascularized. Could the authors explain this discrepancy in color and clarify whether this finding reflects insufficient luminal perfusion or delayed mucosalization?

Response: We appreciate your insightful observation of the macroscopic and endoscopic findings, and we apologize for such confusing images in our initial submission. Actually, the relatively dark appearance of the graft lumen in porcine model images was not indicative of ischemia or delayed mucosalization, but rather resulted from our inadvertent use of photographs taken from thawing samples after freezing storage. It is important to note that all grafts in this study underwent two weeks of prevascularization prior to transplantation and were transferred with the vascular pedicle preserved, ensuring sufficient graft perfusion. Our primary intention in providing these images was to demonstrate luminal patency and structural integrity, and we did not anticipate that the use of thawed specimens would create a misleading impression of insufficient luminal perfusion or delayed mucosalization.

To address this concern, we have now provided additional images in **Figure 8E** taken from the same sample immediately upon fresh tissue harvest, demonstrating the true healthy appearance of the graft lumen. Additional images from other porcine samples have also been presented in **Figure S17**, which presented a well-vascularized luminal appearance. We hope these corrections address your concern.

(Main text) Please refer to Section 2.6, Figure 8E as follows:

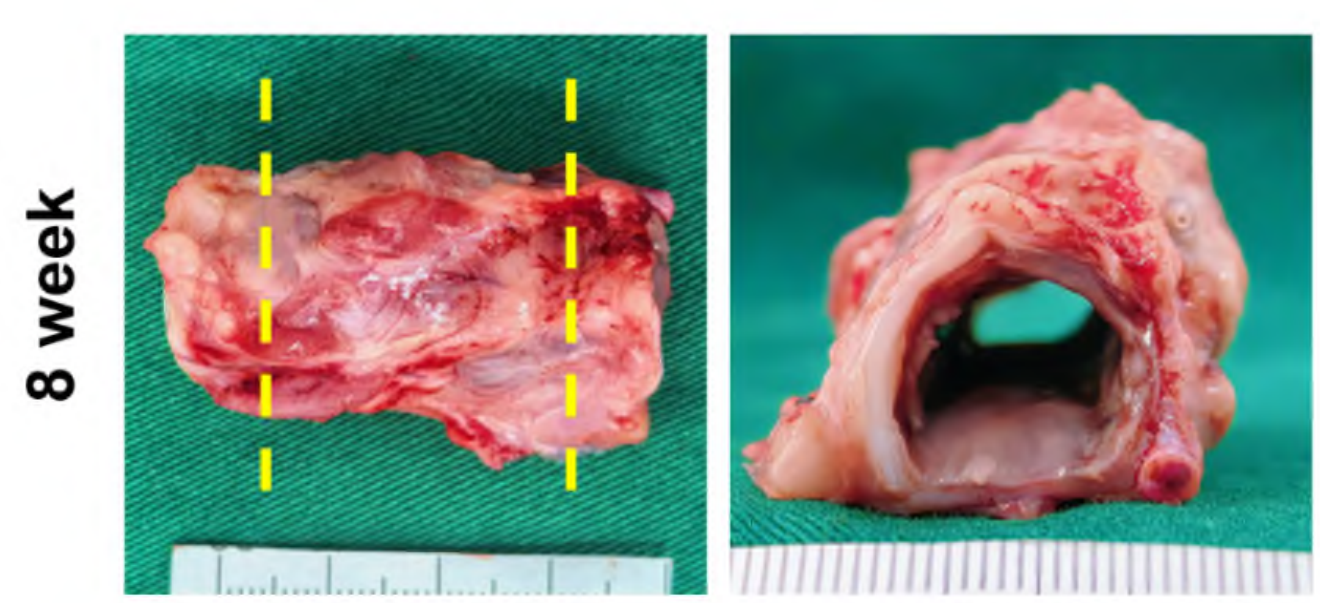

Figure 8. (E) Gross image of tracheal graft at 8 weeks post-implantation, demonstrating graft integration and tissue remodeling.

(Supplementary Materials) Please refer to Figure S14, as follows:

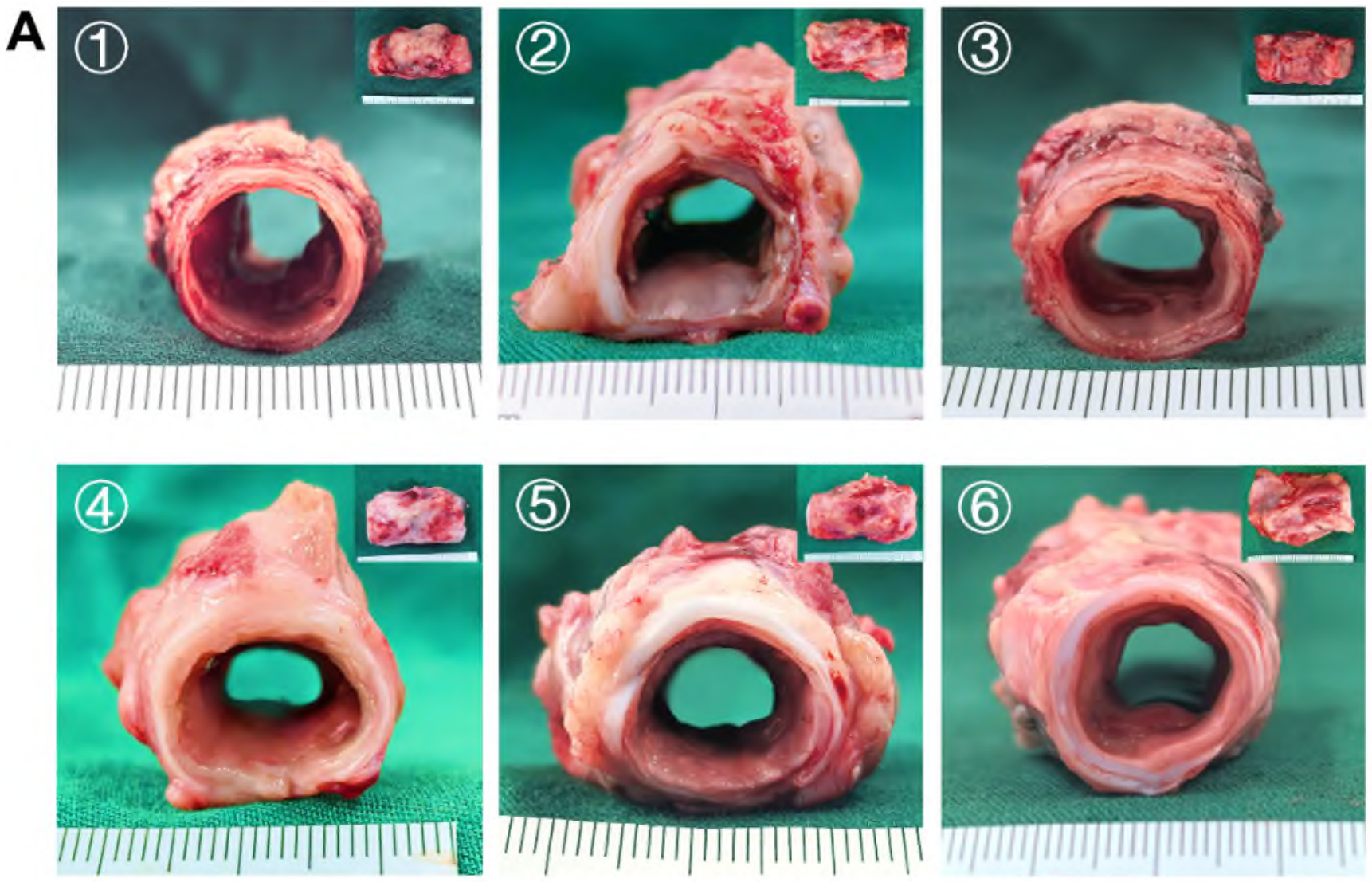

Figure S17. Gross analysis of explanted porcine tracheal grafts. (A) Lumen opening images of grafts harvested from six pigs. ①Tracheal graft harvested at 2 weeks, showing no significant granulation tissue or luminal stenosis in the graft. ②-④ Tracheal graft harvested at 8 weeks. ⑤ Tracheal grafts harvested at 12 weeks (86 days). ⑥ Tracheal grafts harvested at 16 weeks (115 days).

4. As discussed by the authors, the graft does not contain epithelium, which is essential for long-term airway clearance and function. Did the authors observe any migration or regeneration of native tracheal epithelium from the anastomotic edges into the graft during the follow-up period?

Response: Thank you for your question. Yes, we observed clear evidence of progressive epithelial migration from the anastomotic edges into the graft. At 2 weeks, cytokeratin (CK)-positive squamous epithelial cells were detected only at the anastomotic margins, with no positive cells in the quarter or midportion regions. By 8 weeks, the squamous epithelium had extended discontinuously to the quarter points, where occasional acetylated tubulin (Ac-Tub)-positive ciliated cells were also observed. These findings demonstrate a "creeping regeneration" pattern in which host-derived epithelium gradually migrates from the native margins toward the graft center. We have now incorporated these detailed observations into **Section 2.4 and 3** with the

corresponding figures (**Figure S14**).

(Supplementary Materials) Please refer to Figure S14, as follows:

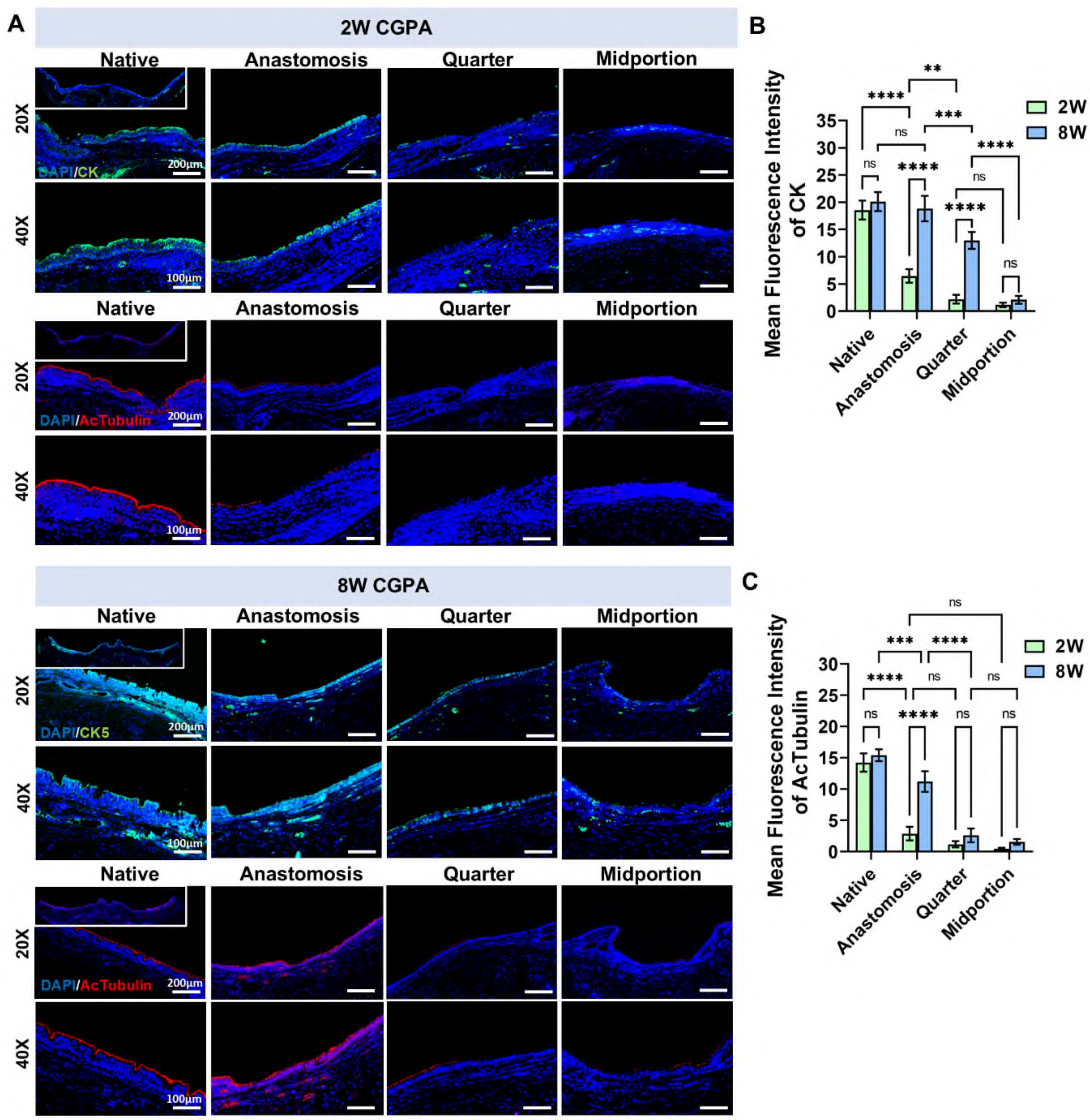

Figure S14. Longitudinal analysis of epithelial regeneration along rabbit tracheal grafts. (A) Immunofluorescence staining for cytokeratin (CK, green) and acetylated tubulin (Ac-Tubulin, red) along the graft axis at 2- and 8-weeks post-implantation (scale bars: 200 μm and 100 μm). (B-C) Quantitative analysis of CK (B) and Ac-Tubulin (C) fluorescence intensity at the anastomosis, quarter, and midportion regions at 2 and 8 weeks (n=4). Fig. S14B, and C significance was

determined by one-way ANOVA followed by Tukey's post hoc analysis. $^{\#}p > 0.05$; $^{*}p < 0.05$, $^{**}p < 0.01$, $^{***}p < 0.001$, $^{****}p < 0.0001$.

(Main text) Please refer to Pars 4, Section 2.4, as follows:

To characterize epithelial regeneration, immunofluorescence staining for CK and Ac-Tub along the graft axis at 2 and 8 weeks was performed (Fig. S14). At 2 weeks, CK-positive squamous epithelial cells were confined to the anastomotic regions (6.47 ± 1.25), with only sporadic positive cells at quarter points (2.21 ± 0.81) and rare scattered cells in the midportion (1.19 ± 0.41) (all vs. native: $p < 0.0001$). Ac-Tub expression was negligible beyond the anastomosis, with only occasional positive cells at the anastomotic margins (2.89 ± 1.09). By 8 weeks, CK-positive coverage had extended discontinuously from the anastomotic regions (18.87 ± 2.33) to the quarter points (12.99 ± 1.55, $p < 0.001$), both significantly increased versus 2 weeks ($p < 0.0001$). However, the midportion showed only sparse CK-positive cells (2.10 ± 0.70), which was not significantly different from 2 weeks ($p > 0.05$). Notably, Ac-Tub-positive ciliated cells appeared more prominently at anastomotic sites (11.21 ± 1.64, vs. 2 weeks: $p < 0.0001$) and extended in a scattered pattern to the quarter points, while the midportion remained negative (1.58 ± 0.45, vs. 2 weeks: $p > 0.05$). These findings confirm a centripetal "creeping regeneration" pattern in which host-derived epithelium progressively migrates from the defect margins toward the graft center over time. However, complete squamous epithelial coverage of the graft lumen was not achieved within the 8-week observation period.

(Main text) Please refer to Pars 6, Section 3, as follows:

Longitudinal assessment of the graft revealed a progressive "creeping regeneration" pattern, with squamous epithelium migrating from the anastomotic margins toward the graft center over time. By 8 weeks, squamous coverage had extended to the quarter points, and scattered ciliated cells had also begun to appear at these sites, indicating ongoing functional differentiation. However, complete squamous epithelial coverage of the graft lumen was not achieved within the observation period. The absence of epithelial coverage in the midportion allowed sputum crusts to accumulate at this site, leading to progressive secretion retention and eventual respiratory symptoms. Notably, this regenerative pattern suggests that with extended postoperative duration

and consistent airway care routinely available in clinical settings, full squamous coverage and subsequent ciliated differentiation could be achieved. Nevertheless, to minimize the window of airway dysfunction and accelerate functional restoration, future research will focus on strategies to promote rapid epithelial regeneration.

We have tried our best to improve the manuscript and made some changes in the manuscript. These changes did not influence the content or framework of the paper. Here, we did not list the changes but marked them in red in the revised manuscript.

We earnestly appreciate your warm work and hope that the corrections will be met with approval.

Once again, thank you very much for your comments and suggestions. Please free to inform me if you remain further concerned, we will revise them according to your further requests.

Response letter by Xinchi Zhang et al titled ***3D minichannels morphed "Adipose stem cell–cartilage granule" reconstituted tough grafts for repairing segmental tracheal defects in preclinical animal models.*** (Manuscript ID: **NCOMMS-25-31353D**)

Page No.

Cover ……………………………………………………………………………………………… 1

Response to Reviewer 2……………………………………………………………………… 2-4

Response to Reviewer 3 ……………………………………………………………………….5-7

Response to Reviewer 6 ………………………………………………………………………8-13

Response to Reviewer #2:

The authors present very interesting data that has the potential for use in replacing the trachea. The defects that require this will be long segment (short segment defects are amenable to resection with excellent expectation of results and interventions similar to those identified here should not be undertaken). Many types of scaffolds will have an excellent appearance even for the first several months. It is the stability of the scaffolds past one year of time that is key. The animals do not survive this time period. There is insufficient data to warrant use in humans at present outside of exceptional circumstances. The limitations of the study and that this data should not be seen as a justification for human use at present and this should be explicitly and unequivocally stated in the text.

Response: We sincerely appreciated the reviewers for the insightful comments. We agree that the defect lengths in our animal models represent a limitation, and longer defects are required in future studies to more closely match clinical needs. We also agree that tracheal grafts need to remain structurally stable for more than one year to be considered successful in the long term, which remains to be evaluated in further studies.

In the revised manuscript, we have corrected relevant descriptions in *Main-text: Abstract, line 23; Introduction, paragraph 4, line 91-93; Results: 2.6, paragraph 4, line 438-440; Discussion, paragraph 10, line 653-654*, and we have addressed the gap between our current results and clinical translation, as was presented in *Main-text: Discussion, paragraph 9, line 638-650.*

Location	Original text	Revised text
Main-text: Abstract, line 23	This study presents a clinically feasible approach for tracheal reconstruction using 3D-printed minichannel scaffolds filled with adipose-derived stem cells (ADSCs) and cartilage granules.	This study presents a tracheal reconstruction approach with clinical potential, using 3D-printed minichannel scaffolds filled with adipose-derived stem cells (ADSCs) and cartilage granules.
Main-text: Introduction, paragraph 4, line 91-93	This method addresses the challenges associated with the limited availability of young chondrocytes, prolonged cell culture during tracheal cartilage preparation, and the difficulty of achieving adequate graft revascularization-indicating promising clinical applications.	This method addresses the challenges associated with the limited availability of young chondrocytes, prolonged cell culture during tracheal cartilage preparation, and the difficulty of achieving adequate graft revascularization, thereby offering key advantages for clinical translation, including reduced ex vivo manipulation, accelerated graft manufacturing, and enhanced potential for host integration and mechanical strength.
Main-text: Results, 2.6, paragraph 4, line 438-440	The successful integration and mechanical stability of the cartilage granule-based 3D minichannel grafts in a large animal model highlight a promising pathway toward clinical application, pending further optimization of epithelial regeneration strategies to mitigate late-phase complications.	The successful integration and mechanical stability of the cartilage granule-based 3D minichannel grafts in a large animal model suggested translational potential, but significant challenges remained, particularly regarding epithelial regeneration and the prevention of late-phase complications.
Main-text: Discussion, paragraph 10, line 653-654	By eliminating dependency on chondrocyte isolation and lengthy in vitro culture, our approach aligns with surgical workflows and addresses critical barriers to clinical adoption. Further refinements in epithelial integration could enable human trials, potentially transforming the management of extensive tracheal defects.	By eliminating dependency on chondrocyte isolation and lengthy in vitro culture, our approach may address some barriers to clinical adoption. Further refinements in epithelial integration could enable human trials, potentially transforming the management of extensive tracheal defects.

(Main-text: Discussion, paragraph 9, line 638-650)

While our 3D minichannel grafts successfully maintained structural integrity and supported animal survival, this study has several notable limitations. First, the observation period was too brief to confirm long-term (>1 year) biomechanical stability, which is essential for preventing late-stage tracheomalacia and airway collapse[3,5,10]. Second, the 3-cm defect in the porcine model did not reach the 50% tracheal length threshold that defines the need for prosthetic substitution in humans[8,43]. Third, ciliary regeneration in the graft's midportion was slow, suggesting the requirement for improved epithelialization strategies. Finally, the PCL-based scaffold slowly degrades *in vivo*, which may restrict radial and longitudinal expansion in growing airways. Replacing partial PCL content with fast degrading materials such as PLA, PLGA, and PGS may match the growth potential of the native trachea[71]. The data presented in our study revealed that this graft could achieve rapid structural integration and mechanical stability in segmental tracheal defects. However, these findings failed to show that the grafts could remain stable over the long term. Further studies in large animal models with longer defects and observation periods are warranted.

Reference:

(3) Martinod, E. *et al.* Feasibility of Bioengineered Tracheal and Bronchial Reconstruction Using Stented Aortic Matrices. *Jama* **319**, doi:10.1001/jama.2018.4653 (2018).

(5) Grillo, H. C. Tracheal replacement: a critical review. *Ann Thorac Surg* **73**, 1995-2004, doi:10.1016/s0003-4975(02)03564-6 (2002).

(8) Ren, J. *et al.* Reconstruction of the trachea and carina: Surgical reconstruction, autologous tissue transplantation, allograft transplantation, and bioengineering. *Thoracic Cancer* **13**, 284-295, doi:10.1111/1759-7714.14315 (2022).

(10) Wei, S. *et al.* Tissue-engineered tracheal implants: Advancements, challenges, and clinical considerations. *Bioengineering & Translational Medicine* **9**, doi:10.1002/btm2.10671 (2024).

(43) Soriano, L. *et al.* Development and clinical translation of tubular constructs for tracheal tissue engineering: a review. *European Respiratory Review* **30**, doi:10.1183/16000617.0154-2021 (2021).

Response to Reviewer #3:

The issue of epithelium is important. We found that free tissue engineered tracheal grafts do re-epithelialize but that this process can take many months depending on the length of graft. Most workers between 2010 and 2020 would stent such grafts, but this compromises epithelialization, so difficult line to tread. Addressing this more fully requires further revision.

Response: We appreciate your professional suggestions. We fully agree that the conflict between providing mechanical stenting and facilitating rapid re-epithelialization is a major bottleneck in tracheal reconstruction. In the revised manuscript, we have discussed this issue in *Main-text: Discussion, paragraph 6, line 573-596.*

(Main-text: Discussion, paragraph 6, line 573-596)

Between 2010 and 2020, most researchers stented tissue-engineered tracheal grafts to ensure early luminal patency. However, stent placement may physically hinder epithelial migration and provoke local inflammation, creating a well-recognized dilemma: adequate patency but compromised re-epithelialization[3,11,43]. In the present study, we attempted to avoid stenting. Nevertheless, some animals developed sputum accumulation and fatal complications, suggesting that without temporary mechanical support, maintaining the patency of free tracheal grafts may be challenging before complete re-epithelialization. Without a stent, there is a risk of sputum stasis and obstruction; with a stent, epithelial quality may suffer, increasing infection, granulation, and restenosis risks[8,14]. It is speculated that tracheostomy or bronchoscopic intervention may be resorted to for relief of the above complications in human cases. However, their feasibility and efficacy would require dedicated evaluation in future large-animal studies with longer follow-up. Addressing the above dilemma may not require a binary choice. First, degradable or removable stents could provide early support and then disappear as epithelialization progresses[53]. Second, bioactive coatings or growth factor delivery might also accelerate epithelial migration and reduce stent dependence[66]. For

instance, our previous work demonstrated that cartilaginous extracellular matrix (cACM) enriched with stem-cell-derived vesicles can actively recruit epithelial progenitors[67]. By optimizing the culturing strategy, such bioactive matrices could be reinforced to provide sufficient mechanical strength while remaining highly bioactive. Admittedly, these strategies need systematic validation in large animal models. We believe that the observed complications did not negate the value of a stent free approach. Rather, they reminded us that before slow epithelialization was adequately addressed, relying solely on stent free grafts for long segment reconstruction may still pose preclinical safety concerns. Future studies may benefit from synergistic optimization of stent design and epithelialization promoting strategies.

Reference:

(3) Martinod, E. et al. Feasibility of Bioengineered Tracheal and Bronchial Reconstruction Using Stented Aortic Matrices. Jama 319, doi:10.1001/jama.2018.4653 (2018).

(8) Ren, J. et al. Reconstruction of the trachea and carina: Surgical reconstruction, autologous tissue transplantation, allograft transplantation, and bioengineering. Thoracic Cancer 13, 284-295, doi:10.1111/1759-7714.14315 (2022).

(11) Mammana, M., Bonis, A., Verzeletti, V., Dell'Amore, A. & Rea, F. Tracheal Tissue Engineering: Principles and State of the Art. Bioengineering 11, doi:10.3390/bioengineering11020198 (2024).

(14) Hiwatashi, S., Iwai, R., Nakayama, Y., Moriwaki, T. & Okuyama, H. Successful tracheal regeneration using biofabricated autologous analogues without artificial supports. Scientific Reports 12, doi:10.1038/s41598-022-24798-y (2022).

(43) Soriano, L. et al. Development and clinical translation of tubular constructs for tracheal tissue engineering: a review. European Respiratory Review 30, doi:10.1183/16000617.0154-2021 (2021).

(53) Liu, C. S. et al. Preparation and evaluation of a silk fibroin–polycaprolactone biodegradable biomimetic tracheal scaffold. Journal of Biomedical Materials Research Part B: Applied Biomaterials 110, 1292-1305, doi:10.1002/jbm.b.35000 (2022).

(66) Chen, Y. et al. Multiplexed self-adaptable Janus hydrogels rescue epithelial malfunction to promote complete trachea repair. Nature Communications 16, doi:10.1038/s41467-025-61135-z (2025).
(67) Zeng, T. et al. Cartilaginous Extracellular Matrix Enriched with Human Gingival Mesenchymal Stem Cells Derived "Matrix Bound Extracellular Vesicles" Enabled Functional Reconstruction of Tracheal Defect. Adv Sci (Weinh) 9, e2102735, doi:10.1002/advs.202102735 (2022).

Response to Reviewer #6:

I appreciate the authors' responses to my comments and questions. I am satisfied with most of the revisions; however, I still have concerns regarding the explanation for the cause of late death.

Tracheal graft stenosis caused by secretion retention should be considered a consequence of graft failure. The authors state that tracheostomy and bronchoscopic interventions would eliminate this complication in human cases, but this remains speculative.

Response: We appreciate your satisfaction with our previous revisions and thank you for thoughtful comments. We accepted your point that secretion retention leading to graft stenosis should be viewed as graft failure under stent-free conditions, not merely a manageable complication. We have added text stating that slow re-epithelialization leads to functional deficits of the tracheal graft.

We also agree that our suggestion regarding tracheostomy or bronchoscopic intervention in humans was speculative. We now have corrected this statement, and stated that such interventions would require dedicated evaluation in future large-animal studies, and clinical extrapolation is premature. These changes have been made and are presented in the table below. (*Main-text: Discussion, paragraph 2, line 487-489; Discussion, paragraph 8, line 620-622; Discussion, paragraph 8, line 631-634; Discussion, paragraph 8, line 635-637; Discussion, paragraph 10, line 653-654*). In addition, we believe that future strategies should focus on degradable or removable stents combined with active epithelialization-promoting approaches. We have added the relevant descriptions in *Main-text: Discussion, paragraph 6, line 573-596* and added a more rigorous perspective on the limitations of our current approach *(Main-text: Discussion, paragraph 9, line 638-650)*.

Location	Original text	Revised text
Main-text: Discussion, paragraph 2, line 487-489	In our research, adverse events reflected secondary pathophysiology: sputum accumulation in epithelium-deficient grafts ultimately led to respiratory failure.	In our research, the observed adverse events revealed a functional limitation in early-stage grafts, where immature mucociliary function hindered secretion clearance, resulting in localized sputum retention and respiratory compromise.
Main-text: Discussion, paragraph 8, line 620-622	The limited long-term survival observed in the animal models was primarily attributable to progressive airway obstruction resulting from the accumulation and inspissation of luminal secretions, rather than to graft failure, infection, or systemic complications.	The limits on long-term survival were primarily attributable to progressive airway obstruction caused by the accumulation of secretions. This highlights a functional limitation of the graft: incomplete mucociliary clearance remains a key challenge in long-segment tracheal reconstruction.
Main-text: Discussion, paragraph 8, line 631-634	Notably, this complication is entirely preventable in human clinical practice through standard postoperative airway care that cannot be ethically or practically implemented in animal models.	In human cases, such complications might be mitigated through standard airway management. However, the inability to implement long-term airway care in this animal model makes accelerating functional epithelial maturation critical.
Main-text: Discussion, paragraph 8, line 635-637	Given appropriate clinical monitoring and standard postoperative care, the mechanically robust and infection-resistant graft described herein is expected to achieve excellent long-term outcomes in human applications.	With appropriate clinical management, this graft shows promise for long-term airway reconstruction; however, extended observation in large animal models is warranted to confirm its long-term stability.

Main-text: Discussion, paragraph 10, line 653-654	By eliminating dependency on chondrocyte isolation and lengthy in vitro culture, our approach aligns with surgical workflows and addresses critical barriers to clinical adoption. Further refinements in epithelial integration could enable human trials, potentially transforming the management of extensive tracheal defects.	By eliminating dependency on chondrocyte isolation and lengthy in vitro culture, our approach may address some barriers to clinical adoption. Further refinements in epithelial integration could enable human trials, potentially transforming the management of extensive tracheal defects.

(Main-text: Discussion, paragraph 6, line 573-596)

Between 2010 and 2020, most researchers stented tissue-engineered tracheal grafts to ensure early luminal patency. However, stent placement may physically hinder epithelial migration and provoke local inflammation, creating a well-recognized dilemma: adequate patency but compromised re-epithelialization[3,11,43]. In the present study, we attempted to avoid stenting. Nevertheless, some animals developed sputum accumulation and fatal complications, suggesting that without temporary mechanical support, maintaining the patency of free tracheal grafts may be challenging before complete re-epithelialization. Without a stent, there is a risk of sputum stasis and obstruction; with a stent, epithelial quality may suffer, increasing infection, granulation, and restenosis risks[8,14]. It is speculated that tracheostomy or bronchoscopic intervention may be resorted to for relief of the above complications in human cases. However, their feasibility and efficacy would require dedicated evaluation in future large-animal studies with longer follow-up. Addressing the above dilemma may not require a binary choice. First, degradable or removable stents could provide early support and then disappear as epithelialization progresses[53]. Second, bioactive coatings or growth factor delivery might also accelerate epithelial migration and reduce stent dependence[66]. For instance, our previous work demonstrated that cartilaginous extracellular matrix (cACM) enriched with stem-cell-derived vesicles can actively recruit epithelial

progenitors[67]. By optimizing the culturing strategy, such bioactive matrices could be reinforced to provide sufficient mechanical strength while remaining highly bioactive. Admittedly, these strategies need systematic validation in large animal models. We believe that the observed complications did not negate the value of a stent free approach. Rather, they reminded us that before slow epithelialization was adequately addressed, relying solely on stent free grafts for long segment reconstruction may still pose preclinical safety concerns. Future studies may benefit from synergistic optimization of stent design and epithelialization promoting strategies.

Reference:

(3) Martinod, E. et al. Feasibility of Bioengineered Tracheal and Bronchial Reconstruction Using Stented Aortic Matrices. Jama 319, doi:10.1001/jama.2018.4653 (2018).

(8) Ren, J. et al. Reconstruction of the trachea and carina: Surgical reconstruction, autologous tissue transplantation, allograft transplantation, and bioengineering. Thoracic Cancer 13, 284-295, doi:10.1111/1759-7714.14315 (2022).

(11) Mammana, M., Bonis, A., Verzeletti, V., Dell'Amore, A. & Rea, F. Tracheal Tissue Engineering: Principles and State of the Art. Bioengineering 11, doi:10.3390/bioengineering11020198 (2024).

(14) Hiwatashi, S., Iwai, R., Nakayama, Y., Moriwaki, T. & Okuyama, H. Successful tracheal regeneration using biofabricated autologous analogues without artificial supports. Scientific Reports 12, doi:10.1038/s41598-022-24798-y (2022).

(43) Soriano, L. et al. Development and clinical translation of tubular constructs for tracheal tissue engineering: a review. European Respiratory Review 30, doi:10.1183/16000617.0154-2021 (2021).

(53) Liu, C. S. et al. Preparation and evaluation of a silk fibroin–polycaprolactone biodegradable biomimetic tracheal scaffold. Journal of Biomedical Materials Research Part B: Applied Biomaterials 110, 1292-1305, doi:10.1002/jbm.b.35000 (2022).

(66) Chen, Y. et al. Multiplexed self-adaptable Janus hydrogels rescue epithelial malfunction to promote complete trachea repair. Nature Communications 16, doi:10.1038/s41467-025-61135-z (2025).

(67) Zeng, T. et al. Cartilaginous Extracellular Matrix Enriched with Human Gingival Mesenchymal Stem Cells Derived "Matrix Bound Extracellular Vesicles" Enabled Functional Reconstruction of Tracheal Defect. Adv Sci (Weinh) 9, e2102735, doi:10.1002/advs.202102735 (2022).

(Main-text: Discussion, paragraph 9, line 638-650)

While our 3D minichannel grafts successfully maintained structural integrity and supported animal survival, this study has several notable limitations. First, the observation period was too brief to confirm long-term (>1 year) biomechanical stability, which is essential for preventing late-stage tracheomalacia and airway collapse[3,5,10]. Second, the 3-cm defect in the porcine model did not reach the 50% tracheal length threshold that defines the need for prosthetic substitution in humans[8,43]. Third, ciliary regeneration in the graft's midportion was slow, suggesting the requirement for improved epithelialization strategies. Finally, the PCL-based scaffold slowly degrades *in vivo*, which may restrict radial and longitudinal expansion in growing airways. Replacing partial PCL content with fast degrading materials such as PLA, PLGA, and PGS may match the growth potential of the native trachea[71]. The data presented in our study revealed that this graft could achieve rapid structural integration and mechanical stability in segmental tracheal defects. However, these findings failed to show that the grafts could remain stable over the long term. Further studies in large animal models with longer defects and observation periods are warranted.

Reference:

(3) Martinod, E. *et al.* Feasibility of Bioengineered Tracheal and Bronchial Reconstruction Using Stented Aortic Matrices. *Jama* **319**, doi:10.1001/jama.2018.4653 (2018).

(5) Grillo, H. C. Tracheal replacement: a critical review. *Ann Thorac Surg* **73**, 1995-2004, doi:10.1016/s0003-4975(02)03564-6 (2002).

(8) Ren, J. *et al.* Reconstruction of the trachea and carina: Surgical reconstruction, autologous tissue transplantation, allograft transplantation, and bioengineering. *Thoracic Cancer* **13**, 284-295, doi:10.1111/1759-7714.14315 (2022).

(10) Wei, S. *et al.* Tissue-engineered tracheal implants: Advancements, challenges, and clinical considerations. *Bioengineering & Translational Medicine* **9**, doi:10.1002/btm2.10671 (2024).

(43) Soriano, L. *et al.* Development and clinical translation of tubular constructs for tracheal tissue engineering: a review. *European Respiratory Review* **30**, doi:10.1183/16000617.0154-2021 (2021).

We have made efforts to address these concerns and have incorporated the necessary revisions throughout the manuscript. These changes did not influence the content or framework of the paper. Here, we did not list the changes but marked them in blue in the revised manuscript.

Once again, thank you very much for your comments and suggestions. Should you have any further concerns, we remain fully committed to refining the manuscript in accordance with your guidance.

Response letter by Xinchi Zhang et al titled ***3D minichannels morphed adipose stem cell-cartilage granule reconstituted tough grafts for repairing segmental tracheal defects.*** (Manuscript ID: **NCOMMS-25-31353E**)

Page No.

Cover ………………………………………………………………………………………… 1

Response to Reviewer 6……………………………………………………………………… 2-3

Response to Reviewer #6:

I am satisfied with the authors' responses to all of my comments and questions. Overall, this small-animal study demonstrates satisfactory short-term outcomes of the tracheal graft. However, the long-term results remain suboptimal because of insufficient epithelial regeneration.

Response: We sincerely thank the reviewer for the positive evaluation of our revised manuscript and for the constructive final comment. We fully agree with the reviewer's insightful assessment that incomplete and slow mucosal re-epithelialization remains a primary functional limitation affecting long-term graft performance. In the revised manuscript, we have further refined the Discussion section *(Main-text: Discussion, paragraph 1, line 504-520; paragraph 9, line 691-704)* to address this issue explicitly and transparently.

(Main-text: Discussion, paragraph 1, line 504-520)

Despite reported clinical successes in cartilage regeneration[23,41,42], replacing segmental tracheal defects remains a major surgical bottleneck, principally because functional graft integration requires systematically overcoming four hurdles: cell and scaffold preparation, mechanical stenting, graft vascularization, and luminal epithelialization. Traditional tissue-engineered constructs, ranging from decellularized allografts to 3D-printed polymeric frameworks, commonly fail because they cannot satisfy these criteria at the same time[7,15,43]. Decellularized matrices suffer from low cell retention and chondrogenic failure, leading to structural collapse, whereas standard 3D-printed scaffolds often delay host vascularization and invite infection due to limited cell-seeding efficiency and transmural porosity[44-46]. In this study, our strategy effectively addresses the first three hurdles. By embedding autologous cryo-ground cartilage granules, PRP, and ADSCs within 3D-printed PCL-PGS minichannels, we eliminate the need for lengthy in vitro cell expansion, deliver immediate mechanical stenting against breathing-induced circumferential compression, and achieve rapid transmural vascularization within two weeks. The fourth hurdle, establishing a mature, ciliated epithelium across the graft center, remains only partially overcome within our 8-week observation window, highlighting where future regenerative interventions must focus.

(Main-text: Discussion, paragraph 9, line 691-704)

While our 3D minichannel grafts successfully maintained structural integrity, several limitations must be noted. Experimentally, the observation period was insufficient to evaluate long-term (> 1 year) biomechanical stability against late-stage tracheomalacia[3,5,10], and the 3-cm defect in our porcine model did not reach the critical

threshold (> 6 cm or 50% tracheal length) typically requiring replacement in human clinical scenarios[8,48]. For direct clinical application, two main issues remain. First, ciliary regeneration across the graft midportion was slow, underscoring the challenge of establishing rapid functional re-epithelialization. Second, the slow degradation of PCL may restrict radial and longitudinal growth, limiting its immediate suitability for growing pediatric airways. To address these limitations, the required next steps include refining scaffold material compositions to match tissue growth[67], integrating bioactive coatings or temporary bioresorbable stents to accelerate ciliary maturation, and evaluating long-segment repairs in large animal models receiving clinical-grade postoperative care.

We have made efforts to address these concerns and have incorporated the necessary revisions throughout the manuscript. All corresponding modifications have been fully incorporated into the revised Discussion and highlighted in red in the manuscript for your convenient review.

Once again, thank you very much for your comments and suggestions. Should you have any further concerns, we remain fully committed to refining the manuscript in accordance with your guidance.

General Comment

This manuscript presents a compelling example of integrating traditional scaffold materials, hydrogels, and regenerative medicine components—specifically, a cartilage granule–PRP–ADSC composite—for tracheal reconstruction. The approach is innovative, and the data generated in both small and large animal models are encouraging. However, several methodological, interpretative, and clarity-related issues should be addressed to enhance the scientific rigour and translational relevance of the study.

Specific Comments

Introduction

1. **Paediatric Growth Compatibility**
   The scaffold does not appear to support growth, which may limit its suitability for congenital cases. Please clarify whether the design targets adults only and consider addressing this in the Discussion or Limitations.

Results

2.2 Rheology and Structural Properties

2. PRP Mixture and Viscosity
   The 5% PRP group is described as having "low viscosity," yet extrusion jams were observed. Low viscosity typically facilitates flow; the issue may instead relate to poor dispersion or granule aggregation. Please clarify and, if available, support with rheological data.

3. Interpretation of BMD Values
   BMD is typically applied to mineralised constructs. Since the scaffold lacks hydroxyapatite, the increased value may simply reflect radiodensity. Please explain its relevance or consider alternate terminology.

2.3 Capillary Infiltration

4. Thickness vs. Porosity
   Rather than thickness alone, reduced porosity likely limits capillary ingrowth. Please clarify if pore interconnectivity was evaluated and considered a contributing factor.

2.4 Rabbit Model

5. Typographical Error
   Please confirm whether "CGP group was superior to CGP and native trachea" should read "CGPA group."

6. Excess GAG and pH Effects
   Higher GAG levels may indicate chondrogenesis but could acidify the microenvironment. Were any pH-related effects observed, or could this be discussed as a potential limitation?

2.6 Pig Model

7. Endpoint Selection
   Adverse events appeared by week 6, yet the study endpoint was set at week 8. Please clarify whether this was predefined or adjusted based on animal condition.

8. Epithelial Migration Evidence
   Both Rabbit and Pig experiments encountered adverse events relating to sputum retention. Was there any indication of epithelial migration onto the scaffold surface, particularly with the fascia pre-coverage? If so, was this ciliated or squamous? If epithelialisation did not occur or occurred and was non-ciliated, then such retention would be expected.

Materials and Methods

9. PCL Longevity and Scaffold Role
   Please specify the scaffold's intended functional duration and discuss whether inflammation might compromise PCL integrity. Has CGPA been tested without the PCL framework?

10. Reagent-Grade PCL
    Was the reagent-grade PCL purified or endotoxin-tested prior to in vivo use?

11. Residual HFIP
    Has residual solvent been assessed post-fabrication? Data (e.g., FTIR, TGA) would help confirm safety.

12. Cryo-Grinding Protocol
    Were cryoprotectants used during cartilage grinding? Including this detail would aid reproducibility.

13. GMP/Quality Standards
    Were any GMP or equivalent standards applied to fabrication or sterilization? This would speed regulatory approvals for clinical trials.

Supplementary

14. CCK-8 Volume Discrepancy
    Using 1 mL in a 96-well plate seems inconsistent with well capacity. Please clarify.

15. Adverse Events Interpretation
    Since epithelial regeneration was limited, could the complications also involve scaffold inflammation or remodelling issues? The latter is a major issue with previous attempts at tracheal scaffold production (e.g. Eur Respir J. 2020 Jun 18;55(6):1901200). Clarifying this would improve interpretation.

16. Inflammatory Markers
    Were cytokines such as IL-1β, TNF-α, or IL-6 assessed to evaluate immune response? This could help differentiate structural from immunological failure.

Reading Note

CGPA: CG (cartilage granular) - ADSc (Allograft) - PRP (host)

CGP: control, without ADSc

CG – Cryo grinding in $N_2(l)$: 30s, rabbit ear(host); 20s, pig rib (host)

PRP: gel as a carrier (host)

ADSc: allograft in rabbit; autograft in Bama minipigs

With ADSc, well-chondrocyte growth, well vascularisation established

Without ADSc, Necrotic chondrocytes, and less vascularisation are found

PCL – PGS (polycaprolactone - poly glycerol sebacate) composite coating on caramel-based sacrifice mould. After removing the caramel, the CGP/CGPA hydrogel is injected into the micro tunnel left by the caramel.

PCL: 2-4 years degradation

PGS: 2 weeks to 60 days degradation

No degradation test results

Harvest at the 2nd and 8th week after implanting onto the airway.

Scaffold underwent 2 weeks of remodelling in the rabbit platysma/bam pig subcutaneous layer to prevascularise the scaffold by vascularised fascia before implanting it onto the airway.

Introduction

While the Introduction references congenital airway disorders as part of the clinical context, the scaffold described in this study does not appear to possess any growth accommodation features. For pediatric or congenital stenosis cases, long-term success would require the implant to adapt to somatic growth. The authors may wish to clarify whether their design is intended exclusively for adult or post-growth-stage applications, or to address this limitation in the Discussion section.

Result

2.2.

"The 20% PRP mixture exhibited high fluidity, with significant discrepancies between the printed image and the original image, achieving an accuracy of only 43.00±3.00%. In contrast, the 5%PRP mixture, where cartilage granules predominated, displayed low viscosity, resulting in extrusion jams and other complications"

The authors describe the 5% PRP mixture—where cartilage granules predominated—as having "low viscosity," which in turn led to extrusion jams. However, viscosity refers to a material's resistance to flow, and low viscosity typically facilitates extrusion rather than causing clogging. Given the dominance of cartilage granules in this formulation, one would expect increased viscosity or complex non-Newtonian behavior, particularly if the granules were not well-dispersed.

It may be more accurate to attribute the extrusion jams to poor homogeneity or phase separation, rather than low viscosity per se. The authors are encouraged to clarify the basis of this viscosity assessment and whether any rheological measurements were performed to support this interpretation.

"Mechanical testing and micro-CT analysis revealed significant improvements in scaffold properties post-granule incorporation (red indicating higher density, green indicating lower density) (Fig. 3M, N). The bone mineral density (BMD) increased from 39.87 ± 2.04 mg/cm² (Pre-filling) to 71.37 ± 2.84 mg/cm² (Post-filling), with a corresponding increase in compressive strength from 0.63 ± 0.02 N to 1.33 ± 0.03 N for 50% structural collapse (Fig. 3O). "

The manuscript reports an increase in bone mineral density (BMD) as measured by micro-CT following post-filling with the hydrogel-cartilage granule mixture. However, BMD analysis in micro-CT is typically calibrated against hydroxyapatite standards and is most meaningful when evaluating mineralized tissues or constructs containing mineral phases.

Given that the composite material does not appear to contain hydroxyapatite or other mineralized components, could the authors clarify the relevance and interpretation of the BMD data in this context? Is the observed increase simply reflective of higher radiodensity due to space-filling by the hydrogel and granules, rather than true mineral content? Additional explanation or reframing of the term "BMD" might be helpful to avoid misinterpretation.

2.3 previsualisation

1. “Additionally, coating thickness could hinder the transmural growth of capillaries, thereby affecting the survival and integration of cartilage granules within the minichannels. “

The authors suggest that increased coating thickness may hinder capillary transmural growth and thereby impair the survival and integration of cartilage granules. However, it may be worth distinguishing whether the critical factor is the absolute thickness of the coating or the resultant decrease in porosity that accompanies increased thickness.

From a biomaterial’s perspective, reduced pore interconnectivity and diffusion pathways due to densification are more directly associated with impaired vascular ingrowth. The authors are encouraged to clarify whether porosity or structural occlusion, rather than thickness per se, may be the dominant factor impeding capillary penetration and cell integration within the scaffold.

2.4 rabbit, cartilage from the ear

1. “Consistent with the satisfactory histological results, the mechanical strength of the CGP group was superior to that of both the CGP group and the native trachea.”

It appears that the group designation may have been misstated. Should this be “CGPA group” instead of the first “CGP group”? Kindly confirm and revise if this was a typographical error.

2. The data indicate that the CGPA group exhibited higher T2 collagen and GAG content compared to native tracheal tissue. While this may reflect enhanced chondrogenesis, could the authors comment on whether excessive GAG accumulation might create an overly acidic microenvironment, particularly in the context of avascular or poorly perfused constructs?

As GAG biosynthesis during chondrogenic differentiation is known to be associated with acidic byproducts, it would be helpful to know whether any pH-related effects (e.g., tissue necrosis, matrix degradation, or inflammatory responses) were observed, especially in vivo. If not measured, the authors may wish to discuss this as a potential consideration for long-term scaffold integration.

2.6 Pig, cartilage from rib

The authors report that clinical manifestations of respiratory dysfunction—including reduced feeding and weight loss—became evident at 6 weeks post-implantation, necessitating euthanasia of all remaining animals at day 57. Given this timeline, could the authors clarify whether the 8-week endpoint was selected based on preliminary survival data or predefined as the intended study duration? Considering that significant adverse events were already apparent by week 6, further justification of the endpoint selection would help contextualise the relevance and limitations of the model.

Additionally, given the importance of epithelial coverage in restoring mucociliary function, is there any histological or gross evidence of epithelial migration from the native tissue margins toward the scaffold surface, particularly this scaffold is covered by the vascularized fascia before implantation? This information could provide mechanistic insights into the observed failure of epithelial integration and help guide future design iterations.

Limitation

"PGS-PCL walls may compromise the mechanical strength of grafts remains to be determined after long-term survival is achieved."

Given that polycaprolactone (PCL) is known to degrade over several years, could the authors clarify the intended functional duration of the CGPA–PCL–PGS composite scaffold prior to significant degradation? In particular, under inflammatory conditions—as partially observed in the CGP group—would the structural integrity or retention of the scaffold be compromised before the intended functional timeline is reached?

If the authors plan to extend this model to longer-term in vivo studies, further clarification on how the composite responds to prolonged inflammatory stimuli (e.g., degradation rate, mechanical retention at the site) would be valuable.

Additionally, has the CGPA formulation been tested independently—without the PCL scaffold—as a standalone injectable or filler? Such data, if available, could help distinguish the specific role of the PCL framework in structural support versus the bioactivity of the cartilage granule–PRP phase alone.

Material

1. Use of Reagent-Grade PCL and Potential Inflammatory Response
The manuscript specifies the use of reagent-grade polycaprolactone (PCL). Could the prolonged inflammatory response observed be partially attributed to the use of non-medical grade PCL? Please clarify whether any additional purification or endotoxin testing was performed to ensure its suitability for biological applications, particularly in vivo.

2. Residual Solvent: Hexafluoroisopropanol (HFIP)
Given the use of hexafluoroisopropanol (HFIP) during scaffold preparation, have the authors assessed the extent of solvent evaporation post-fabrication? Although HFIP is commonly used in electrospinning and scaffold processing, residual solvent trapped in highly porous structures can be cytotoxic and may elicit local inflammation in vivo. Please consider including any data (e.g., FTIR, TGA, or drying protocols) that support complete solvent removal.

3. Cryogenic Grinding of Cartilage
The methodology describes cryo-grinding of cartilage tissue after immersion in liquid nitrogen for 3 minutes. Although the final in vitro results appear promising, it would be helpful if the authors could briefly comment on whether any cryoprotective measures were considered during cryo-grinding. This information could be valuable for reproducibility and for readers aiming to replicate the protocol in different settings.

4. Compliance with GMP or Quality Standards in Scaffold Preparation
Given the translational potential of the scaffold described, did the authors follow any aspects of Good Manufacturing Practice (GMP) or equivalent quality control guidelines during fabrication, sterilisation, or material handling, at least before the revascularisation step? This information would strengthen the translational relevance of the study, especially for in vivo or preclinical applications.

Supplement:

1. CCK-8 Assay Volume Discrepancy

In the methods section, the authors state that 1 mL of CCK-8 reagent was added in the 96-well culture. Please clarify this point, as standard 96-well plates typically accommodate ~100–200 µL per well. A volume of 1 mL would exceed the well capacity, suggesting either a typographical error or a different experimental setup.

2. Adverse Events and Survival Outcomes – Supplementary S8, Table S1 and S2

The authors have acknowledged the lack of functional epithelial regeneration in CGPA group as a limitation. In this context, it would be valuable to clarify whether the adverse events (e.g., sputum retention persisting in 1/3 of animals at week 8) are primarily due to this epithelial deficit or whether other factors, such as scaffold-induced inflammation or incomplete remodelling, might also contribute.

Furthermore, since some complications began to emerge from week 6, do the authors believe that the 8-week endpoint remains the most appropriate for assessing scaffold performance? This clarification may guide future studies seeking to extend functional durability.

3. Inflammatory Status – Cytokine Assessment

Given that the experiment concludes at week 8—still within the commonly accepted acute rejection period for xenotransplantation or tissue-engineered implants—did the authors assess any inflammatory cytokines (e.g., IL-1β, TNF-α, IL-6) in local tissue or systemic circulation? Such data would help distinguish between failure due to structural inadequacy versus immune-mediated complications, especially in the CGP group with higher adverse event rates.
