# Supplementary material for: 3D minichannel-morphed adipose stem cell-cartilage granule reconstituted tough grafts for repairing segmental tracheal defects in preclinical models
Source: Nat Commun. 2026 Sep 11;17:10735. doi: 10.1038/s41467-026-77738-z (PMC13658815; doi:10.1038/s41467-026-77738-z)
Supplement: Supplementary file 3 [file 41467_2026_77738_MOESM1_ESM.pdf]

Supplementary Materials

3D minichannel-morphed adipose stem cell-cartilage granule reconstituted tough grafts for repairing segmental tracheal defects in preclinical models

Xinchi Zhang[1, 2†], Na Wei[1†], Bing Liu[3†], Pingping Yuan[1], Xiaopeng Wu[1], Zheqian Zhang[1], Yidong Hu[1], Pengyu Wang[1], Jingyi Zhang[2], Yimin Zhao[2], Wei Wu[1*]

Page No.

Cover………………………………………………………………………………… 1

Part I. Supplementary Fig. 1-17………………………………………………… 2-19

Part II. Supplementary Table 1 ……………………………………………….. 20

Part I: Supplementary Fig. 1-17

Supplementary Fig. 1 Demonstration of the plasticity of caramel scaffolds.

Supplementary Fig. 2 FTIR spectra of the PCL-PGS scaffold.

Supplementary Fig. 3 Endotoxin test results.

Supplementary Fig. 4 Fabrication process of cartilage granule-based 3D minichannel grafts.

Supplementary Fig. 5 Analysis of cell viability in cartilage granules treated with cryopreservation solution

Supplementary Fig. 6 Multilineage Differentiation of ADSCs.

Supplementary Fig. 7 Tubulogenesis assay and quantitative analysis.

Supplementary Fig. 8 Histological evaluation of aggregates with varying ratios of ADSCs to cartilage granules in nude mice.

Supplementary Fig. 9 Rheological properties of PRP-cartilage granule mixtures.

Supplementary Fig. 10 Characterization of scaffold-free CGPA grafts after 2 weeks of prevascularization.

Supplementary Fig. 11 Display of the compressive stress resistance of tracheal grafts with various coating layers.

Supplementary Fig. 12 Epithelial marker expression in tracheal grafts.

Supplementary Fig. 13 Inflammation marker expression in different tracheal grafts.

Supplementary Fig. 14 Longitudinal analysis of epithelial regeneration along tracheal grafts.

Supplementary Fig. 15 Safety assessment of 3D cartilage ring grafts.

Supplementary Fig. 16 Characterization of porcine rib cartilage microparticles.

Supplementary Fig. 17 Gross analysis of explanted porcine tracheal grafts.

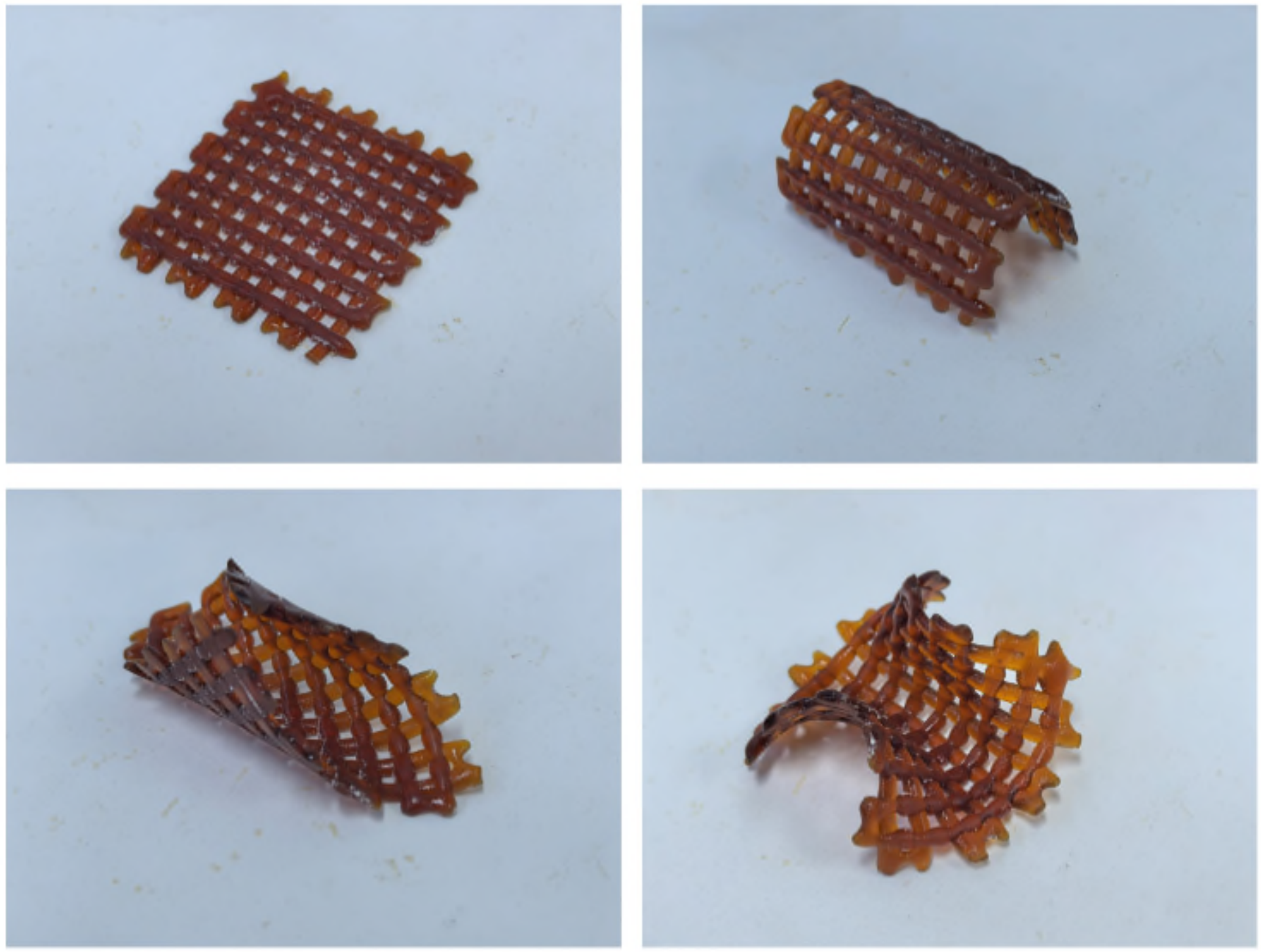

Supplementary Fig. 1. Demonstration of the plasticity of caramel scaffolds. Caramel scaffolds can be morphed into different shapes without compromising structural integrity, demonstrating their adaptability for customized tissue engineering and regenerative medicine applications.

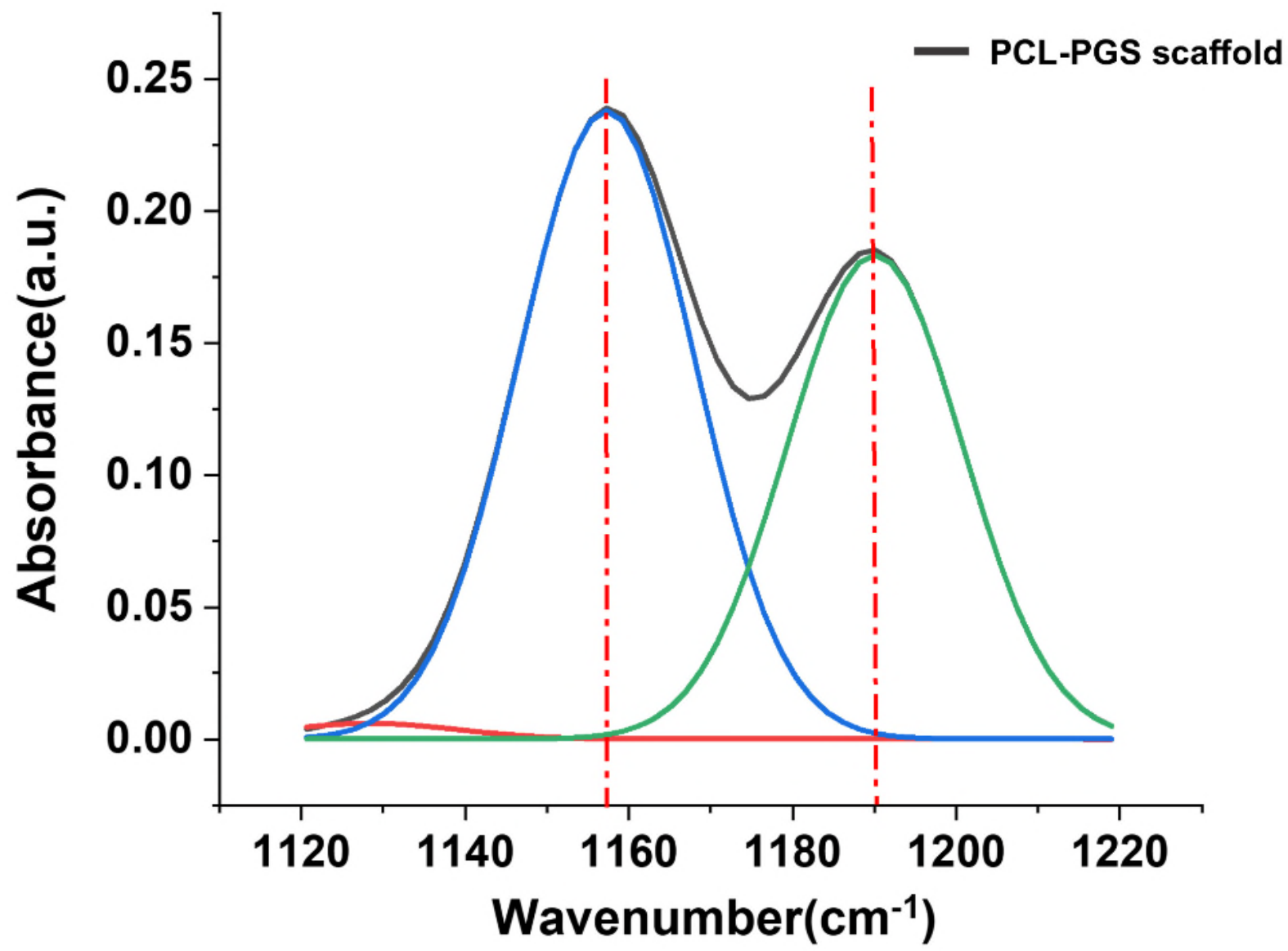

Supplementary Fig. 2 FTIR spectra of the PCL-PGS scaffold. FTIR spectra confirming the absence of residual HFIP in the scaffolds (gray). After deconvolution, characteristic PCL (1157.3 cm^{-1}, blue) and PGS (1190.1 cm^{-1}, green) peaks are labeled. HFIP control results in C–F bonds at 1218 cm^{-1}, which are absent in the scaffolds.

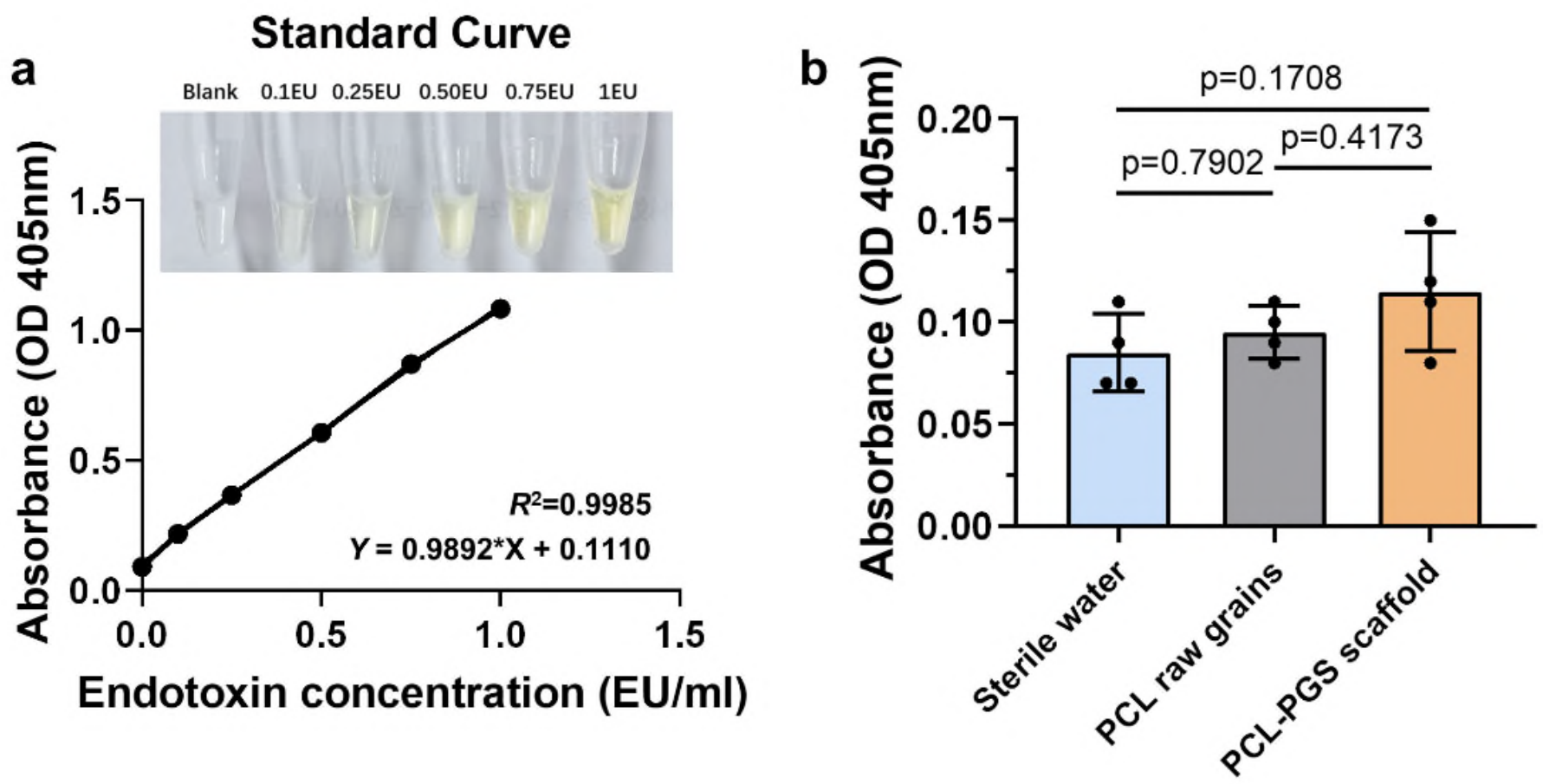

Supplementary Fig. 3 Endotoxin test results. a Endotoxin detection standard curve. **b** Endotoxin levels in sterile water, PCL raw material, and PCL-PGS minichannels (n = 4 independent biological samples). Data in b are presented as mean values ± SD. Statistical significance was determined via two-sided one-way analysis of variance (ANOVA) followed by Tukey's post-hoc test. Source data are provided in Source Data file.

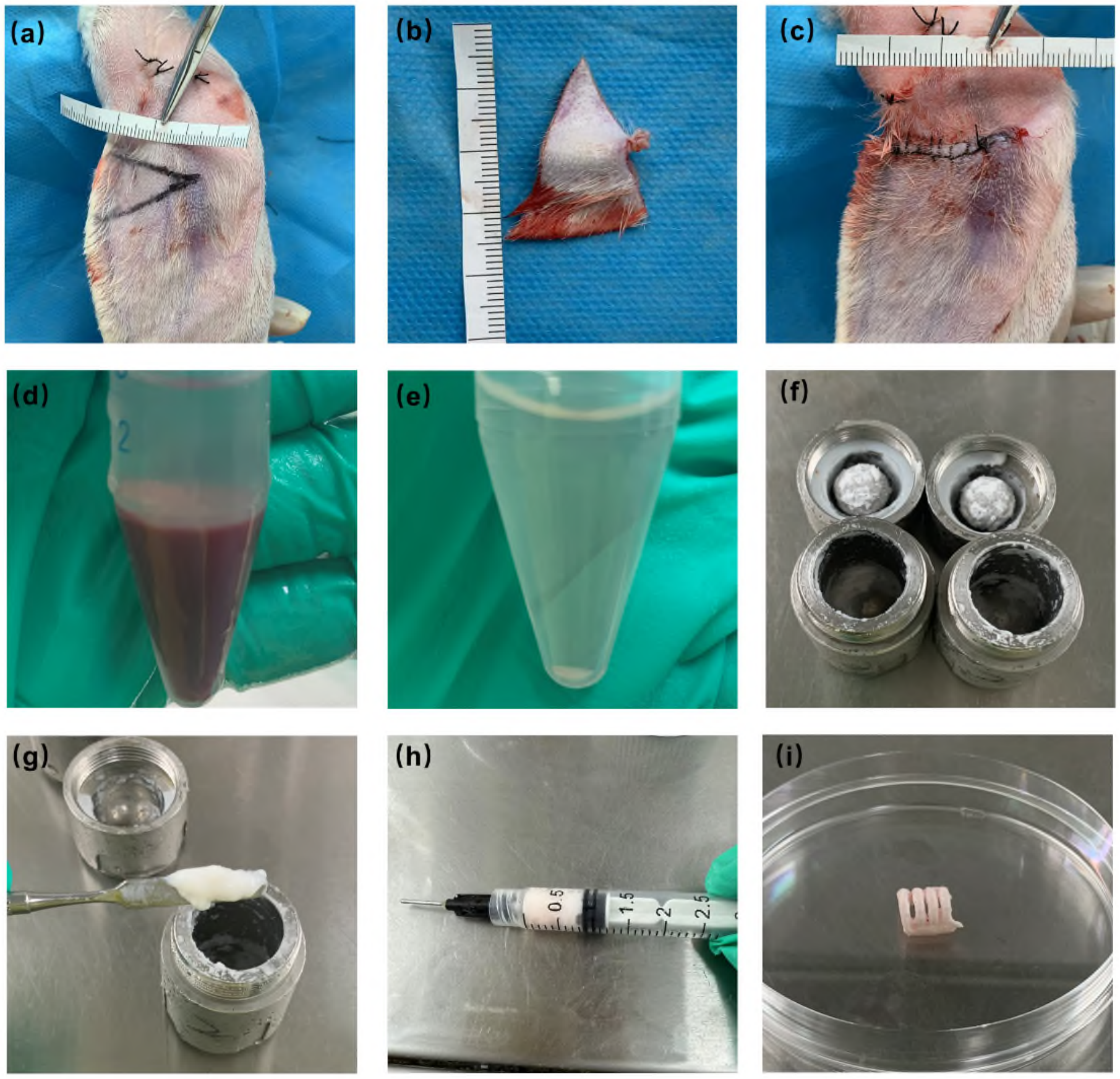

Supplementary Fig. 4 Fabrication process of cartilage granule-based 3D minichannel grafts. (a-c) Harvesting of auricular cartilage tissue (4 cm^2). (d-e) Autologous PRP extraction. (f-h) Preparation of injectable cartilage granules: grinding and formulation of cartilage granules; mixing of cartilage granules, PRP, and ADSCs. (i) Gross appearance of the C-shaped cartilage ring graft.

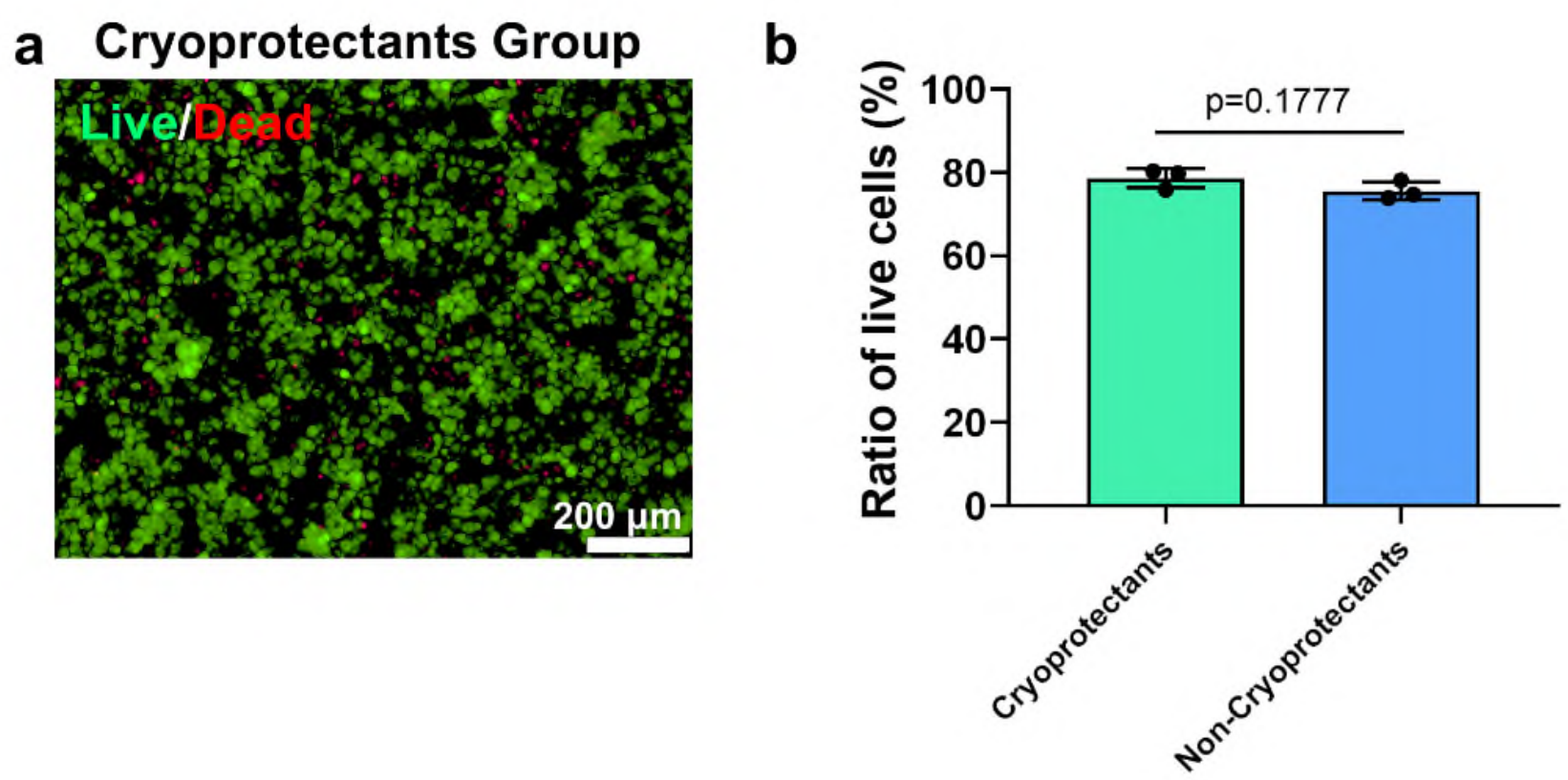

Supplementary Fig. 5 Analysis of cell viability in cartilage granules treated with cryopreservation solution. a Live/Dead cell staining of cartilage particles under cryopreservation conditions (n = 3 independent biological experiments with similar results; scale bar: 200 μm). **b** Quantitative analysis of cell survival between groups with and without cryopreservation solution (n = 3 independent biological samples). Data in b are presented as mean values ± SD. Statistical significance was determined v via a two-tailed unpaired Student's *t*-test. Source data are provided in Source Data file.

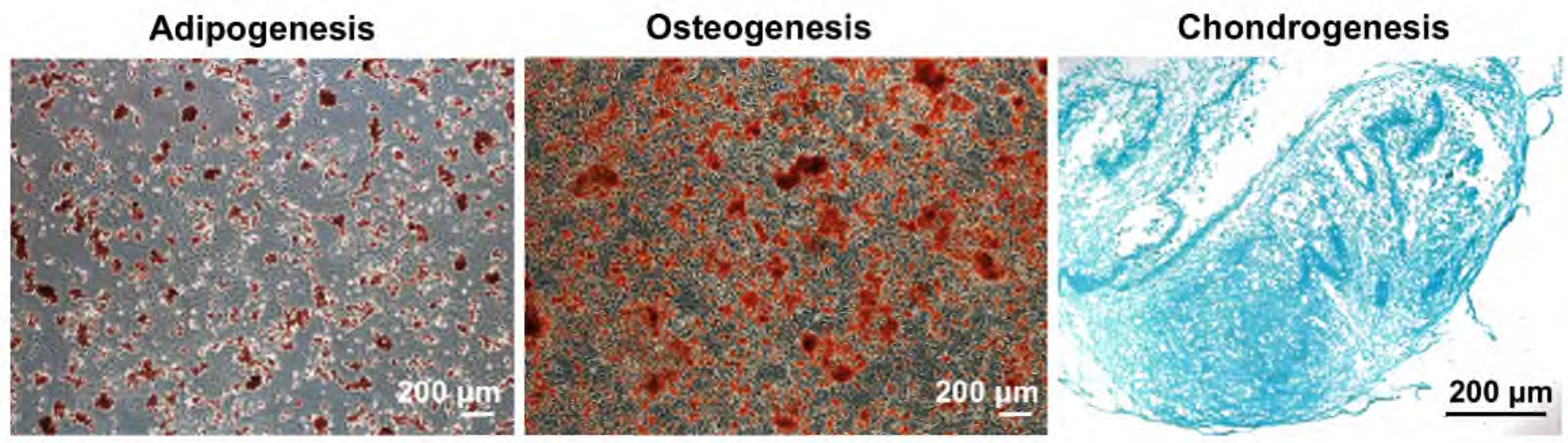

Supplementary Fig. 6 Multilineage Differentiation of ADSCs. The results collectively confirmed the multilineage differentiation potential of ADSCs, thereby verifying their stemness and mesenchymal origin (n = 3 independent biological experiments with similar results; scale bar: 200 μm).

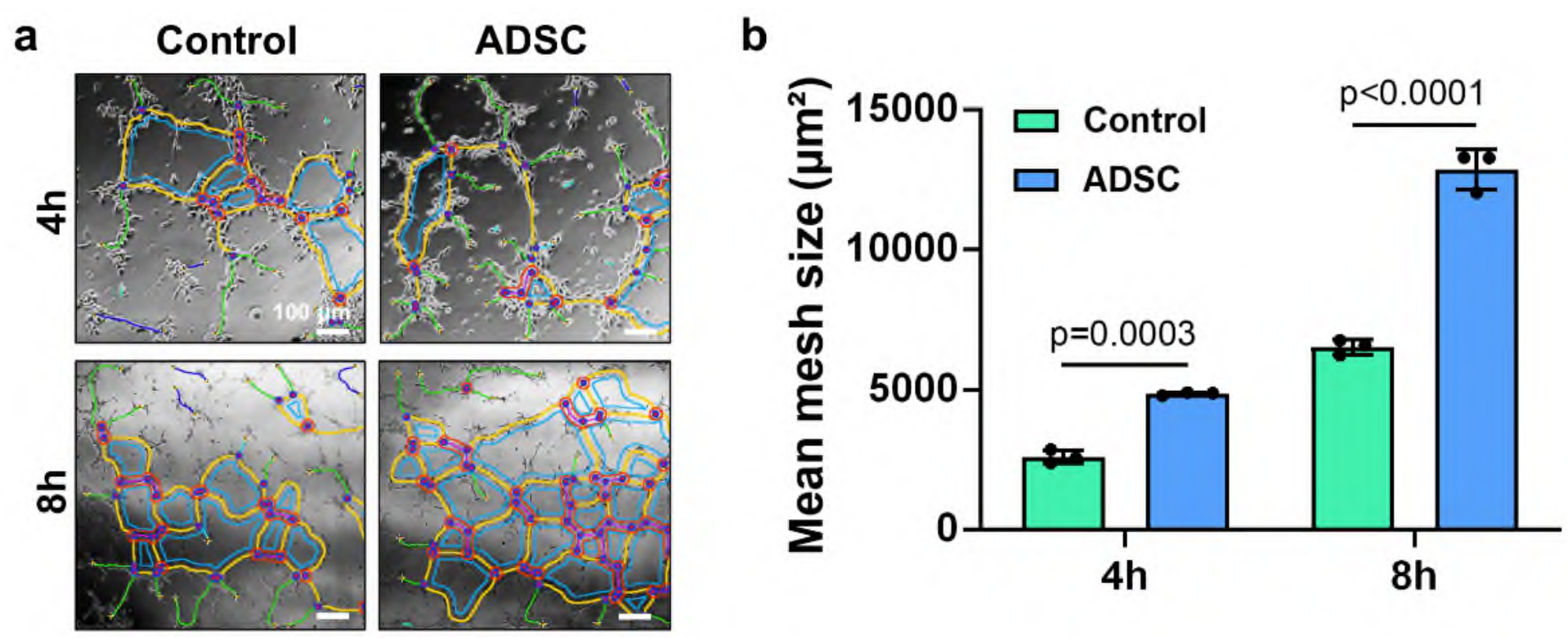

Supplementary Fig. 7 Tubulogenesis assay and quantitative analysis. a Representative image of tubulogenesis in the control and ADSC (co-cultured with ADSC) groups at 4 and 8 hours (n = 3 independent experiments with similar results; scale bar: 100 μm). **b** Quantitative analysis of tubule formation (n = 3 independent biological samples). Data in b are presented as mean values ± SD. Statistical significance was determined via two-sided one-way analysis of variance (ANOVA) followed by Tukey's post-hoc test. Source data are provided in Source Data file.

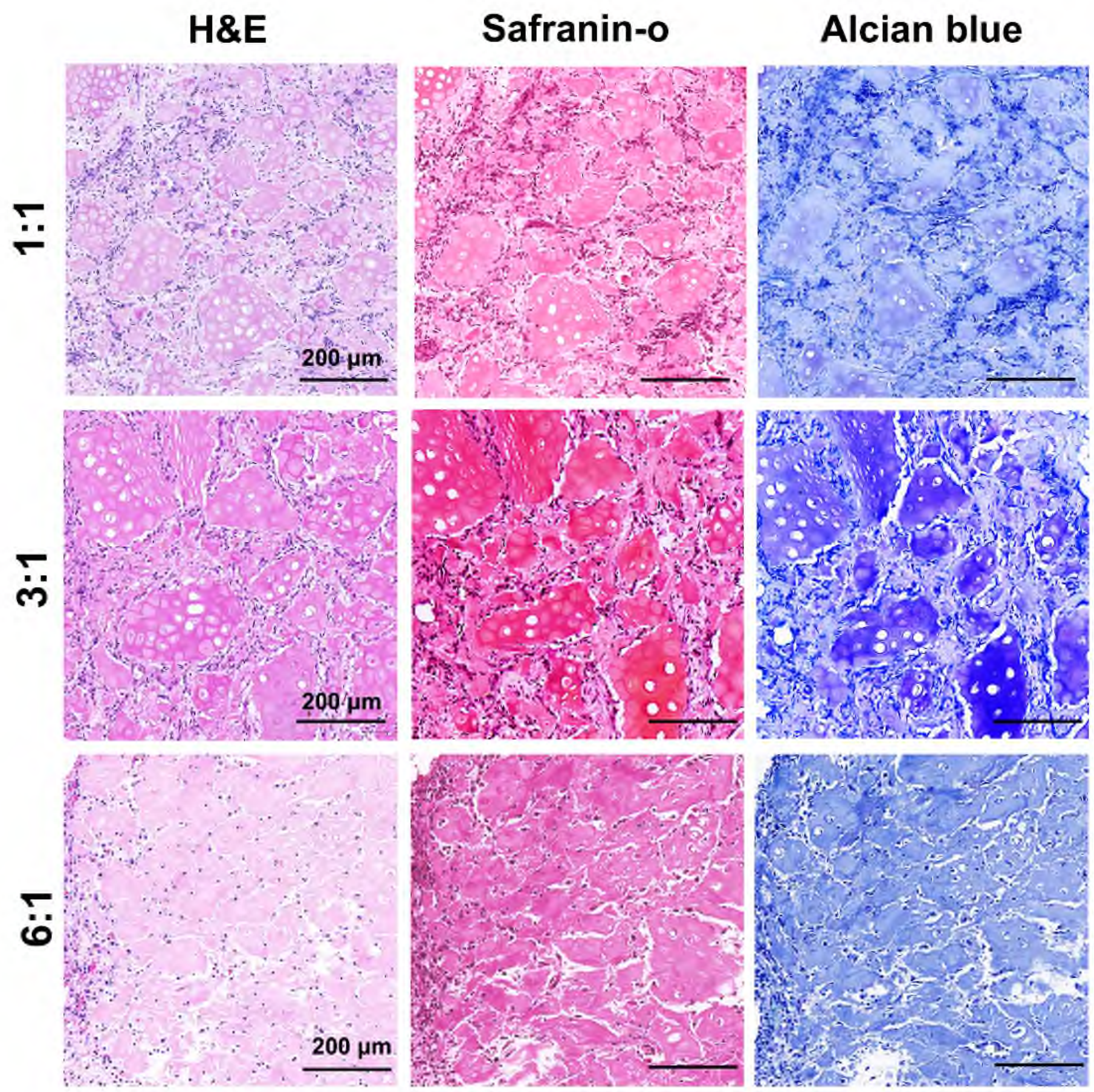

Supplementary Fig. 8 Histological evaluation of aggregates with varying ratios of ADSCs to cartilage granules in nude mice. H&E, Safranin-O, and Alcian blue staining of the implanted aggregates revealed tissue integration and matrix deposition at three ratios (1:1, 3:1, and 6:1; n = 3 independent experiments with similar results; scale bar: 200 μm).

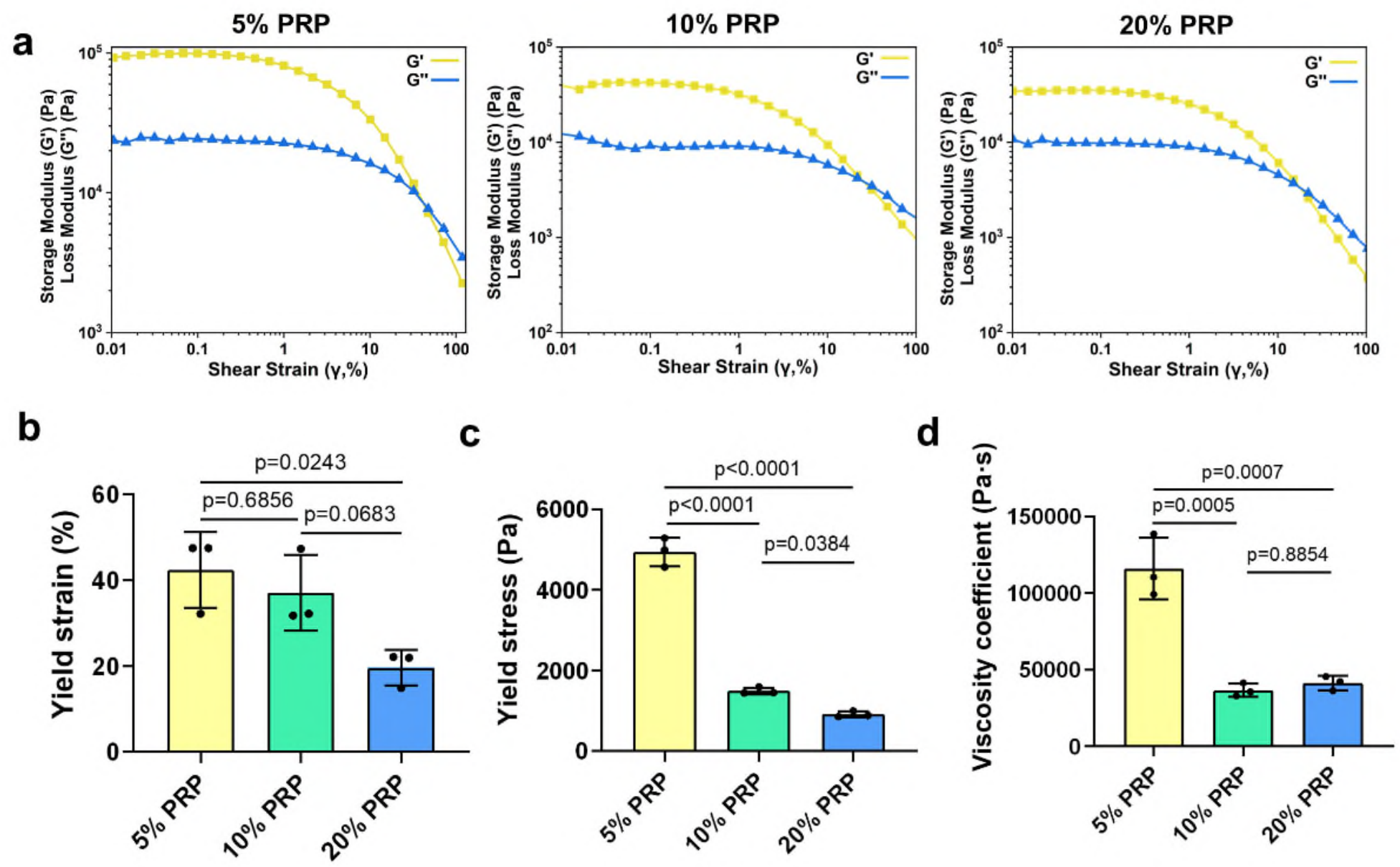

Supplementary Fig. 9. Rheological properties of PRP-cartilage granule mixtures. **a** Storage (*G'*) and loss (*G''*) moduli vs. shear strain for hydrogels with 5%, 10%, and 20% PRP. **b** Yield strain, **c** yield stress, **d** viscosity coefficient across different PRP concentrations (n = 3 independent samples). Data in b-d are presented as mean values ± SD. Statistical significance was determined via two-sided one-way analysis of variance (ANOVA) followed by Tukey's post-hoc test. Source data are provided in Source Data file.

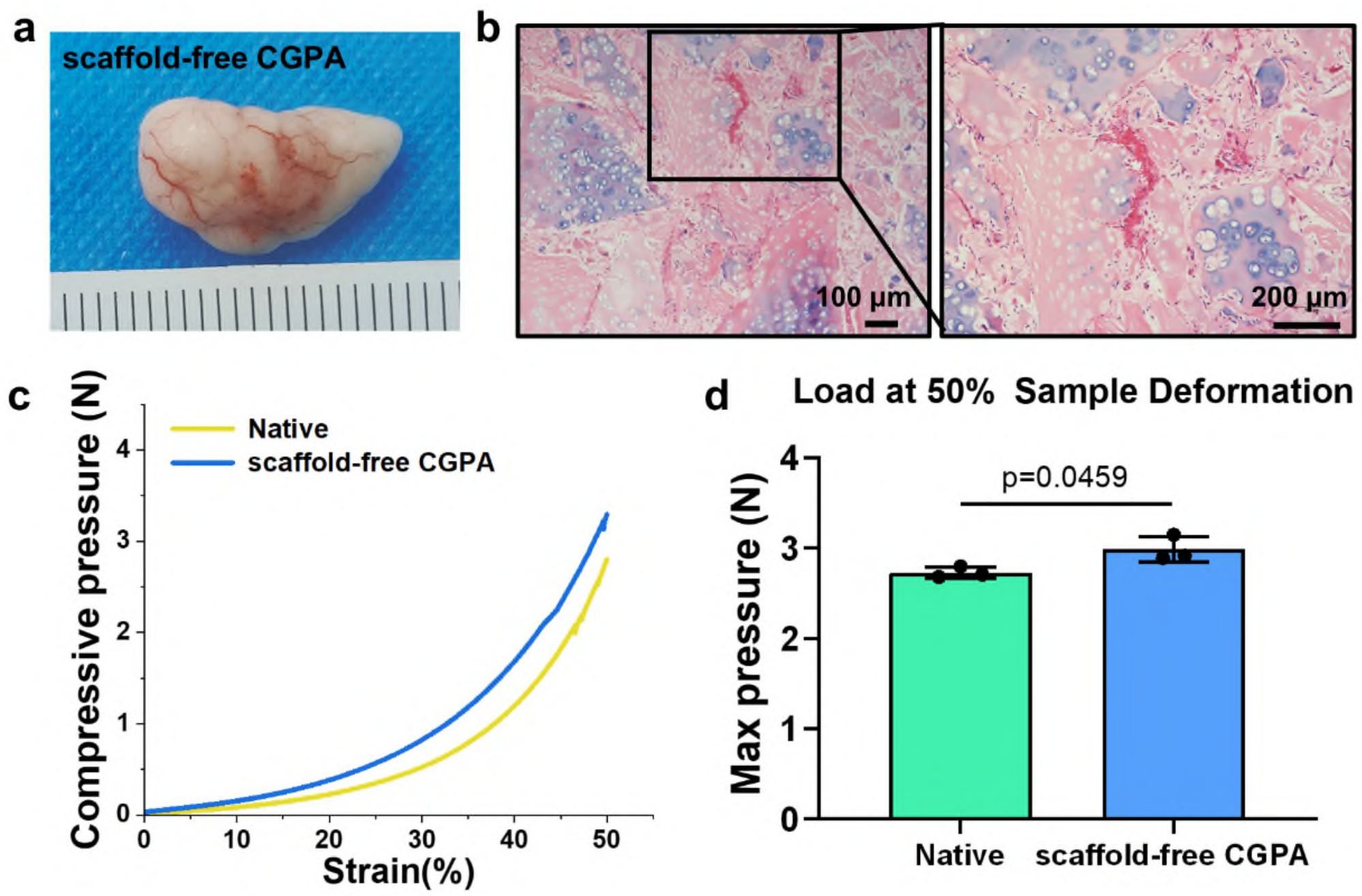

Supplementary Fig. 10 Characterization of scaffold-free CGPA grafts after 2 weeks of prevascularization. **a** Gross image of a scaffold-free CGPA graft showing cartilage formation with superficial vascularization. **b** H&E-stained section demonstrating well-integrated cartilage granules with visible cartilage lacunae and matrix deposition (n = 3 independent experiments with similar results; scale bars: 100 μm, 200 μm). **c** Compressive stress-strain curves of native tracheal cartilage and scaffold-free CGPA grafts. **d** Quantitative analysis of the maximum compressive force at 50% deformation (n = 3 independent samples). Data in d are presented as mean values ± SD. Statistical significance was determined via a two-tailed unpaired Student's t-test. Source data are provided in Source Data file.

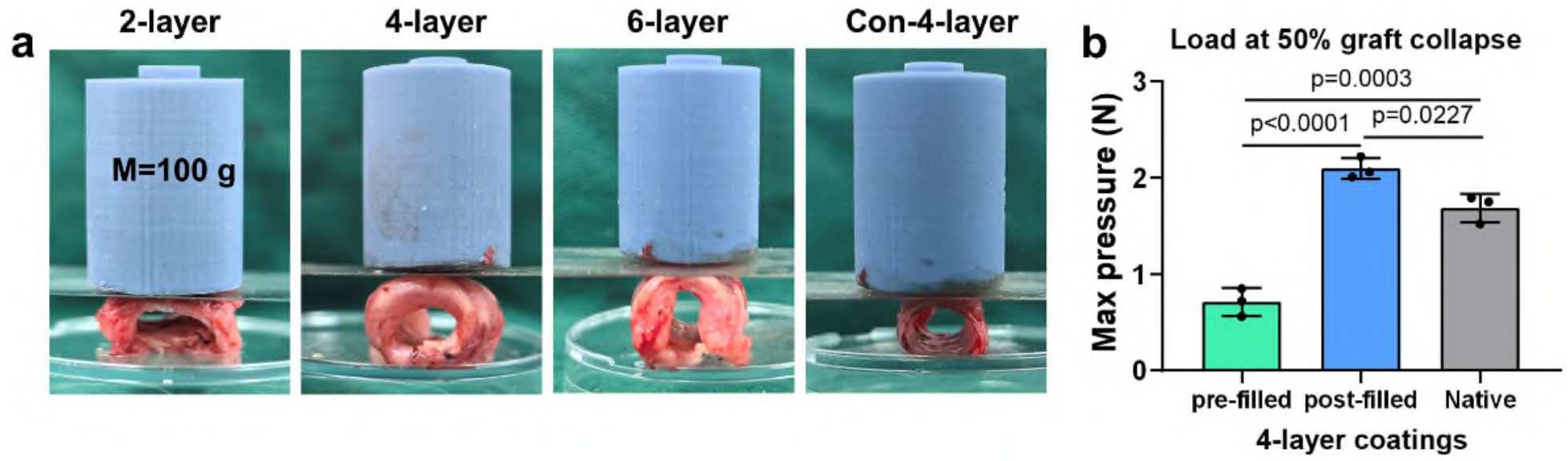

Supplementary Fig. 11 Mechanical characterization of 3D minichannel tracheal grafts. a Representative image of minichannel grafts under a 100 g load. **b** Maximum load at 50% collapse for 4-layer minichannels before filling (pre-filled), after filling (post-filled), and native cartilage (Native). Data in b are presented as mean values ± SD (n = 3 independent samples). Statistical significance was determined via two-sided one-way ANOVA with Tukey's post-hoc test. Source data are provided in Source Data file.

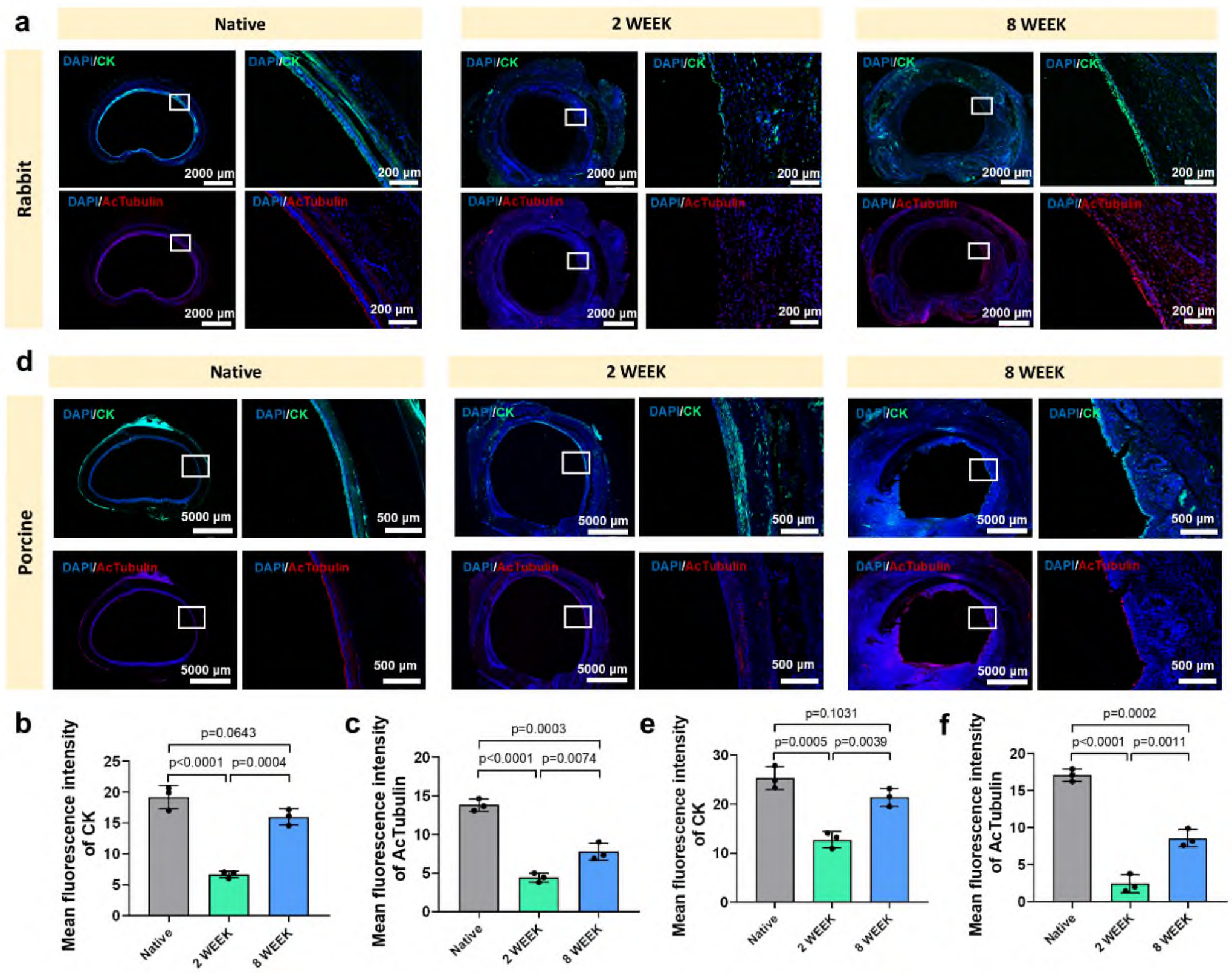

Supplementary Fig. 12 Epithelial marker expression in tracheal grafts. a Immunofluorescence of cytokeratin (CK, green) and AC-tubulin (red) in rabbit grafts: native trachea, graft anastomosis, and midportion (n = 3 independent experiments with similar results; scale bars: 200 μm, 2000 μm). **b-c** Quantification of CK and AC-tubulin fluorescence intensity in rabbit grafts (n = 3 independent biological samples). **d** CK/AC-tubulin staining of pig grafts (scale bars: 500 μm, 5000 μm). **e-f** CK and AC-tubulin intensities in pig grafts (n = 3 independent samples). Data in b-c and e-f are presented as mean values ± SD. Statistical significance was determined via two-sided one-way ANOVA with Tukey's post-hoc test. Source data are provided in Source Data file.

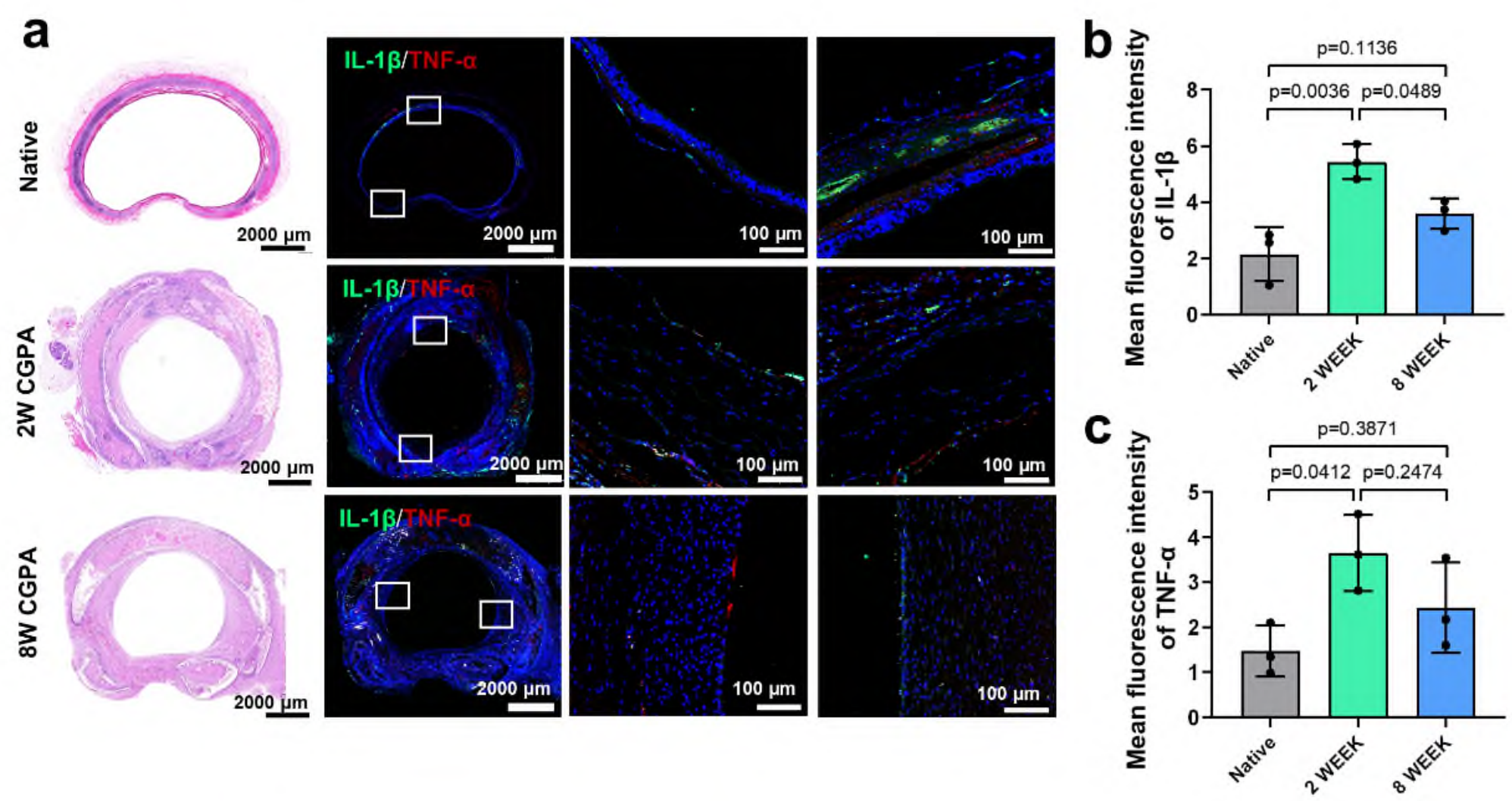

Supplementary Fig. 13 Inflammation marker expression in different tracheal grafts.

a Immunofluorescence for IL-1β (green) and TNF-α (red) in grafts. Native tissue shows baseline signals (n = 3 independent experiments with similar results; scale bars: 2000 μm and 100 μm). The 2W CGPA grafts exhibited mild inflammation. 8W CGPA grafts reveal intense IL-1β/TNF-α in granulation tissue. **b** Quantitative analysis of IL-1β intensity (n = 3 independent biological samples). **c** Quantitative analysis of TNF-α intensity (n = 3 independent biological samples). Data in b-c are presented as mean values ± SD. Statistical significance was determined via two-sided one-way ANOVA with Tukey's post-hoc test. Source data are provided in Source Data file.

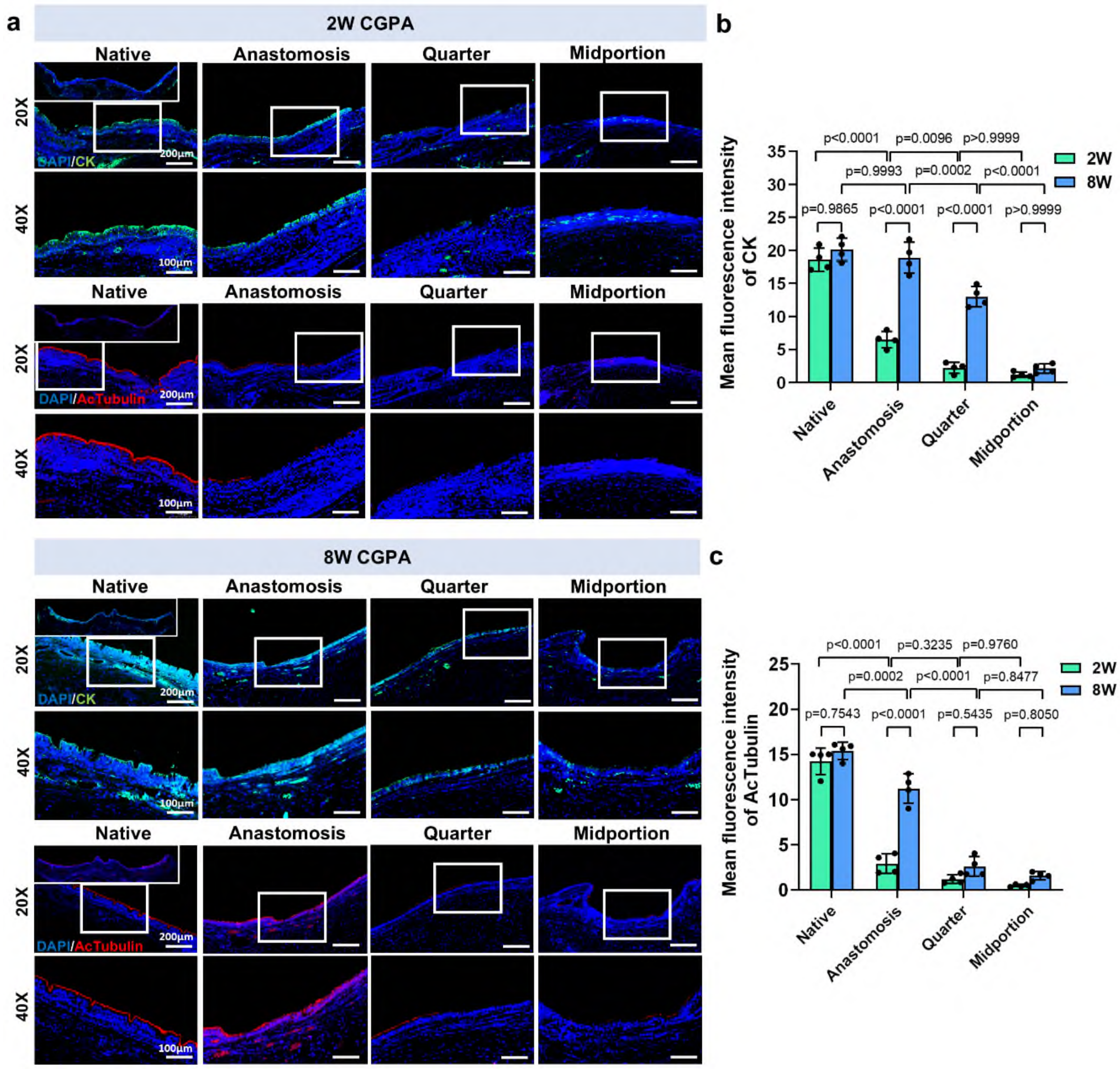

Supplementary Fig. 14 Longitudinal analysis of epithelial regeneration along tracheal grafts.

a Immunofluorescence staining for cytokeratin (CK, green) and acetylated tubulin (Ac-Tubulin, red) along the graft axis at 2- and 8-weeks post-implantation (n = 4 independent experiments with similar results; scale bars: 100 μm, 2000 μm scale bars: 200 μm and 100 μm). **b-c** Quantitative analysis of CK and Ac-Tubulin fluorescence intensity at the anastomosis, quarter, and midportion regions at 2 and 8 weeks (n = 4 independent biological samples). Data in b-c are presented as mean values ± SD. Statistical significance was determined via two-sided one-way ANOVA with Tukey's post-hoc test. Source data are provided in Source Data file.

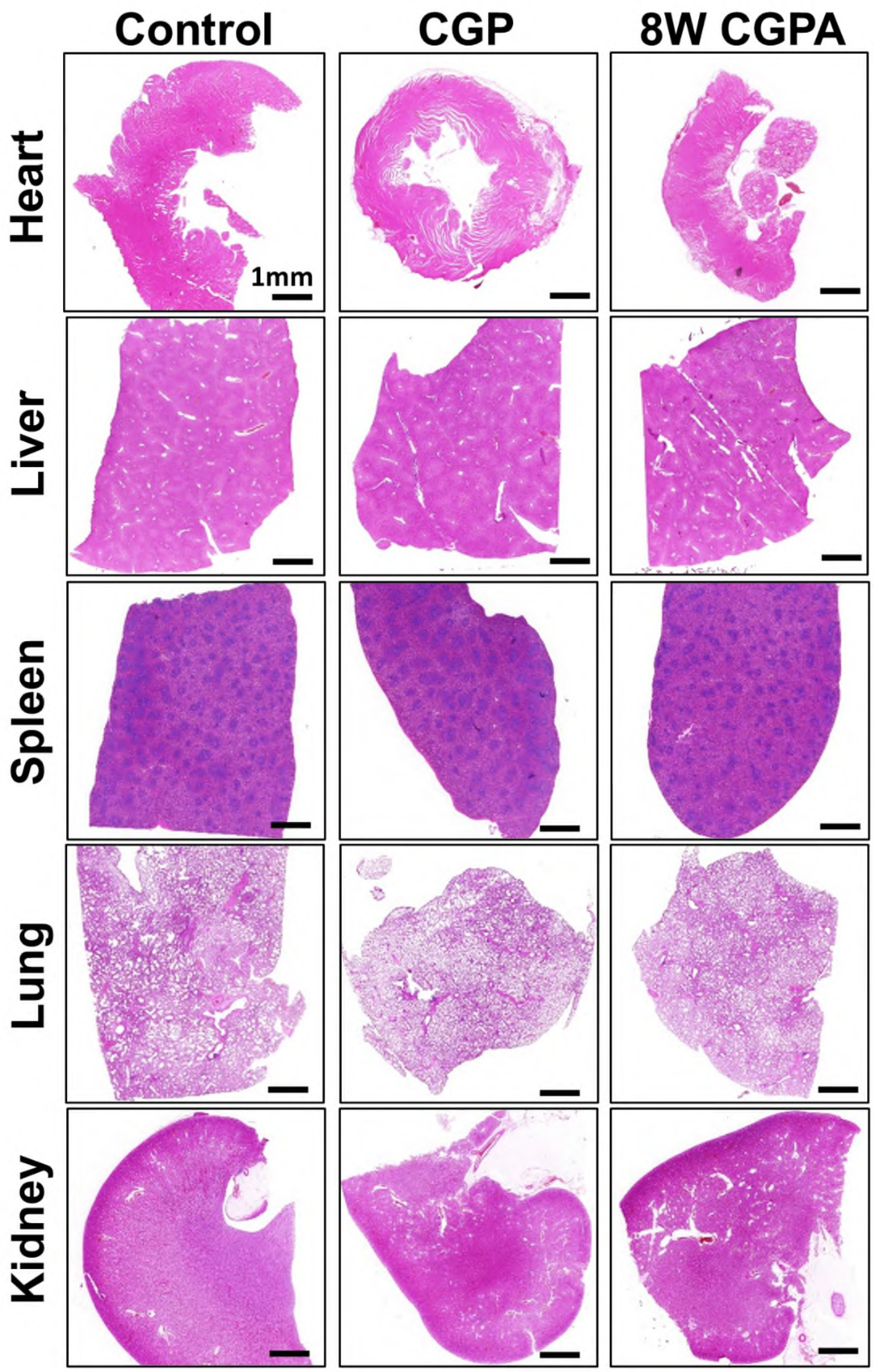

Supplementary Fig. 15 Safety assessment of 3D cartilage granule grafts. Histological analysis of major organs (heart, liver, spleen, lungs, and kidneys) from the control and experimental groups (CGP and CGPA) revealed no significant adverse effects or tissue damage (n = 3 animals with similar results; scale bar: 1 mm).

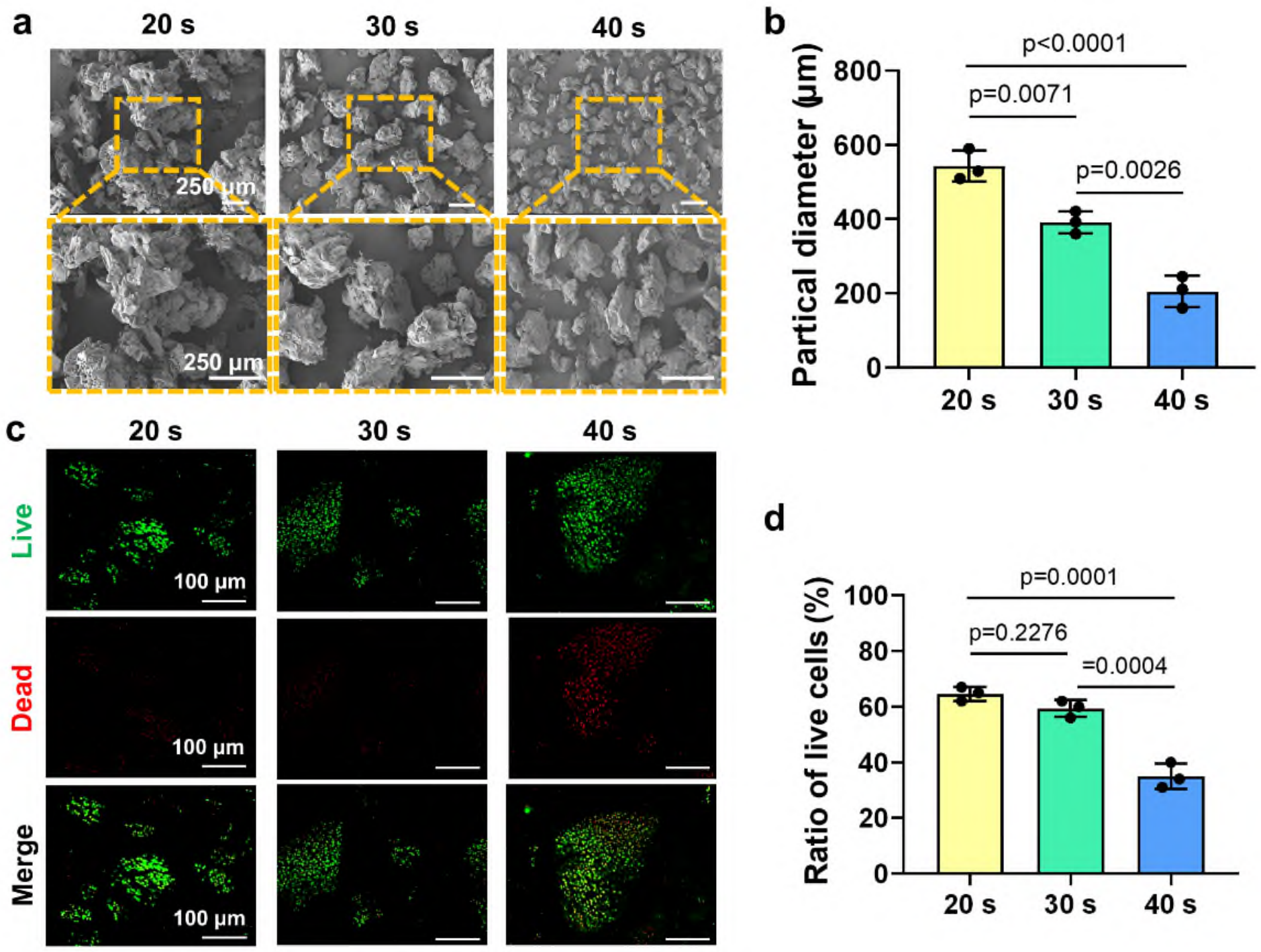

Supplementary Fig. 16 Characterization of porcine rib cartilage granules. a SEM images of microparticles ground for different durations (n = 3 independent experiments with similar results; scale bar: 250 μm). **b** Analysis of the size distribution of microparticles from different groups (n = 3 independent samples). **c** Live/dead cell staining of cartilage granules from each group (n = 3 independent experiments with similar results; scale bar: 100 μm). **d** Quantitative analysis of live cells (n = 3 independent biological samples). Data in b and d are presented as mean values ± SD. Statistical significance was determined via two-sided one-way ANOVA with Tukey's post-hoc test. Source data are provided in Source Data file.

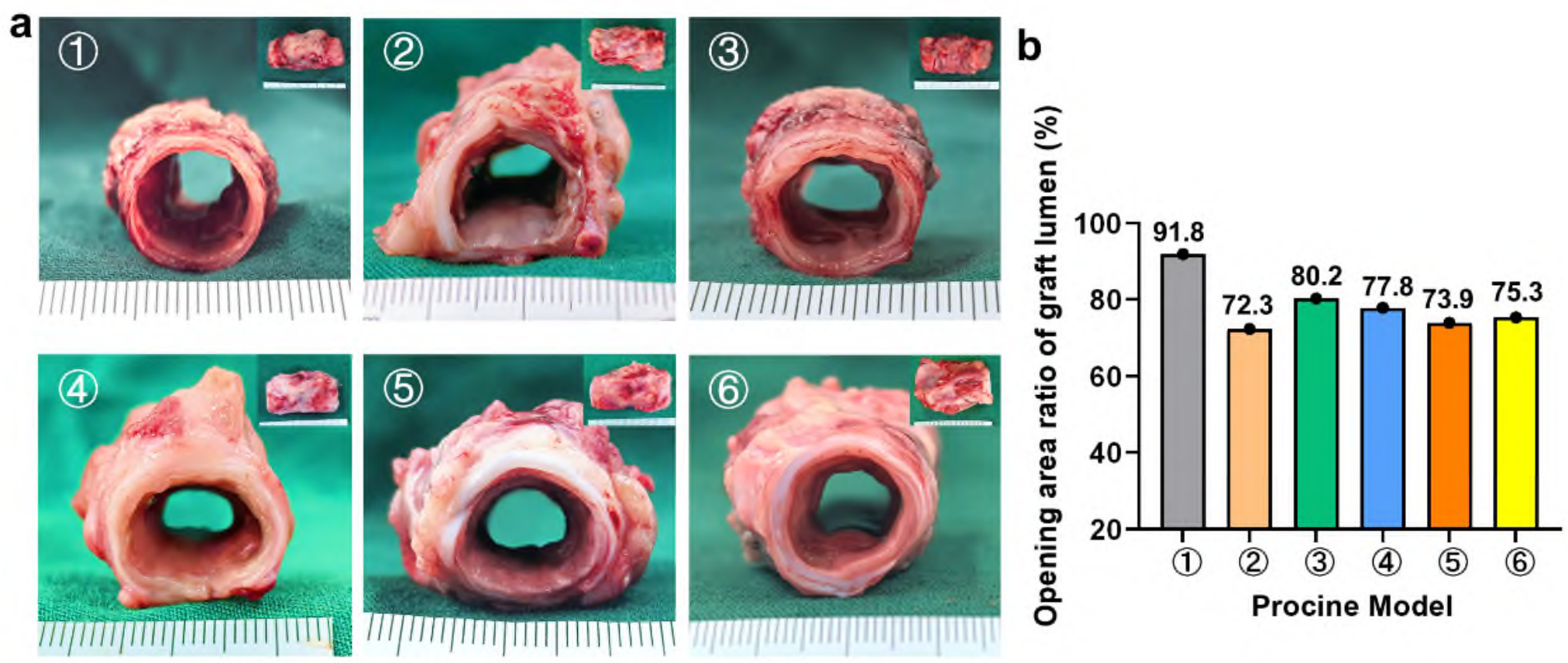

Supplementary Fig. 17 Gross analysis of explanted porcine tracheal grafts.

a Lumen opening images of grafts harvested from six pigs. ①Tracheal graft harvested at 2 weeks, showing no significant granulation tissue or luminal stenosis in the graft. ②-④ Tracheal graft harvested at 8 weeks. ⑤ Tracheal grafts harvested at 12 weeks (86 days). ⑥ Tracheal grafts harvested at 16 weeks (115 days). **b** Quantitative evaluation of the opening area ratio of graft lumens (%) harvested from the six pigs (①-⑥). Source data are provided as a Source Data file.

Part II: Supplementary Table 1

Supplementary Table 1.

Wesley-Croup scoring system for animal models

Severity Classification:

Mild: ≤4 | Moderate: 5--6 | Severe: 7--11 | Respiratory failure: ≥12

Signs, Symptoms, and Indications	Score
Subcostal retractions	
None	0
Mild	1
Moderate	2
Severe	3
Stridor	
None	0
With agitation	1
At rest	2
Cyanosis	
None	0
With agitation	4
At rest	5
Level of Consciousness	
Normal	0
Disoriented	5
Air entry	
Normal	0
Decreased	1
Markedly decreased	2
