# Supplementary material for: 3D minichannel-morphed adipose stem cell-cartilage granule reconstituted tough grafts for repairing segmental tracheal defects in preclinical models
Source: Nat Commun. 2026 Sep 11;17:10735. doi: 10.1038/s41467-026-77738-z (PMC13658815; doi:10.1038/s41467-026-77738-z)
Supplement: Supplementary file 4 [file 41467_2026_77738_MOESM2_ESM.pdf]

Description of Additional Supplementary Files

Supplementary Movie 1 - Demonstration of the plastic performance of a caramel scaffold.

Supplementary Movie 2 - Fabrication process of a C-shaped tracheal scaffold.

Supplementary Movie 3 - Injection performance of cartilage granule composites with varying PRP contents.

Supplementary Movie 4 - Injection and filling process of a C-shaped hollow scaffold.

Supplementary Movie 5 - Prevascularization of a cartilage granule C-shaped scaffold.

Supplementary Movie 6 - 13-Day CGP Group: Bronchoscopy Video

Supplementary Movie 7 - 8-week CGPA Group: Bronchoscopy Video
